# Interventions for myopia control in children: a living systematic review and network meta‐analysis

**DOI:** 10.1002/14651858.CD014758.pub2

**Published:** 2023-02-16

**Authors:** John G Lawrenson, Rakhee Shah, Byki Huntjens, Laura E Downie, Gianni Virgili, Rohit Dhakal, Pavan K Verkicharla, Dongfeng Li, Sonia Mavi, Ashleigh Kernohan, Tianjing Li, Jeffrey J Walline

**Affiliations:** Centre for Applied Vision Research, School of Health & Psychological Sciences City, University of LondonLondonUK; Department of Optometry and Vision SciencesThe University of MelbourneMelbourneAustralia; Department of Neurosciences, Psychology, Drug Research and Child Health (NEUROFARBA)University of FlorenceFlorenceItaly; Centre for Public HealthQueen's University BelfastBelfastUK; Myopia Research Lab, Prof. Brien Holden Eye Research CentreL V Prasad Eye InstituteHyderabadIndia; Department of Ophthalmology, Sichuan Provincial People’s HospitalUniversity of Electronic Science and Technology of ChinaChengduChina; Population Health Sciences InstituteNewcastle UniversityNewcastle upon TyneUK; Department of OphthalmologyUniversity of Colorado Denver Anschutz Medical CampusAuroraCOUSA; College of OptometryThe Ohio State UniversityColumbusOhioUSA

**Keywords:** Child, Humans, Atropine, Atropine/therapeutic use, Myopia, Network Meta-Analysis, Refraction, Ocular, Refractive Errors

## Abstract

**Background:**

Myopia is a common refractive error, where elongation of the eyeball causes distant objects to appear blurred. The increasing prevalence of myopia is a growing global public health problem, in terms of rates of uncorrected refractive error and significantly, an increased risk of visual impairment due to myopia‐related ocular morbidity. Since myopia is usually detected in children before 10 years of age and can progress rapidly, interventions to slow its progression need to be delivered in childhood.

**Objectives:**

To assess the comparative efficacy of optical, pharmacological and environmental interventions for slowing myopia progression in children using network meta‐analysis (NMA). To generate a relative ranking of myopia control interventions according to their efficacy. To produce a brief economic commentary, summarising the economic evaluations assessing myopia control interventions in children. To maintain the currency of the evidence using a living systematic review approach.

**Search methods:**

We searched CENTRAL (which contains the Cochrane Eyes and Vision Trials Register), MEDLINE; Embase; and three trials registers. The search date was 26 February 2022.

**Selection criteria:**

We included randomised controlled trials (RCTs) of optical, pharmacological and environmental interventions for slowing myopia progression in children aged 18 years or younger. Critical outcomes were progression of myopia (defined as the difference in the change in spherical equivalent refraction (SER, dioptres (D)) and axial length (mm) in the intervention and control groups at one year or longer) and difference in the change in SER and axial length following cessation of treatment ('rebound').

**Data collection and analysis:**

We followed standard Cochrane methods. We assessed bias using RoB 2 for parallel RCTs. We rated the certainty of evidence using the GRADE approach for the outcomes: change in SER and axial length at one and two years. Most comparisons were with inactive controls.

**Main results:**

We included 64 studies that randomised 11,617 children, aged 4 to 18 years. Studies were mostly conducted in China or other Asian countries (39 studies, 60.9%) and North America (13 studies, 20.3%). Fifty‐seven studies (89%) compared myopia control interventions (multifocal spectacles, peripheral plus spectacles (PPSL), undercorrected single vision spectacles (SVLs), multifocal soft contact lenses (MFSCL), orthokeratology, rigid gas‐permeable contact lenses (RGP); or pharmacological interventions (including high‐ (HDA), moderate‐ (MDA) and low‐dose (LDA) atropine, pirenzipine or 7‐methylxanthine) against an inactive control. Study duration was 12 to 36 months. The overall certainty of the evidence ranged from very low to moderate.

Since the networks in the NMA were poorly connected, most estimates versus control were as, or more, imprecise than the corresponding direct estimates. Consequently, we mostly report estimates based on direct (pairwise) comparisons below.

At one year, in 38 studies (6525 participants analysed), the median change in SER for controls was −0.65 D. The following interventions may reduce SER progression compared to controls: HDA (mean difference (MD) 0.90 D, 95% confidence interval (CI) 0.62 to 1.18), MDA (MD 0.65 D, 95% CI 0.27 to 1.03), LDA (MD 0.38 D, 95% CI 0.10 to 0.66), pirenzipine (MD 0.32 D, 95% CI 0.15 to 0.49), MFSCL (MD 0.26 D, 95% CI 0.17 to 0.35), PPSLs (MD 0.51 D, 95% CI 0.19 to 0.82), and multifocal spectacles (MD 0.14 D, 95% CI 0.08 to 0.21). By contrast, there was little or no evidence that RGP (MD 0.02 D, 95% CI −0.05 to 0.10), 7‐methylxanthine (MD 0.07 D, 95% CI −0.09 to 0.24) or undercorrected SVLs (MD −0.15 D, 95% CI −0.29 to 0.00) reduce progression.

At two years, in 26 studies (4949 participants), the median change in SER for controls was −1.02 D. The following interventions may reduce SER progression compared to controls: HDA (MD 1.26 D, 95% CI 1.17 to 1.36), MDA (MD 0.45 D, 95% CI 0.08 to 0.83), LDA (MD 0.24 D, 95% CI 0.17 to 0.31), pirenzipine (MD 0.41 D, 95% CI 0.13 to 0.69), MFSCL (MD 0.30 D, 95% CI 0.19 to 0.41), and multifocal spectacles  (MD 0.19 D, 95% CI 0.08 to 0.30). PPSLs (MD 0.34 D, 95% CI −0.08 to 0.76) may also reduce progression, but the results were inconsistent. For RGP, one study found a benefit and another found no difference with control. We found no difference in SER change for undercorrected SVLs (MD 0.02 D, 95% CI −0.05 to 0.09).

At one year, in 36 studies (6263 participants), the median change in axial length for controls was 0.31 mm. The following interventions may reduce axial elongation compared to controls: HDA (MD −0.33 mm, 95% CI −0.35 to 0.30), MDA (MD −0.28 mm, 95% CI −0.38 to −0.17), LDA (MD −0.13 mm, 95% CI −0.21 to −0.05), orthokeratology (MD −0.19 mm, 95% CI −0.23 to −0.15), MFSCL (MD −0.11 mm, 95% CI −0.13 to −0.09), pirenzipine (MD −0.10 mm, 95% CI −0.18 to −0.02), PPSLs (MD −0.13 mm, 95% CI −0.24 to −0.03), and multifocal spectacles (MD −0.06 mm, 95% CI −0.09 to −0.04). We found little or no evidence that RGP (MD 0.02 mm, 95% CI −0.05 to 0.10), 7‐methylxanthine (MD 0.03 mm, 95% CI −0.10 to 0.03) or undercorrected SVLs (MD 0.05 mm, 95% CI −0.01 to 0.11) reduce axial length.

At two years, in 21 studies (4169 participants), the median change in axial length for controls was 0.56 mm. The following interventions may reduce axial elongation compared to controls: HDA (MD −0.47mm, 95% CI −0.61 to −0.34), MDA (MD −0.33 mm, 95% CI −0.46 to −0.20), orthokeratology (MD −0.28 mm, (95% CI −0.38 to −0.19), LDA (MD −0.16 mm, 95% CI −0.20 to  −0.12), MFSCL (MD −0.15 mm, 95% CI −0.19 to −0.12), and multifocal spectacles (MD −0.07 mm, 95% CI −0.12 to −0.03). PPSL may reduce progression (MD −0.20 mm, 95% CI −0.45 to 0.05) but results were inconsistent. We found little or no evidence that undercorrected SVLs (MD ‐0.01 mm, 95% CI −0.06 to 0.03) or RGP (MD 0.03 mm, 95% CI −0.05 to 0.12) reduce axial length.

There was inconclusive evidence on whether treatment cessation increases myopia progression. Adverse events and treatment adherence were not consistently reported, and only one study reported quality of life.

No studies reported environmental interventions reporting progression in children with myopia, and no economic evaluations assessed interventions for myopia control in children.

**Authors' conclusions:**

Studies mostly compared pharmacological and optical treatments to slow the progression of myopia with an inactive comparator. Effects at one year provided evidence that these interventions may slow refractive change and reduce axial elongation, although results were often heterogeneous. A smaller body of evidence is available at two or three years, and uncertainty remains about the sustained effect of these interventions. Longer‐term and better‐quality studies comparing myopia control interventions used alone or in combination are needed, and improved methods for monitoring and reporting adverse effects.

## Plain language summary

**Interventions to slow the progression of short‐sightedness in children**

**Key messages**

• Medications such as atropine, given as eye drops, can slow the progression of short‐ or near‐sightedness (myopia) in children, and also reduce elongation of the eyeball due to myopia. Higher doses of atropine are most effective. We are uncertain about the effects of lower doses of atropine.

• Several treatments, including special types of lenses in eye glasses as well as contact lenses, may slow the progression of short‐sightedness, but their effect is still uncertain and there is insufficient information on the risk of unwanted effects.

• It is also unclear whether the reported benefit of medications or lenses on myopia progression is maintained over the years.

**What is short‐sightedness?**

Short‐sightedness (or near‐sightedness or myopia) means people struggle to see objects that are far away clearly, while objects that are near remain clear. It is very common worldwide, and  affects more than half of children in China and South‐East Asia. Short‐sightedness may impair many aspects of life, including educational and occupational activities. Moreover, short‐sighted people have longer eyes, which means that the retina is  stretched. This puts the eye at greater risk of eye diseases such as glaucoma, maculopathy and retinal detachment later in life.

**How is short‐sightedness treated?**

Although conventional eyeglasses or contact lenses are able to correct short sight, they do not slow its progression. A number of optical treatments (glasses and contact lenses) and medications are available that aim to slow the progression of short‐sightedness. But they need to be given in childhood, when short‐sightedness progresses most quickly. Medications such as atropine eye drops may be effective, but can cause increased sensitivity to glare and cause problems when reading, especially at higher doses. Special eyeglasses are also available, that include more than one focus power within the lens (multifocal or peripheral‐plus lenses). These can also be provided as soft contact lenses. Other contact lenses, called orthokeratology, aim to temporarily change the shape of the eye surface and are worn during sleep and removed during the day. Both soft contact lenses and orthokeratology may increase the risk of infections to the eye surface

**What did we want to find out?**

We aimed to find out whether medications used as eye drops, and special lenses in eyeglasses or contact lenses, can slow the progression of myopia, as well as the elongation of the eyeball. We also documented the risk of unwanted effects of such interventions.

**What did we do?**

We searched for studies that tested medications and lenses aiming to slow progression of short‐sightedness in children, compared with a control group or with other medications and lenses. The control group generally received a placebo (sham) treatment or single vision eye glasses or contact lenses.

**What did we find?**

• Higher doses of atropine may reduce the progression of short‐sightedness, but the effect of low‐dose atropine could be small and is uncertain.

• Based on short‐term studies, orthokeratology is the most effective of the optical treatments in slowing elongation of the eyeball. These lenses were often difficult to tolerate, however, with more than half of children not completing the treatment in some studies.

• Other types of contact lenses, known as multifocal soft contact lenses, may also reduce the progression of short‐sightedness, but, again, we remain uncertain about their beneficial effects.

• Unwanted effects associated with myopia control interventions were not consistently reported. Eye discomfort in bright light and blurred near vision were the most common treatment‐related unwanted effects in studies using atropine. Lower doses of atropine appear to have fewer unwanted effects.

• Although studies that tested contact lenses did not report any serious unwanted effects, it is unclear what the true rate of unwanted effects would be for children outside a research study or when wearing contact lenses for longer periods.

**What are the limitations of the evidence?**

Most of the evidence came from studies conducted in ways that may have introduced errors into their results, and potential unwanted effects were not well reported. The majority of the studies followed participants up for 2 years or less and therefore there is insufficient evidence on whether incremental benefits are found over the years and whether the effects are sustained.

**How up to date is the evidence?**

This review is up‐to‐date to February 2022.

## Summary of findings

**Summary of findings 1:** Summary of findings 1: change in refractive error at 1 year

**Interventions for myopia control in children: a living systematic review and network meta‐analysis**				
**Population:** children with progressive myopia (38 studies, 6525 participants in analyses) **Interventions:** optical and pharmacological **Comparator:** control (36 studies, 2846 participants). Control arms for optical interventions are either single vision spectacles or contact lenses. Placebo eyedrops were the usual comparator for pharmacological interventions **Outcome:** progression of myopia (difference in change in spherical equivalent refraction (SER)) at 1 year (dioptres) **Setting:** primary eye care **Assumed control risk:** median change in SER in control arms at 1 year −0.65D **Equivalence criterion:** difference in change in spherical equivalent less than 0.25 D				
**Treatment (vs control)**	**Number of studies in the treatment arm (participants)**	**Corresponding intervention risk MD (95%CI). ** **Direct estimates from pairwise MA**	**Corresponding intervention risk MD (95%CI).** **Estimates from NMA**	**Certainty of evidence **
High‐dose atropine (≥ 0.5%)	3 (512)	0.90 (0.62 to 1.18)	0.89 (0.65 to 1.12)	Moderate^a^
Moderate‐dose atropine (0.1% to < 0.5%)	2 (254)	‐	0.65 (0.27 to 1.03)	Moderate^a^
Low‐dose atropine (< 0.1%)	4 (497)	0.38 (0.10 to 0.66)	0.43 (0.24 to 0.61)	Very low^b^
Pirenzepine	2 (210)	0.32 (0.15 to 0.49)	0.27 (−0.13 to 0.67)	Very low^b^
7‐methyxanthine	1 (77)	0.07 (−0.09 to 0.24)	0.07 (−0.33 to 0.48)	Low^c^
Multifocal soft contact lenses	8 (712)	0.26 (0.17 to 0.35)	0.23 (0.09 to 0.37)	Very low^b^
Rigid gas‐permeable contact lenses	2 (178)	0.02 (−0.05 to 0.10)	0.17 (−0.12 to 0.46)	Very low^b^
Peripheral plus spectacle lenses	5 (480)	0.51 (0.19 to 0.82)	0.28 (0.05 to 0.51)	Very low^b^
Multifocal spectacle lenses	9 (729)	0.14 (0.08 to 0.21)	0.14 (−0.04 to 0.32)	Low^c^
Undercorrected single vision spectacles	2 (72)	−0.15 (−0.29 to 0.00)	−0.15 (−0.45 to 0.15)	Low^c^
**GRADE Working Group grades of evidence** **High‐certainty:** we are very confident that the true effect lies close to that of the estimate of the effect. **Moderate‐certainty:** we are moderately confident in the effect estimate; the true effect is likely to be close to the estimate of effect, but there is a possibility that it is substantially different. **Low‐certainty:** our confidence in the effect estimate is limited; the true effect may be substantially different from the estimate of the effect. **Very low‐certainty:** we have very little confidence in the effect estimate; the true effect is likely to be substantially different from the estimate of effect.				
**Explanation** Negative mean differences for changes in refractive error represent faster progression of myopia in the intervention group compared to progression in the control group. Measurement of refractive error is not an appropriate outcome in orthokeratology (ortho‐K) studies. Overnight wear of ortho‐K lenses flattens the central cornea and temporally reduces refractive error. It is therefore not possible to assess the true progression of refractive error without ceasing lens wear for a period of time to allow the cornea to return to its pre‐treatment state **CI:** confidence interval; **MA:** meta‐analysis**; MD:** mean difference; **NMA:** network meta‐analysis** **				

**Reasons for downgrade** ^a^Downgraded one level for risk of bias, not downgraded for inconsistency since all studies show clinically important effects. ^b.^Downgraded one level for risk of bias, imprecision and inconsistency. ^c^Downgraded one level for risk of bias and imprecision In each case, downgrading due to risk of bias was due to concerns arising from the randomisation process and in the selection of the reporting of the results; downgrading for imprecision was due to a confidence interval that included small and clinically unimportant effects or optimal information size not met (using fewer than 400 participants as a 'rule of thumb'); downgrading for inconsistency was due to substantial heterogeneity.

**Summary of findings 2:** Summary of findings 2: change in refractive error at 2 years

**Interventions for myopia control in children: a living systematic review and network meta‐analysis**				
**Population:** children with progressive myopia (26 studies, 4949 participants in the analysis) **Interventions:** optical and pharmacological **Comparator:** control (24 studies, 2282 participants). Control arms for optical interventions are either single vision spectacles or contact lenses. Placebo eyedrops were the usual comparator for pharmacological interventions **Outcome:** progression of myopia (difference in change in spherical equivalent refraction (SER)) at 2 years (dioptres) **Setting:** primary eye care **Assumed control risk:** median change in SER in control arms at 2 years −1.02 D **Equivalence criterion:** difference in change in spherical equivalent less than 0.25 D				
**Treatment (vs control)**	**Number of studies in the treatment arm (participants)**	**Corresponding intervention risk MD (95%CI)** **Direct estimates from pairwise MA**	**Corresponding intervention risk MD (95%CI)** **Estimates from NMA**	**Certainty of evidence **
High‐dose atropine (≥ 0.5%)	2 (428)	1.26 (1.17 to 1.36)	0.74 (0.44 to 1.05)	Moderate^a^
Moderate‐dose atropine (0.1% to < 0.5%)	2 (247)	‐	0.45 (0.08 to 0.83)	Low^b^
Low‐dose atropine (< 0.1%)	2 (249)	0.24 (0.17 to 0.31)	0.31 (0.07 to 0.56)	Low^b^
Pirenzepine	1 (53)	0.41 (0.13 to 0.69)	0.41 (−0.05 to 0.87)	Low^b^
Multifocal soft contact lenses	5 (540)	0.30 (0.19 to 0.41)	0.31 (0.12 to 0.49)	Low^b^
Rigid gas‐permeable contact lenses	2 (154)	One study showed no difference and the other a beneficial effect	0.22 (−0.09 to 0.53)	Very low^c^
Peripheral plus spectacle lenses	2 (188)	0.34 (−0.08 to 0.76)	0.34 (0.05 to 0.63)	Very low^c^
Multifocal spectacle lenses	8 (696)	0.19 (0.08 to 0.30)	0.19 (0.03 to 0.36)	Low^b^
Undercorrected single vision spectacles	2 (122)	0.02 (−0.05 to 0.09)	−0.07 (−0.36 to 0.22)	Very low^c^
**GRADE Working Group grades of evidence** **High‐certainty:** we are very confident that the true effect lies close to that of the estimate of the effect. **Moderate‐certainty:** we are moderately confident in the effect estimate; the true effect is likely to be close to the estimate of effect, but there is a possibility that it is substantially different. **Low‐certainty:** our confidence in the effect estimate is limited; the true effect may be substantially different from the estimate of the effect. **Very low‐certainty:** we have very little confidence in the effect estimate; the true effect is likely to be substantially different from the estimate of effect.				
**Explanation** Negative mean differences (MDs) for changes in refractive error represent faster progression of myopia in the intervention group compared to progression in the control group. Measurement of refractive error is not an appropriate outcome in orthokeratology (ortho‐K) studies. Overnight wear of ortho‐K lenses flattens the central cornea and temporally reduces refractive error. It is therefore not possible to assess the true progression of refractive error without ceasing lens wear for a period of time to allow the cornea to return to its pre‐treatment state **CI:** confidence interval; **MA:** meta‐analysis**; MD:** mean difference; **NMA:** network meta‐analysis** **				

**Reasons for downgrade** ^a^Downgraded one level for risk of bias, not downgraded for inconsistency since all studies show clinically important effects. ^b^Downgraded one level for risk of bias and imprecision. ^c^Downgraded one level for risk of bias, imprecision and inconsistency In each case, downgrading due to risk of bias was due to concerns arising from the randomisation process and in the selection of the reporting of the results; downgrading for imprecision was due to a confidence interval that included small and clinically unimportant effects or optimal information size not met (using fewer than 400 participants as a 'rule of thumb'); downgrading for inconsistency was due to substantial heterogeneity.

**Summary of findings 3:** Summary of findings 3: change in axial length at 1 year

**Interventions for myopia control in children: a living systematic review and network meta‐analysis**				
**Population:** children with progressive myopia (36 studies, 6263 participants) in the analysis **Interventions:** optical and pharmacological **Comparator:** control (35 studies, 2732 participants). Control arms for optical interventions are either single vision spectacles or contact lenses. Placebo eyedrops were the usual comparator for pharmacological interventions **Setting:** primary eye care **Outcome:** difference in change in axial length at 1 year (mm) **Assumed control risk: median** change in axial length in control arms at 1 year 0.31 mm **Equivalence criterion:** difference in change in axial length less than 0.1 mm				
**Treatment (vs control)**	**Number of studies in the treatment arm** **(participants)**	**Corresponding intervention risk MD (95%CI)** **Direct estimates from pairwise MA**	**Corresponding intervention risk MD (95%CI)** **Estimates from NMA**	**Certainty of evidence **
High‐dose atropine (≥ 0.5%)	3 (512)	−0.33 (−0.35 to −0.30)	−0.32 (−0.38 to −0.26)	Moderate^a^
Moderate‐dose atropine (0.1% to < 0.5%)	1 (155)	‐	−0.28 (−0.38 to −0.17)	Moderate^a^
Low‐dose atropine (< 0.1%)	4 (497)	−0.13 (−0.21 to −0.05)	−0.14 (−0.19 to −0.08)	Very low^b^
Pirenzepine	2 (210)	−0.10 (−0.18 to −0.02)	−0.08 (−0.19 to 0.02)	Very low^b^
7‐methylxanthine	1 (35)	−0.03 (−0.10 to 0.03)	−0.03 (−0.15 to 0.08)	Low^c^
Orthokeratology	7 (402)	−0.19 (−0.23 to −0.15)	−0.18 (−0.24 to −0.12)	Moderate^a^
Multifocal soft contact lenses	8 (712)	−0.11 (−0.13 to −0.09)	−0.11 (−0.14 to −0.07)	Low^c^
Rigid gas‐permeable contact lenses	2 (176)	0.02 (−0.05 to 0.10)	0.02 (−0.07 to 0.12)	Low^c^
Peripheral plus spectacle lenses	3 (340)	−0.13 (−0.24 to −0.03)	−0.14 (−0.20 to −0.07)	Very low^b^
Multifocal spectacle lenses	4 (445)	−0.06 (−0.09 to −0.04)	−0.04 (−0.16 to 0.08)	Low^c^
Undercorrected single vision spectacles	1 (47)	0.05 (−0.01 to 0.11)	0.05 (−0.06 to 0.16)	Low^c^
**GRADE Working Group grades of evidence** **High‐certainty:** we are very confident that the true effect lies close to that of the estimate of the effect. **Moderate‐certainty:** we are moderately confident in the effect estimate; the true effect is likely to be close to the estimate of effect, but there is a possibility that it is substantially different. **Low‐certainty:** our confidence in the effect estimate is limited; the true effect may be substantially different from the estimate of the effect. **Very low‐certainty:** we have very little confidence in the effect estimate; the true effect is likely to be substantially different from the estimate of effect.				
**Explanation** For the measurement of changes in axial length, negative mean differencess for changes in axial length represent faster axial elongation in the control group compared to the intervention group. **CI:** confidence interval; **MA:** meta‐analysis**; MD:** mean difference; **NMA:** network meta‐analysis** **				

**Reasons for downgrade** ^a^Downgraded one level for risk of bias. ^b^Downgraded one level for risk of bias, imprecision and inconsistency. ^c^Downgraded one level for risk of bias and imprecision. In each case, downgrading due to risk of bias was due to concerns arising from the randomisation process and in the selection of the reporting of the results; downgrading for imprecision was due to a confidence interval that included small and clinically unimportant effects or optimal information size not met (using fewer than than 400 participants as a 'rule of thumb'); downgrading for inconsistency was due to substantial heterogeneity

**Summary of findings 4:** Summary of findings 4: change in axial length at 2 years

**Interventions for myopia control in children: a living systematic review and network meta‐analysis**				
**Population:** children with progressive myopia (21 studies, 4169 participants in the analysis) **Interventions:** optical and pharmacological **Comparator:** control (20 studies, 1894 participants). Control arms for optical interventions are either single vision spectacles or contact lenses. Placebo eyedrops are the usual comparator for pharmacological interventions. **Outcome:** median change in axial length in control arms at 2 years **Setting:** primary eye care **Assumed control risk:** change in axial length at 2 years 0.56 mm **Equivalence criterion:** difference in change in axial length less than 0.1 mm				
**Treatment (vs control)**	**Number of studies in the treatment arm** **(participants)**	**Corresponding intervention risk MD (95%CI)** **Direct estimates from pairwise MA**	**Corresponding intervention risk MD (95%CI) ** **Estimates from NMA**	**Certainty of evidence **
High‐dose atropine (≥ 0.5%)	2 (428)	−0.47 (−0.61 to −0.34)	−0.36 (−0.46 to −0.26)	Moderate^a^
Moderate‐dose atropine (0.1% to < 0.5%)	1 (144)	‐	−0.33 (−0.46 to −0.20)	Moderate^a^
Low‐dose atropine (< 0.1%)	2 (249)	−0.16 (−0.20 to −0.12)	−0.17 (−0.25 to −0.10)	Low^b^
Orthokeratology	2 (49)	−0.28 (−0.38 to −0.19)	−0.29 (−0.41 to −0.16)	Moderate^a^
Multifocal soft contact lenses	5 (540)	−0.15 (−0.19 to −0.12)	−0.16 (−0.22 to −0.10)	Moderate^a^
Rigid gas‐permeable contact lenses	2 (154)	0.03 (−0.05 to 0.12)	0.03 (−0.08 to 0.15)	Low^b^
Peripheral plus spectacle lenses	2 (188)	−0.20 (−0.45 to 0.05)	−0.23 (−0.33 to −0.12)	Very low^c^
Multifocal spectacle lenses	3 (404)	−0.07 (−0.12 to −0.03)	−0.09 (−0.17 to −0.01)	Low^b^
Undercorrected single vision spectacles	2 (122)	−0.01 (−0.06 to 0.03)	0.01 (−0.09 to 0.10)	Low^b^
**GRADE Working Group grades of evidence** **High‐certainty:** we are very confident that the true effect lies close to that of the estimate of the effect. **Moderate‐certainty:** we are moderately confident in the effect estimate; the true effect is likely to be close to the estimate of effect, but there is a possibility that it is substantially different. **Low‐certainty:** our confidence in the effect estimate is limited; the true effect may be substantially different from the estimate of the effect. **Very low‐certainty:** we have very little confidence in the effect estimate; the true effect is likely to be substantially different from the estimate of effect.				
**Explanation** For the measurement of changes in axial length, negative MDs for changes in axial length represent faster axial elongation in the control group compared to the intervention group **CI:** confidence interval; **MA:** meta‐analysis**; MD:** mean difference; **NMA:** network meta‐analysis** **				

**Reasons for downgrade** ^a^Downgraded one level for risk of bias. ^b^Downgraded one level for risk of bias and imprecision. ^c^Downgraded one level for risk of bias, imprecision and inconsistency. In each case, downgrading due to risk of bias was due to concerns arising from the randomisation process and in the selection of the reporting of the results; downgrading for imprecision was due to a confidence interval that included small and clinically unimportant effects or optimal information size not met (using fewer than 400 participants as a 'rule of thumb'); downgrading for inconsistency was due to substantial heterogeneity.

## Background

### Description of the condition

Myopia, or short‐ or near‐sightedness, is a common refractive anomaly of the eye that occurs when parallel rays of light are brought to a focus in front of the retina with accommodation at rest, causing distant objects to appear blurred and near objects to remain clear (Morgan 2012). Myopia most often results from the eyeball being too long (i.e. there is excessive axial elongation), but can also occur when the image‐forming structures of the eye are too strong (Flitcroft 2019).

The prevalence of myopia shows significant age, ethnic and regional variation (Rudnicka 2016). Currently, 30% to 50% of adults in the USA and Europe have myopia (Dolgin 2015). Myopia is already reaching 'epidemic' proportions in children and young adults in urban areas of East and South East Asia, with over 80% of children being myopic by the time they complete their high school education (Dolgin 2015). If current trends continue, it is estimated that by 2050 there will be approximately 5 billion (5000 million) people with myopia (i.e. about 50% of the world's population), with around 10% having high myopia (when defined as a spherical equivalent of −5.00 dioptres (D) or worse) (Holden 2016).

The aetiology of myopia involves a complex interaction between environmental and genetic factors. Although genetic inheritance is a well‐established predisposing factor for myopia, genetic factors cannot explain the rapidly rising prevalence of the condition (Williams 2019). A Mendelian randomisation study, using the UK Biobank cohort, provided strong evidence for the cumulative effect of additional years in education on myopia development (Mountjoy 2018). Mendelian randomisation is a statistical approach that uses genetics to provide information about the relationship between an exposure and outcome. This study estimated that for each additional year in education, myopic spherical equivalent increased by −0.27 D. Evidence from a number of observational studies further supports the causal association between environmental and social factors and myopia development (Morgan 2018).

Epidemiological studies have shown that myopia is an established risk factor for a number of ocular pathologies, including cataract, glaucoma and retinal detachment (Flitcroft 2012). Although myopia‐related complications can occur irrespective of age and degree of myopia (Dhakal 2018), the excessive axial elongation associated with higher degrees of myopia causes biomechanical stretching of the outer coat of the eye, increasing the risk of sight‐threatening pathologies such as posterior staphyloma and myopic maculopathy (Saw 2005; Verkicharla 2015). A meta‐analysis of population studies reporting blindness and visual impairment due to myopic maculopathy (Fricke 2018), estimated that in 2015, approximately 10 million people had visual impairment due to myopic macular degeneration, of whom three million were blind. Although the sight‐threatening pathologies associated with myopia usually occur later in life, the underlying myopia develops during childhood and therefore interventions to reduce the progression of myopia have the potential to reduce future visual impairment.

### Description of the intervention

Most cases of myopia develop during childhood and the prevalence of myopia begins to increase noticeably after the age of six years (McCullough 2016). Progression rates vary significantly, with rates in Asian children being approximately 0.20 D per year faster than their age‐matched European counterparts (Donovan 2012). Since myopia tends to stabilise in late adolescence, interventions to slow myopia progression need to be delivered in childhood.

Interventions to slow progression of myopia can be grouped into three broad categories: optical, pharmacological and environmental (Wildsoet 2019). Optical interventions include a variety of spectacle and contact lens designs. Spectacles are the least invasive and most accessible method for potentially slowing myopia progression. Spectacle options include refractive under‐correction, bifocal and progressive addition lenses and, more recently, specialised 'myopia control' designs. Soft multifocal and approved myopia control contact lenses are increasingly being used for myopia management in children (Efron 2020). Centre‐distance soft multifocal lens designs incorporate a central zone that contains the distance refractive correction, with peripheral regions of the lens having relatively increased positive power (myopic defocus). This is achieved by either a gradual increase in power towards the periphery or using concentric peripheral zones of alternating myopic defocus and distance correction. Orthokeratology involves the use of specialised rigid contact lenses that are worn during sleep to change the topography of the cornea to reduce myopic refractive error and also manipulate peripheral retinal defocus. Safety remains a concern because of the greater risk of sight‐threatening microbial keratitis with overnight wear compared with daily contact lens wear modalities (Dart 2008). 

The most commonly used topical pharmacological intervention for myopia control is atropine, a non‐selective muscarinic antagonist, which has been widely used in clinical trials in concentrations ranging from 0.01% to 1.0%. Although higher atropine concentrations have been shown to be effective in retarding myopia progression in children, the higher incidence of side effects with higher doses, including cycloplegia (inhibition of accommodation) and pupil dilation (which causes blur for near vision and photophobia) limits its use. Furthermore, a rebound effect (involving more rapid myopia progression) after discontinuation of therapy is more pronounced with higher concentrations of atropine (Chia 2014). More recent studies have evaluated the efficacy of lower concentrations to reduce side effects and lessen the likelihood of rebound. The results of these studies have led to a renewed interest in the clinical application of low‐dose atropine (i.e. 0.01% to 0.05%) for myopia control (Wu 2019). Other pharmacological agents that have been evaluated for myopia control include topical tropicamide, cyclopentolate and pirenzipine (a selective M1 muscarinic antagonist) and the oral adenosine antagonist, 7‐methylxanthine.

Evidence that more time spent on near work activities is associated with higher odds of developing myopia (Huang 2015), and the observation that increased time spent outdoors is protective against myopia, after adjusting for near work, parental myopia and ethnicity (Rose 2008), have raised the possibility that environmental or behavioural interventions could be effective for myopia control. Trials of school‐based programmes that promote outdoor activities, conducted in East Asia, have reported a lower incidence of myopia onset but have limited impact on progression following onset of myopia (Dhakal 2022).

### How the intervention might work

Animal studies have shown that optically‐induced changes to the effective refractive status of the eye can regulate eye growth and influence refractive development (Troilo 2019). Specifically, the observation that imposed relative myopic defocus (image focused in front of the retina) can slow axial elongation has been the impetus for the development of novel multifocal spectacles and contact lenses that provide clear central vision, whilst at the same time presenting myopic defocus over a large proportion of the visual field. The critical area ratio required for these simultaneous competing defocus signals to dominate eye growth is currently unclear. However, the relative treatment effects reported for different optical treatment regimens suggest that there appears to be an eccentricity‐dependent decrease in the efficacy of myopic defocus beyond the near periphery (Smith 2014; Smith 2020).

Orthokeratology involves corneal reshaping lenses that are worn overnight to flatten the central cornea and reduce its dioptric power. The geometry of these lenses also creates a corneal profile that produces relative myopic defocus.

The precise mechanism by which anti‐muscarinic agents reduce myopic progression is not fully understood. A non‐accommodative mechanism is thought to be the most likely, and alternative targets have been proposed, including eye growth regulatory pathways that arise in the retina and are relayed to the sclera via the retinal pigment epithelium and choroid (McBrien 2013; Upadhyay 2020).

The protective effect of increased time outdoors on myopia development is thought to be related to the higher light intensity of sunlight and possibly its spectral composition (French 2013). Light levels have been shown to influence refractive development in animal models (Smith 2012). Higher light intensities stimulate retinal dopamine production, which is thought to inhibit axial elongation (Feldkaemper 2013).

### Why it is important to do this review

As a result of its increasing global prevalence and association with sight‐threatening pathologies, myopia is emerging as a major public health concern. Myopia is predicted to affect almost half of the world’s population by 2050, and the pathologic consequences of high myopia increase the risk of irreversible visual impairment and blindness. There has been considerable interest in the development of strategies to delay the onset of myopia and slow its progression. Myopia control interventions are increasingly being used in routine clinical practice (Efron 2020; Wolffsohn 2016). Evidence from randomised controlled trials (RCTs) indicates that the progression of myopia can be slowed by different interventions, although treatment efficacy is highly variable.

There is a broad consensus that the primary endpoints for judging efficacy in clinical trials of myopia control interventions should include change in axial length, in addition to change in refractive error (Brennan 2020; Walline 2018; Wolffsohn 2019). Myopia development and progression usually occur due to abnormal axial elongation. Therefore, axial length may be a better predictor of future progression and consequent risk of posterior pole complications (Brennan 2020). In terms of a minimal clinically important difference of the key efficacy outcomes in myopia control studies, an expert panel concluded that a mean difference between intervention groups of 0.25 D per year would be regarded as clinically significant (i.e. 0.75 D over the course of a three‐year study) (Walline 2018). This would correspond to a difference in axial length of approximately 0.3 mm.

An updated Cochrane systematic review, published in January 2020 (Walline 2020), evaluated the efficacy of a number of interventions, including spectacles, contact lenses and pharmaceutical agents, for slowing the progression of myopia in children. Walline 2020  concluded that topical anti‐muscarinic medication was effective in slowing myopia progression. Multifocal lenses, either spectacles or contact lenses, also conferred a small benefit. Although the update was published in 2020, the review only included evidence published up to the end of 2018. In this rapidly moving field, the results of additional important trials have subsequently been reported.

Eye care professionals often find it difficult to assimilate potentially conflicting evidence to inform their clinical decision‐making (Douglass 2020). It is therefore important that practitioners can access high‐quality and up‐to‐date evidence to inform practice. Moreover, parents of myopic children also need reliable information to help them to understand and interpret research findings. Given the large number of different interventions available for myopia control and the large number of completed and ongoing RCTs on this topic, there is an urgent need to evaluate the comparative effectiveness of different interventions. A network meta‐analysis (NMA) offers an advantage over a standard pairwise meta‐analysis in that it provides both direct comparisons of individual trials and indirect comparisons not directly evaluated in trials across a network of studies, thus generating the comparativeness of all interventions in a coherent manner. A NMA can also provide relative rankings of interventions to inform clinical decision‐making.

There are significant resource implications associated with myopia for both individuals and healthcare systems. This includes both corrected and uncorrected myopic refractive error. Lim 2009 estimated the mean direct costs of managing myopia in school‐aged children in Singapore. These costs included optometrist visits, spectacles, contact lenses and travel costs. The mean cost was estimated as USD 148 (median SGD 83.33) per year in 2006. In addition, Zheng 2013 estimated the lifetime costs for a person with myopia over an 80‐year lifespan to be USD 17,020 in 2011. There are also associated costs and quality‐of‐life impacts associated with uncorrected refractive error. Tahhan 2013 found a significant reduction in health state utility (a preference‐based quality‐of‐life measure) associated with uncorrected refractive error. Fricke 2012 estimated that the direct costs of correcting all cases of uncorrected refractive error globally would be approximately USD 28 billion (USD 28,000 million; price year not stated). Given these cost estimates, understanding the current evidence base for myopia control is key for both individuals and healthcare decision‐makers.

We plan to maintain this review as a living systematic review. This will involve searching the literature every six months and incorporating new evidence as it becomes available. This approach is appropriate for this review since it addresses an important clinical topic and there is currently significant uncertainty as to the most effective intervention. It is therefore important that consumers and healthcare providers have access to the most up‐to‐date evidence to make informed decisions. The review authors are aware of several relevant ongoing trials that will be important to incorporate in a timely manner.

## Objectives

To assess the comparative efficacy of optical, pharmacological and environmental interventions for slowing myopia progression in children using network meta‐analysis (NMA). To generate a relative ranking of myopia control interventions according to their efficacy. To produce a brief economic commentary, summarising the economic evaluations assessing interventions for myopia control in children. To maintain the currency of the evidence using a living systematic review approach.

## Methods

### Criteria for considering studies for this review

#### Types of studies

We included randomised controlled trials (RCTs) of optical, pharmacological and environmental interventions used alone or in combination for slowing the progression of myopia in children.

#### Types of participants

This review considered studies that included children 18 years old and younger. We excluded studies in which the majority of participants were older than 18 years at the start of the study. We also excluded studies that included participants with spherical equivalent myopia less than −0.50 D at baseline. The spherical equivalent is calculated by the sum of the spherical power plus half the cylindrical power of the refractive error.

We included studies that compared interventions of interest and reported having measured the relevant outcomes, irrespective of whether data for the outcomes were available.

#### Types of interventions

We included studies that compared any of the interventions listed below with a control group, or with each other. For the purposes of the analysis, we defined a control group as a placebo intervention or single vision spectacles or contact lenses.

- Undercorrection of myopia with single vision spectacle lenses
- Multifocal (bifocal or progressive addition) spectacle lenses, peripheral defocus spectacle lenses
- Multifocal soft contact lenses (MFSCL; concentric ring or progressive designs), rigid gas‐permeable contact lenses or corneal reshaping (orthokeratology) contact lenses
- Atropine (stratified according to dosing regime as high (≥ 0.5%), moderate (0.1% to < 0.5%) and low (< 0.1%)
- Other pharmaceutical agents (e.g. pirenzepine, 7‐methylxanthine)
- Environmental interventions (e.g. time spent outdoors, modifications to the performance of near work)

#### Types of outcome measures

##### Critical outcomes

###### Progression of myopia

Progression of myopia was assessed by:

- mean change in refractive error (spherical equivalent in D) from baseline for each year of follow‐up and measured by any method (e.g. objective or subjective refraction); and
- mean change in axial length for each year of follow‐up in millimetres (mm) from baseline for each year of follow‐up and measured by any method (e.g. ultrasound or optical biometry).

###### Change in refractive error and axial length following cessation of treatment ('rebound')

Rebound was evaluated when children in the treatment group were switched to the control treatment and then followed for a minimum period of one year. 

##### Important outcomes

###### Risk of adverse events

We described adverse events relating to the interventions as reported in the included studies, irrespective of severity. These included but were not limited to blurred vision, photophobia, hypersensitivity reactions, corneal infiltrative events and infections. In studies that graded clinical signs using standard anterior eye grading scales from normal to severe, we recorded the number of clinically significant signs (grade 3 or 4) that would usually require a clinical action.

Where data were available we documented withdrawals due to adverse events and number of 'serious' events.

###### Quality of life

We documented vision‐related or health‐related quality of life when reported, measured by any validated questionnaire (e.g. National Eye Institute (NEI) Visual Function Questionnaire 25 (NEI VFQ‐25), or EuroQol questionnaire, EQ‐5D).

###### Treatment adherence

Studies evaluated adherence with the prescribed treatment regimen using a variety of compliance measures, including daily wearing time with contact lenses and spectacle interventions as reported by parents or children, or both, or the proportion of participants in pharmacological studies following the required dosing regime.

###### Follow‐up

We have reported outcomes at one year, two years and as available for the duration of the study. We imposed no restrictions based on the length of follow‐up.

##### Brief economic commentary

We present evidence regarding relevant economic evaluations, as a brief economic commentary.

### Search methods for identification of studies

#### Electronic searches

The Cochrane Eyes and Vision Information Specialist searched the electronic databases below for RCTs and controlled clinical trials. There were no restrictions to language or date of publication. Given the similarity in the PICO and corresponding search strategies between the current review and a previous Cochrane Review on interventions for myopia control in children (Walline 2020), and the likelihood that studies included in Walline 2020 would meet the inclusion criteria for this review, we ran the search for the current review in parallel with the search strategy used by Walline 2020 up to the search date for the earlier review (26 February 2019) and removed duplicates. We combined the search results with all records identified up to 4 February 2022. 

We did not perform the generic search described in Electronic searches for adverse events, however we added a filter to the search strategy to identify systematic reviews of adverse events associated with myopia control interventions. We compared the findings of these reviews to the adverse events reported in the studies included in the current review.

In addition to these searches we carried out a MEDLINE and Embase search using economic search filters to specifically identify economic studies.

We have developed this review as a living systematic review, and we will re‐run the searches on a six‐monthly basis.

- Cochrane Central Register of Controlled Trials (CENTRAL; which contains the Cochrane Eyes and Vision Trials Register; 2022, Issue 2) in the Cochrane Library (Appendix 1)
- MEDLINE Ovid (1946 to 26 February 2022; Appendix 2)
- MEDLINE Ovid ‐ economic search (1946 to 26 February 2022; Appendix 3)
- MEDLINE Ovid ‐ adverse events (1946 to 26 February 2022; Appendix 4)
- Embase Ovid (1980 to 26 February 2022; Appendix 5)
- Embase Ovid ‐ economic search (1980 to 4 February 2022; Appendix 6)
- Embase Ovid ‐ adverse events (1980 to 26 February 2022; Appendix 7)
- ISRCTN registry (www.isrctn.com/editAdvancedSearch) (Appendix 8)
- US National Institutes of Health Ongoing Trials Register ClinicalTrials.gov (www.clinicaltrials.gov; Appendix 9)
- World Health Organization (WHO) International Clinical Trials Registry Platform (ICTRP; www.who.int/ictrp; Appendix 10)

#### Searching other resources

We searched the reference lists of identified study reports to identify additional studies. We also contacted the principal investigators of included studies for details of other potentially relevant studies not identified by the electronic searches, and of recently completed or ongoing studies.

### Data collection and analysis

#### Selection of studies

The Information Specialist at Cochrane Eyes and Vision downloaded all titles and abstracts retrieved from the electronic searches to EndNote (Endnote X9 2013) and removed duplicates before uploading to Covidence. Two review authors (from JGL, RS, BH, RD, PV) independently reviewed the titles and abstracts of the search results based on the eligibility criteria stated above. We categorised Abstracts for inclusion as 'Yes', 'Maybe' or 'No'. We obtained the full text of articles for the studies categorised as 'Maybe' and 'Yes', and reassessed them for final eligibility. After examining the full text, we labelled studies as 'include' or 'exclude'. Studies selected as 'exclude' by both authors were excluded from the review. We documented the reasons for exclusion. We resolved any screening discrepancies through discussion and, if necessary, through consultation with a third review author. One review author (AK) screened the economic search results.

##### Living systematic review considerations

We plan to screen any new citations retrieved by the six‐monthly searches immediately.

#### Data extraction and management

For eligible studies, two review authors independently extracted the data. We contacted the authors of the original reports to obtain further details if the data reported were unclear or incomplete. We exported the collected data into Review Manager Web (RevMan Web) (RevMan Web 2022). We extracted the following study characteristics.

- Methods: study design, number and location of study centre(s), date of study and total duration
- Participants: inclusion and exclusion criteria, number randomised, number lost to follow‐up or withdrawn, number analysed, mean age and standard deviation (SD), age range, gender
- Interventions: description of intervention and comparator
- Outcomes: primary and secondary outcomes specified and collected, and time points reported. Unit of analysis
- Notes: funding for study and conflicts of interest of study authors

#### Assessment of risk of bias in included studies

Pairs of review authors (from JGL, BH, RS, RD, PV, SM, DL) independently assessed the risk of bias in the included studies for all outcomes using the revised Cochrane risk of bias tool for randomised trials (RoB 2) 22 August 2019 version, described in Chapter 8 of the *Cochrane Handbook for Systematic Reviews of Interventions* (Higgins 2022a). RoB 2 covers five domains of bias:

- bias arising from the randomisation process;
- bias due to deviations from intended interventions;
- bias due to missing outcome data;
- bias in measurement of the outcome; and
- bias in selection of the reported result.

These domain‐level judgements provide the basis for an overall risk of bias judgement for the specific outcome being assessed. The response options for an overall risk of bias judgement in RoB 2 are the same as for individual domains (i.e. 'low risk of bias'; 'some concerns'; 'high risk of bias'). The following criteria were adopted:

- Low risk of bias: low risk of bias for all domains;
- Some concerns: 'some concerns' in at least one domain, but not at high risk of bias for any domain;
- High risk of bias: high risk of bias in at least one domain or the study is judged to have some concerns for multiple domains in a way that substantially lowers confidence in the result.

To implement RoB 2 assessments we used the Excel tool available at https://www.riskofbias.info/welcome/rob-2-0-tool/current-version-of-rob-2.

We did not include cluster‐randomised trials. In the case of cross‐over trials, we only used data from the first phase prior to the cross over and therefore used the version of the tool for parallel trials. Should cluster‐randomised and cross‐over trials be included in future updates of the review, we will use the versions of RoB 2 with additional considerations for these designs.

For all outcomes we assessed the effect of assignment to intervention (the intention‐to‐treat effect).

##### Assessment of bias in conducting the systematic review

We conducted the review according to this published protocol and have reported any deviations from it in the Differences between protocol and review section of the review.

#### Measures of treatment effect

We used mean differences (MDs) as the measure of treatment effect for the critical outcome 'progression of myopia', that is, difference in mean change in refractive error (SER) and axial length from baseline at each year of follow‐up.

#### Unit of analysis issues

When studies randomised only one eye per participant, the unit of analysis was the individual eye (participant). When studies randomised both eyes from the same participant (either to the same or different interventions), we analysed data adjusted for clustering or paired‐eye design. In the NMA, we accounted for the correlation between the effect sizes derived from the same study.

In multiple‐arm trials, to overcome a unit‐of‐analysis error for a study that could contribute multiple, correlated data, we combined groups to create a single pair‐wise comparison.

If we identify cluster‐RCTs in future updates, we will include them in meta‐analyses directly, where the sample size has been adjusted for clustering. We will combine them with the results from individual studies if there is little heterogeneity between the study designs and the interaction between the effect of the intervention and the unit of randomisation is considered to be unlikely. If studies present outcomes at individual level (i.e. a unit of analysis error), we will use established methods to adjust for clustering by calculating an effective sample size by dividing the original sample size by the design effect. This can be calculated from the average cluster size and the intra‐class correlation coefficient (ICC). Where the ICC is unknown, we will use an estimation from similar trials (Higgins 2022b).

#### Dealing with missing data

We contacted study authors to verify key study characteristics and to obtain missing outcome data. If we did not receive a response within eight weeks, we analysed the studies based on available data. We used the RevMan calculator to calculate missing standard deviations using other data from the study (e.g. confidence intervals) based on methods outlined in Chapter 10 of the *Cochrane Handbook for Systematic Reviews of Interventions* (Deeks 2022). 

#### Assessment of heterogeneity

We assessed clinical and methodological heterogeneity for each pairwise meta‐analysis by comparing the characteristics of included studies and by visual inspection of forest plots. We assessed statistical heterogeneity quantitatively for pairwise comparisons using the values of the Chi^2^ test and the I^2^ statistic (Higgins 2003). We interpreted I^2^ statistic values according to Chapter 10 of the *Cochrane Handbook for Systematic Reviews of Interventions* (Deeks 2022), as follows:

- 0% to 40% may not be important;
- 30% to 60% may represent moderate heterogeneity;
- 50% to 90% may represent substantial heterogeneity;
- 75% to 100% represents considerable heterogeneity.

For the NMA, we assumed a common estimate for the heterogeneity variance across the different comparisons. The assessment of statistical heterogeneity was based on the magnitude of the heterogeneity variance parameter (Tau^2^) estimated from the NMA models.

##### Assessment of statistical inconsistency

###### Local approaches for evaluating inconsistency

To evaluate the presence of inconsistency locally, we used the node splitting approach (Dias 2010), which assesses the agreement between direct and indirect evidence for each treatment comparison.

###### Global approaches for evaluating inconsistency

To check the assumption of consistency across the entire network, we used the 'design by treatment' interaction model (White 2015). This method accounts for different sources of inconsistency that can occur when studies with different designs are incorporated into the network (e.g. two‐arm trials versus multi‐arm trials), as well as inconsistency between direct and indirect evidence. 

#### Assessment of reporting biases

 If there are sufficient studies in future updates, we plan to run network meta‐regression models to detect associations between study size and effect size.

#### Data synthesis

We initially carried out standard pairwise meta‐analyses to combine outcome data using random‐effects models in RevMan Web. For comparisons with three or fewer trials, we used a fixed‐effect model. We combined change from baseline data in meta‐analyses with mean outcome data using the generic inverse variance (unstandardised) MD method, as outlined in Chapter 10 of the *Cochrane Handbook for Systematic Interventions* (Deeks 2022). In the case of substantial clinical, methodological or statistical heterogeneity, we generally did not attempt to combine data from individual trials but reported study results separately, however, subtotals were included in some analyses when presenting subgroups with varying degrees of heterogeneity.

For cross‐over trials we only extracted data from the first phase prior to cross over.

We conducted a NMA using the network suite of programs available in STATA (http://www.stata.com) for myopia progression, as defined by difference in change in SER and axial length at 12 and 24 months, using random‐effects multivariate models (Chaimani 2013; Chaimani 2015; White 2015). An important concept in NMA is 'transitivity', which implies that the distribution of effect modifiers is similar across all sources of direct evidence. The statistical manifestation of transitivity is consistency, which refers to the statistical agreement between the direct and indirect sources of evidence.  We checked for consistency in the network both locally (node‐splitting approach) and globally (design by treatment model).

We assumed a common heterogeneity across all comparisons in the network. We used te surface under the cumulative ranking curve (SUCRA) to rank the interventions for all available outcomes. SUCRA values range from 0% to 100%. The higher the SUCRA value (i.e. the closer to 100%), the greater the probability of an intervention ranking best. (Chaimani 2015; Salanti 2012).

In the primary NMA, we considered MFSCL, rigid gas‐permeable lenses and orthokeratology lenses as separate nodes. For spectacle lens interventions, there were separate nodes for undercorrected single vision spectacle lenses, multifocal spectacle lenses and peripheral plus spectacle lenses. We considered each pharmacological intervention as a separate node regardless of the dose. We did not anticipate a strong dose‐response effect except for atropine. We grouped atropine according to dosing regime as high (≥ 0.5%), moderate (0.1 % to < 0.5%) and low (< 0.1%).  We grouped all control arms (single vision spectacle lenses, single vision contact lenses, placebo eyedrops or no treatment) into a single node. 

When we were unable to perform a meta‐analysis, we undertook a narrative synthesis following guidance in Chapter 12 of the *Cochrane Handbook for Systematic Reviews of Interventions* (McKenzie 2022b). Specifically, we presented the effect estimates in structured tables and provided a descriptive summary of the range and distribution of the observed effects. In particular, we noted the direction of effects and whether these were consistent in the individual studies.

##### Brief economic commentary

Following the search outlined in the Search methods for identification of studies, we developed a brief economic commentary to summarise the availability and principal findings of the full economic evaluations assessing interventions for myopia control in children as outlined in Chapter 20 of the *Cochrane Handbook for Systematic Reviews of Interventions* (Aluko 2022). This brief economic commentary was planned to encompass full economic evaluations (i.e. cost‐effectiveness analyses, cost‐utility analyses and cost‐benefit analyses) conducted as part of a single empirical study, such as a RCT, a model based on a single such study or a model based on several such studies.

##### Living systematic review considerations

Whenever we identify new evidence in future updates (i.e. new studies, data, or other information) that is relevant to the review, we will extract the data and assess risk of bias, as appropriate. We will wait until the accumulating evidence changes one or more of the following components of the review before incorporating it and re‐publishing the review.

- The findings of one or more outcomes (e.g. clinically important change in size or direction of effect)
- Credibility (e.g. change in the overall confidence in the effect estimates for critical outcomes)

We will not use formal sequential meta‐analysis approaches for updated meta‐analyses.

##### Methods for future updates

We will review the scope and methods of this review annually in light of potential changes in the topic area or in evidence available for inclusion in the review. Each year, we will consider the necessity for the review to be a living systematic review by assessing ongoing relevance of the question to decision‐makers and by determining whether uncertainty is ongoing in the evidence and whether further relevant research is likely.

#### Subgroup analysis and investigation of heterogeneity

We performed predefined subgroup analyses for types of intervention modalities (i.e. spectacle and contact lens designs, and dose of particular pharmaceutical interventions (e.g. low‐, moderate‐ and high‐dose atropine)). There were insufficient data to carry out other proposed subgroup analyses.

#### Sensitivity analysis

We planned a sensitivity analysis on the exclusion of studies that we judged to be at high risk of bias or to raise some concerns in at least one domain of RoB 2. However, since we judged almost all the included studies at high risk of bias or with some concerns we did not seek to conduct a sensitivity analysis.

#### Summary of findings and assessment of the certainty of the evidence

 We planned to follow methods presented in Yepes‐Nunez 2019 to prepare summary of findings tables for the NMA, however because the network was not well‐connected, we primarily based our comparisons on direct evidence from classical pairwise meta‐analyses, except for moderate‐dose atropine.  We prepared summary of findings tables for progression of myopia at one and two years, with separate tables for change in spherical equivalent and change in axial length.

##### Evaluating confidence in the evidence

Instead of the planned CINeMA framework for evaluating confidence in the domains (Nikolakopoulou 2020; Salanti 2014) we summarised four levels of confidence for each relative treatment effect, corresponding to the usual GRADE approach: very low, low, moderate, or high (Schünemann 2022). In fact, because most evidence was direct versus control in NMAs, we used NMA estimates only when direct evidence was not available.

## Results

### Description of studies

We considered that all studies that met the inclusion criteria for Walline 2020 would potentially meet the inclusion criteria for the current review.

#### Results of the search

The searches performed by Walline 2020 to 26 February 2020 identified 41 studies with 74 ongoing studies and 25 studies awaiting classification. Updated electronic searches for the current review identified a further 1473 potentially eligible studies after removal of duplicates. We independently screened these studies for inclusion. We discarded 1290 citations and examined the full texts of the remaining 183 records. In total, we included 64 studies (reported in 225 records) and two studies published as conference abstracts are awaiting classification (for a full description see Characteristics of included studies and Characteristics of studies awaiting classification). 

The economic search was carried out on 4 February 2022 and yielded 80 studies that were screened by AK. No studies met the inclusion criteria. 

A search for systematic reviews of adverse events was carried out on 8 July 2022 and yielded 79 studies. These were screened, and we discuss relevant reviews in Agreements and disagreements with other studies or reviews.

For a summary of the screening process, see the study flow diagram (Figure 1; Liberati 2009).

**1:**
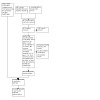


#### Study design

Sixty‐one studies used a parallel‐group design and three studies used a cross‐over design (Anstice 2011; Fujikado 2014; Hasebe 2008). The median sample size was 150 (range 24 to 660). Most participants were recruited from academic clinic settings, hospitals and in a few cases from private optometry or ophthalmology practices. The studies took place in China or other Asian countries (39 studies, 60.9%), North America (13 studies, 20.3%), Europe (7 studies, 10.9%), Australasia (2 studies, 3.1%), Israel (1 study, 1.6%) and Ghana (1 study, 1.6%); one multicentre study recruited participants in both Europe and Asia (1 study, 1.6%).

Fifty‐seven studies (89%) compared one or more myopia control interventions against a placebo intervention (generally single vision spectacles or contact lenses for optical interventions, and placebo or no treatment for pharmacological interventions). Four studies included a combined intervention group compared with control (Han 2019; MIT Study 2001; Schwartz 1981), and eight studies compared single or combined interventions with each other (ATOM 2 Study 2012; Cui 2021; Guo 2021; Kinoshita 2020; Shih 1999; Swarbrick 2015; Tan 2020; Zhao 2021).

Twenty‐two (34.4%) of the studies were of 12‐month duration, five studies(7.8%) had a duration of 18 to 20 months, 25 (39.1%) studies were 24 months, 11 (17.2%) up to 36 months and only one reported data over 36 months (Zhu 2021).

Seven studies were conducted before the year 2000 (Fulk 1996; Houston Study 1987; Jensen 1991; Pärssinen 1989; Schwartz 1981; Shih 1999; Yen 1989). Of the 49 studies that declared a source of funding, 19 (38.8%) were funded by the optical or pharmaceutical industry.

#### Characteristics of the participants

The review included 64 studies that randomised a total of 11,617 children, aged between 4 and 18 years, with a pooled mean age of 10.35 (range 7.6 to 14.0) years and 48% of participants were male. In the 58 studies that documented the level of myopia for inclusion, all but five studies recruited low to moderate myopes of −6.00 D or less; the other five studies included participants with higher levels of myopia up to −8.75 D (Charm 2013; Garcia‐del Valle 2021; Lyu 2020; Shih 1999; Zhu 2021). Most studies adopted an upper astigmatism limit of 1.00 D or 1.50 D. Three studies specifically recruited myopes with both myopia and near esophoria (Fulk 1996; Fulk 2002; STAMP Study 2012). One study selectively recruited participants with anisomyopia with an interocular difference of 1.00 D or greater (Zhang 2021). Eight studies restricted recruitment to those demonstrating a minimum myopic progression rate of at least 0.50 D in the year prior to enrolment (ATOM 2 Study 2012; Anstice 2011; Cheng 2010; CONTROL Study 2016; Lu 2015; Swarbrick 2015; LAMP Study 2019; Zhu 2021). Participants were sufficiently similar to satisfy the transitivity assumption for the NMA, that is, that there were no systematic differences between the available comparisons other than the treatments being compared.

#### Characteristics of the comparisons

##### Myopia control intervention versus control or placebo

###### Optical interventions

####### Spectacles

- **Undercorrection versus fully corrected single vision spectacle lenses (SVLs)** (3 studies; Adler 2006; Chung 2002; Koomson 2016). These studies, conducted in Israel, China and Ghana, compared the effect of under correcting myopia by either 0.50 D or 0.75 D versus fully corrected SVL. The follow‐up periods were 18 months for Adler 2006 and 24 months for  Chung 2002 and Koomson 2016.
- **Multifocal spectacle lenses (MFSLs) versus single vision spectacle lenses (SVLs)** (13 studies; Cheng 2010; COMET Study 2003: COMET2 Study 2011; Edwards 2002; Fulk 1996; Fulk 2002; Hasebe 2008; Houston Study 1987; Jensen 1991; MIT Study 2001; Pärssinen 1989; STAMP Study 2012; Yang 2009). These studies were conducted in North America (7 studies), Asia (4 studies) and Europe (2 studies). All studies enroled children aged 8 to 15 years. MFSLs were either bifocal (6 studies) or progressive addition lenses (7 studies) with near additions between +1.00 D and +2.00 D. The study durations were between 18 and 36 months. Eight studies had two arms and five studies had three arms. Hasebe 2008 compared bifocals with two add powers (+1.00 D and +2.00 D) to SVLs. Jensen 1991 randomised children to one of three groups, bifocals, SVLs or timolol maleate eye drops, and Pärssinen 1989 compared a group wearing bifocals (+1.75 D add) to a group wearing SVLs for distance vision only and a reference group wearing SVLs continuously. 
- **Peripheral plus spectacle lenses (PPSL) versus single vision spectacle lenses (SVLs)** (6 studies; Bao 2021; Hasebe 2014; Han 2018; Lam 2020: Lu 2015: Sankaridurg 2010). Novel spectacle lens designs have been developed that aim to reduce peripheral hyperopic defocus. These lenses, designated PPSLs, were compared to SVLs in Chinese and Japanese myopic children aged 6 to 16 years. Study durations were 1 to 2 years. Sankaridurg 2010 tested three lens designs (designated types I, II and III) that provided different relative peripheral power against SVLs in children aged 6 to 16 years. Hasebe 2014 compared two positively aspherised progressive addition lens designs, with +1.00 D or +2.00 D near add powers and a relative plus power in the upper portion of the lens, to SVLs. Lu 2015 randomised children to receive either PPSLs with up to a +2.50 D near addition or SVLs. Han 2018 conducted a three‐arm study in which children were randomised to PPSLs, SVLs, or orthokeratology lenses. Lam 2020 adapted a design that had previously been used in contact lenses (DISC Study 2011), to develop a spectacle lens with a clear central zone for distance correction and an annular peripheral zone consisting of a multiple array of segments approximately 1 mm in diameter, providing +3.50 D of myopic defocus. The lens, which is termed the ‘Defocus Incorporated Multiple Segments' (DIMS) lens, was tested in a two‐year study involving Chinese children aged 9 to 13 years, who were randomised to wear either DIMS lenses or SVLs. Finally, Bao 2021 tested a lens design based on the same principal that consisted of concentric rings of aspheric lenslets to provide myopic defocus. Children aged 8 to 13 years were randomised in a three‐arm study to receive either a lens with highly aspherical lenslets, a lens with slightly aspherical lenslets, or SVLs. The study reported interim results on myopia progression at one year.

####### Contact lenses

- **Multifocal soft contact lenses (MFSCL) versus single vision soft contact lenses (SVSCLs)** (9 studies; Anstice 2011; BLINK Study 2020; Chamberlain 2019; CONTROL Study 2016; DISC Study 2011; Fujikado 2014; Garcia‐del Valle 2021: Ruiz‐Pomeda 2018; Sankaridurg 2019). Nine studies investigated the efficacy of a variety of MFSCL designs compared to SVSCL. The MFSCLs incorporated a central zone to provide clear distance vision with relatively more positive peripheral lens power, which either increased gradually towards the periphery (progressive design) or presented as discrete peripheral annular zones (concentric ring design). Three studies followed participants for 12 months, four provided data to 20 to 24 months, and two had a duration of 36 months (BLINK Study 2020; Chamberlain 2019). Seven studies used a parallel‐group design, comparing MFSCLs with SVSCLs, and two studies used a cross‐over design (Anstice 2011; Fujikado 2014). Six studies adopted similar eligibility criteria and randomised children, aged 6 to 18 years with low to moderate myopia up to −6.00 D; Garcia‐del Valle 2021 included myopes to −8.75 D. Anstice 2011 and CONTROL Study 2016) only included children with documented myopia progression of −0.50 D or greater in the previous year, and the CONTROL Study 2016 additionally restricted inclusion to myopic children with near esophoria. Three studies used a similar centre distance dual focus concentric ring design with alternating distance correction zones and peripheral zones providing +2.00 D of defocus (Anstice 2011; Chamberlain 2019; Ruiz‐Pomeda 2018). These studies were conducted in New Zealand (Anstice 2011), Spain (Ruiz‐Pomeda 2018) and at sites in Europe, Asia and Canada (Chamberlain 2019). Garcia‐del Valle 2021 tested a MFSCL with a progressive design (+2.00 D addition) compared to SVSCL in Spanish schoolchildren age 7 to 15 years. Two studies, conducted in the USA (CONTROL Study 2016; BLINK Study 2020), used commercially available MFSCLs. The CONTROL Study 2016 evaluated children aged 8 to 18 years with progressive myopia, randomised to wear either a concentric bifocal soft contact lens or SVSCLs. The near add was selected based on the add power to neutralise the associated esophoria. The ‘Bifocal Lenses in Near‐sighted Kids' (BLINK) study (BLINK Study 2020), tested the efficacy of bifocal soft contact lenses with a central correcting zone for myopia and either a medium add (+1.50 D) or high add (+2.50 D) compared to SVSCLs. Three studies, conducted in China and Japan, used novel custom MFSCL designs (DISC Study 2011; Fujikado 2014; Sankaridurg 2019). The DISC Study 2011 tested the ‘Defocus Incorporated Soft Contact (DISC) lens’, a custom‐made bifocal soft contact lens of concentric ring design with a +2.50 D addition alternating with the normal distance correction. The DISC lens was compared to SVSCL in Chinese school children aged 8 to13 years, who were followed for two years. Fujikado 2014 used a cross‐over study design, in which Japanese children aged 6 to 16 years were randomised to wear a progressive MFSCL with a peripheral power of +0.50 D or SVSCLs in both eyes for 1 year and then were switched to the other type of lens for the second year. Sankaridurg 2019 randomised Chinese children aged 8 to 13 years to one of five groups: two groups wore MFSCLs that imposed peripheral myopic defocus of +1.50 D or +2.50 D with a stepped, relative positive power centrally of up to +1.00 D; and two groups wore extended depth of focus soft lens designs to optimise focus in front of and on the retina and degrade focus behind the retina. The control lens was a SVSCL.
- **Spherical aberration soft contact lenses versus single vision soft contact lenses (SVSCLs)** (1 study; Cheng 2016). This study randomised children aged 8 to 11 years to receive soft contact lenses with or without positive spherical aberration. Although the study was conducted in the USA, it enroled mostly Asian children (91%). The study was planned for two years, but was stopped early and reported only one‐year data.
- **Rigid gas‐permeable (RGP) contact lenses versus single vision soft contact lenses (SVSCLs) or single vision spectacle lenses (SVLs)** (2 studies; CLAMP Study 2004; Katz 2003). Two studies investigated the impact of RGP lenses on myopia progression compared to SVLs. Katz 2003 randomised Singaporean children aged 6 to 12 years to SVLs or RGP lenses. Myopia progression was evaluated at 1 and 2 years. The Contact Lens and Myopia Progression (CLAMP) Study (CLAMP Study 2004), was conducted in the USA and randomised children to RGP or soft single vision contact lenses. Annual myopia progression was reported based on change in SER and axial length, for the three‐year duration of the study.
- **Orthokeratology lenses versus single vision spectacle lenses (SVLs) or contact lenses** (9 studies; Bian 2020; Charm 2013; Han 2018; Jakobsen 2022; Lyu 2020; Ren 2017; ROMIO Study 2012; Tang 2021; Zhang 2021). Eight parallel‐group studies compared overnight orthokeratology contact lenses or SVLs, and in one study SVSCLs (Tang 2021). Participants were followed for 1 to 2 years. Seven studies enroled children with low to moderate degrees of myopia (up to −6.00 D), and two studies selectively recruited children with myopia 5.00 D or greater (Charm 2013; Lyu 2020). Zhang 2021 included participants with anisomyopia with a difference in myopia between eyes of 1.00 D or greater. Eight of the nine studies were conducted in China and one in Denmark (Jakobsen 2022). Axial length was the primary outcome in all studies. The 'Retardation of Myopia in Orthokeratology' (ROMIO) Study (ROMIO Study 2012), randomised 102 Chinese children aged 6 to 12 years to overnight orthokeratology lenses or SVLs, who were followed for two years. Charm 2013 randomised 52 highly myopic children (aged 8 to 11 years), with a SER of at least −5.75 D to partial reduction overnight orthokeratology lenses and daily SVLs for residual myopia, or a control group who were fully corrected with SVLs. Axial length was measured at six‐monthly intervals for two years. Lyu 2020 similarly investigated partial reduction orthokeratology lenses in participants with myopia up to −8.75 D. They randomised 102 children aged 8 to12 years into three groups: (1) orthokeratology lenses with a target reduction of 6.00 D; (2) orthokeratology lenses with a 4.00 D target reduction; or (3) SVLs. Axial length was measured at baseline and at 12 months. Jakobsen 2022 randomised 60 Danish children aged 6 to 12 years to orthokeratology lenses or SVLs, and followed them for 18 months. Four studies compared orthokeratology lenses to SVLs in Chinese children aged 8 to 15 years with myopia (Bian 2020; Han 2018; Ren 2017; Tang 2021). Ren 2017 also included a group that was treated with 0.01% atropine, and  Han 2018 included a group wearing PPSLs.

###### Pharmacological

####### Anti‐muscarinic agents

- **Atropine eye drops versus placebo or untreated control** (11 studies; ATOM Study 2006; Han 2019; Hieda 2021; LAMP Study 2019; Moriche‐Carretero 2021; Ren 2017; Wang 2017; Wei 2020; Yen 1989; Yi 2015; Zhu 2021). Twelve parallel‐group studies compared atropine to either placebo or an untreated control. These studies enroled children with low to high myopia (up to −8.00 D), aged from 4 to 15 years. Eligibility criteria in LAMP Study 2019 and Zhu 2021 additionally included a documented level of myopic progression in the past year. All studies were conducted in Asia, except for Moriche‐Carretero 2021, which was conducted in Spain. Participants were followed for periods ranging from one to four years. 
  - **High‐dose atropine (≥ 0.5%):** four studies compared 1% atropine to placebo or untreated control. The 'Atropine in the Treatment of Myopia' (ATOM) Study (ATOM Study 2006), was conducted in Singapore and involved 400 children aged 6 to 12 years, who were randomised to receive either 1% atropine or placebo to one eye and were followed for two years. Yi 2015 randomised 140 Chinese children, aged 7 to 12 years with low myopia (−0.50 to −2.00 D) to 1% atropine or placebo eyedrops nightly for 12 months. Zhu 2021 compared 1% atropine to placebo using a novel dosing regime in 660 Chinese children. The study was divided into three phases. In Phase 1, the treatment group received 1% atropine once per month for 24 months, this was reduced to once every two months for 12 months (Phase 2), followed by no drops for 12 months in Phase 3. The placebo group received the same dosing regime. Wang 2017 compared daily 0.5% atropine with placebo in 126 Chinese children with low myopia, who were followed for one year. Two, three‐armed studies included a 1% atropine arm. Han 2019 randomised 150 Chinese children aged 6 to 12 years in a 1:2:2 ratio to either an untreated control group, 1% atropine or a combination of 1% atropine with 0.5% raceanisodamine (a non‐selective muscarinic antagonist, used as an ingredient of traditional Chinese medicines). Yen 1989 included a group receiving 1% cyclopentolate.
  - **Low‐dose atropine (< 0.1%):** five studies tested lower doses of atropine, ranging from 0.01% to 0.05%. The 'Low‐concentration Atropine for Myopia Progression' (LAMP) Study (LAMP Study 2019), randomised 438 Chinese children aged 4 to 12 years with myopia of at least −1.00 D to four groups (in a 1:1:1:1 ratio): low‐concentration atropine eye drops at 0.05%, 0.025%, or 0.01% concentration or placebo. Participants were followed for one year. Four studies tested the efficacy of 0.01% atropine eyedrops versus placebo or an untreated control in participants aged between 5 and 15 years with low or moderate myopia, who were followed for one or two years (Hieda 2021; Moriche‐Carretero 2021; Ren 2017; Wei 2020). Participants in Hieda 2021 included 171 Japanese children, Wei 2020 included 220 Chinese children and Moriche‐Carretero 2021 randomised 339 Spanish children. In Ren 2017, 150 Chinese children were randomised (1:1:1 ratio) to 0.01% atropine, orthokeratology or single vision spectacle lenses. 
- **Pirenzepine eye drops versus placebo** (2 studies; PIR‐205 Study 2004;  Tan 2005). These two studies compared 2% pirenzepine gel, a selective M1 muscarinic receptor antagonist, to placebo. PIR‐205 Study 2004 was a two‐year, multicentre study conducted in the USA that randomised 174 myopic children aged 8 to 12 years in a 2:1 ratio to twice‐daily pirenzepine gel or placebo. Tan 2005 was conducted in centres in Singapore, Thailand and China, and randomised 353 children aged 6 to 13 years to one of three arms: (1) 2% pirenzepine gel twice daily; (2) placebo once daily and 2% pirenzepine gel once daily; or (3) placebo twice daily.

####### Anti‐muscarinic agent with co‐intervention

- **Tropicamide and multifocal spectacles (MFSLs)** (1 study; Schwartz 1981). This study was conducted in the USA and randomised 26 monozygous twin pairs aged 7 to 14 years to either a combination of MFSLs combined with 1% tropicamide or SVLs.
- **Atropine and multifocal spectacles (MFSLs) versus placebo** (2 studies; MIT Study 2001; Yen 1989). The 'Myopia Intervention Trial' (MIT) (MIT Study 2001), was conducted in Taiwan and evaluated SVLs, progressive addition lenses and progressive addition lenses combined with 0.5% atropine eyedrops. Yen 1989 randomly divided 247 Taiwanese children aged 6 to 14 years into three groups. Group 1 received 1% atropine and bifocal spectacles; group 2 received 1% cyclopentolate; and group 3 received saline eye drops. All groups were followed for 12 months.

####### Other pharmacological interventions

- **Timolol eyedrops versus single vision spectacle lenses (SVLs)** (1 study; Jensen 1991). One arm of Jensen 1991 investigated topical 0.25% timolol maleate, a non‐selective beta antagonist. Timolol eyedrops were given twice a day for two years and compared to MFSL or SVL control.
- **Systemic 7‐methylxanthine versus placebo** (1 study; Trier 2008). This study investigated the effectiveness of systemic 7‐methylxanthine, an adenosine receptor antagonist, in 83 Danish children aged 8 to 13 years. Participants were randomised to once daily 7‐methylxanthine or a placebo tablet.

##### Myopia control intervention versus myopia control interventions

- **Comparison of atropine doses** (2 studies; ATOM 2 Study 2012; Cui 2021). The ATOM 2 Study 2012 was conducted in Singapore and compared the efficacy and safety of three doses of topical atropine: 0.5%, 0.1%, and 0.01% in 400 children of Chinese ethnicity. Cui 2021 evaluated the safety and efficacy of 0.02% and 0.01% atropine in 400 myopic Chinese children, who were randomly allocated to atropine 0.02% (138 children) or 0.01% (142 children). The study also included a non‐randomised control group wearing single vision spectacle lenses (120 children). All participants were followed for two years.
- **Atropine and multifocal spectacle lenses (MFSLs) versus tropicamide** (1 study; Shih 1999). This study evaluated low‐concentration atropine in Taiwanese children aged 6 to 13 years. It randomly allocated 200 children to one of three atropine groups (0.5%, 0.25% or 0.1%) or 1% tropicamide as a control. The 0.5% atropine group were advised to wear MFSLs and the 0.25% atropine group were advised to wear slightly undercorrected SVLs.
- **Combined orthokeratology plus atropine versus orthokeratology alone** (3 studies; Kinoshita 2020*;* Tan 2020; Zhao 2021).  Kinoshita 2020* *randomly allocated 80 Japanese children with low to moderate myopia, aged 8 to 12 years, to receive either a combined orthokeratology and 0.01% atropine, or orthokeratology monotherapy. Tan 2020 randomised 72 Chinese children aged 6 to 11 years to receive combined orthokeratology/0.01% atropine compared to monotherapy. Similarly, Zhao 2021* * included as a separate parallel‐group comparison, combined 0.01% atropine/orthokeratology versus orthokeratology alone. Zhao 2021 randomised 40 children who had been wearing orthokeratology lenses for three months to orthokeratology and 0.01% atropine or orthokeratology only.
- **Comparison of orthokeratology designs** (1 study; Guo 2021). This study compared two designs of orthokeratology lenses with different back optic zone diameters. It randomly assigned 82 Chinese children aged 6 to 11 years to wear orthokeratology lenses with either a 6 mm or 5 mm back optic zone diameter and followed them for two years.
- **Orthokeratology versus rigid gas‐permeable contact lenses (RGP)** (1 study; Swarbrick 2015). This study conducted a randomised, contralateral‐eye cross‐over study over a one‐year period. Although the study was conducted in Australia, all 26 children were of East Asian ethnicity. Participants were fitted with an orthokeratology lens in one eye, chosen at random, and a conventional RGP lens worn daily in the contralateral eye. Children wore the lenses for six months. After a two‐week washout period, the lenses were reversed and lens wear was continued for a further six months.
- **Orthokeratology versus atropine** (2 studies; Ren 2017; Zhao 2021). Both studies compared 0.01% atropine to orthokeratology. 

##### Environmental interventions

We excluded studies that reported the impact of environmental interventions (e.g. elevated light levels in classrooms, increased outdoor time or regulated near working distances) mostly because the populations included participants both with and without myopia, or the primary outcome was incident myopia. One ongoing study, The 'Shanghai Time Outside to Reduce Myopia' (STORM) Study (NCT02980445), is a two‐year, school‐based, prospective, cluster‐randomised study that is investigating the effect of two 'doses' of increased outdoor time (40 and 80 minutes over normal time outdoors). Outcomes include the incidence of myopia in non‐myopic children, and the progression of myopia in myopic children.

#### Characteristics of the outcomes

All the included studies evaluated progression of myopia, either by measuring the mean change in refractive error, defined as spherical equivalent refraction (SER), mean change in axial length or both. Nine studies reported SER only (Adler 2006; Han 2018; Hasebe 2008; Jensen 1991, Pärssinen 1989; Schwartz 1981; Shih 1999; Yang 2009; Yen 1989), six studies investigating the efficacy of orthokeratology lenses reported axial length only (Bian 2020; Jakobsen 2022; Kinoshita 2020; ROMIO Study 2012; Swarbrick 2015; Zhang 2021), and the remaining 49 studies provided data on both SER and axial length.

Six studies investigated change in refractive error and axial length following cessation of treatment (commonly referred to as 'rebound'). In the STAMP Study 2012, children were randomly assigned to MFSL or SVLs for one year and all children wore SVLs in the second year. Cheng 2016 invited participants who had been randomised to soft contact lenses with positive spherical aberration or single vision soft lenses for one year to participate in a withdrawal phase where all children wore single vision contact lenses. To assess a potential rebound effect, Ruiz‐Pomeda 2018 invited children to participate in an additional year of follow‐up. Children were divided into three groups: a group in which children from the original study group continued wearing MFSCL; a group in which children discontinued MFSCL wear; and an SVL group, in which children from the original control group continued wearing SVLs. Three studies investigated the impact of terminating atropine treatment. In the ATOM Study 2006, children received 0.5%, 0.1% or 0.01% atropine for 12 months, after which treatment was terminated and the children were followed for a further 24 months. In the ATOM 2 Study 2012, children who received topical atropine 0.5%, 0.1% or 0.01% for 24 months entered Phase 2, the washout phase, where atropine was discontinued and SER and axial length assessed at 26, 32 and 36 months. In Zhu 2021, the frequency of 1% atropine eyedrop instillation was reduced from year 1 from once per month to once every two months in years 2 and 3, and withdrawn completely in year 4.

Twenty‐six studies provided data on safety outcomes in terms of the occurrence of adverse events. These included five studies reporting on adverse events with spectacle lens interventions, 11 reporting on contact lens interventions (including orthokeratology) and 10 studies reporting on various pharmacological interventions.

Only one study (LAMP Study 2019), which investigated the efficacy of low‐dose topical atropine, measured vision‐related quality of life. At the 12‐month follow‐up visit, the Chinese version of the 25‐Item National Eye Institute Visual Function Questionnaire was administered to all participants to determine the impact of different treatment groups on vision‐related quality of life.

Twenty‐one studies provided data on treatment adherence. Ten studies reported on compliance with spectacle lens wear including undercorrection with SVL, bifocal or progressive addition lenses. Compliance was typically based on parent or child, or both, self‐reporting wearing time (hours per day) and overall compliance, expressed as a % of participants in each arm. Similarly, six studies using contact lens interventions, reported on wearing time and percentage compliance between the intervention and control lenses. Four studies of pharmacological interventions provided data on self‐reported compliance with the study medication.

#### Ongoing studies

We identified 120 ongoing studies (see Characteristics of ongoing studies). These studies compare contact lenses or spectacle lenses (MFSCL, MFSL or orthokeratology) or pharmacological interventions to a control or other myopia control interventions. The majority of the ongoing studies investigate the efficacy of various doses of atropine used alone or in combination with other interventions.

#### Studies awaiting classification

We classified two studies published as conference abstracts as awaiting classification (see Characteristics of studies awaiting classification; Wang 2005; Viswanath 2022).

#### Excluded studies

We excluded a total of 137 studies. We excluded the Cambridge Anti‐Myopia Study 2013, which had been included in Walline 2020. The main reasons for exclusion were that the study was not randomised, population not eligible, intervention not eligible or ineligible outcome (see Characteristics of excluded studies for further details).

### Risk of bias in included studies

We assessed risk of bias using the RoB 2 (Higgins 2022a). A graphical representation of risk of bias for each comparison for the critical outcome 'progression of myopia' can be seen in Analysis 1.1; Analysis 1.2; Analysis 2.1; Analysis 2.2; Analysis 2.3; Analysis 3.1; Analysis 3.2; Analysis 4.1; Analysis 4.2; Analysis 5.1; Analysis 5.2; Analysis 6.1; Analysis 7.1; Analysis 7.3; Analysis 7.2; Analysis 7.4; Analysis 7.5; Analysis 8.1; Analysis 8.2; Analysis 9.1. 

The overall risk of bias across studies in each analysis reporting this outcome ranged from some concerns to high risk of bias depending on the particular intervention used.   

For the comparison 'undercorrection versus full correction spectacles', we assumed an overall high risk of bias, since two out of three studies had a high risk of bias in one domain, due to either an inappropriate method used to measure the outcome or no reasons given for missing data Table 188; Table 189.

Of the 10 studies that reported on progression of myopia in the analysis 'multifocal spectacle lenses versus single vision spectacles', we judged one study to be at high risk of bias (Cheng 2010). We judged the remainder as 'some concerns', usually due to insufficient information concerning allocation concealment and lack of an a priori statistical analysis plan. We therefore assumed an overall bias of 'some concerns' (see Table 190; Table 191). For 'peripheral plus spectacle lenses versus single vision spectacles', three of the six studies reporting this outcome had some concerns, with three studies judged to be at high risk of bias (Han 2018 Hasebe 2014; Lu 2015); see Table 193; Table 194).  

Seven of the eight studies reporting progression of myopia in the comparison 'multifocal soft contact lenses versus single‐vision soft contact lenses' were judged as 'some concerns', primarily due to failure to describe the method of allocation concealment and no information on the predetermined analysis plan. We gave an overall judgement of 'some concerns' for this outcome (see Table 195; Table 196).

Only two studies evaluated progression of myopia following RGP wear. We judged one study at high risk (Katz 2003). We therefore gave an overall judgement of 'high risk' for this outcome (see Table 199; Table 200).

We judged four of the seven studies reporting change in axial length from baseline after wearing orthokeratology lenses as 'some concerns' (Analysis 1.2). We judged three studies at high risk of bias (Lyu 2020; Ren 2017; ROMIO Study 2012). We gave an overall judgement of 'some concerns' for this outcome (see Table 201).

For progression of myopia in the comparison 'anti‐muscarinics versus control', we assumed an overall risk of bias of 'some concerns' for studies using different doses of atropine, as we judged the majority of the studies reporting the outcome as 'some concerns'. However, we judged both of the studies reporting on 2% pirenzepine to be at high risk of bias (PIR‐205 Study 2004; Tan 2005), which gave an overall judgement of 'high risk' for this outcome (see Table 202; Table 204; Table 203; Table 205).

For the outcome 'change in refractive error and axial length following cessation of treatment', three of the four included studies had some concerns and one was at high risk of bias (Zhu 2021; see Analysis 2.3; Analysis 4.3; Analysis 4.4; Analysis 7.5; Analysis 7.6).

Detailed risk of bias assessments are available at: osf.io/ms83h/

### Effects of interventions

See: Table 1; Table 2; Table 3; Table 4

See summary of findings tables for overall treatment effects for any myopia control intervention on progression of myopia compared to placebo. The certainty of the evidence is also provided and if appropriate, the reasons for downgrading (Table 1; Table 2; Table 3; Table 4).

We performed standard pairwise and network meta‐analyses for optical and pharmacological interventions compared to a control group (consisting of either standard SVLs or contact lenses or a placebo) for the critical outcome 'progression of myopia'. We also compared myopia control interventions to each other. In total, we included 52 studies, analysing 8152 participants, in either the standard or network meta‐analysis. 

Twelve studies did not contribute directly to the quantitative synthesis (Cheng 2016; Fulk 1996; Houston Study 1987; Han 2018; Han 2019; Hasebe 2008; Lu 2015; Schwartz 1981; Shih 1999; Swarbrick 2015; Wang 2017; Yen 1989). For these studies, we reported study‐specific results, as appropriate.

Quantitative synthesis was not possible for the outcomes 'risk of adverse events', 'quality of life', or 'treatment adherence' due to insufficient available data. For these outcomes we provided a descriptive summary of the range and distribution of the observed effects, and summarised the study‐level findings in structured tables.

#### Critical outcomes

##### Progression of myopia

###### Myopia control intervention versus control

####### Pairwise meta‐analysis results

Direct treatment estimates from pairwise meta‐analysis for optical and pharmacological interventions versus control/placebo are reported in Analysis 1.1; Analysis 1.2; Analysis 2.1; Analysis 2.2; Analysis 3.1; Analysis 3.2; Analysis 4.1; Analysis 4.2, Analysis 5.1 Analysis 6.1; Analysis 7.1; Analysis 7.3; Analysis 7.2; Analysis 7.4; Analysis 8.1; Analysis 8.2). We assessed progression of myopia as mean change in refractive error (defined as SER) from baseline for each year of follow‐up or change in axial length from baseline, or both. To facilitate interpretation of the forest plots, negative mean differences (MDs) for changes in refractive error represent faster progression of myopia in the intervention group compared to progression in the control group. Thus, point estimates to the left of the null on the forest plots favour the control group. For the measurement of changes in axial length, negative MDs for changes in axial length represent faster axial elongation in the control group compared to the intervention group, and therefore estimates to the left of the null favour the intervention group.

- **Optical interventions: spectacles**
  - Spectacle interventions designed to reduce accommodative demand and lag during near work by undercorrection, or the use of MFSLs have been common in practice for many years. Three studies, involving 292 participants, compared spectacles that were undercorrected by −0.50 D to −0.75 D to fully corrected SVLs (Analysis 1.1; Analysis 1.2). There was no evidence that undercorrection slowed myopic progression, based either on change in refractive error (MD at 1 year −0.15 D (95% CI −0.29 to 0.00); MD at 2 years 0.02 D (95% CI −0.05 to 0.09)) or change in axial length (MD at 1 year 0.05 mm (95% CI −0.01 to 0.11); MD at 2 years −0.01 mm (95% CI −0.06 to 0.03)).
  - Thirteen studies compared bifocal or progressive addition lenses to SVLs. We included 10 studies with 1612 participants in a quantitative synthesis (Analysis 2.1; Analysis 2.2). Eight studies provided data up to two years and four studies followed participants for up to three years (Cheng 2010; COMET Study 2003; COMET2 Study 2011; Pärssinen 1989). There was a small reduction in myopia progression at both one‐ and two‐year follow‐up (change in refractive error at 1 year, MD 0.14 D, 95% CI 0.08 to 0.21; and at 2 years, MD 0.19 D, 95% CI 0.08 to 0.30). The three‐year results showed considerable heterogeneity (I^2^ = 86%), however after removing two studies judged to be at high risk of bias (Cheng 2010; Pärssinen 1989), heterogeneity was substantially reduced (I^2^ = 0%). The pooled three‐year MD after removing these studies was 0.21 D (95% CI 0.08 to 0.34). The three studies not included in the meta‐analysis reported inconsistent results (Fulk 1996; Hasebe 2008; Houston Study 1987). Hasebe 2008 reported significantly less myopia progression in children wearing progressive addition lenses over the first 18 months of a cross‐over study compared to children wearing SVLs, but no difference in the second 18‐month period. In another 18‐month study (Fulk 1996), children wearing bifocals progressed at a rate of −0.39 D per year compared to −0.57 D per year in the SVL group, however these differences were not significant (P = 0.26). The Houston Study 1987 found no significant difference between groups wearing bifocals (+1.00 D and +2.00 D add) and SVLs. We included four studies with 896 participants that reported change in axial length with progressive addition lenses in a quantitative analysis (Analysis 2.2). There was a small reduction in axial elongation in progressive addition lens wearers for each year of follow‐up (1‐year MD −0.06 mm, 95% CI −0.09 to −0.04; 2‐year MD −0.07 mm, 95% CI −0.12 to −0.03; 3‐year MD −0.12 mm, 95% CI −0.18 to −0.07).
  - The rationale for prescribing peripheral plus spectacle lenses (PPSL) is to reduce hyperopic defocus in the peripheral retina. Six studies compared PPSL to SVLs (Bao 2021; Han 2018; Hasebe 2014; Lam 2020; Lu 2015; Sankaridurg 2010). Changes in refractive error from baseline showed considerable heterogeneity (I^2^ = 89% to 91%; Analysis 3.1). Mean differences at one year ranged from 0.02 D to 0.97 D. Only two studies followed children for two years (Hasebe 2014; Lam 2020). These studies showed contrasting results. Hasebe 2014 found no difference in myopia progression with positively aspherised progressive addition lenses (MD 0.12 D, 95% CI −0.06 to 0.31). In contrast, Lam 2020 found a significant reduction in progression using the Defocus Incorporated Multiple Segments (DIMS) spectacle lens (MD 0.55 D, 95% CI 0.38 to 0.72). Four studies provided data on changes in axial length from baseline, showing similarly high heterogeneity (Analysis 3.2). The combination of all three novel lenses tested by Sankaridurg 2010 were not significantly different from SVLs at one year (MD −0.02 mm, 95% CI −0.08 to 0.04), contrasting with designs used by Lam 2020 and Bao 2021, which showed less axial elongation. Only two studies provided two‐year data for axial length. Hasebe 2014 reported no significant difference (MD −0.07mm, 95% CI −0.20 to 0.07), whereas Lam 2020 showed that the reduced axial elongation demonstrated at one year, continued into the second year (−0.32 mm, 95% CI −0.39 to −0.25).
- **Optical interventions: contact lenses**
  - Studies tested a variety of contact lens design, including multifocal soft contact lenses (MFSCL), positive spherical aberration contact lenses, rigid gas‐permeable (RGP) and orthokeratology lenses. Conceptually, MFSCL use a progressive or concentric ring design to create myopic defocus and reduce myopia progression. Nine studies, compared MFSCL to single vision soft contact lenses (SVSCL). We excluded Fujikado 2014 from the quantitative analysis since it used a lens design that was distinct from the other lenses in the comparison. Consequently, we included eight studies with a total of 1135 participants in a quantitative synthesis (Analysis 4.1; Analysis 4.2). Five studies provided data on change in refractive error and axial length up to two years and two studies followed children for up to three years (BLINK Study 2020; Chamberlain 2019). Over the three‐year reporting period, there was a progressive reduction in myopia progression with MFSCL compared to SVCL, although there was a suggestion that the change in refractive error reduced over time (1‐year MD 0.26 D, 95% CI 0.17 to 0.35; 2‐year MD 0.30 D, 95% CI 0.19 to 0.41; 3‐year MD 0.47 D, 95% CI 0.13 to 0.82). A significant reduction in axial elongation was seen across all three years (1‐year MD −0.11 mm, 95% CI −0.13 to −0.09; 2‐year MD −0.15 mm, 95% CI −0.19 to −0.12; 3‐year MD −0.22 mm, 95% CI −0.34 to −0.10).
  - Cheng 2016 investigated the effect of soft contact lenses with positive spherical aberration. The mean reported change in refractive error in this study was 0.137 D (95% CI −0.007 to 0.281) amongst 52 children in the spherical aberration group compared with 57 children in the single vision contact lens group at one‐year follow‐up. In terms of axial elongation, children in the positive spherical aberration group showed 0.143 mm (95% CI −0.188 to −0.098) less elongation compared with the control at one year.
  - Two studies investigated the use of rigid gas‐permeable contact lenses (RGPs) in slowing the progression of myopia compared to single vision contact lenses in one study (CLAMP Study 2004), and SVLs in the other (Katz 2003) (Analysis 5.1; Analysis 5.2). The CLAMP Study 2004 followed up participants for three years and Katz 2003 followed up participants for two years. We did not pool data on change in refractive error due to considerable heterogeneity (I^2^ > 90%). The two studies reported contrasting results, with the CLAMP Study 2004 showing significantly less myopia progression over three years with the RGPs compared to participants wearing single vision contact lenses (1‐year MD 0.40 D, 95% CI 0.19 to 0.61; 2‐year MD 0.54 D, 95% CI 0.27 to 0.81; 3‐year MD 0.63 D, 95% CI 0.30 to 0.96). By contrast, Katz 2003 observed no difference in myopia progression over two years (1‐year MD −0.02, 95% CI −0.14 to 0.10; 2‐year MD −0.05 D, 95% CI −0.25 to 0.15). Neither study was able to show any effect on axial elongation over three years (pooled estimate 1‐year MD 0.02 mm, 95% CI −0.05 to 0.10; 2‐year MD 0.03 mm, 95% CI −0.05 to 0.12; 3‐year MD 0.05 mm, 95% CI −0.12 to 0.22).
  - Eight studies, involving 787 participants, compared orthokeratology lenses to SVLs or SVSCLs and provided data up to two years (Analysis 6.1). Overnight wear of orthokeratology lenses flattens the central cornea and temporarily reduces refractive error. Since it is not possible to assess the true progression of refractive error without ceasing lens wear for a period of time to allow the cornea to return to its pre‐treatment state, the included studies presented change in axial length as the primary efficacy outcome. A significant reduction in axial elongation was seen across both years (1‐year MD −0.19 mm, 95% CI −0.23 to −0.15; 2‐year MD −0.28 mm, 95% CI −0.38 to −0.19).
- **Pharmacological interventions: antimuscarinics**
  - **Atropine:** 11 studies compared topical atropine to control (placebo, no treatment or SVLs). These were grouped according to dosing regime into high dose (≥ 0.1%) or low dose (< 0.1%). The majority of studies testing atropine reported data at one year, with only four studies (ATOM Study 2006; Hieda 2021; Moriche‐Carretero 2021; Zhu 2021) reporting at two years.  
  - Several studies tested atropine as a co‐intervention or in head‐to‐head dose comparisons and these are described below.
    - **High‐dose atropine:** three studies (1072 participants) compared high‐dose atropine (1%) to control (ATOM Study 2006; Yi 2015; Zhu 2021). At one year, effect sizes for change in refractive error ranged from MD 0.79 D to 1.1 7 D in favour of high‐dose atropine Analysis 7.1. A reduction in axial elongation was also seen with 1% atropine at one year (MD −0.31 to −0.35 mm; Analysis 7.2). Studies reporting at two years similarly showed that high‐dose atropine had greater efficacy. The ATOM Study 2006 showed a change in refractive error of MD 0.92 D (95% CI 0.75 to 1.09); and change in axial length of MD −0.40 mm (95% CI −0.48 to −0.32). Zhu 2021 showed a change in refractive error of MD 1.41 D (95% CI 1.30 to 1.52) and change in axial length of MD −0.54 mm (95% CI −0.57 to −0.51) (Analysis 7.3; Analysis 7.4). Two studies, not included in the meta‐analysis, also investigated 1% atropine versus placebo and reported significantly less myopia progression in children in the atropine group at the end of the follow‐up period, however the data were not presented in a form that could be included in the meta‐analysis (Han 2019; Wang 2017). We also excluded Yen 1989 and MIT Study 2001 since the atropine groups also wore MFSL.
    - **Low‐dose atropine:** five studies (1143 participants) compared lower atropine doses (0.01% to 0.05%) to control (Hieda 2021; LAMP Study 2019; Moriche‐Carretero 2021; Ren 2017; Wei 2020). Results for these comparisons for each year of follow‐up showed considerable heterogeneity (I^2^ > 90%), however all effects were in the same direction, and we included subgroup summary effect estimates in the forest plots as the best estimate of the intervention effect. At one year, effect sizes for change in refractive error were in the range 0.08 D to 0.80 D, and change in axial length ranged from −0.04 mm to −0.35 mm in favour of low‐dose atropine (Analysis 7.1; Analysis 7.2). Studies reporting at two years similarly showed a greater efficacy for low‐dose atropine (Hieda 2021 change in refractive error MD 0.22 D, 95% CI 0.09 to 0.35; change in axial length −0.14 mm, 95% CI −0.20 to −0.08; Moriche‐Carretero 2021, change in refractive error MD 0.25 D, 95% CI 0.17 to 0.33; change in axial length −0.17 mm, 95% CI −0.22 to −0.12) in favour of atropine (Analysis 7.3; Analysis 7.4).
  - **Pirenzepine:** two studies investigated 2% pirenzepine eyedrops (PIR‐205 Study 2004; Tan 2005; see Analysis 7.1; Analysis 7.2 Analysis 7.3). At one‐year follow‐up, average myopia progression was less for participants treated with pirenzepine compared to placebo (PIR‐205 Study 2004 MD 0.27 D, 95% CI 0.11 to 0.43; Tan 2005 MD 0.47 D, 95% CI 0.16 to 0.78). The difference in progression between groups continued at two years (PIR‐205 Study 2004 MD 0.41 D, 95% CI 0.13 to 0.69). Data for axial length were only available at one year. Tan 2005 found a slowing of axial elongation (MD −0.13 mm, 95% CI −0.14 to −0.12), whereas the PIR‐205 Study 2004 found no significant difference in axial length (MD −0.04 mm, 95% CI −0.15 to 0.07).
- **Other pharmacological interventions**
  - One arm of Jensen 1991 investigated the non‐selective beta‐antagonist timolol maleate compared to a SVL control. The differences in myopia progression were not significant at one year (MD −0.05 D, 95% CI −0.21 to 0.11) or at two years (MD −0.04 D, 95% CI −0.30 to 0.22). This study did not measure axial length.
  - Trier 2008 compared systemic 7‐methylxanthine, an oral adenosine receptor antagonist, versus a placebo tablet for one year. At one‐year follow‐up, the differences in myopia progression were not significant (change in refractive error MD 0.07 D, 95% CI −0.09 to 0.24; change in axial length MD −0.03 mm, 95% CI −0.10 to 0.03; Analysis 8.1; Analysis 8.2).

####### Network meta‐analysis results

We conducted NMAs for change in SER and axial length at 12 and 24 months. See Figure 2 for the network maps for each comparison, and Table 5 presenting the number of study arms (participants) for each intervention. Direct comparisons between interventions were limited to different doses of atropine, meaning that only indirect comparisons were possible. League‐tables presenting all indirect and mixed comparisons for SER and axial length at 12 and 24 months can be seen at https://osf.io/ms83h/. Figure 3 presents forest plots of NMA comparisons with control.

**2:**
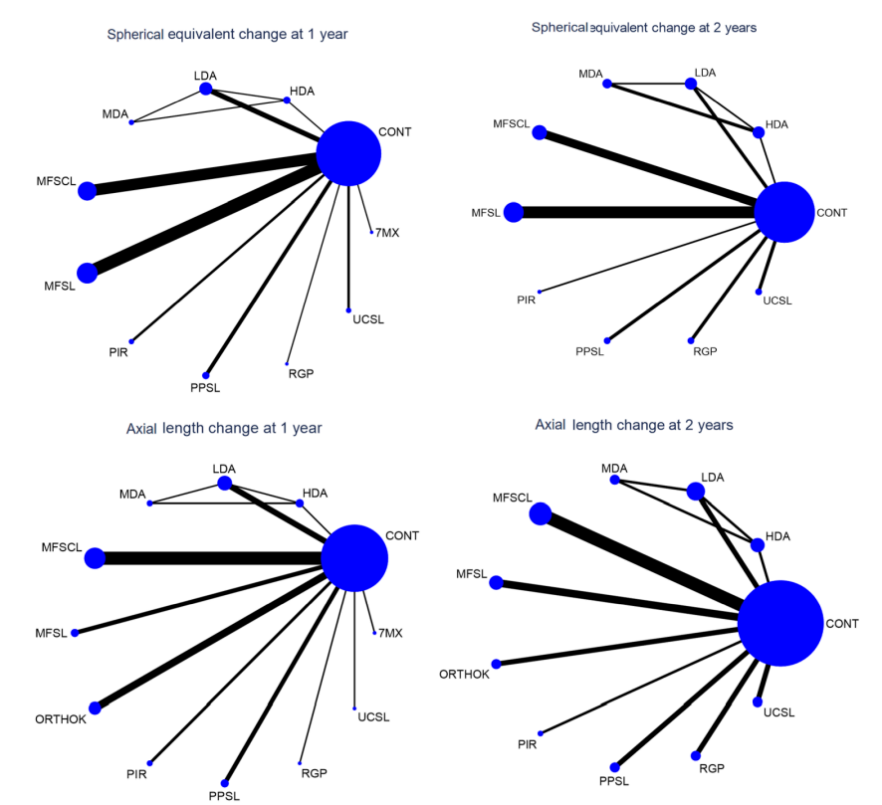
Network maps for change in spherical equivalent and change in axial length at 1 and 2 years **7MX**: 7‐methylxanthine; **HDA**: high‐dose atropine, **LDA:** low‐dose atropine; **MDA**: moderate‐dose atropine; **MFSCL**: multifocal soft contact lenses; **MFSL**: multifocal spectacle lenses; **ORTHOK**: orthokeratology; **PIR**: pirenzipine; **PPSL**: peripheral plus spectacle lenses ; **RGP**: rigid gas‐permeable contact lenses; **UCSVL**: undercorrected single vision spectacles

**1:** Number of trial arms and participants for each intervention and outcome in all NMAs

	**Outcome**			
	**Spherical equivalent at 1 year ** Number of treatment arms (participants)	**Spherical equivalent at 2 years ** Number of treatment arms (participants)	**Axial length at 1 year ** Number of treatment arms (participants)	**Axial length at 2 years ** Number of treatment arms (participants)
**Treatment arm**				
**Control**	35 (2459)	22 (1899)	33 (2319)	20 (1730)
**High‐dose atropine**	3 (411)	3 (346)	3 (411)	2 (305)
**Moderate‐dose atropine**	1 (155)	2 (237)	1 (155)	1 (141)
**Low‐dose atropine**	5 (581)	3 (324)	5 (581)	3 (324)
**Pirenzipine**	2 (210)	1 (53)	2 (210)	1 (53)
**7‐methylxanthine**	1 (35)	‐	1 (35)	‐
**Orthokeratology**	‐	‐	5 (234)	2 (49)
**Multiifocal soft contact lenses**	9 (723)	5 (540)	9 (723)	5 (540)
**Peripheral plus spectacle lenses**	‐	2 (188)	3 (340)	2 (188)
**Rigid gas‐permeable contact lenses**	2 (176)	2 (154)	2 (176)	2 (154)
**Multifocal spectacle lenses**	4 (445)	7 (622)	4 (445)	3 (404)
**Undercorrected single vision spectacles**	1 (47)	2 (122)	1 (47)	2 (122)

**3:**
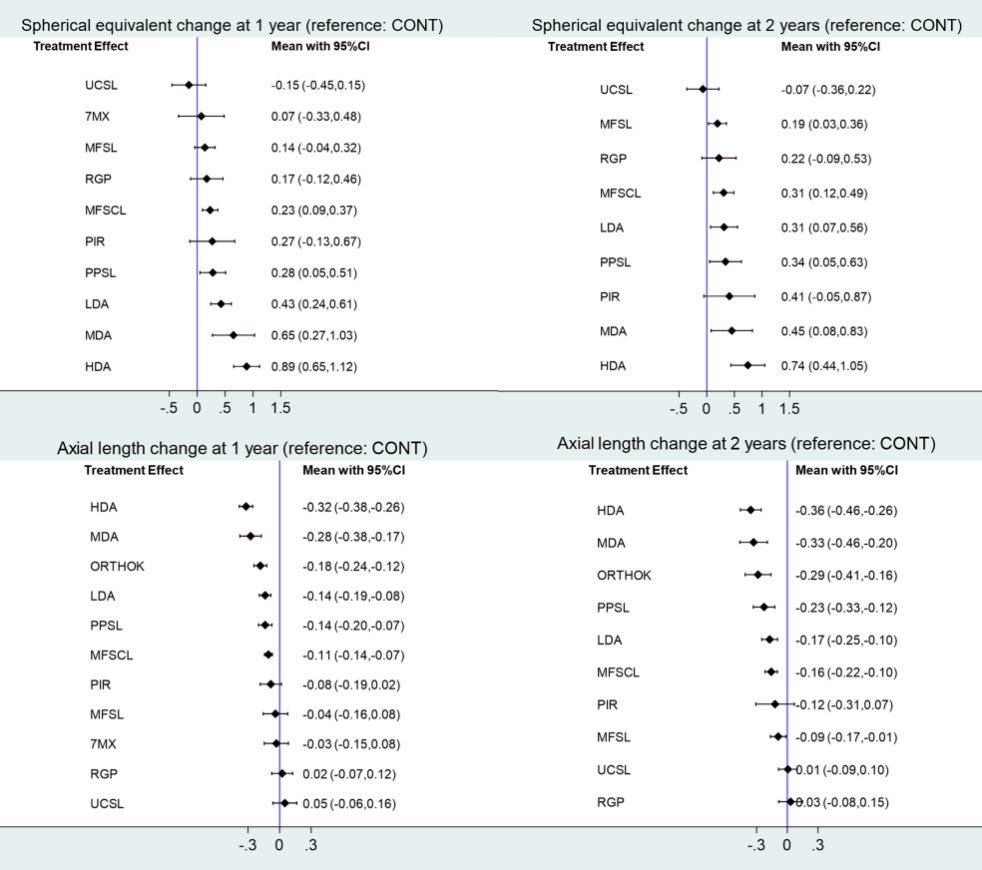
Estimates of effect from network meta‐analyses for all treatments versus control for progression of myopia (based on spherical equivalent and axial length) at 1 and 2 years. Comparisons with control are less precise than direct meta‐analyses due to the lack of directly comparative evidence. **7MX**: 7‐methylxanthine; **HDA**: high‐dose atropine; **LDA:** low‐dose atropine; **MDA**: moderate‐dose atropine; **MFSCL**: multifocal soft contact lenses; **MFSL**: multifocal spectacle lenses ; **ORTHOK**: orthokeratology; **PIR**: pirenzipine;**PPSL**: peripheral plus spectacle lenses; **RGP**: rigid gas‐permeable contact lenses; **UCSVL**: undercorrected single vision spectacles

- **Change in SER at 1‐year**
  - The NMA included 30 studies (4694 participants) with two connected closed loops comparing different doses of atropine or control. There was no overall inconsistency (p=0.185). The only two closed loops were partly overlapping and showed no inconsistency.
  - The overall NMA between‐study SD was large (0.19 D), which made most NMA estimates versus control as, or more, imprecise than the corresponding direct evidence. For this reason, Table 1 presents direct and indirect evidence for all comparisons versus control, including the certainty of evidence assessment, except for moderate‐dose atropine versus control, for which only indirect evidence was available.
- **Change in axial length at 1‐year**
  - The NMA included 31 studies (4864 participants) with two connected closed loops comparing different doses of atropine or control. There was no overall inconsistency  (P = 0.236). The only two closed loops were partly overlapping and showed no inconsistency.
  - The overall NMA between‐study SD was large (0.048 mm), which made most NMA estimates versus control as, or more, imprecise than the corresponding direct evidence. Table 3 presents direct and indirect evidence for all comparisons versus control, except for moderate‐dose atropine versus control, for which only indirect evidence was available.
- **Change in SER and change in axial length at 2‐years**
  - The NMAs of the change in SER and change in axial length at two years included 24 studies (4485 participants) and 21 studies (4010 participants), respectively. Table 2 and Table 4 present the evidence from direct and indirect comparisons, except for moderate‐dose atropine versus control, for which only indirect evidence was available.
- **SUCRAs and mixed comparisons in NMAs**
  - All indirect comparisons are presented for illustrative purposes as league‐tables at https://osf.io/ms83h/, where differences not including nil are marked in grey. 

Table 6 presents all SUCRA values for NMAs of change in SER and axial length at one and two years, where the three highest SUCRAs are highlighted in bold print. As can be seen, high‐dose atropine and moderate‐dose atropine were amongst the three best SUCRAs for all outcomes. Low‐dose atropine and PPSLs were third for SER at one and two years respectively, while orthokeratology was amongst the best three for axial length at one and two years.

**2:** SUCRAs in all NMAs

**Intervention**	**SER ** **1 year**	**SER ** **2 years**	** AL ** **1 year**	**AL ** **2 years**
High‐dose atropine	**98.9**	**97.9**	**98.1**	**94.2**
Moderate‐dose atropine	**87.8**	**72.3**	**92.2**	**88.1**
Low‐dose atropine	**74.5**	55.9	64.9	54.9
Peripheral plus spectacle lenses	57.2	**70.6**	65.7	68.4
Pirenzepine	54.2	65.8	45.3	43.6
Multifocal soft contact lenses	50.6	56.5	52.8	51
Rigid gas‐permeable contact lenses	40.2	41.1	12.9	7.9
Multifocal spectacles	36.0	35.6	32.2	34.4
7‐methylxanthine	30.4	‐	29	‐
Control	14.9	9.2	19.5	13.6
Undercorrected single vision spectacles	5.3	6.5	8.5	12.8
Orthokeratology	‐	‐	**79**	**81.1**
The three highest ranking interventions for each outcome are highlighted in bold **NMA:** network meta‐analysis; **SUCRA:** surface under the cumulative ranking curve				

###### Antimuscarinics combined with multifocal spectacle co‐intervention versus single vision spectacles

Three studies compared antimuscarinics combined with MFSLs to SVLs (Schwartz 1981; MIT Study 2001; Yen 1989). Schwartz 1981 randomised monozygous twin pairs to receive either bifocal spectacles combined with 1% tropicamide or SVLs. The paper did not present any numerical results, but the study authors stated that control twins showed more progression of myopia than their co‐twins who received tropicamide and bifocals, but the difference was not statistically significant.

The Myopia Intervention Trial (MIT) (MIT Study 2001), evaluated progressive addition spectacle lenses combined with 0.5% atropine compared to placebo eyedrops plus SVLs. At the end of the 18‐month follow‐up period, participants in the atropine plus progressive addition spectacle lenses group showed significantly less myopia progression (MD 0.98 D, 95% CI 0.76 to 1.20) and significantly less axial elongation (MD −0.47 mm, 95% CI −0.47 to −0.27) than participants in the placebo plus SVL group.

Yen 1989 randomly divided children into three groups. Group 1 received 1% atropine and bifocal spectacles; group 2 received 1% cyclopentolate, and group 3 received placebo eyedrops plus SVLs. At one year there was less myopia progression in the atropine plus bifocal group compared to the control group (MD 0.70 D, 95% CI 0.43 to 0.97).

###### Myopia control intervention versus myopia control interventions

Three studies compared different doses of topical atropine to each other ( ATOM 2 Study 2012;  Cui 2021; Shih 1999). The ATOM 2 Study 2012 compared the efficacy of 0.5%, 0.1%, and 0.01% atropine in children of Chinese ethnicity. The mean change in refractive error at two years was −0.30 D (95% CI −0.40 to −0.20); −0.38 D (95% CI −0.48 to 0.29) and −0.49 D (95% CI −0.64 to −0.35) in the atropine 0.5%, 0.1%, and 0.01% groups, respectively. Pairwise differences were statistically significant for the comparison between 0.01% and 0.5% atropine (P = 0.02). Changes in axial length were 0.27 mm (95% CI 0.21 to 0.33); 0.28 mm (95% CI 0.24 to 0.33) and 0.41 mm (95% CI 0.36 to 0.46) for the high, moderate and low doses.

Cui 2021 evaluated the efficacy of 0.02% and 0.01% atropine in Chinese children. The mean changes in refractive error at two years were −0.80 D (95% CI −0.90 to −0.70) for the 0.02% concentration and −0.93 D (95% CI −1.04 to −0.82) for the 0.01% concentration. The corresponding changes in axial length were 0.62 mm (95% CI 0.56 to 0.68) and 0.72 mm (95% CI 0.66 to 0.78). 

Shih 1999 compared three doses of atropine (0.1%, 0.25% and 0.5%) versus 1% tropicamide control. Participants in the 0.5% group wore bifocal spectacles, those in the 0.25% group were provided with slightly undercorrected SVLs and the 0.1% group wore fully corrected SVLs. Myopia progression at the end of the two‐year follow‐up period was significantly slowed for each atropine group compared with tropicamide, with the 0.5% atropine dose showing the least progression compared with the tropicamide group, MD 1.95 D (95% CI 1.60 to 2.30) for 0.1% atropine; MD 1.98 D (95% CI 1.68 to 2.28) for 0.25% atropine; and MD 2.42 D (95% CI 2.16 to 2.68) for 0.5% atropine.

Three studies evaluated the combination of low‐dose atropine (0.01%) combined with orthokeratology, compared to orthokeratology alone (Kinoshita 2020; Tan 2020; Zhao 2021). These studies were conducted in Japan and China and followed participants for up to two years. The primary outcome was change in axial length (Analysis 9.1). We found a reduction in axial elongation for the combination therapy group compared to monotherapy at each year of follow‐up (1‐year MD −0.13 mm, 95% CI −0.16 to −0.09; 2‐year MD −0.11 mm, 95% CI −0.21 to −0.01).

Two studies included treatment arms that compared low‐dose atropine (0.01%) to orthokeratology at one year. There was no significant difference in axial length between treatments (Ren 2017 MD 0.03 mm, 95% CI −0.17 to 0.03; Zhao 2021 MD 0.05 mm, 95% CI−0.02 to  0.12).

Guo 2021 compared two designs of orthokeratology lenses with 6 mm or 5 mm back optic zone diameters. The rationale was that a smaller back optic zone diameter would increase myopia control efficacy by inducing a steeper distribution of the relative corneal refractive power profile within the pupillary diameter and further increase higher order aberrations. Axial elongation was lower in the 5 mm group (MD 0.04 mm, 95% CI −0.005 to 0.08) than the 6 mm group (MD 0.17 mm, 95% CI 0.13 to 0.21).

Swarbrick 2015 conducted a randomised, contralateral‐eye cross‐over study comparing a regular RGP lens in one eye and an orthokeratology lens in the other, conducted over a one‐year period. Lenses were worn for six months and then crossed over after a two‐week washout period for a further six months. This study did not report data eligible for analysis.

##### Change in refractive error and axial length following cessation of treatment

Six studies investigated changes in refractive error and axial length following cessation of the myopia control intervention (ATOM Study 2006; ATOM 2 Study 2012; Cheng 2016; Ruiz‐Pomeda 2018; STAMP Study 2012; Zhu 2021). These studies compared the rate of myopia progression in the intervention group after switching to the control intervention to progression in the original control group.

In the STAMP Study 2012, children wore MFSL or SVLs for one year and all children wore SVLs in the second year. At the end of year 2 there was no difference in myopia progression between groups (MD 0.00 D, 95% CI −0.17 to 0.17; Analysis 2.3). This study did not measure progression of axial length.

In Ruiz‐Pomeda 2018, children who had worn MFSCL or SVSCL for two years were invited to participate in an additional one‐year follow‐up study to investigate rebound. One group discontinued MFSCL wear and switched to SVLs and progression was then compared to the original control group, who continued wearing SVLs. After one year there was no significant difference in progression of refractive error (MD 0.09 D, 95% CI −0.16 to 0.34) or axial length (MD 0.01, 95% CI −0.05 to 0.07; Analysis 4.4). 

Cheng 2016 invited participants who had worn either novel soft contact lenses with positive spherical aberration or conventional single vision soft lenses to participate in a 12‐month withdrawal study, where all children wore single vision lenses. The study authors reported that they found no evidence of a rebound effect at one year.

Three studies investigated the impact of terminating atropine treatment. The ATOM Study 2006 discontinued atropine treatment after children had received 1% atropine for two years. Children were followed for a further year. At the end of this period, the study compared myopia progression to the placebo‐treated group. Progression of refractive error in the original 1% atropine‐treated group was significantly greater than the control group at the end of the second year (MD −0.76 D, 95% CI −0.90 to −0.62; Analysis 7.5). The study authors reported axial length data as change from baseline over the entire three‐year duration of the study and therefore these data were not suitable for evaluating rebound.

Zhu 2021 used a novel dosing regime. Participants received 1% atropine eye drops once per month for 24 months, then every other month for 12 months followed by no drops for 12 months. Progression at the end of the one‐year withdrawal period was evaluated and compared to the placebo group. One year after terminating treatment the atropine group still showed a slowing in the progression of refractive error (MD 0.34 D, 95% CI 0.26 to 0.42; Analysis 7.5) and a reduction of axial elongation (MD −0.21 mm, 95% CI −0.23 to −0.19; Analysis 7.6) compared to the placebo group.  

The ATOM 2 Study 2012 was a dose comparison study, with participants randomised to receive 0.5%, 0.1% or 0.01% atropine for 24 months followed by a 12‐month withdrawal phase. During the washout period, myopic progression was greater in participants treated with 0.5% atropine (MD −0.87 D, 95% CI −0.96 to −0.78) compared to 0.1% (MD −0.68 D, 95% CI −0.76 to −0.61) and 0.01% atropine (MD −0.28 D, 95% CI −0.36 to −0.20; P < 0.001). Axial elongation was also greater in the 0.5% group (MD 0.35 mm, 95% CI 0.32 to 0.38) compared to the 0.1% (MD 0.33 mm, 95% CI 0.30 to 0.36) and 0.01% (MD 0.19 mm, 95% CI 0.16 to 0.22) groups. 

#### Important outcomes

##### Risk of adverse events

The risk of adverse events was generally poorly reported. We extracted data on the frequency of adverse events from 26 studies (see Table 7; Table 8; Table 9), comprising five studies reporting on the effects of spectacle interventions (Adler 2006; Bao 2021; COMET2 Study 2011; Hasebe 2008; Sankaridurg 2010), 11 on contact lens interventions, including orthokeratology (BLINK Study 2020; Chamberlain 2019; Cheng 2016; CLAMP Study 2004; Garcia‐del Valle 2021; Guo 2021; Jakobsen 2022; Kinoshita 2020; Lyu 2020; Ruiz‐Pomeda 2018; Tan 2020) and 10 using pharmacological interventions (ATOM 2 Study 2012; Cui 2021; Hieda 2021; LAMP Study 2019; PIR‐205 Study 2004; Shih 1999; Tan 2005; Wei 2020; Yen 1989; Zhu 2021). Multiple adverse events occurring in the same study participant, including events affecting both eyes, were counted as independent events.

**3:** Risk of adverse events: spectacle lens interventions

**Study**	**Arm (participants)**	**Total number of events ** ** (participants)**	**Dizziness**	**Blurred vision**	**Distortion**	**Headache**	**Difficulty with stairs**	**Other**
Adler 2006	UC (25)	2 (2)	‐	2	‐	‐	‐	‐
	FC (23)	0 (0)	‐	‐	‐	‐	‐	‐
Bao 2021	PPSL (115)	0 (0)	‐	‐	‐	‐	‐	‐
	SVL (55)	0 (0)	‐	‐	‐	‐	‐	‐
COMET2 Study 2011	MFSL (59)	3 (3)	1	1	‐	‐	‐	1
	SVL (59)	14 (14)	9	3	‐	‐	‐	2
Hasebe 2008^a^	MFSL (87)	37 (37)	10	19	‐	0	8	‐
	SVL (91)	24 (24)	6	14	‐	0	4	‐
Sankaridurg 2010	MFSL (160)	13 (13)	2	9	1	1	‐	‐
	SVL (74)	3 (3)	0	1	2	0	‐	‐
**FC:** full correction; **MFSL:** multifocal spectacle lenses; **PPSL:** peripheral plus lenses;**UC:** undercorrection; **SVL:** single vision spectacle lenses								

^a^Results of 6/12 questionnaire survey (reported in Suemaru 2008 (secondary reference to to Hasebe 2008 ).

**4:** Risk of adverse events: contact lens interventions

**Study**	**Arm (number of participants)**	**Total number of events (participants)**	**Grade ≥ 3 slit‐lamp** **findings**	**Corneal infiltrates**	**Allergy/** **hypersensitivity** **reactions**	**Corneal erosions/staining**	**Corneal neovascularisation**	**Papillary reaction**	**Other**
**Multifocal soft contact lenses**									
BLINK Study 2020^a^	Total lens wearers (294 )	35 (35)	NR	10	7	4	‐	9	5
	MFSCL (196) SVSCL (98)	‐	‐	‐	‐	‐	‐	‐	‐
Chamberlain 2019	MFSC (70)	8 (6)	1	4	‐	‐	‐	1	2
	SVSCL (74)	7 (5)	0	3	‐	‐	‐	‐	4
Cheng 2016	PSASL (64)	2 (1)	0	‐	2	‐	0	‐	0
	SVSCL (63)	3 (2)	0	‐	2	‐	1	‐	0
Garcia‐del Valle 2021	MFSCL (32)	10 (8)	NR	‐	‐	2	3	4	1
	SVSCL (26)	4 (4)	NR	‐	‐	1	1	2	0
Ruiz‐Pomeda 2018^b^	MFSCL (41)	11 (NR)	0	‐	‐	5	2	4	‐
	SVL (33 )	3 (NR)	0	‐	‐	1	0	2	‐
**Rigid gas‐permeable lenses**									
CLAMP Study 2004	RGP (59)	0 (0)	NR	‐	0	‐	‐	‐	‐
	SVSCL (57)	4 (4)	NR	‐	1	‐	‐	‐	3
**Orthokeratology**									
Guo 2021	Ortho‐K 6 mm (32)	26 (NR)	0	2	‐	11	‐	‐	13
	Ortho‐K 5 mm (26)	16 (NR)	0	3	‐	6	‐	‐	7
Jakobsen 2022	Ortho‐K (19)	2 (2)	2	‐	‐	2	‐	‐	‐
	SVL (28)	0 (0)	0	‐	‐	0	‐	‐	‐
Kinoshita 2020	Ortho‐K + 0.01% atropine (38)	3 (3)	NR	0	‐	2	‐	‐	‐
	Ortho‐K monotherapy (35)	1 (1)	NR	1	‐	1	‐	‐	‐
Lyu 2020	Ortho‐K (68)	16 (16)	2	‐	‐	14	‐	‐	‐
	SVL (34)	3 (3)	0	‐	‐	3	‐	‐	‐
Tan 2020	Ortho‐K + 0.01% atropine (35)	1 (1)	0	0	‐	‐	‐	‐	1
	Ortho‐K (30)	2 (2)	0	1	‐	‐	‐	‐	1
**MFSCL:** multifocal soft contact lenses; **NR**: not reported; **Ortho‐K:** orthokeratology; **PSASCL:** positive spherical abberation soft contact lenses; **SVL:** single vision spectacle lenses; **SVSCL:** single vision soft contact lenses									

^a^Data combined for intervention and control lenses ^b^Data at final 24‐month visit

**5:** Risk of adverse events: antimuscarinics

**Study**	**Arm (number of participants)**	**Total number of events (participants)**	**Photophobia/glare**	**Blurred vision**	**Hypersensitivity** **reactions**	**Ocular irritation**	**Systemic complications**	**Other**
**Higher‐dose atropine**								
ATOM 2 Study 2012	Atropine 0.5% (161)	43 (23)	1	13	10	‐	‐	15
	Atropine 0.1% (155)	47 (41)	NR	20	7	‐	‐	14
	Atropine 0.01% (84)	15 (14)	NR	11	0	‐	‐	3
Shih 1999	Atropine 0.5% (41)	10 (10)	9	0	1	‐	0	‐
	Atropine 0.25%, (47)	3 (3)	3	0	0	‐	0	‐
	Atropine 0.1% (49)	0 (0)	0	0	0	‐	9	‐
Yen 1989	Atropine 1% (32)	32 (32)	32	0	0	0	0	‐
	Cyclopentolate 1% ((32)	0 (0)	0	0	0	0	0	‐
	Placebo (32)	0 (0)	0	0	0	0	0	‐
Zhu 2021	Atropine 1% (330)	352 (330)	205	65	3	61	‐	18
	Placebo (308)	NR	NR	NR	NR	NR	‐	NR
**Lower‐dose atropine**								
Cui 2021	Atropine 0.02% (138)	32 (32)	32	‐	0	0	‐	‐
	Atropine 0.01% (142)	33 (33)	33	‐	0	0	‐	‐
	SVL (120 )	3 (3)	3	‐	0	0	‐	‐
Hieda 2021	Atropine 0.01% ((85)	2 (2)	1	0	‐	‐	‐	1
	Placebo (86)	1 (1)	0	1	‐	‐	‐	0
LAMP Study 2019	Atropine 0.05% (93)	17 (17)	8	‐	9	‐	‐	‐
	Atropine 0.025% (86)	14 (14))	4	‐	10	‐	‐	‐
	Atropine 0.01% (91)	17 (17)	6	‐	11	‐	‐	‐
Wei 2020	Atropine 0.01% (110)	8 (8)	5	0	3	‐	‐	‐
	Placebo (110)	2 (2)	1	0	1	‐	‐	‐
**Pirenzepine**								
PIR‐205 Study 2004	Pirenzepine 2% (117)	163 (NR)	7	91	47	18	‐	Several 'other' AEs documented. No significant difference between test vs placebo
	Placebo (57)	29 (NR)	1	13	10	5	‐	
Tan 2005	Pirenzepine 2% (142)	178 (NR)	‐	95	83	‐	‐	Several 'other' AEs documented. No significant difference between test vs placebo
	Placebo (171)	16 (NR)	‐	6	10	‐	‐	
**AEs:** adverse events;**NR**: not reported; **SVL:** single vision spectacles								

Studies used a variety of methods to record adverse events. Symptoms were usually elicited using questionnaires, telephone interviews or were self‐reported at follow‐up appointments by parents or children, or both. Objective clinical signs were usually based on clinical examination at each follow‐up visit, however in some studies, participants were advised to return to the clinic for unscheduled visits should adverse events arise.

###### Spectacle interventions

Data on adverse events were available from 446 participants wearing spectacle lens interventions (undercorrection, MFSLs and PPSLs) and 302 SVL‐wearing controls. Study duration was one to three years. MFSLs and PPLSs were usually well tolerated following a short adaptation period and the reported adverse events were generally mild. There were 55 events in the active arm and 41 in the control arm. Dizziness and blurred vision were the most commonly reported adverse events with similar rates in SVL controls (dizziness: active arm 13/446, controls 15/302; blurred vision: active arm 31/446, controls 18/302) see Table 7. Overall there were three withdrawals due to adverse events in studies using MFSLs. 

###### Contact lens interventions

Eleven studies provided safety data on 1068 participants receiving contact lens interventions, which included various soft contact lens designs (including MFSCLs and SVSCLs), RGP and orthokeratology. Study duration was one to three years. The control arm in orthokeratology studies was typically SVLs. Two studies compared orthokeratology monotherapy to combined orthokeratology and low‐dose atropine. Safety outcomes were monitored by clinical examination of the anterior segment of the eye using the slit‐lamp biomicroscope at follow‐up appointments. Many studies graded clinical signs using standard grading scales, which used either artist‐rendered or photographic images to grade corneal and conjunctival signs on a 0 to 4 scale from normal to severe. Grade 3 and 4 are regarded as clinically significant and usually require a clinical action. For the most part, studies using MFSCLs and SVSCLSs reported adverse events separately for each arm, however the largest study (BLINK Study 2020), reported safety data for all arms combined (294 children). The most commonly reported adverse events in studies involving soft contact lenses were corneal infiltrative events (17/664 wearers), conjunctival papillary reaction (20/664), and corneal staining (12/664), see Table 8. The number of events were similar for test and control lenses. These events were generally not serious, with only one grade 3 event, and one participant in the BLINK Study 2020 was reported as a 'probable microbial keratitis'. There were four reported adverse effect‐related withdrawals in these studies (incidence: approx 0.6%).

Adverse events in orthokeratology studies were more common: corneal infiltrates (7/254 wearers), corneal staining (36/254), with four cases of corneal staining graded 3 or higher. There were 12 withdrawals due to adverse events from these studies (incidence approx. 5%).

###### Pharmacological interventions

####### Atropine

Safety data were available for eight studies using various doses of atropine. The three most common adverse events were photophobia or glare, blurred vision (particularly for near vision) and hypersensitivity reactions see Table 9. Atropine studies used high (≥ 0.5%; 564 children), moderate (0.1% to < 0.5%; 251 children), and low (< 0.1%; 829 children) atropine doses, with study durations ranging from 12 to 48 months. Adverse events were generally dose dependent with a greater likelihood of adverse events with higher atropine doses (high‐dose 437 events in 564 children; moderate‐dose 150 events in 251 children; low‐dose 138 events in 829 children). There were higher numbers of withdrawals due to adverse events in studies using high‐dose atropine (7% over 1 year in ATOM Study 2006 and 21% over 2 years in Zhu 2021) compared to 2% or fewer in studies using lower atropine doses (Hieda 2021; Moriche‐Carretero 2021: Wei 2020).

Evaluating the rates of photophobia and difficulties with near vision was confounded by the use of photochromic spectacle lenses or sunglasses to mitigate photophobia, and multifocal lenses for near vision problems in some studies, which may have reduced reporting of symptoms. 

####### Pirenzepine

PIR‐205 Study 2004 and Tan 2005 (259 children in the active treatment arms) documented ocular and systemic adverse events. The three systemic adverse events most frequently reported were headache, common cold, and flu syndrome in the PIR‐205 Study 2004, and increased cough, respiratory infection, and rhinitis in Tan 2005. The three ocular adverse events most frequently reported by both studies were symptoms of decreased accommodation, papillae/follicles, and medication residue on the eyelids or eyelashes. Forty‐three children in the active treatment arms withdrew due to adverse events.

##### Quality of life

One study (LAMP Study 2019) reported on vision‐related quality of life using the Chinese version of the 25‐item National Eye Institute Visual Function Questionnaire (NEI VFQ‐25). This validated quality‐of‐life instrument assesses 11 subscales: general health, general vision, ocular pain, near vision, distance vision, social function, mental health, role limitations, dependency, colour vision and peripheral vision. LAMP Study 2019 evaluated quality of life in 438 participants receiving one of three doses of atropine (0.05%, 0.02% or 0.01%) or placebo. The study authors reported no difference between groups in vision‐related quality of life, with similar scores across all 11 domains.

##### Treatment adherence

Quantitative data on adherence to myopia control treatment were available in 21 studies, comprising 10 studies investigating spectacle interventions (Bao 2021; COMET Study 2003; COMET2 Study 2011; Fulk 2002; Hasebe 2008; Koomson 2016; Lam 2020; Pärssinen 1989; STAMP Study 2012; Yang 2009), six studies evaluating contact lens interventions (Anstice 2011; BLINK Study 2020; Chamberlain 2019; DISC Study 2011; Fujikado 2014; Katz 2003), and five using pharmacological interventions (ATOM 2 Study 2012; Hieda 2021; LAMP Study 2019; PIR‐205 Study 2004; Trier 2008). Where adherence data were available at multiple time points we report the results at the longest time point (see Table 10; Table 11; Table 12).

**6:** Adherence: spectacle interventions

**Study**	**Arm** **(number of participants)**	**Wearing time** **hours per day** **Mean (SD)**	**% compliant ** **(always or most of the time)**	**P value**
Bao 2021	PPSL HAL (54)	13.4 (2.1)	‐	P = 0.35
	PPSL SAL (53)	13.4 (1.8)	‐	
	SVL (50)	13.1 (1.7)	‐	
COMET Study 2003	MFSL (235)	‐	93%	NR
	SVL (234)	‐	96%	
COMET2 Study 2011^a^	MFSL (58)	‐	72%	NR
	SVL (58)	‐	90%	
Fulk 2002	MFSL (42)	‐	90%	NR
	SVL (40)	‐	96%	
Hasebe 2008	MFSL (87)	‐	96%	Reported as ‘not significant’
	SVL (91)	‐	94%	
Koomson 2016	UC (75)	‐	97%	NR
	FC (75)	‐	96%	
Lam 2020	PPSL (79)	15.5 (2.6)	‐	Reported as ‘not significantly different’
	SVL (81)	15.3 (2.1)	‐	
Pärssinen 1989	MFSL (79)	‐	77%	NR
	SVL (79)	‐	82%	
STAMP Study 2012	MFSL (40	‐	93%^a^	NR
	SVL (43)	‐	91%^a^	
Yang 2009	MFSL (89)	‐	87% (combined)	NR
	SVL (89)	‐		
**FC:** fully corrected single vision spectacles; **HAL:** highly aspheric; **MFSL:** multifocal spectacle lenses; **NR:** not reported; **PPSL:** peripheral plus spectacle lenses; **SD:** standard deviation; **SVL:** single vision spectacle lenses;**PPSL:** peripheral plus lenses; **SAL:** slightly aspheric; **UC:** undercorrected single vision spectacles				

^a^Compliance during school hours.

**7:** Adherence: contact lens interventions

**Study**	**Arm (number of participants)**	**Wearing time** **hours per day** **Mean (SD)**	**% compliant ** **(always or most of the time)**	**P value**
Anstice 2011	MFSCL (20)	13.2 (2.8)	100%	P = 0.41
	SVSCL (20)			
BLINK Study 2020	MFSCL (196)	11.0 (4.4)^a^	‐	NR
	SVSCL (98)			
Chamberlain 2019	MFSCL (70)	13.7 (1.5)	‐	Reported P > 0.05
	SVSCL (74)	13.3 (1.5)		
DISC Study 2011	MFSCL (111)	6.5 (2.2)	‐	P = 0.644
	SVCL (110)	6.3 (1.7)		
Fujikado 2014	MFSCL (11)	13.2(1.0)	‐	P = 1.00
	SVCL (13)	13.2(1.1)		
Katz 2003**	RGP (75)	‐	31.5%	NR
	SVL (75)		98.4%	
**MFSCL:** multifocal soft contact lenses; **NR:** not reported; **RGP**: rigid gas‐permeable lenses; **SD:** standard deviation; **SVSCL:** single vision soft contact lenses; **SVL:** single vision spectacle lenses				

^a^Both arms combined.

**8:** Adherence: pharmacological interventions

**Study**	**Arm (number of participants)**	**Compliance with medication**	**P value**
ATOM 2 Study 2012	Atropine 0.5% (161)	98.7%	NR
	Atropine 0.25% (155)	96.8%	
	Atropine 0.1% (84)	98.8%	
Hieda 2021	Atropine 0.01% (85)	83.3%	NR
	Placebo (86)	85.7%	
LAMP Study 2019	Atropine 0.05% (109)	93.6%	NR
	Atropine 0.025% (108)	95.4%	
	Atropine 0.01% (110)	90.9%	
	Placebo (111)	90.1%	
PIR‐205 Study 2004	Pirenzepine 2% (117)	79%	NR
	Placebo (57)	79%	
Trier 2008	7‐methylxanthine (35)	89%	NR
	Placebo (42)	92%	
**NR:** not reported			

Studies usually assessed adherence to optical interventions through an estimate by parents or children, or both, of wearing time per day of spectacles or contact lenses and the number of days per week the optical appliances were worn (6 to 7 days per week was usually judged as being fully compliant). Studies used a variety of methods for data collection, including questionnaires or discussion of compliance at follow‐up appointments. These data were available for 1731 participants wearing a variety of spectacle lens interventions (undercorrection, multifocal, peripheral plus and single vision). The range of daily wearing times were between 13.1 and 15.5 hours per day. The percentage of participants who were judged to be compliant were similar between test and control lenses, although the COMET Study 2003 and Pärssinen 1989 reported a lower proportion of participants wearing MFSL than the SVL controls.

Adherence data were available for 873 participants in contact lens studies, including MFSCL, SVSCL and RGP. Daily wearing times were between 6.3 and 13.7 hours per day, with no statistical differences between multifocal and single vision contact lenses. In Katz 2003, which investigated RGP lenses versus SVLs, the percentage compliance at 24 months in the RGP group was 31.5% compared to 98.4% in spectacle lens‐wearing controls. Adherence was not formally assessed in orthokeratology studies, but these studies were often associated with high dropout rates (over 50% in some studies).

For most pharmacological interventions, adherence was monitored by self‐reported questionnaires. Only PIR‐205 Study 2004 used electronic monitoring. Compliance was defined as using the study medication 75% to 80% of the time. Percentage compliance in 919 participants taking low‐dose atropine ranged from 83.3% to 98.8%, with similar levels of compliance between active and placebo arms. Compliance in the intervention arm of PIR‐205 Study 2004 and Trier 2008 were 79% and 89% respectively, which were similar to participants taking the placebo.

## Discussion

### Summary of main results

This review summarises evidence from 64 studies, involving a total of 11,617 participants with low to moderate myopia. Studies investigated 11 interventions to slow the progression of myopia in children. Participants were girls and boys aged between 4 and 18 years, with an average age of 10.4 years. Interventions were broadly categorised into optical, pharmacological and environmental modalities. Fifty‐seven studies compared one or more myopia control interventions relative to a control or placebo intervention. Four studies included a combined intervention arm compared to control, and seven studies compared single or combined interventions to each other. Over 60% of studies were conducted in China or other Asian countries. In terms of study duration, 34% of the studies had a 12‐month duration, 46% reported to 24 months, 17% up to 36 months and only one study measured outcomes over 36 months. We defined the critical outcome 'progression of myopia' as both change in refractive error (as SER from baseline) and the more clinically meaningful, change in axial length. We judged most of the studies to be at 'high' or 'some concern' for risk of bias. Because the network was not well‐connected, we based our comparisons on direct evidence from classical pairwise meta‐analyses, except for moderate‐dose atropine. 

In terms of SER and axial length at 12 and 24 months, all interventions, except for undercorrection with SVLs, RGP and the adenosine antagonist 7‐methylxanthine, were superior to placebo in reducing the change in SER and slowing axial elongation. The certainty of evidence ranged from very low to moderate, depending on the comparison (see Table 1; Table 2; Table 3; Table 4). Although statistically significant, many of the efficacy estimates were small and clinically insignificant. There was evidence of retardation in efficacy of myopia control treatment over time, with most of the reduction in progression occurring in the first year.

Overall, high‐dose topical atropine (≥ 0.5 %) and orthokeratology were the most effective interventions in slowing axial elongation at two years of follow‐up, corresponding to a 0.3 mm to 0.5 mm slowing of axial elongation (moderate‐certainty evidence). MFSCLs were similar to low‐dose topical atropine (< 0.1%), with a reduction in axial elongation of 0.15 mm and 0.16 mm respectively (moderate‐certainty evidence).

The most commonly studied combination therapy was orthokeratology plus low‐dose atropine. Compared to orthokeratology monotherapy, the combination was associated with a significant reduction in axial elongation. 

We did not identify any relevant studies reporting on the effect of environmental interventions on childhood myopia progression.

Data on changes to SER and axial length following cessation of treatment ('rebound') were available in four studies. There was no evidence of rebound in two studies that investigated optical interventions (Ruiz‐Pomeda 2018; STAMP Study 2012), but there was inconsistent evidence on rebound for topical atropine. One study, which abruptly terminated 1% topical atropine, found that there was a significant rebound effect (ATOM Study 2006), whilst another, which reduced the frequency of atropine instillation over time, reported a maintained slowing of myopia progression (Zhu 2021).

In terms of the risk of adverse events, based on limited evidence that was often poorly reported, spectacle interventions were well tolerated with minimal and mild adverse events, similar to controls. In contact lens studies, the incidence of adverse events for multifocal soft contact lenses was also similar to the single vision soft contact lens controls. Adverse events in these studies generally consisted of expected contact lens‐related adverse events that were generally non‐serious. However, the incidence and severity of corneal staining was higher in studies using orthokeratology. The most commonly reported adverse events with antimuscarinic agents were photophobia, blurred near vision and hypersensitivity reactions, which increased with increasing drug concentration.

Treatment adherence was generally high with levels of adherence that were similar across study arms.

Only one study provided information on the effect of myopia control treatment on vision‐specific quality of life (LAMP Study 2019). This study compared three doses of atropine to placebo and reported no difference between groups at 12 months.

#### Brief economic commentary

We found no economic evaluation studies comparing different methods of myopia control in children. The apparent shortage of relevant economic evaluations indicates that there is a paucity of evidence regarding the costs and consequences of measures of myopia control in children. Future research could consider economic as well as clinical evaluation of interventions for myopia control. 

### Overall completeness and applicability of evidence

Several factors limit the applicability of the evidence in our review. Although we were able to include evidence from 64 RCTs, approximately 80% of the studies followed participants for two years or less. A consensus report produced by the International Myopia Institute (IMI), guiding principles of myopia control clinical study design, recommend three years as the minimum length to assess the efficacy of a treatment for myopia control, since treatment needs to be applied over multiple years during the period of most rapid myopia progression (Wolffsohn 2019 ). Extrapolation of efficacy data for outcomes measured at one year is therefore likely to overestimate the effectiveness of treatment.

A number of factors complicated the comparison of studies, including differences in the demographic characteristics of the participants, and variability in the parameters used within similar treatments (e.g. different add powers and lens designs for multifocal spectacles and soft contact lenses and variable doses of atropine). Although the majority of studies adopted similar eligibility criteria, recruiting children aged 6 to 13 years, other studies used a wider age range of up to 18 years. This is important since faster progression occurs in younger myopes and progression slows in older teenagers. Furthermore, studies were conducted in different ethnic groups, particularly in children from South East Asian countries that typically have faster progression of myopia (Morgan 2012; Morgan 2018).

These factors may, at least in part, explain the considerable heterogeneity of treatment effects identified in the review for some comparisons. 

It was difficult to compare the incidence of adverse events across studies due to different methods used to classify and report them. Furthermore, the use of photochromic and multifocal spectacles in pharmacological studies to mitigate potential side effects of higher topical atropine doses (≥ 0.5%) may have underestimated the incidence of glare, photophobia and reading difficulties reported in these studies. Similarly, the evaluation of treatment adherence between studies was complicated by the use of different methods to measure compliance (e.g. retrospective self‐report by parents or children, questionnaires or diaries). Lastly, the short time frame of many studies may have overestimated compliance, since it is possible that compliance may reduce over time.

### Quality of the evidence

The certainty of the evidence for the critical outcome 'Progression of myopia' at one and two years ranged from very low to moderate, depending on the intervention. The main reasons for downgrading the certainty of evidence was risk of bias (principally due to lack of reporting details) and unexplained heterogeneity or inconsistency in the results.

We were unable to conduct a quantitative analysis for other outcomes and we summarised the results for these outcomes at study level in summary tables with an indication of the overall risk of bias for each of the included studies.

### Potential biases in the review process

We followed the *Cochrane Handbook for Systematic Reviews of Interventions *to conduct this systematic review (Higgins 2022c). We applied a broad search strategy to ensure that all relevant papers were included. Pairs of review authors independently extracted data and assessed risk of bias; we also followed prespecified methods for classical and network meta‐analyses. We therefore believe that there should be no bias in the review process with respect to study selection and analysis of available data. 

Whenever possible, we used random‐effects pairwise meta‐analyses to incorporate heterogeneity amongst studies. Since we were unable to carry out the planned subgroup and sensitivity analyses to explore heterogeneity, the presence of considerable unexplained heterogeneity for several comparisons reduces our confidence in effect estimates.

We judged many of the RoB 2 assessments as ‘some concerns’ across the studies in our review, which often reflected an inadequate reporting of information by the study authors, for example, no information on allocation concealment and lack of a prespecified analysis plan. Consequently, we may have overestimated the impact of bias on our findings by downgrading the certainty of evidence of the critical and important outcomes due to risk of bias.

### Agreements and disagreements with other studies or reviews

A previous Cochrane systematic review on myopia control interventions in children (Walline 2020), reviewed evidence from 41 RCTs and concluded, similar to the current review, that there was moderate‐certainty evidence favouring antimuscarinic drugs to reduce myopia progression and axial elongation with inconclusive evidence for other interventions. By contrast, a NMA of 16 interventions for myopia control in children conducted in 2016 concluded that "a range of interventions can significantly reduce myopia progression when compared with single vision spectacle lenses or placebo" (Huang 2016). More recently, a systematic review and NMA comparing the efficacy and safety of different concentrations of topical atropine for myopia control reported that 1%, 0.5% and 0.05% atropine were the three most efficacious atropine concentrations (Ha 2022). 

Two systematic reviews and an Ophthalmic Technology Assessment by the American Academy of Ophthalmology (AAO) have considered safety outcomes of contact lens interventions for reducing myopia progression in children (Cheng 2020; VanderVeen 2019; Yu 2022). With respect to daily disposable soft contact lenses, a review of retrospective data of adverse events from six RCTs estimated an incidence of 4.5 adverse events per 100 patient years and suggested that soft contact lenses can be safely worn by children (Cheng 2020). Yu 2022 analysed data from three studies and found no difference in adverse events between MFSCLs and control single vision lenses. VanderVeen 2019 reviewed published evidence on orthokeratology treatment for an AAO Health Technology Assessment and identified a sparsity of evidence in paediatric populations; it was noted that orthokeratology carries a small but definite risk of sight‐threatening keratitis. Bullimore 2013 estimated the incidence of microbial keratitis associated with orthokeratology as 13.9 per 10,000 patient‐years (95% CI 1.7 to 50.4), which is similar to the overall incidence of microbial keratitis in overnight soft contact lens wear.

In their NMA of efficacy and safety of topical atropine for myopia control, Huang 2016 considered the safety profiles of different atropine concentrations based on changes in pupil size and accommodation. The authors found that based on these proxies for photophobia and near vision difficulties, lower atropine concentrations had higher safety ranking probabilities.

## Authors' conclusions

**Implications for practice**

Based on the best available evidence, topical antimuscarinic agents and orthokeratology (ortho‐K) currently appear to be the most effective treatments for slowing childhood myopia progression. There is some uncertainty as to the optimal dose of atropine, the most studied antimuscarinic agent. Although higher doses slow overall axial elongation by approximately 0.5 mm over two years, corresponding to an approximate 1.00 D reduction in myopia, higher concentrations are more likely to cause adverse events and may increase the risk of rebound following cessation of treatment. The current review found limited evidence that rebound could potentially be reduced by tapering the treatment prior to termination. 

There are logistical difficulties in assessing change in refractive error in ortho‐K studies and therefore evidence of efficacy is based on slowing axial elongation. Ortho‐k may also require more specialised knowledge by the eye care practitioner, and therefore it may not be as available as some of the other treatment modalities

Evidence on the efficacy of other treatments was limited by short study durations and considerable heterogeneity in treatment response. The finding that treatment efficacy reduces over time would add to the perception of greater efficacy in studies of short duration.

Uncertainty remains regarding the risk‐benefit of ortho‐K and other contact lens interventions in children. Adverse events across the included studies were generally poorly described with a lack of standardisation of reporting.  Although none of the included studies reported serious adverse events, the duration of follow‐up in trials may have been insufficient to capture long‐term or rare adverse events.

Myopia control is a rapidly moving field, which emphasises the need for a living systematic review in this area that is underpinned by continual and active monitoring of new evidence.

**Implications for research**

There are a number of research priorities in this field. Epidemiological evidence has shown that the age of onset and rate of myopia progression in children varies considerably. There is a need to develop better predictive models to identify children who are most likely to progress rapidly and will therefore potentially derive most benefit from treatment. The absence of long‐term data provides little evidence as to when myopia control interventions can be stopped or modified during treatment.

Although topical atropine shows considerable promise as a treatment for myopia control, the optimal dose is yet to be established, which balances efficacy, safety and propensity to rebound. There are many ongoing trials investigating the efficacy of various doses of atropine, used either as monotherapy or in combination with another intervention, which may provide further data to determine the optimal drug dose for myopia control.

The International Myopia Institute has developed a consensus set of principles on study design to guide the development of myopia control trial protocols (Wolffsohn 2019). Many of the included trials in this review did not meet these recommendations and researchers should be encouraged to adopt these principles to facilitate harmonised reporting of outcomes, including standardised reporting of AEs. There was also a tendency for authors to report a relative percentage reduction in myopia progression to express treatment effect, which can be misleading.

To address uncertainty in the safety of myopia control interventions, particularly relating to rare and potentially sight‐threatening adverse events, it may be necessary to seek evidence from non‐randomised studies, since such events are unlikely to be seen in randomised controlled trials due to their small size and relatively short duration. Future systematic reviews considering safety could also, therefore, consider evidence from non‐randomised studies for a comprehensive evaluation of safety.

Only one of the included studies evaluated the impact of myopia control interventions on quality of life. There is therefore a need for further studies using validated instruments to measure vision‐related and health‐related quality of life as an outcome of myopia control studies.There is also a lack of health economic studies that could inform policy‐makers and healthcare decision‐makers, enabling them to identify which interventions, policies or services provide the best value for money.

## History

Protocol first published: Issue 4, 2021

## Risk of bias

**Table:** Risk of bias for analysis 1.1 Change in refractive error from baseline

**Study**	**Bias**											
	**Randomisation process**		**Deviations from intended interventions**		**Missing outcome data**		**Measurement of the outcome**		**Selection of the reported results**		**Overall**	
	**Authors' judgement**	**Support for judgement**	**Authors' judgement**	**Support for judgement**	**Authors' judgement**	**Support for judgement**	**Authors' judgement**	**Support for judgement**	**Authors' judgement**	**Support for judgement**	**Authors' judgement**	**Support for judgement**
**Subgroup 1.1.1 At 1 year**												
Adler 2006	Low risk of bias	Randomisation was performed by tossing a coin. Allocation performed by someone uninvolved in the project. There were no baseline imbalances that would suggest a problem with randomisation.	Some concerns	It was not possible to mask the participants or investigators providing clinical care given the nature of the interventions but investigating optometrists were masked from previous results. Results analysed using ITT principles without imputation of missing data.	Some concerns	Outcome data available for 77.4% of randomised participants. No analysis to correct for bias due to missing data. Missingness balanced across arms. Three non‐compliant participants were excluded.	High risk of bias	Inappropriate method used for outcome measurement (non‐cycloplegic refraction).	Some concerns	No pre‐registered method (registry or protocol) available.	High risk of bias	The study is judged to be at high risk of bias in at least one domain for this result.
Chung 2002	Some concerns	Randomisation was performed using a block randomisation technique. Once participants were paired, allocation to intervention and control using a coin toss. There were no baseline imbalances that would suggest a problem with randomisation.	Some concerns	It was not possible to mask the participants, carers or investigating clinicians. Deviations from intended interventions are not reported and the analysis was appropriate.	High risk of bias	Outcome data were not available for all, or nearly all, randomized participants, and there is no evidence that the result was not biased by missing outcome data, and no information on whether missingness in the outcome could depend on its true value.	Low risk of bias	Appropriate methods of measuring outcome data were used. Same study protocol for all participants and the team were blinded to the group allocation of patients.	Some concerns	No pre‐registered method (registry or protocol) available.	High risk of bias	The study is judged to be at high risk of bias in at least one domain for this result.
**Subgroup 1.1.2 At 2 years**												
Chung 2002	Some concerns	Randomisation was performed using a block randomisation technique. Once participants were paired, allocation to intervention and control using a coin toss. There were no baseline imbalances that would suggest a problem with randomisation.	Some concerns	It was not possible to mask the participants, carers or investigating clinicians. Deviations from intended interventions are not reported and the analysis was appropriate.	High risk of bias	Outcome data were not available for all, or nearly all, randomized participants, and there is no evidence that the result was not biased by missing outcome data, and no information on whether missingness in the outcome could depend on its true value.	Low risk of bias	Appropriate methods of measuring outcome data were used. Same study protocol for all participants and the team were blinded to the group allocation of patients.	Some concerns	No pre‐registered method (registry or protocol) available.	High risk of bias	The study is judged to be at high risk of bias in at least one domain for this result.
Koomson 2016	Low risk of bias	Randomisation was performed using the permuted block method and tossing a coin. Sealed envelopes to conceal allocation. Baseline differences did not suggest a problem with the randomisation process.	Low risk of bias	It was not possible to mask the participants due to functional differences in interventions being studied. Investigator providing clinical care and obtaining measurements were masked to group allocation. There were no deviations from intended interventions. Analysis were performed on the ITT principles.	Low risk of bias	Data available for all participants.	Low risk of bias	Appropriate methods of measuring outcome data were used. Same study protocol for all participants and the team were blinded to the group allocation of patients.	Some concerns	No pre‐registered method (registry or protocol) available.	Some concerns	The study is judged to raise some concerns in at least one domain for this result, but not to be at high risk of bias for any domain.

**Table:** Risk of bias for analysis 1.2 Change in axial length from baseline

**Study**	**Bias**											
	**Randomisation process**		**Deviations from intended interventions**		**Missing outcome data**		**Measurement of the outcome**		**Selection of the reported results**		**Overall**	
	**Authors' judgement**	**Support for judgement**	**Authors' judgement**	**Support for judgement**	**Authors' judgement**	**Support for judgement**	**Authors' judgement**	**Support for judgement**	**Authors' judgement**	**Support for judgement**	**Authors' judgement**	**Support for judgement**
**Subgroup 1.2.1 At 1 year**												
Chung 2002	Some concerns	Randomisation was performed using a block randomisation technique. Once participants were paired, allocation to intervention and control using a coin toss. There were no baseline imbalances that would suggest a problem with randomisation.	Some concerns	It was not possible to mask the participants,carers or investigating clinicians. Deviations from intended interventions are not reported and the method of analysis was not reported.	High risk of bias	Outcome data were not available for all, or nearly all, randomized participants, and there is no evidence that the result was not biased by missing outcome data, and no information on whether missingness in the outcome could depend on its true value.	Low risk of bias	Appropriate methods of measuring outcome data were used. Same study protocol for all participants and the team were blinded to the group allocation of patients.	Some concerns	No pre‐registered method (registry or protocol) available.	High risk of bias	The study is judged to be at high risk of bias in at least one domain for this result.
**Subgroup 1.2.2 At 2 years**												
Chung 2002	Some concerns	Randomisation was performed using a block randomisation technique. Once participants were paired, allocation to intervention and control using a coin toss. There were no baseline imbalances that would suggest a problem with randomisation.	Some concerns	It was not possible to mask the participants,carers or investigating clinicians. Deviations from intended interventions are not reported and the method of analysis was not reported.	High risk of bias	Outcome data were not available for all, or nearly all, randomized participants, and there is no evidence that the result was not biased by missing outcome data, and no information on whether missingness in the outcome could depend on its true value.	Low risk of bias	Appropriate methods of measuring outcome data were used. Same study protocol for all participants and the team were blinded to the group allocation of patients.	Some concerns	No pre‐registered method (registry or protocol) available.	High risk of bias	The study is judged to be at high risk of bias in at least one domain for this result.
Koomson 2016	Low risk of bias	Randomisation was performed using the permuted block method and tossing a coin. Sealed envelopes to conceal allocation. Baseline differences did not suggest a problem with the randomisation process.	Low risk of bias	It was not possible to mask the participants due to functional differences in interventions being studied. Investigator providing clinical care and obtaining measurements were masked to group allocation. There were no deviations from intended interventions. Analysis were performed on the ITT principles.	Low risk of bias	Data available for all participants.	Low risk of bias	Appropriate methods of measuring outcome data were used. Same study protocol for all participants and the team were blinded to the group allocation of patients.	Some concerns	No pre‐registered method (registry or protocol) available.	Some concerns	The study is judged to raise some concerns in at least one domain for this result, but not to be at high risk of bias for any domain.

**Table:** Risk of bias for analysis 2.1 Change in refractive error from baseline

**Study**	**Bias**											
	**Randomisation process**		**Deviations from intended interventions**		**Missing outcome data**		**Measurement of the outcome**		**Selection of the reported results**		**Overall**	
	**Authors' judgement**	**Support for judgement**	**Authors' judgement**	**Support for judgement**	**Authors' judgement**	**Support for judgement**	**Authors' judgement**	**Support for judgement**	**Authors' judgement**	**Support for judgement**	**Authors' judgement**	**Support for judgement**
**Subgroup 2.1.1 At 1 year**												
Cheng 2010	High risk of bias	Randomisation was performed by randomly drawing file numbers written on slips of paper from a container. There was no allocation concealment and no analysis to investigate baseline differences that arose as a result of the allocation process.	High risk of bias	It was not possible to mask the participants, carers or investigating optometrists given the nature of the interventions. Nine participants and their parents did not accept their group allocations. Results analysed using ITT principles.	High risk of bias	Outcome data were not available for all, or nearly all, randomized participants, and there is no evidence that the result was not biased by missing outcome data, and no information on whether missingness in the outcome could depend on its true value.	Low risk of bias	Appropriate and comparable methods of measuring outcome data were used. Outcome assessors are likely to have been aware of the group allocation, but bias was minimised by using objective measurements. Therefore, it is unlikely that the outcome was influenced by knowledge of the intervention received.	Some concerns	Protocol registered. However, not enough information in the protocol on a statistical plan and no published statistical plan found.	High risk of bias	The study is judged to be at high risk of bias in at least one domain for this result.
COMET2 Study 2011	Some concerns	Randomisation was performed using a permuted block design. Allocation concealment was not reported. There were no baseline imbalances that would suggest a problem with randomisation.	Low risk of bias	Participants, carers and those delivering the intervention were unaware of intervention received. Results analysed using ITT principles	Low risk of bias	Data available for approx 93% of participants.	Low risk of bias	Appropriate methods of measuring outcome data were used. Same study protocol for all participants and the team were blinded to the group allocation of patients.	Some concerns	Protocol registered. Not enough information in the protocol on a statistical plan and no published statistical plan found.	Some concerns	The study is judged to raise some concerns in at least one domain for this result, but not to be at high risk of bias for any domain.
COMET Study 2003	Low risk of bias	Randomisation was stratified by coordinating center, using a random permuted block design. There were no baseline imbalances that would suggest a problem with randomisation.	Low risk of bias	It was not possible to mask the participants due to functional differences in interventions being studied. Investigator providing clinical care and obtaining measurements were masked to group allocation. There were no deviations from intended interventions. Analysis were performed on the modified ITT principles.	Low risk of bias	Data available for approx 98% of participants.	Low risk of bias	Appropriate and comparable methods of measuring outcome data were used. Outcome assessors may have been aware of the group allocation, but bias is minimised by using objective measurements. Therefore, it is unlikely that the outcome was influenced by knowledge of the intervention received.	Some concerns	Protocol registered. Not enough information in the protocol on a statistical plan and no published statistical plan found.	Some concerns	The study is judged to raise some concerns in at least one domain for this result, but not to be at high risk of bias for any domain.
Edwards 2002	Some concerns	Randomisation was performed using a pre‐determined randomisation sequence. Allocation concealment was not reported. There were no significant differences in the baseline parameters that would suggest a problem with randomisation.	Some concerns	It was not possible to mask the participants due to functional differences in interventions being studied. Investigator providing clinical care and obtaining measurements were masked to group allocation. Deviations from intended interventions were not reported. Only participants who completed the study were analysed.	Low risk of bias	Outcome data available for 85.2% of randomised participants. No analysis to correct for bias due to missing data. Missingness balanced across arms.	Low risk of bias	Appropriate methods of measuring outcome data were used. Same study protocol for all participants and the team were blinded to the group allocation of patients.	Some concerns	No pre‐registered method (registry or protocol) available.	Some concerns	The study is judged to raise some concerns in at least one domain for this result, but not to be at high risk of bias for any domain.
Fulk 2002	Some concerns	Randomisation was performed using the permuted block method and tossing a coin. Allocation concelament was maintained until after enrolment. There were baseline differences for initial myopia. There were no baseline differences for age, gender, axial length and vitreous chamber.	Low risk of bias	It was not possible to mask the participants due to functional differences in interventions being studied. Investigator providing clinical care and obtaining measurements were masked to group allocation. There were no deviations from intended interventions. Analysis were performed on the Intention to Treat Principles.	Low risk of bias	Outcome data were not available for all, or nearly all, randomized participants, and there is no evidence that the result was biased by missing outcome data, and unlikely that missingness in the outcome depended on its true value.	Low risk of bias	Appropriate and comparable methods of measuring outcome data were used. Outcome assessors may have been aware of the group allocation, but bias is minimised by using objective measurements. Therefore, it is unlikely that the outcome was influenced by knowledge of the intervention received.	Some concerns	No pre‐registered method (registry or protocol) available.	Some concerns	The study is judged to raise some concerns in at least one domain for this result, but not to be at high risk of bias for any domain.
Jensen 1991	Some concerns	Randomisation was performed using block randomisation. There was no information relating to allocation concealment. There were no baseline imbalances that would suggest a problem with randomisation.	Low risk of bias	It was not possible to mask the participants due to functional differences in interventions being studied. Masking of the investigatoris not reported. There were no deviations from intended interventions. Analysis were performed on the ITT principles.	Low risk of bias	Outcome data available for 91.8% of randomised participants. No analysis to correct for bias due to missing data. Missingness balanced across arms.	Low risk of bias	Appropriate and comparable methods of measuring outcome data were used. Outcome assessors may have been aware of the group allocation, but bias is minimised by using objective measurements. Therefore, it is unlikely that the outcome was influenced by knowledge of the intervention received.	Some concerns	No pre‐registered method (registry or protocol) available.	Some concerns	The study is judged to raise some concerns in at least one domain for this result, but not to be at high risk of bias for any domain.
MIT Study 2001	Some concerns	Initial stratification based on age, sex and myopic severity followed by randomisation to one of three treatment groups. No information on allocation concealment.	Low risk of bias	It was not possible to mask the participants due to functional differences in interventions being studied. There were no deviations from intended interventions and the analysis was appropriate.	Low risk of bias	Outcome data available for 82.8% of randomised participants. No analysis to correct for bias due to missing data. Missingness balanced across arms.	Low risk of bias	Appropriate and comparable methods of measuring outcome data were used. Outcome assessors may have been aware of the group allocation, but bias is minimised by using objective measurements. Therefore, it is unlikely that the outcome was influenced by knowledge of the intervention received.	Some concerns	No pre‐registered method (registry or protocol) available.	Some concerns	The study is judged to raise some concerns in at least one domain for this result, but not to be at high risk of bias for any domain.
Pärssinen 1989	Low risk of bias	Randomisation was performed using stratified coding. Sealed envelopes for allocation concealment. No information on baseline differences between intervention groups was reported.	Some concerns	It was not possible to mask the participants or investigating clinicians. Deviations from intended interventions arose as a result of the trial context. Data were analysed for participants who completed the study.	Low risk of bias	Data available for approx 98% of participants.	Some concerns	No information provided on the methods used to measure SER (objective or subjective).	Some concerns	No pre‐registered method (registry or protocol) available.	Some concerns	The study is judged to raise some concerns in at least one domain for this result, but not to be at high risk of bias for any domain.
STAMP Study 2012	Low risk of bias	Adaptive randomisation using online software for sequence generation. Allocation was concealed until participants were enrolled and assigned to interventions.	Low risk of bias	Participants, carers and those delivering the intervention were aware of intervention received. Unlikely that ther were deviations from the intended intervention due to trial context. Results analysed using ITT principles.	Low risk of bias	Data available for approx 99% of participants.	Low risk of bias	Appropriate methods of measuring outcome data were used. Same study protocol for all participants and the team were blinded to the group allocation of patients.	Some concerns	Protocol registered. Not enough information in the protocol on a statistical plan and no published statistical plan found.	Some concerns	The study is judged to raise some concerns in at least one domain for this result, but not to be at high risk of bias for any domain.
**Subgroup 2.1.2 At 2 years**												
Cheng 2010	High risk of bias	Randomisation was performed by randomly drawing file numbers written on slips of paper from a container. There was no allocation concealment and no analysis to investigate baseline differences that arose as a result of the allocation process.	High risk of bias	It was not possible to mask the participants, carers or investigating optometrists given the nature of the interventions. Nine participants and their parents did not accept their group allocations. Results analysed using ITT principles.	High risk of bias	Outcome data were not available for all, or nearly all, randomized participants, and there is no evidence that the result was not biased by missing outcome data, and no information on whether missingness in the outcome could depend on its true value.	Low risk of bias	Appropriate and comparable methods of measuring outcome data were used. Outcome assessors are likely to have been aware of the group allocation, but bias was minimised by using objective measurements. Therefore, it is unlikely that the outcome was influenced by knowledge of the intervention received.	Some concerns	Protocol registered. However, not enough information in the protocol on a statistical plan and no published statistical plan found.	High risk of bias	The study is judged to be at high risk of bias in at least one domain for this result.
COMET2 Study 2011	Some concerns	Randomisation was performed using a permuted block design. Allocation concealment was not reported. There were no baseline imbalances that would suggest a problem with randomisation.	Low risk of bias	Participants, carers and those delivering the intervention were unaware of intervention received. Results analysed using ITT principles	Low risk of bias	Data available for approx 93% of participants.	Low risk of bias	Appropriate methods of measuring outcome data were used. Same study protocol for all participants and the team were blinded to the group allocation of patients.	Some concerns	Protocol registered. Not enough information in the protocol on a statistical plan and no published statistical plan found.	Some concerns	The study is judged to raise some concerns in at least one domain for this result, but not to be at high risk of bias for any domain.
COMET Study 2003	Low risk of bias	Randomisation was stratified by coordinating center, using a random permuted block design. There were no baseline imbalances that would suggest a problem with randomisation.	Low risk of bias	It was not possible to mask the participants due to functional differences in interventions being studied. Investigator providing clinical care and obtaining measurements were masked to group allocation. There were no deviations from intended interventions. Analysis were performed on the modified ITT principles.	Low risk of bias	Data available for approx 98% of participants.	Low risk of bias	Appropriate and comparable methods of measuring outcome data were used. Outcome assessors may have been aware of the group allocation, but bias is minimised by using objective measurements. Therefore, it is unlikely that the outcome was influenced by knowledge of the intervention received.	Some concerns	Protocol registered. Not enough information in the protocol on a statistical plan and no published statistical plan found.	Some concerns	The study is judged to raise some concerns in at least one domain for this result, but not to be at high risk of bias for any domain.
Edwards 2002	Some concerns	Randomisation was performed using a pre‐determined randomisation sequence. Allocation concealment was not reported. There were no significant differences in the baseline parameters that would suggest a problem with randomisation.	Some concerns	It was not possible to mask the participants due to functional differences in interventions being studied. Investigator providing clinical care and obtaining measurements were masked to group allocation. Deviations from intended interventions were not reported. Only participants who completed the study were analysed.	Low risk of bias	Outcome data available for 85.2% of randomised participants. No analysis to correct for bias due to missing data. Missingness balanced across arms.	Low risk of bias	Appropriate methods of measuring outcome data were used. Same study protocol for all participants and the team were blinded to the group allocation of patients.	Some concerns	No pre‐registered method (registry or protocol) available.	Some concerns	The study is judged to raise some concerns in at least one domain for this result, but not to be at high risk of bias for any domain.
Fulk 2002	Some concerns	Randomisation was performed using the permuted block method and tossing a coin. Allocation concelament was maintained until after enrolment. There were baseline differences for initial myopia. There were no baseline differences for age, gender, axial length and vitreous chamber.	Low risk of bias	It was not possible to mask the participants due to functional differences in interventions being studied. Investigator providing clinical care and obtaining measurements were masked to group allocation. There were no deviations from intended interventions. Analysis were performed on the Intention to Treat Principles.	Low risk of bias	Outcome data were not available for all, or nearly all, randomized participants, and there is no evidence that the result was biased by missing outcome data, and unlikely that missingness in the outcome depended on its true value.	Low risk of bias	Appropriate and comparable methods of measuring outcome data were used. Outcome assessors may have been aware of the group allocation, but bias is minimised by using objective measurements. Therefore, it is unlikely that the outcome was influenced by knowledge of the intervention received.	Some concerns	No pre‐registered method (registry or protocol) available.	Some concerns	The study is judged to raise some concerns in at least one domain for this result, but not to be at high risk of bias for any domain.
Jensen 1991	Some concerns	Randomisation was performed using block randomisation. There was no information relating to allocation concealment. There were no baseline imbalances that would suggest a problem with randomisation.	Low risk of bias	It was not possible to mask the participants due to functional differences in interventions being studied. Masking of the investigatoris not reported. There were no deviations from intended interventions. Analysis were performed on the ITT principles.	Low risk of bias	Outcome data available for 91.8% of randomised participants. No analysis to correct for bias due to missing data. Missingness balanced across arms.	Low risk of bias	Appropriate and comparable methods of measuring outcome data were used. Outcome assessors may have been aware of the group allocation, but bias is minimised by using objective measurements. Therefore, it is unlikely that the outcome was influenced by knowledge of the intervention received.	Some concerns	No pre‐registered method (registry or protocol) available.	Some concerns	The study is judged to raise some concerns in at least one domain for this result, but not to be at high risk of bias for any domain.
Pärssinen 1989	Low risk of bias	Randomisation was performed using stratified coding. Sealed envelopes for allocation concealment. No information on baseline differences between intervention groups was reported.	Some concerns	It was not possible to mask the participants or investigating clinicians. Deviations from intended interventions arose as a result of the trial context. Data were analysed for participants who completed the study.	Low risk of bias	Data available for approx 98% of participants.	Some concerns	No information provided on the methods used to measure SER (objective or subjective).	Some concerns	No pre‐registered method (registry or protocol) available.	Some concerns	The study is judged to raise some concerns in at least one domain for this result, but not to be at high risk of bias for any domain.
Yang 2009	Some concerns	Participants were randomised though no details of tthe exact method of randomisation are recorded. There was no information relating to allocation concealment. There were no baseline imbalances that would suggest a problem with randomisation.	Low risk of bias	It was not possible to mask the participants due to functional differences in interventions being studied. Investigator providing clinical care and obtaining measurements were masked to group allocation. There were no deviations from intended interventions. Analysis were performed on the ITT principles.	Low risk of bias	Outcome data available for 83.7% of randomised participants. No analysis to correct for bias due to missing data. Missingness balanced across arms.	Low risk of bias	Appropriate methods of measuring outcome data were used. Same study protocol for all participants and the team were blinded to the group allocation of patients.	Some concerns	No pre‐registered method (registry or protocol) available.	Some concerns	The study is judged to raise some concerns in at least one domain for this result, but not to be at high risk of bias for any domain.
**Subgroup 2.1.3 At 3 years**												
Cheng 2010	High risk of bias	Randomisation was performed by randomly drawing file numbers written on slips of paper from a container. There was no allocation concealment and no analysis to investigate baseline differences that arose as a result of the allocation process.	High risk of bias	It was not possible to mask the participants, carers or investigating optometrists given the nature of the interventions. Nine participants and their parents did not accept their group allocations. Results analysed using ITT principles.	High risk of bias	Outcome data were not available for all, or nearly all, randomized participants, and there is no evidence that the result was not biased by missing outcome data, and no information on whether missingness in the outcome could depend on its true value.	Low risk of bias	Appropriate and comparable methods of measuring outcome data were used. Outcome assessors are likely to have been aware of the group allocation, but bias was minimised by using objective measurements. Therefore, it is unlikely that the outcome was influenced by knowledge of the intervention received.	Some concerns	Protocol registered. However, not enough information in the protocol on a statistical plan and no published statistical plan found.	High risk of bias	The study is judged to be at high risk of bias in at least one domain for this result.
COMET2 Study 2011	Some concerns	Randomisation was performed using a permuted block design. Allocation concealment was not reported. There were no baseline imbalances that would suggest a problem with randomisation.	Low risk of bias	Participants, carers and those delivering the intervention were unaware of intervention received. Results analysed using ITT principles	Low risk of bias	Data available for approx 93% of participants.	Low risk of bias	Appropriate methods of measuring outcome data were used. Same study protocol for all participants and the team were blinded to the group allocation of patients.	Some concerns	Protocol registered. Not enough information in the protocol on a statistical plan and no published statistical plan found.	Some concerns	The study is judged to raise some concerns in at least one domain for this result, but not to be at high risk of bias for any domain.
COMET Study 2003	Low risk of bias	Randomisation was stratified by coordinating center, using a random permuted block design. There were no baseline imbalances that would suggest a problem with randomisation.	Low risk of bias	It was not possible to mask the participants due to functional differences in interventions being studied. Investigator providing clinical care and obtaining measurements were masked to group allocation. There were no deviations from intended interventions. Analysis were performed on the modified ITT principles.	Low risk of bias	Data available for approx 98% of participants.	Low risk of bias	Appropriate and comparable methods of measuring outcome data were used. Outcome assessors may have been aware of the group allocation, but bias is minimised by using objective measurements. Therefore, it is unlikely that the outcome was influenced by knowledge of the intervention received.	Some concerns	Protocol registered. Not enough information in the protocol on a statistical plan and no published statistical plan found.	Some concerns	The study is judged to raise some concerns in at least one domain for this result, but not to be at high risk of bias for any domain.
Pärssinen 1989	Low risk of bias	Randomisation was performed using stratified coding. Sealed envelopes for allocation concealment. No information on baseline differences between intervention groups was reported.	Some concerns	It was not possible to mask the participants or investigating clinicians. Deviations from intended interventions arose as a result of the trial context. Data were analysed for participants who completed the study.	Low risk of bias	Data available for approx 98% of participants.	Some concerns	No information provided on the methods used to measure SER (objective or subjective).	Some concerns	No pre‐registered method (registry or protocol) available.	Some concerns	The study is judged to raise some concerns in at least one domain for this result, but not to be at high risk of bias for any domain.

**Table:** Risk of bias for analysis 2.2 Change in axial length from baseline

**Study**	**Bias**											
	**Randomisation process**		**Deviations from intended interventions**		**Missing outcome data**		**Measurement of the outcome**		**Selection of the reported results**		**Overall**	
	**Authors' judgement**	**Support for judgement**	**Authors' judgement**	**Support for judgement**	**Authors' judgement**	**Support for judgement**	**Authors' judgement**	**Support for judgement**	**Authors' judgement**	**Support for judgement**	**Authors' judgement**	**Support for judgement**
**Subgroup 2.2.1 At 1 year**												
Cheng 2010	High risk of bias	Randomisation was performed by randomly drawing file numbers written on slips of paper from a container. There was no allocation concealment and no analysis to investigate baseline differences that arose as a result of the allocation process.	High risk of bias	It was not possible to mask the participants,carers or investigating optometrists given the nature of the interventions. Nine participants and their parents did not accept their group allocations. Results analysed using ITT principles.	High risk of bias	Outcome data were not available for all, or nearly all, randomized participants, and there is no evidence that the result was not biased by missing outcome data, and no information on whether missingness in the outcome could depend on its true value.	Low risk of bias	Appropriate and comparable methods of measuring outcome data were used. Outcome assessors are likely to have been aware of the group allocation, but bias was minimised by using objective measurements. Therefore, it is unlikely that the outcome was influenced by knowledge of the intervention received.	Some concerns	Protocol registered. Not enough information in the protocol on a statistical plan and no published statistical plan found.	High risk of bias	The study is judged to be at high risk of bias in at least one domain for this result.
COMET Study 2003	Low risk of bias	Randomisation was stratified by coordinating center, using a random permuted block design. There were no baseline imbalances that would suggest a problem with randomisation.	Low risk of bias	It was not possible to mask the participants due to functional differences in interventions being studied. Investigator providing clinical care and obtaining measurements were masked to group allocation. There were no deviations from intended interventions. Analysis were performed on the modified ITT principles.	Low risk of bias	Data available for approx 98% of participants.	Low risk of bias	Appropriate and comparable methods of measuring outcome data were used. Outcome assessors may have been aware of the group allocation, but bias is minimised by using objective measurements. Therefore, it is unlikely that the outcome was influenced by knowledge of the intervention received.	Some concerns	Protocol registered. Not enough information in the protocol on a statistical plan and no published statistical plan found.	Some concerns	The study is judged to raise some concerns in at least one domain for this result, but not to be at high risk of bias for any domain.
Edwards 2002	Some concerns	Randomisation was performed using a pre‐determined randomisation sequence. Allocation concealment was not reported. There were no significant differences in the baseline parameters that would suggest a problem with randomisation.	Some concerns	It was not possible to mask the participants due to functional differences in interventions being studied. Investigator providing clinical care and obtaining measurements were masked to group allocation. Deviations from intended interventions were not reported. Only participants who completed the study were analysed.	Low risk of bias	Outcome data available for 85.2% of randomised participants. No analysis to correct for bias due to missing data. Missingness balanced across arms.	Low risk of bias	Appropriate methods of measuring outcome data were used. Same study protocol for all participants and the team were blinded to the group allocation of patients.	Some concerns	No pre‐registered method (registry or protocol) available.	Some concerns	The study is judged to raise some concerns in at least one domain for this result, but not to be at high risk of bias for any domain.
STAMP Study 2012	Low risk of bias	Adaptive randomisation using online software for sequence generation. Allocation was concealed until participants were enrolled and assigned to interventions.	Low risk of bias	Participants, carers and those delivering the intervention were aware of intervention received. It is unlikely that there were deviations from the intended intervention that arose due to trial context. Results analysed using ITT principles.	Low risk of bias	Data available for approx 99% of participants.	Low risk of bias	Appropriate methods of measuring outcome data were used. Same study protocol for all participants and the team were blinded to the group allocation of patients.	Some concerns	Protocol registered. Not enough information in the protocol on a statistical plan and no published statistical plan found.	Some concerns	The study is judged to raise some concerns in at least one domain for this result, but not to be at high risk of bias for any domain.
**Subgroup 2.2.2 At 2 years**												
Cheng 2010	High risk of bias	Randomisation was performed by randomly drawing file numbers written on slips of paper from a container. There was no allocation concealment and no analysis to investigate baseline differences that arose as a result of the allocation process.	High risk of bias	It was not possible to mask the participants,carers or investigating optometrists given the nature of the interventions. Nine participants and their parents did not accept their group allocations. Results analysed using ITT principles.	High risk of bias	Outcome data were not available for all, or nearly all, randomized participants, and there is no evidence that the result was not biased by missing outcome data, and no information on whether missingness in the outcome could depend on its true value.	Low risk of bias	Appropriate and comparable methods of measuring outcome data were used. Outcome assessors are likely to have been aware of the group allocation, but bias was minimised by using objective measurements. Therefore, it is unlikely that the outcome was influenced by knowledge of the intervention received.	Some concerns	Protocol registered. Not enough information in the protocol on a statistical plan and no published statistical plan found.	High risk of bias	The study is judged to be at high risk of bias in at least one domain for this result.
COMET Study 2003	Low risk of bias	Randomisation was stratified by coordinating center, using a random permuted block design. There were no baseline imbalances that would suggest a problem with randomisation.	Low risk of bias	It was not possible to mask the participants due to functional differences in interventions being studied. Investigator providing clinical care and obtaining measurements were masked to group allocation. There were no deviations from intended interventions. Analysis were performed on the modified ITT principles.	Low risk of bias	Data available for approx 98% of participants.	Low risk of bias	Appropriate and comparable methods of measuring outcome data were used. Outcome assessors may have been aware of the group allocation, but bias is minimised by using objective measurements. Therefore, it is unlikely that the outcome was influenced by knowledge of the intervention received.	Some concerns	Protocol registered. Not enough information in the protocol on a statistical plan and no published statistical plan found.	Some concerns	The study is judged to raise some concerns in at least one domain for this result, but not to be at high risk of bias for any domain.
Edwards 2002	Some concerns	Randomisation was performed using a pre‐determined randomisation sequence. Allocation concealment was not reported. There were no significant differences in the baseline parameters that would suggest a problem with randomisation.	Some concerns	It was not possible to mask the participants due to functional differences in interventions being studied. Investigator providing clinical care and obtaining measurements were masked to group allocation. Deviations from intended interventions were not reported. Only participants who completed the study were analysed.	Low risk of bias	Outcome data available for 85.2% of randomised participants. No analysis to correct for bias due to missing data. Missingness balanced across arms.	Low risk of bias	Appropriate methods of measuring outcome data were used. Same study protocol for all participants and the team were blinded to the group allocation of patients.	Some concerns	No pre‐registered method (registry or protocol) available.	Some concerns	The study is judged to raise some concerns in at least one domain for this result, but not to be at high risk of bias for any domain.
**Subgroup 2.2.3 At 3 years**												
Cheng 2010	High risk of bias	Randomisation was performed by randomly drawing file numbers written on slips of paper from a container. There was no allocation concealment and no analysis to investigate baseline differences that arose as a result of the allocation process.	High risk of bias	It was not possible to mask the participants,carers or investigating optometrists given the nature of the interventions. Nine participants and their parents did not accept their group allocations. Results analysed using ITT principles.	High risk of bias	Outcome data were not available for all, or nearly all, randomized participants, and there is no evidence that the result was not biased by missing outcome data, and no information on whether missingness in the outcome could depend on its true value.	Low risk of bias	Appropriate and comparable methods of measuring outcome data were used. Outcome assessors are likely to have been aware of the group allocation, but bias was minimised by using objective measurements. Therefore, it is unlikely that the outcome was influenced by knowledge of the intervention received.	Some concerns	Protocol registered. Not enough information in the protocol on a statistical plan and no published statistical plan found.	High risk of bias	The study is judged to be at high risk of bias in at least one domain for this result.
COMET Study 2003	Low risk of bias	Randomisation was stratified by coordinating center, using a random permuted block design. There were no baseline imbalances that would suggest a problem with randomisation.	Low risk of bias	It was not possible to mask the participants due to functional differences in interventions being studied. Investigator providing clinical care and obtaining measurements were masked to group allocation. There were no deviations from intended interventions. Analysis were performed on the modified ITT principles.	Low risk of bias	Data available for approx 98% of participants.	Low risk of bias	Appropriate and comparable methods of measuring outcome data were used. Outcome assessors may have been aware of the group allocation, but bias is minimised by using objective measurements. Therefore, it is unlikely that the outcome was influenced by knowledge of the intervention received.	Some concerns	Protocol registered. Not enough information in the protocol on a statistical plan and no published statistical plan found.	Some concerns	The study is judged to raise some concerns in at least one domain for this result, but not to be at high risk of bias for any domain.

**Table:** Risk of bias for analysis 2.3 Change in refractive error following cessation of treatment (1 year)

**Study**	**Bias**											
	**Randomisation process**		**Deviations from intended interventions**		**Missing outcome data**		**Measurement of the outcome**		**Selection of the reported results**		**Overall**	
	**Authors' judgement**	**Support for judgement**	**Authors' judgement**	**Support for judgement**	**Authors' judgement**	**Support for judgement**	**Authors' judgement**	**Support for judgement**	**Authors' judgement**	**Support for judgement**	**Authors' judgement**	**Support for judgement**
STAMP Study 2012	Low risk of bias	Adaptive randomisation using online software for sequence generation. Allocation was concealed until participants were enrolled and assigned to interventions.	Low risk of bias	Participants, carers and those delivering the intervention were aware of the intervention received. It is unlikely that there were deviations from the intended intervention that arose due to trial context. Results analysed using ITT principles	Low risk of bias	Data available for approx. 99% of participants	Low risk of bias	Appropriate methods of measuring outcomes were used.	Some concerns	Protocol registered. Not enough infformation in the protocol on a statistical plan and no published statistical plan found.	Some concerns	The study is judged to raise some concerns in at least one domain for this result, but not to be at high risk of bias for any domain.

**Table:** Risk of bias for analysis 3.1 Change in refractive error from baseline

**Study**	**Bias**											
	**Randomisation process**		**Deviations from intended interventions**		**Missing outcome data**		**Measurement of the outcome**		**Selection of the reported results**		**Overall**	
	**Authors' judgement**	**Support for judgement**	**Authors' judgement**	**Support for judgement**	**Authors' judgement**	**Support for judgement**	**Authors' judgement**	**Support for judgement**	**Authors' judgement**	**Support for judgement**	**Authors' judgement**	**Support for judgement**
**Subgroup 3.1.1 At 1 year**												
Bao 2021	Some concerns	Adaptive randomisation with online software for sequence generation. No information relating to whether allocation was concealed until participants were enrolled and assigned to interventions. Observed imbalances of higher proportion of girls in one group and shorter AL could have occurred by chance.	Low risk of bias	Spectacles were not labelled to identify the treatment group assigned. Losses to follow up documented and no deviations due to trial context. Modified ITT analysis used to estimate the effect of assignment to intervention.	Low risk of bias	Data available for approx 95% of participants.	Low risk of bias	Methods of outcome measurement were appropriate. Comparable methods of outcome assessment were used. Assessors were masked to the intervention status.	Some concerns	Primary outcomes analysed as pre‐specified in trial registry.Primary outcomes analysed as pre‐specified in trial registry. No information to judge whether this result was analysed in accordance with a pre‐specified analysis plan	Some concerns	The study is judged to raise some concerns in at least one domain for this result, but not to be at high risk of bias for any domain.
Han 2018	Some concerns	There was no information on the method of randomisation. There were no significant differences in the baseline parameters that would suggest a problem with randomisation.	Some concerns	It was not possible to mask the participants or investigating clinicians. Deviations from intended interventions are not reported and the analysis was appropriate.	Low risk of bias	Data available for all participants.	Some concerns	No information provided on the methods used to conduct the eye examination. Masking of outcome assessors was not reported.	Some concerns	No pre‐registered method (registry or protocol) available.	High risk of bias	The study is judged as some concerns in four out of five domains for this result. We therefore judged that the study was at an overall high risk of bias.
Lam 2020	Some concerns	Group allocation using random software sequence generated from Excel. Insufficient information to evaluate allocation concealment. There were no baseline imbalances that would suggest a problem with randomisation.	Low risk of bias	Everyone masked to group assignment until after study completion. Data analysed based on ITT principles.	Low risk of bias	Data was available for approx 87% of participants. No analysis performed to correct for bias due to missing data. Missing data occurred for documented reasons that were unrelated to the study outcome.	Low risk of bias	Appropriate and comparable methods of measuring outcome data were used.	Some concerns	Trial registry entry available but no pre‐specified information on the statistical analysis plan. Outcomes included as pre‐specified.	Some concerns	The study is judged to raise some concerns in at least one domain for this result, but not to be at high risk of bias for any domain.
Lu 2015	Some concerns	Participants were randomly divided into two groups. No information was provided relating to method of allocation sequence, allocation sequence concealment and baseline differences between intervention groups.	Some concerns	It was not possible to mask the participants or investigating clinicians. Deviations from intended interventions are not reported and the analysis was appropriate.	Low risk of bias	Data available for all participants.	Some concerns	No information provided on the methods used to conduct the eye examination. Masking of outcome assessors was not reported.	Some concerns	No pre‐registered method (registry or protocol) available.	High risk of bias	The study is judged as some concerns in four out of five domains for this result. We therefore judged that the study was at an overall high risk of bias.
Sankaridurg 2010	Low risk of bias	Randomisation was performed using website randomisation. Allocation concealment was maintained until after enrollment and participants assigned. No information on baseline differences between intervention groups was reported.	Low risk of bias	Participants, carers and those delivering the intervention were unaware of intervention received. Results analysed using mITT principles	Low risk of bias	Data available for approx 95% of participants.	Low risk of bias	Appropriate methods of measuring outcome data were used. Same study protocol for all participants and the team were blinded to the group allocation of patients.	Some concerns	No pre‐registered trail registry record or protocol available.	Some concerns	The study is judged to raise some concerns in at least one domain for this result, but not to be at high risk of bias for any domain.
**Subgroup 3.1.2 At 2 years**												
Hasebe 2014	Low risk of bias	Randomisation was performed using a lens allocation table designed on Microsoft Excel. Allocation concealment was maintained until after enrollment and participants assigned. There were no baseline imbalances that would suggest a problem with randomisation.	Low risk of bias	Participants, carers and those delivering the intervention were unaware of intervention received. Results analysed using ITT principles	High risk of bias	Outcome data were not available for all, or nearly all, randomized participants, and there is no evidence that the result was not biased by missing outcome data, and no information on whether missingness in the outcome could depend on its true value.	Low risk of bias	Appropriate and comparable methods of measuring outcome data were used. Outcome assessors may have been aware of the group allocation, but bias is minimised by using objective measurements. Therefore, it is unlikely that the outcome was influenced by knowledge of the intervention received.	Some concerns	Protocol registered. Not enough information in the protocol on a statistical plan and no published statistical plan found.	High risk of bias	The study is judged to be at high risk of bias in at least one domain for this result.
Lam 2020	Some concerns	Group allocation using random software sequence generated from Excel. Insufficient information to evaluate allocation concealment. There were no baseline imbalances that would suggest a problem with randomisation.	Low risk of bias	Everyone masked to group assignment until after study completion. Data analysed based on ITT principles.	Low risk of bias	Data was available for approx 87% of participants. No analysis performed to correct for bias due to missing data. Missing data occurred for documented reasons that were unrelated to the study outcome.	Low risk of bias	Appropriate and comparable methods of measuring outcome data were used.	Some concerns	Trial registry entry available but no pre‐specified information on the statistical analysis plan. Outcomes included as pre‐specified.	Some concerns	The study is judged to raise some concerns in at least one domain for this result, but not to be at high risk of bias for any domain.

**Table:** Risk of bias for analysis 3.2 Change in axial length from baseline

**Study**	**Bias**											
	**Randomisation process**		**Deviations from intended interventions**		**Missing outcome data**		**Measurement of the outcome**		**Selection of the reported results**		**Overall**	
	**Authors' judgement**	**Support for judgement**	**Authors' judgement**	**Support for judgement**	**Authors' judgement**	**Support for judgement**	**Authors' judgement**	**Support for judgement**	**Authors' judgement**	**Support for judgement**	**Authors' judgement**	**Support for judgement**
**Subgroup 3.2.1 At 1 year**												
Bao 2021	Some concerns	Adaptive randomisation with online software for sequence generation. No information relating to whether allocation was concealed until participants were enrolled and assigned to interventions. Observed imbalances of higher proportion of girls in one group and shorter AL could have occurred by chance.	Low risk of bias	Spectacles were not labelled to identify the treatment group assigned. Losses to follow up documented and no deviations due to trial context. Modified ITT analysis used to estimate the effect of assignment to intervention.	Low risk of bias	Data available for approx 95% of participants.	Low risk of bias	Methods of outcome measurement were appropriate. Comparable methods of outcome assessment were used. Assessors were masked to the intervention status.	Some concerns	Primary outcomes analysed as pre‐specified in trial registry. No information to judge whether this result was analysed in accordance with a pre‐specified analysis plan	Some concerns	The study is judged to raise some concerns in at least one domain for this result, but not to be at high risk of bias for any domain.
Lam 2020	Some concerns	Group allocation using random software sequence generated from Excel. Insufficient information to evaluate allocation concealment. There were no baseline imbalances that would suggest a problem with randomisation.	Low risk of bias	Everyone masked to group assignment until after study completion. Data analysed based on Intention to Treat principles.	Low risk of bias	Data was available for approx 87% of participants. No analysis performed to correct for bias due to missing data. Missing data occurred for documented reasons that were unrelated to the study outcome.	Low risk of bias	Appropriate and comparable methods of measuring outcome data were used. Examiners masked to refractive status at enrolment and the group allocation of participants.	Some concerns	Trial registry entry available, not analysis protocol. Outcomes included as pre‐specified.	Some concerns	The study is judged to raise some concerns in at least one domain for this result, but not to be at high risk of bias for any domain.
Sankaridurg 2010	Low risk of bias	Randomisation was performed using website randomisation. Allocation concealment was maintained until after enrollment and participants assigned. No information on baseline differences between intervention groups was reported.	Low risk of bias	Participants, carers and those delivering the intervention were unaware of intervention received. Results analysed using ITT principles.	Low risk of bias	Data available for approx 95% of participants.	Low risk of bias	Appropriate methods of measuring outcome data were used. Same study protocol for all participants and the team were masked to the group allocation of patients.	Some concerns	No pre‐registered trial registry record or protocol available.	Some concerns	The study is judged to raise some concerns in at least one domain for this result, but not to be at high risk of bias for any domain.
**Subgroup 3.2.2 At 2 years**												
Hasebe 2014	Low risk of bias	Randomisation was performed using a lens allocation table designed on Microsoft Excel. Allocation concealment was maintained until after enrollment and participants assigned. There were no baseline imbalances that would suggest a problem with randomisation.	Low risk of bias	Participants, carers and those delivering the intervention were unaware of intervention received. Results analysed using mITT principles.	High risk of bias	Outcome data were not available for all, or nearly all, randomized participants, and there is no evidence that the result was not biased by missing outcome data, and no information on whether missingness in the outcome could depend on its true value.	Low risk of bias	Appropriate and comparable methods of measuring outcome data were used. Outcome assessors may have been aware of the group allocation, but bias is minimised by using objective measurements. Therefore, it is unlikely that the outcome was influenced by knowledge of the intervention received.	Some concerns	Protocol registered. Not enough information in the protocol on a statistical plan and no published statistical plan found.	High risk of bias	The study is judged to be at high risk of bias in at least one domain for this result.
Lam 2020	Some concerns	Group allocation using random software sequence generated from Excel. Insufficient information to evaluate allocation concealment. There were no baseline imbalances that would suggest a problem with randomisation.	Low risk of bias	Everyone masked to group assignment until after study completion. Data analysed based on Intention to Treat principles.	Low risk of bias	Data was available for approx 87% of participants. No analysis performed to correct for bias due to missing data. Missing data occurred for documented reasons that were unrelated to the study outcome.	Low risk of bias	Appropriate and comparable methods of measuring outcome data were used. Examiners masked to refractive status at enrolment and the group allocation of participants.	Some concerns	Trial registry entry available, not analysis protocol. Outcomes included as pre‐specified.	Some concerns	The study is judged to raise some concerns in at least one domain for this result, but not to be at high risk of bias for any domain.

**Table:** Risk of bias for analysis 4.1 Change in refractive error from baseline

**Study**	**Bias**											
	**Randomisation process**		**Deviations from intended interventions**		**Missing outcome data**		**Measurement of the outcome**		**Selection of the reported results**		**Overall**	
	**Authors' judgement**	**Support for judgement**	**Authors' judgement**	**Support for judgement**	**Authors' judgement**	**Support for judgement**	**Authors' judgement**	**Support for judgement**	**Authors' judgement**	**Support for judgement**	**Authors' judgement**	**Support for judgement**
**Subgroup 4.1.1 At 1 year**												
Anstice 2011	Some concerns	Randomisation was performed using the permuted block design. Allocation concealment was not reported. There were no baseline imbalances that would suggest a problem with randomisation.	Some concerns	It was not possible to mask the participants or investigators providing clinical care given the nature of the interventions but investigating optometrists were masked from previous results. Results analysed using ITT principles without imputation of missing data.	Low risk of bias	Outcome data available for 88% of randomised participants. No analysis to correct for bias due to missing data. Missingness balanced across arms.	Low risk of bias	Appropriate methods of measuring outcome data were used. Same study protocol for all participants and the team were masked to the group allocation of patients.	Some concerns	Protocol registered. Insufficient information in the protocol on a statistical plan and no published statistical plan found.	Some concerns	The study is judged to raise some concerns in at least one domain for this result, but not to be at high risk of bias for any domain.
BLINK Study 2020	Low risk of bias	Central registration system, permuted block method. Everyone masked to CL assignment until after study completion. There were no baseline imbalances that would suggest a problem with randomisation.	Low risk of bias	Everyone masked to group assignment until after study completion. Data analysed based on ITT principles.	Low risk of bias	Outcome data were available for nearly all participants (97.5%).	Low risk of bias	Methods of outcome measurement were appropriate and comparable. The team were masked to the group allocation of patients.	Low risk of bias	Protocol registered, with a pre published protocol and statistical plan.	Low risk of bias	The study is judged to be at low risk of bias in all domains.
Chamberlain 2019	Some concerns	Contract organisation used a a random number ‐ generating computer program. Investigators had access to randomisation codes. No statistically significant differences between the experimental and control groups.	Low risk of bias	Participants and their parents were masked. Modified ITT analysis.	Low risk of bias	Outcome data available for 75% of randomised participants. No analysis to correct for bias due to missing data. Missingness balanced across arms.	Low risk of bias	Appropriate and comparable methods of outcome measurement. Outcome assessors masked to intervention received.	Low risk of bias	Prospective registration. Data analysed according to a pre‐specified analysis plan. Reported outcomes and data analysis pre‐specified.	Some concerns	The study is judged to raise some concerns in at least one domain for this result, but not to be at high risk of bias for any domain.
CONTROL Study 2016	Low risk of bias	Randomisation was performed by an off site clinical coordinator. Allocation concealment maintained until the end of the study. There were no baseline imbalances that would suggest a problem with randomisation.	Some concerns	Participants, carers and those delivering the intervention were unaware of intervention received. Participants for whom only baseline data were available were excluded from the analysis.	Low risk of bias	Outcome data available for 90.7% of randomised participants. Missingness balanced across arms.	Low risk of bias	Appropriate methods of measuring outcome data were used. Same study protocol for all participants and the team weremasked to the group allocation of patients.	Some concerns	Protocol registered. Not enough information in the protocol on a statistical plan and no published statistical plan found.	Some concerns	The study is judged to raise some concerns in at least one domain for this result, but not to be at high risk of bias for any domain.
DISC Study 2011	Some concerns	Randomisation was performed using random software sequence in excel. There was no information relating to allocation concealment. There were no baseline imbalances that would suggest a problem with randomisation.	Low risk of bias	It was not possible to mask the participants due to functional differences in interventions being studied. Investigator providing clinical care and obtaining measurements were masked to group allocation. There were no deviations from intended interventions. Analysis were performed on the ITT principles.	Some concerns	Outcome data available for 57.9% of children that were randomised. Analytical methods to correct for bias were not performed. Posspible that missingness could depend on its true value but missing data balanced across the two groups with similar reasons.	Low risk of bias	Appropriate methods of measuring outcome data were used. Same study protocol for all participants and the team were masked to the group allocation of patients.	Some concerns	Protocol registered. Not enough information in the protocol on a statistical plan and no published statistical plan found.	Some concerns	The study is judged to raise some concerns in at least one domain for this result, but not to be at high risk of bias for any domain.
Garcia‐del Valle 2021	Some concerns	There was no information on method of randomisation, there was no baseline imbalance that would suggest a problem with randomisation.	Low risk of bias	Both participants and those delivering the intervention were unaware of intervention received. There were no deviations from intended interventions and the analysis was appropriate.	Some concerns	Outcome data available for 82% of children that were randomised. Analytical methods to correct for bias were not performed. Posspible that missingness could depend on its true value but missing data balanced across the two groups with similar reasons.	Low risk of bias	Methods of outcome measurement were appropriate and comparable. The team were masked to the group allocation of patients.	Some concerns	No pre‐registered method (registry or protocol) available.	Some concerns	The study is judged to raise some concerns in at least one domain for this result, but not to be at high risk of bias for any domain.
Ruiz‐Pomeda 2018	Low risk of bias	Central registration system, permuted block method. Everyone masked to CL assignment until after study completion. There were no baseline imbalances that would suggest a problem with randomisation.	Low risk of bias	It was not possible to mask the children or carers given the nature of the interventions. Results analysed using ITT principles	Low risk of bias	Outcome data were available for nearly all randomized participants.	Low risk of bias	Appropriate and comparable methods of measuring outcome data were used	Some concerns	Protocol registered. Not enough information in the protocol on a statistical plan and no published statistical plan found.	Some concerns	The study is judged to raise some concerns in at least one domain for this result, but not to be at high risk of bias for any domain.
Sankaridurg 2019	Some concerns	Permuted block randomisation with online software for sequence generation. Insufficient information to evaluate allocation concealment. There were no baseline imbalances that would suggest a problem with randomisation.	Low risk of bias	Everyone masked to group assignment until after study completion. Data analysed based on ITT principles.	Some concerns	Data was available for 61.4% of participants at 1 year and 46% at 2 years. No analysis performed to correct for bias due to missing data.	Low risk of bias	Methods of outcome measurement were appropriate and comparable. The team were masked to the group allocation of patients.	Some concerns	Protocol registered. Not enough information in the protocol on a statistical plan and no published statistical plan found.	Some concerns	The study is judged to raise some concerns in at least one domain for this result, but not to be at high risk of bias for any domain.
**Subgroup 4.1.2 At 2 years**												
BLINK Study 2020	Low risk of bias	Central registration system, permuted block method. Everyone masked to CL assignment until after study completion. There were no baseline imbalances that would suggest a problem with randomisation.	Low risk of bias	Everyone masked to group assignment until after study completion. Data analysed based on ITT principles.	Low risk of bias	Outcome data were available for nearly all participants (97.5%).	Low risk of bias	Methods of outcome measurement were appropriate and comparable. The team were masked to the group allocation of patients.	Low risk of bias	Protocol registered, with a pre published protocol and statistical plan.	Low risk of bias	The study is judged to be at low risk of bias in all domains.
Chamberlain 2019	Some concerns	Contract organisation used a a random number ‐ generating computer program. Investigators had access to randomisation codes. No statistically significant differences between the experimental and control groups.	Low risk of bias	Participants and their parents were masked. Modified ITT analysis.	Low risk of bias	Outcome data available for 75% of randomised participants. No analysis to correct for bias due to missing data. Missingness balanced across arms.	Low risk of bias	Appropriate and comparable methods of outcome measurement. Outcome assessors masked to intervention received.	Low risk of bias	Prospective registration. Data analysed according to a pre‐specified analysis plan. Reported outcomes and data analysis pre‐specified.	Some concerns	The study is judged to raise some concerns in at least one domain for this result, but not to be at high risk of bias for any domain.
DISC Study 2011	Some concerns	Randomisation was performed using random software sequence in excel. There was no information relating to allocation concealment. There were no baseline imbalances that would suggest a problem with randomisation.	Low risk of bias	It was not possible to mask the participants due to functional differences in interventions being studied. Investigator providing clinical care and obtaining measurements were masked to group allocation. There were no deviations from intended interventions. Analysis were performed on the ITT principles.	Some concerns	Outcome data available for 57.9% of children that were randomised. Analytical methods to correct for bias were not performed. Posspible that missingness could depend on its true value but missing data balanced across the two groups with similar reasons.	Low risk of bias	Appropriate methods of measuring outcome data were used. Same study protocol for all participants and the team were masked to the group allocation of patients.	Some concerns	Protocol registered. Not enough information in the protocol on a statistical plan and no published statistical plan found.	Some concerns	The study is judged to raise some concerns in at least one domain for this result, but not to be at high risk of bias for any domain.
Ruiz‐Pomeda 2018	Low risk of bias	Central registration system, permuted block method. Everyone masked to CL assignment until after study completion. There were no baseline imbalances that would suggest a problem with randomisation.	Low risk of bias	It was not possible to mask the children or carers given the nature of the interventions. Results analysed using ITT principles	Low risk of bias	Outcome data were available for nearly all randomized participants.	Low risk of bias	Appropriate and comparable methods of measuring outcome data were used	Some concerns	Protocol registered. Not enough information in the protocol on a statistical plan and no published statistical plan found.	Some concerns	The study is judged to raise some concerns in at least one domain for this result, but not to be at high risk of bias for any domain.
Sankaridurg 2019	Some concerns	Permuted block randomisation with online software for sequence generation. Insufficient information to evaluate allocation concealment. There were no baseline imbalances that would suggest a problem with randomisation.	Low risk of bias	Everyone masked to group assignment until after study completion. Data analysed based on ITT principles.	Some concerns	Data was available for 61.4% of participants at 1 year and 46% at 2 years. No analysis performed to correct for bias due to missing data.	Low risk of bias	Methods of outcome measurement were appropriate and comparable. The team were masked to the group allocation of patients.	Some concerns	Protocol registered. Not enough information in the protocol on a statistical plan and no published statistical plan found.	Some concerns	The study is judged to raise some concerns in at least one domain for this result, but not to be at high risk of bias for any domain.
**Subgroup 4.1.3 At 3 years**												
BLINK Study 2020	Low risk of bias	Central registration system, permuted block method. Everyone masked to CL assignment until after study completion. There were no baseline imbalances that would suggest a problem with randomisation.	Low risk of bias	Everyone masked to group assignment until after study completion. Data analysed based on ITT principles.	Low risk of bias	Outcome data were available for nearly all participants (97.5%).	Low risk of bias	Methods of outcome measurement were appropriate and comparable. The team were masked to the group allocation of patients.	Low risk of bias	Protocol registered, with a pre published protocol and statistical plan.	Low risk of bias	The study is judged to be at low risk of bias in all domains.
Chamberlain 2019	Some concerns	Contract organisation used a a random number ‐ generating computer program. Investigators had access to randomisation codes. No statistically significant differences between the experimental and control groups.	Low risk of bias	Participants and their parents were masked. Modified ITT analysis.	Low risk of bias	Outcome data available for 75% of randomised participants. No analysis to correct for bias due to missing data. Missingness balanced across arms.	Low risk of bias	Appropriate and comparable methods of outcome measurement. Outcome assessors masked to intervention received.	Low risk of bias	Prospective registration. Data analysed according to a pre‐specified analysis plan. Reported outcomes and data analysis pre‐specified.	Some concerns	The study is judged to raise some concerns in at least one domain for this result, but not to be at high risk of bias for any domain.

**Table:** Risk of bias for analysis 4.2 Change in axial length from baseline

**Study**	**Bias**											
	**Randomisation process**		**Deviations from intended interventions**		**Missing outcome data**		**Measurement of the outcome**		**Selection of the reported results**		**Overall**	
	**Authors' judgement**	**Support for judgement**	**Authors' judgement**	**Support for judgement**	**Authors' judgement**	**Support for judgement**	**Authors' judgement**	**Support for judgement**	**Authors' judgement**	**Support for judgement**	**Authors' judgement**	**Support for judgement**
**Subgroup 4.2.1 At 1 year**												
Anstice 2011	Some concerns	Randomisation was performed using the permuted block design. Allocation concealment was not reported. There were no baseline imbalances that would suggest a problem with randomisation.	Some concerns	It was not possible to mask the participants or investigators providing clinical care given the nature of the interventions but investigating optometrists were masked from previous results. Results analysed using ITT principles without imputation of missing data.	Low risk of bias	Outcome data available for 88% of randomised participants. No analysis to correct for bias due to missing data. Missingness balanced across arms.	Low risk of bias	Appropriate methods of measuring outcome data were used. Same study protocol for all participants and the team were masked to the group allocation of patients.	Some concerns	Protocol registered. Insufficient information in the protocol on a statistical plan and no published statistical plan found.	Some concerns	The study is judged to raise some concerns in at least one domain for this result, but not to be at high risk of bias for any domain.
BLINK Study 2020	Low risk of bias	Central registration system, permuted block method. Everyone masked to CL assignment until after study completion. There were no baseline imbalances that would suggest a problem with randomisation.	Low risk of bias	Everyone masked to group assignment until after study completion. Data analysed based on ITT principles.	Low risk of bias	Outcome data were available for nearly all participants (97.5%).	Low risk of bias	Methods of outcome measurement were appropriate and comparable. The team were masked to the group allocation of patients.	Low risk of bias	Protocol registered, with a pre published protocol and statistical plan.	Low risk of bias	The study is judged to be at low risk of bias in all domains.
Chamberlain 2019	Some concerns	Contract organisation used a a random number ‐ generating computer program. Investigators had access to randomisation codes. No statistically significant differences between the experimental and control groups.	Low risk of bias	Participants and their parents were masked. Modified ITT analysis.	Low risk of bias	Outcome data available for 75% of randomised participants. No analysis to correct for bias due to missing data. Missingness balanced across arms.	Low risk of bias	Appropriate and comparable methods of outcome measurement. Outcome assessors masked to intervention received.	Low risk of bias	Prospective registration. Data analysed according to a pre‐specified analysis plan. Reported outcomes and data analysis pre‐specified.	Some concerns	The study is judged to raise some concerns in at least one domain for this result, but not to be at high risk of bias for any domain.
CONTROL Study 2016	Low risk of bias	Randomisation was performed by an off site clinical coordinator. Allocation concealment maintained until the end of the study. There were no baseline imbalances that would suggest a problem with randomisation.	Some concerns	Participants, carers and those delivering the intervention were unaware of intervention received. Participants for whom only baseline data were available were excluded from the analysis.	Low risk of bias	Outcome data available for 90.7% of randomised participants. Missingness balanced across arms.	Low risk of bias	Appropriate methods of measuring outcome data were used. Same study protocol for all participants and the team were masked to the group allocation of patients.	Some concerns	Protocol registered. Not enough information in the protocol on a statistical plan and no published statistical plan found.	Some concerns	The study is judged to raise some concerns in at least one domain for this result, but not to be at high risk of bias for any domain.
DISC Study 2011	Some concerns	Randomisation was performed using random software sequence in excel. There was no information relating to allocation concealment. There were no baseline imbalances that would suggest a problem with randomisation.	Low risk of bias	It was not possible to mask the participants due to functional differences in interventions being studied. Investigator providing clinical care and obtaining measurements were masked to group allocation. There were no deviations from intended interventions. Analysis were performed on the ITT principles.	Some concerns	Outcome data available for 57.9% of children that were randomised. Analytical methods to correct for bias were not performed. Posspible that missingness could depend on its true value but missing data balanced across the two groups with similar reasons.	Low risk of bias	Appropriate methods of measuring outcome data were used. Same study protocol for all participants and the team were masked to the group allocation of patients.	Some concerns	Protocol registered. Not enough information in the protocol on a statistical plan and no published statistical plan found.	Some concerns	The study is judged to raise some concerns in at least one domain for this result, but not to be at high risk of bias for any domain.
Garcia‐del Valle 2021	Some concerns	There was no information on method of randomisation, there was no baseline imbalance that would suggest a problem with randomisation.	Low risk of bias	Both participants and those delivering the intervention were unaware of intervention received. There were no deviations from intended interventions and the analysis was appropriate.	Some concerns	Outcome data available for 82% of children that were randomised. Analytical methods to correct for bias were not performed. It is possible that misssingness could depend on its true value but missingness balanced across the two groups with similar reasons.	Low risk of bias	Methods of outcome measurement were appropriate and comparable. The team were blined to the group allocation of patients.	Some concerns	No pre‐registered method (registry or protocol) available.	Some concerns	The study is judged to raise some concerns in at least one domain for this result, but not to be at high risk of bias for any domain.
Ruiz‐Pomeda 2018	Low risk of bias	Central registration system, permuted block method. Everyone masked to CL assignment until after study completion. There were no baseline imbalances that would suggest a problem with randomisation.	Low risk of bias	It was not possible to mask the children or carers given the nature of the interventions. Results analysed using ITT principles	Low risk of bias	Outcome data were available for nearly all randomized participants.	Low risk of bias	Appropriate and comparable methods of measuring outcome data were used.	Some concerns	Protocol registered. Not enough information in the protocol on a statistical plan and no published statistical plan found.	Some concerns	The study is judged to raise some concerns in at least one domain for this result, but not to be at high risk of bias for any domain.
Sankaridurg 2019	Some concerns	Permuted block randomisation with online software for sequence generation. Insufficient information to evaluate allocation concealment. There were no baseline imbalances that would suggest a problem with randomisation.	Low risk of bias	Everyone masked to group assignment until after study completion. Data analysed based on ITT principles.	Some concerns	Data was available for 61.4% of participants at 1 year and 46% at 2 years. No analysis performed to correct for bias due to missing data.	Low risk of bias	Methods of outcome measurement were appropriate and comparable. The team were masked to the group allocation of patients.	Some concerns	Protocol registered. Not enough information in the protocol on a statistical plan and no published statistical plan found.	Some concerns	The study is judged to raise some concerns in at least one domain for this result, but not to be at high risk of bias for any domain.
**Subgroup 4.2.2 At 2 years**												
BLINK Study 2020	Low risk of bias	Central registration system, permuted block method. Everyone masked to CL assignment until after study completion. There were no baseline imbalances that would suggest a problem with randomisation.	Low risk of bias	Everyone masked to group assignment until after study completion. Data analysed based on ITT principles.	Low risk of bias	Outcome data were available for nearly all participants (97.5%).	Low risk of bias	Methods of outcome measurement were appropriate and comparable. The team were masked to the group allocation of patients.	Low risk of bias	Protocol registered, with a pre published protocol and statistical plan.	Low risk of bias	The study is judged to be at low risk of bias in all domains.
Chamberlain 2019	Some concerns	Contract organisation used a a random number ‐ generating computer program. Investigators had access to randomisation codes. No statistically significant differences between the experimental and control groups.	Low risk of bias	Participants and their parents were masked. Modified ITT analysis.	Low risk of bias	Outcome data available for 75% of randomised participants. No analysis to correct for bias due to missing data. Missingness balanced across arms.	Low risk of bias	Appropriate and comparable methods of outcome measurement. Outcome assessors masked to intervention received.	Low risk of bias	Prospective registration. Data analysed according to a pre‐specified analysis plan. Reported outcomes and data analysis pre‐specified.	Some concerns	The study is judged to raise some concerns in at least one domain for this result, but not to be at high risk of bias for any domain.
DISC Study 2011	Some concerns	Randomisation was performed using random software sequence in excel. There was no information relating to allocation concealment. There were no baseline imbalances that would suggest a problem with randomisation.	Low risk of bias	It was not possible to mask the participants due to functional differences in interventions being studied. Investigator providing clinical care and obtaining measurements were masked to group allocation. There were no deviations from intended interventions. Analysis were performed on the ITT principles.	Some concerns	Outcome data available for 57.9% of children that were randomised. Analytical methods to correct for bias were not performed. Posspible that missingness could depend on its true value but missing data balanced across the two groups with similar reasons.	Low risk of bias	Appropriate methods of measuring outcome data were used. Same study protocol for all participants and the team were masked to the group allocation of patients.	Some concerns	Protocol registered. Not enough information in the protocol on a statistical plan and no published statistical plan found.	Some concerns	The study is judged to raise some concerns in at least one domain for this result, but not to be at high risk of bias for any domain.
Ruiz‐Pomeda 2018	Low risk of bias	Central registration system, permuted block method. Everyone masked to CL assignment until after study completion. There were no baseline imbalances that would suggest a problem with randomisation.	Low risk of bias	It was not possible to mask the children or carers given the nature of the interventions. Results analysed using ITT principles	Low risk of bias	Outcome data were available for nearly all randomized participants.	Low risk of bias	Appropriate and comparable methods of measuring outcome data were used.	Some concerns	Protocol registered. Not enough information in the protocol on a statistical plan and no published statistical plan found.	Some concerns	The study is judged to raise some concerns in at least one domain for this result, but not to be at high risk of bias for any domain.
Sankaridurg 2019	Some concerns	Permuted block randomisation with online software for sequence generation. Insufficient information to evaluate allocation concealment. There were no baseline imbalances that would suggest a problem with randomisation.	Low risk of bias	Everyone masked to group assignment until after study completion. Data analysed based on ITT principles.	Some concerns	Data was available for 61.4% of participants at 1 year and 46% at 2 years. No analysis performed to correct for bias due to missing data.	Low risk of bias	Methods of outcome measurement were appropriate and comparable. The team were masked to the group allocation of patients.	Some concerns	Protocol registered. Not enough information in the protocol on a statistical plan and no published statistical plan found.	Some concerns	The study is judged to raise some concerns in at least one domain for this result, but not to be at high risk of bias for any domain.
**Subgroup 4.2.3 At 3 years**												
BLINK Study 2020	Low risk of bias	Central registration system, permuted block method. Everyone masked to CL assignment until after study completion. There were no baseline imbalances that would suggest a problem with randomisation.	Low risk of bias	Everyone masked to group assignment until after study completion. Data analysed based on ITT principles.	Low risk of bias	Outcome data were available for nearly all participants (97.5%).	Low risk of bias	Methods of outcome measurement were appropriate and comparable. The team were masked to the group allocation of patients.	Low risk of bias	Protocol registered, with a pre published protocol and statistical plan.	Low risk of bias	The study is judged to be at low risk of bias in all domains.
Chamberlain 2019	Some concerns	Contract organisation used a a random number ‐ generating computer program. Investigators had access to randomisation codes. No statistically significant differences between the experimental and control groups.	Low risk of bias	Participants and their parents were masked. Modified ITT analysis.	Low risk of bias	Outcome data available for 75% of randomised participants. No analysis to correct for bias due to missing data. Missingness balanced across arms.	Low risk of bias	Appropriate and comparable methods of outcome measurement. Outcome assessors masked to intervention received.	Low risk of bias	Prospective registration. Data analysed according to a pre‐specified analysis plan. Reported outcomes and data analysis pre‐specified.	Some concerns	The study is judged to raise some concerns in at least one domain for this result, but not to be at high risk of bias for any domain.

**Table:** Risk of bias for analysis 4.3 Change in refractive error following cessation of treatment (1 year)

**Study**	**Bias**											
	**Randomisation process**		**Deviations from intended interventions**		**Missing outcome data**		**Measurement of the outcome**		**Selection of the reported results**		**Overall**	
	**Authors' judgement**	**Support for judgement**	**Authors' judgement**	**Support for judgement**	**Authors' judgement**	**Support for judgement**	**Authors' judgement**	**Support for judgement**	**Authors' judgement**	**Support for judgement**	**Authors' judgement**	**Support for judgement**
Ruiz‐Pomeda 2018	Low risk of bias	Central registration system, permuted block method. Everyone masked to CL assignment until after study completion. There were no baseline imbalances that would suggest a problem with randomisation.	Low risk of bias	It was not possible to mask the children or carers given the nature of the interventions. The differences at baseline were subjected to analysis of variance.	Low risk of bias	Outcome data were available for nearly all randomised participants.	Low risk of bias	Appropriate and comparable methods of measuring outcome data were used.	Some concerns	Protocol registered. Not enough information in the protocol on a statistical plan and no published statistical plan used.	Some concerns	The study is judged to raise some concerns in at least one domain for this result, but not at high risk of bias for any domain.

**Table:** Risk of bias for analysis 4.4 Change in axial length following cessation of treatment (1 year)

**Study**	**Bias**											
	**Randomisation process**		**Deviations from intended interventions**		**Missing outcome data**		**Measurement of the outcome**		**Selection of the reported results**		**Overall**	
	**Authors' judgement**	**Support for judgement**	**Authors' judgement**	**Support for judgement**	**Authors' judgement**	**Support for judgement**	**Authors' judgement**	**Support for judgement**	**Authors' judgement**	**Support for judgement**	**Authors' judgement**	**Support for judgement**
Ruiz‐Pomeda 2018	Low risk of bias	Central registration system, permuted block method. Everyone masked to CL assignment until after study completion. There were no baseline imbalances that would suggest a problem with randomisation.	Low risk of bias	It was not possible to mask the children or carers given the nature of the interventions. The differences at baseline were subjected to analysis of variance.	Low risk of bias	Outcome data were available for nearly all randomised participants.	Low risk of bias	Appropriate and comparable methods of measuring outcome data were used.	Some concerns	Protocol registered. Not enough information in the protocol on a statistical plan and no published statistical plan used.	Some concerns	The study is judged to raise some concerns in at least one domain for this result, but not at high risk of bias for any domain.

**Table:** Risk of bias for analysis 5.1 Change in refractive error from baseline

**Study**	**Bias**											
	**Randomisation process**		**Deviations from intended interventions**		**Missing outcome data**		**Measurement of the outcome**		**Selection of the reported results**		**Overall**	
	**Authors' judgement**	**Support for judgement**	**Authors' judgement**	**Support for judgement**	**Authors' judgement**	**Support for judgement**	**Authors' judgement**	**Support for judgement**	**Authors' judgement**	**Support for judgement**	**Authors' judgement**	**Support for judgement**
**Subgroup 5.1.1 At 1 year**												
CLAMP Study 2004	Some concerns	Randomisation was performed using stratification and block allocation. There was no information relating to allocation concealment. There were no baseline imbalances that would suggest a problem with randomisation.	Some concerns	It was not possible to mask the participants due to functional differences in interventions being studied. Investigator providing clinical care and obtaining measurements were masked to group allocation. No information on deviation from intended outcomes. Data were analysed using ITT methods.	Low risk of bias	Data available for approx 97% of participants.	Low risk of bias	Appropriate methods of measuring outcome data were used. Same study protocol for all participants and the team were masked to the group allocation of patients.	Some concerns	The outcome measurements were taken in accordance with the clinical trials registry record. No statistical analysis plan (SAP) was reported.	Some concerns	The study is judged to raise some concerns in at least one domain for this result, but not to be at high risk of bias for any domain.
Katz 2003	Some concerns	Randomisation was performed using the permuted block method. Allocation concealment using sealed envelopes. Baseline differences were identified between groups.	Low risk of bias	Neither patients nor clinical observers were masked to treatment group. There were no deviations from intended interventions and the analysis was appropriate.	High risk of bias	Outcome data were not available for all, or nearly all, randomized participants, and there is no evidence that the result was not biased by missing outcome data, and no information on whether missingness in the outcome could depend on its true value.	Low risk of bias	Appropriate and comparable methods of measuring outcome data were used. Outcome assessors are likely to have been aware of the group allocation, but bias was minimised by using objective measurements. Therefore, it is unlikely that the outcome was influenced by knowledge of the intervention received.	Some concerns	No pre‐registered method (registry or protocol) available.	High risk of bias	The study is judged to be at high risk of bias in at least one domain for this result.
**Subgroup 5.1.2 At 2 years**												
CLAMP Study 2004	Some concerns	Randomisation was performed using stratification and block allocation. There was no information relating to allocation concealment. There were no baseline imbalances that would suggest a problem with randomisation.	Some concerns	It was not possible to mask the participants due to functional differences in interventions being studied. Investigator providing clinical care and obtaining measurements were masked to group allocation. No information on deviation from intended outcomes. Data were analysed using ITT methods.	Low risk of bias	Data available for approx 97% of participants.	Low risk of bias	Appropriate methods of measuring outcome data were used. Same study protocol for all participants and the team were masked to the group allocation of patients.	Some concerns	The outcome measurements were taken in accordance with the clinical trials registry record. No statistical analysis plan (SAP) was reported.	Some concerns	The study is judged to raise some concerns in at least one domain for this result, but not to be at high risk of bias for any domain.
Katz 2003	Some concerns	Randomisation was performed using the permuted block method. Allocation concealment using sealed envelopes. Baseline differences were identified between groups.	Low risk of bias	Neither patients nor clinical observers were masked to treatment group. There were no deviations from intended interventions and the analysis was appropriate.	High risk of bias	Outcome data were not available for all, or nearly all, randomized participants, and there is no evidence that the result was not biased by missing outcome data, and no information on whether missingness in the outcome could depend on its true value.	Low risk of bias	Appropriate and comparable methods of measuring outcome data were used. Outcome assessors are likely to have been aware of the group allocation, but bias was minimised by using objective measurements. Therefore, it is unlikely that the outcome was influenced by knowledge of the intervention received.	Some concerns	No pre‐registered method (registry or protocol) available.	High risk of bias	The study is judged to be at high risk of bias in at least one domain for this result.
**Subgroup 5.1.3 At 3 years**												
CLAMP Study 2004	Some concerns	Randomisation was performed using stratification and block allocation. There was no information relating to allocation concealment. There were no baseline imbalances that would suggest a problem with randomisation.	Some concerns	It was not possible to mask the participants due to functional differences in interventions being studied. Investigator providing clinical care and obtaining measurements were masked to group allocation. No information on deviation from intended outcomes. Data were analysed using ITT methods.	Low risk of bias	Data available for approx 97% of participants.	Low risk of bias	Appropriate methods of measuring outcome data were used. Same study protocol for all participants and the team were masked to the group allocation of patients.	Some concerns	The outcome measurements were taken in accordance with the clinical trials registry record. No statistical analysis plan (SAP) was reported.	Some concerns	The study is judged to raise some concerns in at least one domain for this result, but not to be at high risk of bias for any domain.

**Table:** Risk of bias for analysis 5.2 Change in axial length from baseline

**Study**	**Bias**											
	**Randomisation process**		**Deviations from intended interventions**		**Missing outcome data**		**Measurement of the outcome**		**Selection of the reported results**		**Overall**	
	**Authors' judgement**	**Support for judgement**	**Authors' judgement**	**Support for judgement**	**Authors' judgement**	**Support for judgement**	**Authors' judgement**	**Support for judgement**	**Authors' judgement**	**Support for judgement**	**Authors' judgement**	**Support for judgement**
**Subgroup 5.2.1 At 1 year**												
CLAMP Study 2004	Some concerns	Randomisation was performed using stratification and block allocation. There was no information relating to allocation concealment. There were no baseline imbalances that would suggest a problem with randomisation.	Some concerns	It was not possible to mask the participants due to functional differences in interventions being studied. Investigator providing clinical care and obtaining measurements were masked to group allocation. No information on deviation from intended outcomes. Data were analysed using ITT methods.	Low risk of bias	Data available for approx 97% of participants.	Low risk of bias	Appropriate methods of measuring outcome data were used. Same study protocol for all participants and the team were masked to the group allocation of patients.	Some concerns	The outcome measurements were taken in accordance with the clinical trials registry record. No statistical analysis plan (SAP) was reported.	Some concerns	The study is judged to raise some concerns in at least one domain for this result, but not to be at high risk of bias for any domain.
Katz 2003	Some concerns	Randomisation was performed using the permuted block method. Allocation concealment using sealed envelopes. Baseline differences were identified between groups.	Low risk of bias	Neither patients nor clinical observers were masked to treatment group. There were no deviations from intended interventions and the analysis was appropriate.	High risk of bias	Outcome data were not available for all, or nearly all, randomized participants, and there is no evidence that the result was not biased by missing outcome data, and no information on whether missingness in the outcome could depend on its true value.	Low risk of bias	Appropriate and comparable methods of measuring outcome data were used. Outcome assessors are likely to have been aware of the group allocation, but bias was minimised by using objective measurements. Therefore, it is unlikely that the outcome was influenced by knowledge of the intervention received.	Some concerns	No pre‐registered method (registry or protocol) available.	High risk of bias	The study is judged to be at high risk of bias in at least one domain for this result.
**Subgroup 5.2.2 At 2 years**												
CLAMP Study 2004	Some concerns	Randomisation was performed using stratification and block allocation. There was no information relating to allocation concealment. There were no baseline imbalances that would suggest a problem with randomisation.	Some concerns	It was not possible to mask the participants due to functional differences in interventions being studied. Investigator providing clinical care and obtaining measurements were masked to group allocation. No information on deviation from intended outcomes. Data were analysed using ITT methods.	Low risk of bias	Data available for approx 97% of participants.	Low risk of bias	Appropriate methods of measuring outcome data were used. Same study protocol for all participants and the team were masked to the group allocation of patients.	Some concerns	The outcome measurements were taken in accordance with the clinical trials registry record. No statistical analysis plan (SAP) was reported.	Some concerns	The study is judged to raise some concerns in at least one domain for this result, but not to be at high risk of bias for any domain.
Katz 2003	Some concerns	Randomisation was performed using the permuted block method. Allocation concealment using sealed envelopes. Baseline differences were identified between groups.	Low risk of bias	Neither patients nor clinical observers were masked to treatment group. There were no deviations from intended interventions and the analysis was appropriate.	High risk of bias	Outcome data were not available for all, or nearly all, randomized participants, and there is no evidence that the result was not biased by missing outcome data, and no information on whether missingness in the outcome could depend on its true value.	Low risk of bias	Appropriate and comparable methods of measuring outcome data were used. Outcome assessors are likely to have been aware of the group allocation, but bias was minimised by using objective measurements. Therefore, it is unlikely that the outcome was influenced by knowledge of the intervention received.	Some concerns	No pre‐registered method (registry or protocol) available.	High risk of bias	The study is judged to be at high risk of bias in at least one domain for this result.
**Subgroup 5.2.3 At 3 years**												
CLAMP Study 2004	Some concerns	Randomisation was performed using stratification and block allocation. There was no information relating to allocation concealment. There were no baseline imbalances that would suggest a problem with randomisation.	Some concerns	It was not possible to mask the participants due to functional differences in interventions being studied. Investigator providing clinical care and obtaining measurements were masked to group allocation. No information on deviation from intended outcomes. Data were analysed using ITT methods.	Low risk of bias	Data available for approx 97% of participants.	Low risk of bias	Appropriate methods of measuring outcome data were used. Same study protocol for all participants and the team were masked to the group allocation of patients.	Some concerns	The outcome measurements were taken in accordance with the clinical trials registry record. No statistical analysis plan (SAP) was reported.	Some concerns	The study is judged to raise some concerns in at least one domain for this result, but not to be at high risk of bias for any domain.

**Table:** Risk of bias for analysis 6.1 Change in axial length from baseline

**Study**	**Bias**											
	**Randomisation process**		**Deviations from intended interventions**		**Missing outcome data**		**Measurement of the outcome**		**Selection of the reported results**		**Overall**	
	**Authors' judgement**	**Support for judgement**	**Authors' judgement**	**Support for judgement**	**Authors' judgement**	**Support for judgement**	**Authors' judgement**	**Support for judgement**	**Authors' judgement**	**Support for judgement**	**Authors' judgement**	**Support for judgement**
**Subgroup 6.1.1 At 1 year**												
Bian 2020	Some concerns	Randomisation was performed using a random number table. There was no information relating to allocation concealment. There were no baseline imbalances that would suggest a problem with randomisation.	Some concerns	It was not possible to mask the participants due to functional differences in interventions being studied. Masking of investigators is not reported. No information on deviation from intended outcomes and appropriate analyses were conducted.	Low risk of bias	Outcome data were available for all randomized participants.	Low risk of bias	Appropriate and comparable methods of measuring outcome data were used. Outcome assessors may have been aware of the group allocation, but bias is minimised by using objective measurements. Therefore, it is unlikely that the outcome was influenced by knowledge of the intervention received.	Some concerns	No trial registry record or protocol available.	Some concerns	The study is judged to raise some concerns in at least one domain for this result, but not to be at high risk of bias for any domain.
Jakobsen 2022	Low risk of bias	Randomisation was performed by a computer‐generated allocation sequence. Allocation concealment using sealed envelopes. There were no baseline imbalance that would suggest a problem with randomisation.	Low risk of bias	It was not possible to mask the children or carers given the nature of the interventions. A greater number of dropouts in the intervention group mostly due to lack of motivation that could have happened outside the trial context. Analysis using ITT principles.	Low risk of bias	Data available for 63% of participants in the intervention group and 93% of those in the control group. Statistical analysis performed to adjust for baseline differences in outcomes. Missingness occurred for documented reasons that were unrelated to the outcome.	Low risk of bias	Appropriate and comparable methods of measuring outcome data were used. No information was available on masking of outcome assessors.Outcomes were measured objectively.	Some concerns	Trial registry entry available, not analysis protocol. Outcomes included as pre‐specified.	Some concerns	The study is judged to raise some concerns in at least one domain for this result, but not to be at high risk of bias for any domain.
Lyu 2020	Some concerns	Random group allocation though details not specified. No information on allocation concealment. No statistically significant baseline imbalances that would suggest a problem with randomisation.	High risk of bias	It was not possible to mask the children or carers given the nature of the interventions. Non‐compliant participants were excluded.	Low risk of bias	Reasons for exclusion stated but missing outcome data not available.	Low risk of bias	Appropriate and comparable methods of measuring outcome data were used. Outcome assessors are likely to have been aware of the group allocation.	Some concerns	No pre‐registered method (registry or protocol) available.	High risk of bias	The study is judged to be at high risk of bias in at least one domain for this result.
Ren 2017	Some concerns	Participants were randomised though no details of method of randomisation are recorded. There was no information relating to allocation concealment. There were no baseline imbalances that would suggest a problem with randomisation.	Some concerns	It was not possible to mask the participants due to functional differences in interventions being studied. Masking of the investigator is not reported. No information on deviation from intended outcomes and appropriate analysis were conducted.	Low risk of bias	Data available for all participants.	Some concerns	No information about the methods of measuring the outcome or masking of the assessors were reported.	Some concerns	No registry record or protocol available.	High risk of bias	The study is judged as some concerns in four out of five domains for this result. We therefore judged that the study was at an overall high risk of bias.
ROMIO Study 2012	High risk of bias	Randomisation was performed using commercial spreadsheet random number generator. Random allocation sequence was revealed to the unmasked examiner after eligibility of subjects was confirmed. There were no baseline imbalances that would suggest a problem with randomisation.	Some concerns	It was not possible to mask the participants, carers or investigating clinicians given the nature of the interventions. There were no deviations from intended interventions. Results were not analysed using ITT principles, although unlikely to have substantial impact.	High risk of bias	Outcome data were not available for all, or nearly all, randomised participants, and there is no evidence that the result was not biased by missing outcome data, and no information on whether missingness in the outcome could depend on its true value.	Low risk of bias	Appropriate and comparable methods of measuring outcome data were used. Outcome assessors are likely to have been aware of the group allocation, but bias was minimised by using objective measurements. Therefore, it is unlikely that the outcome was influenced by knowledge of the intervention received.	Some concerns	Protocol registered. Not enough information in the protocol on a statistical plan and no published statistical plan found.	High risk of bias	The study is judged to be at high risk of bias in at least one domain for this result.
Tang 2021	Some concerns	Participants were randomised though no details of method of randomisation are recorded. There was no information relating to allocation concealment. There were no baseline imbalances that would suggest a problem with randomisation.	Low risk of bias	It was not possible to mask the participants due to functional differences in interventions being studied. Masking of the investigator is not reported. No information was provided about masking investigators. No information on deviation from intended outcomes and appropriate analysis were conducted.	Low risk of bias	Outcome data available for 93.3% of randomised participants. No analysis to correct for bias due to missing data. Missingness balanced across arms.	Low risk of bias	Appropriate and comparable methods of measuring outcome data were used. Outcome assessors may have been aware of the group allocation, but bias is minimised by using objective measurements. Therefore, it is unlikely that the outcome was influenced by knowledge of the intervention received.	Some concerns	No registry record or protocol available.	Some concerns	The study is judged to raise some concerns in at least one domain for this result, but not to be at high risk of bias for any domain.
Zhang 2021	Low risk of bias	Randomisation was performed by sealed envelopes, with a computer‐generated allocation sequence. There were no baseline imbalance that would suggest a problem with randomisation.	Low risk of bias	Participants and their parents were masked. Analysis using ITT principles.	Low risk of bias	Outcome data were available for nearly all participants.	Low risk of bias	Appropriate and comparable methods of measuring outcome data were used.	Some concerns	Protocol registered. Not enough information in the protocol on a statistical plan and no published statistical plan found.	Some concerns	The study is judged to raise some concerns in at least one domain for this result, but not to be at high risk of bias for any domain.
**Subgroup 6.1.2 At 2 years**												
Charm 2013	Some concerns	Randomisation was performed using commercial spreadsheet random number generator. Method for allocation concealment unclear. There were no baseline imbalances that would suggest a problem with randomisation.	High risk of bias	Participants, carers and examiners were not masked to assigned intervention. Participants in the control group explored other myopia control interventions. ITT analysis aas not conducted.	Some concerns	Outcome data available for 53.8% of children that were randomised. Analytical methods to correct for bias were not performed. It is possible that misssingness could depend on its true value but missingness balanced across the two groups with similar reasons.	Low risk of bias	Appropriate methods of measuring outcome data were used. Same study protocol for all participants and the team were blinded to the group allocation of patients.	Some concerns	Protocol registered. Not enough information in the protocol on a statistical plan and no published statistical plan found.	High risk of bias	The study is judged to be at high risk of bias in at least one domain for this result.
ROMIO Study 2012	High risk of bias	Randomisation was performed using commercial spreadsheet random number generator. Random allocation sequence was revealed to the unmasked examiner after eligibility of subjects was confirmed. There were no baseline imbalances that would suggest a problem with randomisation.	Some concerns	It was not possible to mask the participants, carers or investigating clinicians given the nature of the interventions. There were no deviations from intended interventions. Results were not analysed using ITT principles, although unlikely to have substantial impact.	High risk of bias	Outcome data were not available for all, or nearly all, randomised participants, and there is no evidence that the result was not biased by missing outcome data, and no information on whether missingness in the outcome could depend on its true value.	Low risk of bias	Appropriate and comparable methods of measuring outcome data were used. Outcome assessors are likely to have been aware of the group allocation, but bias was minimised by using objective measurements. Therefore, it is unlikely that the outcome was influenced by knowledge of the intervention received.	Some concerns	Protocol registered. Not enough information in the protocol on a statistical plan and no published statistical plan found.	High risk of bias	The study is judged to be at high risk of bias in at least one domain for this result.

**Table:** Risk of bias for analysis 7.1 Change in refractive error from baseline (1 year)

**Study**	**Bias**											
	**Randomisation process**		**Deviations from intended interventions**		**Missing outcome data**		**Measurement of the outcome**		**Selection of the reported results**		**Overall**	
	**Authors' judgement**	**Support for judgement**	**Authors' judgement**	**Support for judgement**	**Authors' judgement**	**Support for judgement**	**Authors' judgement**	**Support for judgement**	**Authors' judgement**	**Support for judgement**	**Authors' judgement**	**Support for judgement**
**Subgroup 7.1.1 Atropine (high dose)**												
ATOM Study 2006	Low risk of bias	Randomisation was performed using a computer generated randomisation list. It is unclear how allocation concelment was conducted. There were no baseline imbalances that would suggest a problem with randomisation.	Low risk of bias	Participants, carers and those delivering the intervention were unaware of intervention received. Results analysed using ITT principles.	Low risk of bias	Outcome data available for 86.5% of randomised participants. No analysis to correct for bias due to missing data. Missingness balanced across arms.	Low risk of bias	Appropriate methods of measuring outcome data were used. Same study protocol for all participants and the team were masked to the group allocation of patients.	Some concerns	No protocol and statistical analysis plan are available.	Some concerns	The study is judged to raise some concerns in at least one domain for this result, but not to be at high risk of bias for any domain.
Yi 2015	Some concerns	Participants were randomised though no details of method of randomisation are recorded. There was no information relating to allocation concealment. There were no baseline imbalances that would suggest a problem with randomisation.	Low risk of bias	It was not possible to mask the participants due to functional differences in interventions being studied. Investigator providing clinical care and obtaining measurements were masked to group allocation. There were no deviations from intended interventions. Analysis were performed on the ITT principles.	Low risk of bias	Data available for approx 95% of participants.	Low risk of bias	Appropriate methods of measuring outcome data were used. Same study protocol for all participants and the team were blinded to the group allocation of patients.	Some concerns	No registry record or protocol available.	Some concerns	The study is judged to raise some concerns in at least one domain for this result, but not to be at high risk of bias for any domain.
Zhu 2021	Low risk of bias	Computer generated random sequence with allocation. No statistically significant baseline imbalances that would suggest a problem with randomisation.	Some concerns	No information on masking participants or parents. Results analysed using ITT principles.	High risk of bias	Data was available for 86.4% of participants. Reasons for missingness not documented and imbalance in missing data between intervention and control.	Low risk of bias	Appropriate and comparable methods of measuring outcome data were used. No information was available on masking of outcome assessors.	Some concerns	No registry record or protocol available.	High risk of bias	The study is judged to be at high risk of bias in at least one domain for this result.
**Subgroup 7.1.2 Atropine eyedrops (low dose)**												
Hieda 2021	Low risk of bias	Permuted block method. The allocation table was generated and maintained by a contracted professional research organization There were no baseline imbalances that would suggest a problem with randomisation.	Low risk of bias	Everyone masked to drug assignment until after study completion. Data analysed based on ITT principles.	Low risk of bias	91.7% completion rate of intervention group and 96.4% for control group.	Low risk of bias	Appropriate methods of measuring outcome data were used. Same study protocol for all participants and the team were blinded to the group allocation of patients.	Some concerns	Protocol registered. Not enough information in the protocol on a statistical plan and no published statistical plan found.	Some concerns	The study is judged to raise some concerns in at least one domain for this result, but not to be at high risk of bias for any domain.
LAMP Study 2019	Some concerns	There was no information on the method of randomisation. No details of method of allocation concealment. There were no significant differences in the baseline parameters that would suggest a problem with randomisation.	Low risk of bias	Everyone masked to group assignment until after study completion. Data analysed based on Intention to Treat principles.	Low risk of bias	Data was available for approx 87% of participants in each intervention group. No analysis performed to correct for bias due to missing data. But proportions of missing outcome data were reasonably well balanced across the arms of the trial and reasons for missingness were clearly documented.	Low risk of bias	Methods of outcome measurement were appropriate and comparable. The team were blinded to the group allocation of patients.	Some concerns	Protocol registered. Not enough information in the protocol on a statistical plan and no published statistical plan found.	Some concerns	The study is judged to raise some concerns in at least one domain for this result, but not to be at high risk of bias for any domain.
Ren 2017	Some concerns	Participants were randomised though no details of method of randomisation are recorded. There was no information relating to allocation concealment. There were no baseline imbalances that would suggest a problem with randomisation.	Some concerns	It was not possible to mask the participants due to functional differences in interventions being studied. Masking of the investigator is not reported. . No information on deviation from intended outcomes and appropriate analysis were conducted.	Low risk of bias	Data available for all participants.	Some concerns	No information about the methods of measuring the outcome or masking of the assessors were reported.	Some concerns	No registry record or protocol available.	High risk of bias	The study is judged as some concerns in four out of five domains for this result. We therefore judged that the study was at an overall high risk of bias.
Wei 2020	Some concerns	Randomisation with online software for sequence generation by third person. Insufficient information to evaluate allocation concealment. There were no baseline imbalances that would suggest a problem with randomisation.	Low risk of bias	Participants and their parents were masked. Analysis using ITT principles.	Some concerns	Data was available for 69% of participants in the intervention group and 75% in the placebo group. Reasons for missingness well documented and balanced across arms. No analysis performed to correct for bias due to missing data.	Low risk of bias	Methods of outcome measurement were appropriate and comparable. The team were masked to the group allocation of patients.	Low risk of bias	Protocol registered, with a pre published protocol and statistical plan.	Some concerns	The study is judged to raise some concerns in at least one domain for this result, but not to be at high risk of bias for any domain.
**Subgroup 7.1.3 Pirenzepine 2% gel**												
PIR‐205 Study 2004	Some concerns	Randomisation was performed using a computer generated sequence. There was no information relating to allocation concealment. There were no baseline imbalances that would suggest a problem with randomisation.	Low risk of bias	Participants, carers and those delivering the intervention were unaware of intervention received. Results analysed using ITT principles.	High risk of bias	22% of data missing from intervention group compared to 5% of control group. Reasons for missingness provided but missingness likely to depend on its true value.	Low risk of bias	Appropriate methods of measuring outcome data were used. Same study protocol for all participants and the team were blinded to the group allocation of patients.	Some concerns	No registry record or protocol available.	High risk of bias	The study is judged to be at high risk of bias in at least one domain for this result.
Tan 2005	Low risk of bias	Randomisation was performed using computer generated sequence. Allocation concealment was maintained until after enrollment and participants assigned. There were no baseline imbalances that would suggest a problem with randomisation.	Low risk of bias	Participants, carers and those delivering the intervention were unaware of intervention received. Results analysed using ITT principles.	High risk of bias	18% of data missing from intervention group compared to 13% of control group. Reasons for missingness provided but missingness in outcome probably depends on its true value.	Low risk of bias	Appropriate methods of measuring outcome data were used. Same study protocol for all participants and the team were masked to the group allocation of patients.	Some concerns	No registry record or protocol available.	High risk of bias	The study is judged to be at high risk of bias in at least one domain for this result.

**Table:** Risk of bias for analysis 7.2 Change in axial length from baseline (1 year)

**Study**	**Bias**											
	**Randomisation process**		**Deviations from intended interventions**		**Missing outcome data**		**Measurement of the outcome**		**Selection of the reported results**		**Overall**	
	**Authors' judgement**	**Support for judgement**	**Authors' judgement**	**Support for judgement**	**Authors' judgement**	**Support for judgement**	**Authors' judgement**	**Support for judgement**	**Authors' judgement**	**Support for judgement**	**Authors' judgement**	**Support for judgement**
**Subgroup 7.2.1 Atropine eyedrops (high dose)**												
ATOM Study 2006	Low risk of bias	Randomisation was performed using a computer generated randomisation list. It is unclear how allocation concelment was conducted. There were no baseline imbalances that would suggest a problem with randomisation.	Low risk of bias	Participants, carers and those delivering the intervention were unaware of intervention received. Results analysed using ITT principles.	Low risk of bias	Outcome data available for 86.5% of randomised participants. No analysis to correct for bias due to missing data. Missingness balanced across arms.	Low risk of bias	Appropriate methods of measuring outcome data were used. Same study protocol for all participants and the team were masked to the group allocation of patients.	Some concerns	No pre‐registered method (registry or protocol) available.	Some concerns	The study is judged to raise some concerns in at least one domain for this result, but not to be at high risk of bias for any domain.
Yi 2015	Some concerns	Participants were randomised though no details of method of randomisation are recorded. There was no information relating to allocation concealment. There were no baseline imbalances that would suggest a problem with randomisation.	Low risk of bias	It was not possible to mask the participants due to functional differences in interventions being studied. Investigator providing clinical care and obtaining measurements were masked to group allocation. There were no deviations from intended interventions. Analysis were performed on the ITT principles.	Low risk of bias	Data available for approx 95% of participants.	Low risk of bias	Appropriate methods of measuring outcome data were used. Same study protocol for all participants and the team were blinded to the group allocation of patients.	Some concerns	No registry record or protocol available.	Some concerns	The study is judged to raise some concerns in at least one domain for this result, but not to be at high risk of bias for any domain.
Zhu 2021	Low risk of bias	Computer generated random sequence with allocation. No statistically significant baseline imbalances that would suggest a problem with randomisation.	Some concerns	No information on masking participants or parents. Results analysed using ITT principles.	High risk of bias	Data was available for 86.4% of participants. Reasons for missingness not documented and imbalance in missing data between intervention and control.	Low risk of bias	Appropriate and comparable methods of measuring outcome data were used. No information was available on masking of outcome assessors.	Some concerns	No registry record or protocol available.	High risk of bias	The study is judged to be at high risk of bias in at least one domain for this result.
**Subgroup 7.2.2 Atropine eyedrops (low dose)**												
Hieda 2021	Low risk of bias	Permuted block method. The allocation table was generated and maintained by a contracted professional research organization There were no baseline imbalances that would suggest a problem with randomisation.	Low risk of bias	Everyone masked to drug assignment until after study completion. Data analysed based on ITT principles.	Low risk of bias	91.7% completion rate of intervention group and 96.4% for control group.	Low risk of bias	Appropriate methods of measuring outcome data were used. Same study protocol for all participants and the team were blinded to the group allocation of patients.	Some concerns	Protocol registered. Not enough information in the protocol on a statistical plan and no published statistical plan found.	Some concerns	The study is judged to raise some concerns in at least one domain for this result, but not to be at high risk of bias for any domain.
LAMP Study 2019	Some concerns	There was no information on the method of randomisation. No details of method of allocation concealment. There were no significant differences in the baseline parameters that would suggest a problem with randomisation.	Low risk of bias	Everyone masked to group assignment until after study completion. Data analysed based on Intention to Treat principles.	Low risk of bias	Data was available for approx 87% of participants in each intervention group. No analysis performed to correct for bias due to missing data. But proportions of missing outcome data were reasonably well balanced across the arms of the trial and reasons for missingness were clearly documented.	Low risk of bias	Methods of outcome measurement were appropriate and comparable. The team were blinded to the group allocation of patients.	Some concerns	Protocol registered. Not enough information in the protocol on a statistical plan and no published statistical plan found.	Some concerns	The study is judged to raise some concerns in at least one domain for this result, but not to be at high risk of bias for any domain.
Ren 2017	Some concerns	Participants were randomised though no details of method of randomisation are recorded. There was no information relating to allocation concealment. There were no baseline imbalances that would suggest a problem with randomisation.	Low risk of bias	It was not possible to mask the participants due to functional differences in interventions being studied. Masking of the investigator is not reported. No information on deviation from intended outcomes and appropriate analysis were conducted.	Low risk of bias	Data available for all participants.	Some concerns	No information about the methods of measuring the outcome or masking of the assessors were reported.	Some concerns	No registry record or protocol available.	High risk of bias	The study is judged as some concerns in four out of five domains for this result. We therefore judged that the study was at an overall high risk of bias.
Wei 2020	Some concerns	Randomisation with online software for sequence generation by third person. Insufficient information to evaluate allocation concealment. There were no baseline imbalances that would suggest a problem with randomisation.	Low risk of bias	Participants and their parents were masked. Analysis using ITT principles.	Some concerns	Data was available for 69% of participants in the intervention group and 75% in the placebo group. Reasons for missingness well documented and balanced across arms. No analysis performed to correct for bias due to missing data.	Low risk of bias	Methods of outcome measurement were appropriate and comparable. The team were masked to the group allocation of patients.	Low risk of bias	Protocol registered, with a pre published protocol and statistical plan.	Some concerns	The study is judged to raise some concerns in at least one domain for this result, but not to be at high risk of bias for any domain.
**Subgroup 7.2.3 Pirenzepine 2% gel**												
PIR‐205 Study 2004	Some concerns	Randomisation was performed using a computer generated sequence. There was no information relating to allocation concealment. There were no baseline imbalances that would suggest a problem with randomisation.	Low risk of bias	Participants, carers and those delivering the intervention were unaware of intervention received. Results analysed using ITT principles.	High risk of bias	22% of data missing from intervention group compared to 5% of control group. Reasons for missingness provided but missingness likely to depend on its true value.	Low risk of bias	Appropriate methods of measuring outcome data were used. Same study protocol for all participants and the team were masked to the group allocation of patients.	Some concerns	No registry record or protocol available.	High risk of bias	The study is judged to be at high risk of bias in at least one domain for this result.
Tan 2005	Low risk of bias	Randomisation was performed using computer generated sequence. Allocation concealment was maintained until after enrollment and participants assigned. There were no baseline imbalances that would suggest a problem with randomisation.	Low risk of bias	Participants, carers and those delivering the intervention were unaware of intervention received. Results analysed using ITT principles.	High risk of bias	18% of data missing from intervention group compared to 13% of control group. Reasons for missingness provided but missingness in outcome probably depends on its true value.	Low risk of bias	Appropriate methods of measuring outcome data were used. Same study protocol for all participants and the team were blinded to the group allocation of patients.	Some concerns	No registry record or protocol available.	High risk of bias	The study is judged to be at high risk of bias in at least one domain for this result.

**Table:** Risk of bias for analysis 7.3 Change in refractive error from baseline (2 years)

**Study**	**Bias**											
	**Randomisation process**		**Deviations from intended interventions**		**Missing outcome data**		**Measurement of the outcome**		**Selection of the reported results**		**Overall**	
	**Authors' judgement**	**Support for judgement**	**Authors' judgement**	**Support for judgement**	**Authors' judgement**	**Support for judgement**	**Authors' judgement**	**Support for judgement**	**Authors' judgement**	**Support for judgement**	**Authors' judgement**	**Support for judgement**
**Subgroup 7.3.1 Atropine eyedrops (high dose)**												
ATOM Study 2006	Low risk of bias	Randomisation was performed using a computer generated randomisation list. It is unclear how allocation concelment was conducted. There were no baseline imbalances that would suggest a problem with randomisation.	Low risk of bias	Participants, carers and those delivering the intervention were unaware of intervention received. Results analysed using ITT principles.	Low risk of bias	Outcome data available for 86.5% of randomised participants. No analysis to correct for bias due to missing data. Missingness balanced across arms.	Low risk of bias	Appropriate methods of measuring outcome data were used. Same study protocol for all participants and the team were masked to the group allocation of patients.	Some concerns	No pre‐registered method (registry or protocol) available.	Some concerns	The study is judged to raise some concerns in at least one domain for this result, but not to be at high risk of bias for any domain.
Zhu 2021	Low risk of bias	Computer generated random sequence with allocation. No statistically significant baseline imbalances that would suggest a problem with randomisation.	Some concerns	No information on masking participants or parents. Results analysed using ITT principles.	High risk of bias	Data was available for 86.4% of participants. Reasons for missingness not documented and imbalance in missing data between intervention and control.	Low risk of bias	Appropriate and comparable methods of measuring outcome data were used. No information was available on masking of outcome assessors.	Some concerns	No registry record or protocol available.	High risk of bias	The study is judged to be at high risk of bias in at least one domain for this result.
**Subgroup 7.3.2 Atropine eyedrops (low dose)**												
Hieda 2021	Low risk of bias	Permuted block method. The allocation table was generated and maintained by a contracted professional research organization There were no baseline imbalances that would suggest a problem with randomisation.	Low risk of bias	Everyone masked to drug assignment until after study completion. Data analysed based on Intention to Treat principles.	Low risk of bias	91.7% completion rate of intervention group and 96.4% for control group.	Low risk of bias	Appropriate methods of measuring outcome data were used. Same study protocol for all participants and the team were blinded to the group allocation of patients.	Some concerns	Protocol registered. Not enough information in the protocol on a statistical plan and no published statistical plan found.	Some concerns	The study is judged to raise some concerns in at least one domain for this result, but not to be at high risk of bias for any domain.
Moriche‐Carretero 2021	Some concerns	Randomisation using online software for sequence generation. Insufficient information to evaluate allocation concealment. There were no baseline imbalances that would suggest a problem with randomisation.	Some concerns	It was not possible to mask the children or carers given the nature of the interventions. Non compliant participants were excluded but unlikely to have substantial effect on outcome.	Low risk of bias	95% completion rate of intervention group and 98% for control group. Reasons for missingness documented.	Low risk of bias	Appropriate and comparable methods of measuring outcome data were used. Outcome assessors are likely to have been aware of the group allocation.	Some concerns	No pre‐registered method (registry or protocol) available.	Some concerns	The study is judged to raise some concerns in at least one domain for this result, but not to be at high risk of bias for any domain.
**Subgroup 7.3.3 Pirenzepine eyedrops 2% gel**												
PIR‐205 Study 2004	Some concerns	Randomisation was performed using a computer generated sequence. There was no information relating to allocation concealment. There were no baseline imbalances that would suggest a problem with randomisation.	Low risk of bias	Participants, carers and those delivering the intervention were unaware of intervention received. Results analysed using ITT principles.	High risk of bias	Approx 40% of those randomised to the intervention provided data at 2 years. Reasons for missingness provided but missingness likely to depend on its true value.	Low risk of bias	Appropriate methods of measuring outcome data were used. Same study protocol for all participants and the team were blinded to the group allocation of patients.	Some concerns	No registry record or protocol available.	High risk of bias	The study is judged to be at high risk of bias in at least one domain for this result.

**Table:** Risk of bias for analysis 7.4 Change in axial length from baseline (2 years)

**Study**	**Bias**											
	**Randomisation process**		**Deviations from intended interventions**		**Missing outcome data**		**Measurement of the outcome**		**Selection of the reported results**		**Overall**	
	**Authors' judgement**	**Support for judgement**	**Authors' judgement**	**Support for judgement**	**Authors' judgement**	**Support for judgement**	**Authors' judgement**	**Support for judgement**	**Authors' judgement**	**Support for judgement**	**Authors' judgement**	**Support for judgement**
**Subgroup 7.4.1 Atropine eyedrops (high dose)**												
ATOM Study 2006	Low risk of bias	Randomisation was performed using a computer generated randomisation list. It is unclear how allocation concelment was conducted. There were no baseline imbalances that would suggest a problem with randomisation.	Low risk of bias	Participants, carers and those delivering the intervention were unaware of intervention received. Results analysed using ITT principles.	Low risk of bias	Outcome data available for 86.5% of randomised participants. No analysis to correct for bias due to missing data. Missingness balanced across arms.	Low risk of bias	Appropriate methods of measuring outcome data were used. Same study protocol for all participants and the team were masked to the group allocation of patients.	Some concerns	No pre‐registered method (registry or protocol) available.	Some concerns	The study is judged to raise some concerns in at least one domain for this result, but not to be at high risk of bias for any domain.
Zhu 2021	Low risk of bias	Computer generated random sequence with allocation. No statistically significant baseline imbalances that would suggest a problem with randomisation.	Some concerns	No information on masking participants or parents. Results analysed using ITT principles.	High risk of bias	Data was available for 86.4% of participants. There is no evidence that the result was not biased by missing outcome data, and no information on whether missingness in the outcome could depend on its true value.	Low risk of bias	Appropriate and comparable methods of measuring outcome data were used. No information was available on masking of outcome assessors.	Some concerns	No registry record or protocol available.	High risk of bias	The study is judged to be at high risk of bias in at least one domain for this result.
**Subgroup 7.4.2 Atropine eyedrops (low dose)**												
Hieda 2021	Low risk of bias	Permuted block method. The allocation table was generated and maintained by a contracted professional research organization There were no baseline imbalances that would suggest a problem with randomisation.	Low risk of bias	Everyone masked to drug assignment until after study completion. Data analysed based on ITT principles.	Low risk of bias	91.7% completion rate of intervention group and 96.4% for control group.	Low risk of bias	Appropriate methods of measuring outcome data were used. Same study protocol for all participants and the team were blinded to the group allocation of patients.	Some concerns	Protocol registered. Not enough information in the protocol on a statistical plan and no published statistical plan found.	Some concerns	The study is judged to raise some concerns in at least one domain for this result, but not to be at high risk of bias for any domain.
Moriche‐Carretero 2021	Some concerns	Randomisation using online software for sequence generation. Insufficient information to evaluate allocation concealment. There were no baseline imbalances that would suggest a problem with randomisation.	Some concerns	It was not possible to mask the children or carers given the nature of the interventions. Non compliant participants were excluded but unlikely would have substantial effect on outcome.	Low risk of bias	95% completion rate of intervention group and 98% for control group. Reasons for missingness documented.	Low risk of bias	Appropriate and comparable methods of measuring outcome data were used. Outcome assessors are likely to have been aware of the group allocation.	Some concerns	No registry record or protocol available.	Some concerns	The study is judged to raise some concerns in at least one domain for this result, but not to be at high risk of bias for any domain.

**Table:** Risk of bias for analysis 7.5 Change in refractive error following cessation of treament (1 year)

**Study**	**Bias**											
	**Randomisation process**		**Deviations from intended interventions**		**Missing outcome data**		**Measurement of the outcome**		**Selection of the reported results**		**Overall**	
	**Authors' judgement**	**Support for judgement**	**Authors' judgement**	**Support for judgement**	**Authors' judgement**	**Support for judgement**	**Authors' judgement**	**Support for judgement**	**Authors' judgement**	**Support for judgement**	**Authors' judgement**	**Support for judgement**
ATOM Study 2006	Low risk of bias	Randomisation was performed using a computer generated randomisation list. It is unclear how allocation concelment was conducted. There were no baseline imbalances that would suggest a problem with randomisation.	Low risk of bias	Participants, carers and those delivering the intervention were unaware of intervention received. Results analysed using ITT principles.	Low risk of bias	Outcome data available for 86.5% of randomised participants. No analysis to correct for bias due to missing data. Missingness balanced across arms.	Low risk of bias	Appropriate methods of measuring outcome data were used. Same study protocol for all participants and the team were blinded to the group allocation of patients.	Some concerns	No protocol and statistical analysis plan are available.	Some concerns	The study is judged to raise some concerns in at least one domain for this result, but not to be at high risk of bias for any domain.
Zhu 2021	Low risk of bias	Computer generated random sequence with allocation. No statistically significant baseline imbalances that would suggest a problem with randomisation.	Some concerns	No information on masking participants or parents. Results analysed using ITT principles.	High risk of bias	Data was available for 86.4% of participants. Reasons for missingness not documented and imbalance in missing data between intervention and control.	Some concerns	Appropriate and comparable methods of measuring outcome data were used. No information was available on masking of outcome assessors.	Some concerns	No registry record or protocol available.	High risk of bias	The study is judged to be at high risk of bias in at least one domain for this result.

**Table:** Risk of bias for analysis 7.6 Change in axial length following cessation of treatment (1 year)

**Study**	**Bias**											
	**Randomisation process**		**Deviations from intended interventions**		**Missing outcome data**		**Measurement of the outcome**		**Selection of the reported results**		**Overall**	
	**Authors' judgement**	**Support for judgement**	**Authors' judgement**	**Support for judgement**	**Authors' judgement**	**Support for judgement**	**Authors' judgement**	**Support for judgement**	**Authors' judgement**	**Support for judgement**	**Authors' judgement**	**Support for judgement**
Zhu 2021	Low risk of bias	Computer generated random sequence with allocation. No statistically significant baseline imbalances that would suggest a problem with randomisation.	Some concerns	No information on masking participants or parents. Results analysed using ITT principles.	High risk of bias	Data was available for 86.4% of participants. Reasons for missingness not documented and imbalance in missing data between intervention and control.	Low risk of bias	Appropriate and comparable methods of measuring outcome data were used. No information was available on masking of outcome assessors.	Some concerns	No registry record or protocol available.	High risk of bias	The study is judged to be at high risk of bias in at least one domain for this result.

**Table:** Risk of bias for analysis 8.1 Change in refractive error from baseline (1 year)

**Study**	**Bias**											
	**Randomisation process**		**Deviations from intended interventions**		**Missing outcome data**		**Measurement of the outcome**		**Selection of the reported results**		**Overall**	
	**Authors' judgement**	**Support for judgement**	**Authors' judgement**	**Support for judgement**	**Authors' judgement**	**Support for judgement**	**Authors' judgement**	**Support for judgement**	**Authors' judgement**	**Support for judgement**	**Authors' judgement**	**Support for judgement**
Trier 2008	Low risk of bias	Randomisation was performed by the pharmacy (details not given). Allocation concealment was maintained until after enrollment and participants assigned. There were no baseline imbalances that would suggest a problem with randomisation.	Low risk of bias	Participants, carers and those delivering the intervention were unaware of intervention received. Results analysed using ITT principles.	Low risk of bias	Data available for approx 93% of participants.	Low risk of bias	Appropriate methods of measuring outcome data were used. Same study protocol for all participants and the team were masked to the group allocation of patients.	Some concerns	The outcome measurements were taken in accordance with the clinical trials registry record. No statistical analysis plan (SAP) was reported.	Some concerns	The study is judged to raise some concerns in at least one domain for this result, but not to be at high risk of bias for any domain.

**Table:** Risk of bias for analysis 8.2 Change in axial length from baseline (1 year)

**Study**	**Bias**											
	**Randomisation process**		**Deviations from intended interventions**		**Missing outcome data**		**Measurement of the outcome**		**Selection of the reported results**		**Overall**	
	**Authors' judgement**	**Support for judgement**	**Authors' judgement**	**Support for judgement**	**Authors' judgement**	**Support for judgement**	**Authors' judgement**	**Support for judgement**	**Authors' judgement**	**Support for judgement**	**Authors' judgement**	**Support for judgement**
Trier 2008	Low risk of bias	Randomisation was performed by the pharmacy (details not given). Allocation concealment was maintained until after enrollment and participants assigned. There were no baseline imbalances that would suggest a problem with randomisation.	Low risk of bias	Participants, carers and those delivering the intervention were unaware of intervention received. Results analysed using ITT principles.	Low risk of bias	Data available for approx 93% of participants.	Low risk of bias	Appropriate methods of measuring outcome data were used. Same study protocol for all participants and the team were masked to the group allocation of patients.	Some concerns	The outcome measurements were taken in accordance with the clinical trials registry record. No statistical analysis plan (SAP) was reported.	Some concerns	The study is judged to raise some concerns in at least one domain for this result, but not to be at high risk of bias for any domain.

**Table:** Risk of bias for analysis 9.1 Change in axial length

**Study**	**Bias**											
	**Randomisation process**		**Deviations from intended interventions**		**Missing outcome data**		**Measurement of the outcome**		**Selection of the reported results**		**Overall**	
	**Authors' judgement**	**Support for judgement**	**Authors' judgement**	**Support for judgement**	**Authors' judgement**	**Support for judgement**	**Authors' judgement**	**Support for judgement**	**Authors' judgement**	**Support for judgement**	**Authors' judgement**	**Support for judgement**
**Subgroup 9.1.1 At 1 year**												
Kinoshita 2020	Low risk of bias	Randomisation was performed by a third person.There were no baseline imbalances that would suggest a problem with randomisation.	Low risk of bias	It was not possible to mask the children or carers given the nature of the interventions. Results were analysed using ITT principles.	Low risk of bias	88% completion rate of intervention group 1 and 95% for intervention group 2.	Low risk of bias	Appropriate and comparable methods of measuring outcome data were used. Examiners masked to the group allocation of participants.	Some concerns	Protocol registered. Not enough information in the protocol on a statistical plan and no published statistical plan found.	Some concerns	The study is judged to raise some concerns in at least one domain for this result, but not to be at high risk of bias for any domain.
Tan 2020	Some concerns	Adaptive randomisation with online software for sequence generation. Insufficient information to evaluate allocation concealment. There were no baseline imbalances that would suggest a problem with randomisation.	Some concerns	It was not possible to mask the children or carers given the nature of the interventions. A small numer refused allocation but unlikely would have substantial effect on outcome.	Some concerns	Data was available for approx 87% of participants in each intervention group. No analysis performed to correct for bias due to missing data. Reasons for missingness provided.	Low risk of bias	Appropriate and comparable methods of measuring outcome data were used.	Low risk of bias	Protocol registered, with a pre published protocol and statistical plan.	Some concerns	The study is judged to raise some concerns in at least one domain for this result, but not to be at high risk of bias for any domain.
Zhao 2021	Some concerns	There was no information on the method of randomisation or allocation concealment. There were no significant differences in the baseline parameters that would suggest a problem with randomisation.	Some concerns	No attempt to mask participants or parents. Method of analysis unclear.	Some concerns	No information on missingness and unclear whether missingness in the outcome could depend on its true value.	Some concerns	Appropriate and comparable methods of measuring outcome data were used. No information was available on masking of outcome assessors. Unlikely that assessment of the outcome was influenced by knowledge of intervention received.	Some concerns	No registry record or protocol available.	High risk of bias	The study is judged as some concerns in all 5 domains and therefore at high risk of bias overall
**Subgroup 9.1.2 At 2 years**												
Kinoshita 2020	Low risk of bias	Randomisation was performed by a third person.There were no baseline imbalances that would suggest a problem with randomisation.	Low risk of bias	It was not possible to mask the children or carers given the nature of the interventions. Results were analysed using ITT principles.	Low risk of bias	88% completion rate of intervention group 1 and 95% for intervention group 2.	Low risk of bias	Appropriate and comparable methods of measuring outcome data were used. Examiners masked to the group allocation of participants.	Some concerns	Protocol registered. Not enough information in the protocol on a statistical plan and no published statistical plan found.	Some concerns	The study is judged to raise some concerns in at least one domain for this result, but not to be at high risk of bias for any domain.

## Appendices

### Appendix 1. CENTRAL search strategy

#1 MeSH descriptor: [Myopia] explode all trees #2 myop* #3 short near sight* #4 #1 or #2 or #3 #5 (undercorrect* or slow* or progress* or control* or retard* or funct*) near/5 (myopia or myopic or myopes) #6 (bifocal or multifocal) near/4 (myopia or myopic) near/4 (slow* or progress* or control*) #7 prismatic bifocal* #8 prism near/2 bifocal* #9 base‐in prism #10 executive near/2 bifocal* #11 progressive next addition near/3 lens* #12 positive next lens* near/3 addition #13 PA‐PALs #14 peripheral near/2 defocus near/4 lens* #15 Defocus Incorporated Multiple Segments #16 MyoVision or MyopiLux or Myosmart #17 #6 or #7 or #8 or #9 or #10 or #11 or #12 or #13 or #14 or #15 or #16 #18 (Concentric or gradient) near/3 lens* #19 dual near/2 focus* #20 extend* near/2 depth near/3 focus #21 extend* near/2 depth near/4 field* #22 extend* near/2 range near/3 focus #23 extend* near/2 range near/4 field* #24 extend* near/2 DOF #25 EDOF #26 #18 or #19 or #20 or #21 or #22 or #23 or #24 or #25 #27 #5 and #26 #28 MiSight or Biofinity Multifocal or Proclear Multifocal #29 MeSH descriptor: [Orthokeratologic Procedures] explode all trees #30 orthokeratology or Ortho‐K #31 #28 or #29 or #30 #32 MeSH descriptor: [Atropine] explode all trees #33 atropine* #34 MeSH descriptor: [Cyclopentolate] explode all trees #35 cyclopentolate* #36 MeSH descriptor: [Pirenzepine] explode all trees #37 pirenzepine* #38 MeSH descriptor: [Tropicamide] explode all trees #39 tropicamide* #40 methylxanthine* #41 #5 #32 or #33 or #34 or #35 #36 or #37 or #38 or #39 or #40 #42 MeSH descriptor: [Leisure Activities] explode all trees #43 outdoor* or out door* #44 outside or out side #45 #42 or #43 or #44 #46 #5 or #17 or #27 or #31 or #41 or #45 #47 MeSH descriptor: [Child] explode all trees #48 MeSH descriptor: [Adolescent] this term only #49 MeSH descriptor: [Pediatrics] explode all trees #50 boy* or girl* or child* or minor* #51 adolescen* or juvenile* or teen or teens or teenage* or youth or youths or underage #52 (primary or elementary or high or secondary) near/1 school* #53 paediatric* or pediatric* #54 #47 or #48 or #49 or #50 or #51 or #52 or #53 #55 #4 and #46 #56 #54 and #55

### Appendix 2. MEDLINE Ovid search strategy

1. randomized controlled trial.pt. 2. (randomized or randomised).ab,ti. 3. placebo.ab,ti. 4. dt.fs. 5. randomly.ab,ti. 6. trial.ab,ti. 7. groups.ab,ti. 8. or/1‐7 9. exp animals/ 10. exp humans/ 11. 9 not (9 and 10) 12. 8 not 11 13. exp myopia/ 14. (myopia or myopic or myopes).tw. 15. ((short or near) adj3 sight$).tw. 16. or/13‐15 17. ((undercorrect$ or slow$ or progress$ or control$ or retard$ or funct$) adj5 (myopia or myopic or myopes)).tw. 18. ((bifocal or multifocal) adj4 (myopia or myopic) adj4 (slow$ or progress$ or control$)).tw. 19. prismatic bifocal$.tw. 20. (near adj1 prism adj4 bifocal$).tw. 21. base‐in prism.tw. 22. (executive adj2 bifocal$).tw. 23. (progressive adj1 addition adj3 lens$).tw. 24. (positive adj1 lens$ adj3 addition).tw. 25. PA‐PALs.tw. 26. (peripheral adj2 defocus adj4 lens$).tw. 27. Defocus Incorporated Multiple Segments.tw. 28. (MyoVision or MyopiLux or Myosmart).tw. 29. or/18‐28 30. ((Concentric or gradient) adj3 lens$).tw. 31. (dual adj2 focus$).tw. 32. (extend$ adj2 depth adj3 focus).tw. 33. (extend$ adj2 depth adj4 field$).tw. 34. (extend$ adj2 range adj3 focus).tw. 35. (extend$ adj2 range adj4 field$).tw. 36. (extend$ adj2 DOF).tw. 37. EDOF.tw. 38. or/30‐37 39. 17 and 38 40. (MiSight or Biofinity Multifocal or Proclear Multifocal).tw. 41. Orthokeratologic Procedures/ 42. (orthokeratology or Ortho‐K).tw. 43. or/40‐42 44. Atropine/ 45. atropine$.tw. 46. Cyclopentolate/ 47. cyclopentolate$.tw. 48. Pirenzepine/ 49. pirenzepine$.tw. 50. Tropicamide/ 51. tropicamide$.tw. 52. methylxanthine$.tw. 53. or/44‐52 54. exp Leisure Activities/ 55. (outdoor$ or out door$).tw. 56. (outside or out side).tw. 57. (near adj2 work$).tw. 58. or/54‐57 59. 17 or 29 or 39 or 43 or 53 or 58 60. exp Child/ 61. Adolescent/ 62. exp Pediatrics/ 63. (boy$ or girl$ or child$ or minor$).tw. 64. (adolescen$ or juvenile$ or teen or teens or teenage$ or youth or youths or underage).tw. 65. ((primary or elementary or high or secondary) adj1 school$).tw. 66. (schoolchild$ or schoolage or schoolboy$ orschoolgirl$ or highschool$).tw. 67. (paediatric$ or pediatric$).tw. 68. or/60‐67 69. 16 and 59 70. 12 and 69 71. 68 and 70

The search filter for trials at the beginning of the MEDLINE strategy is from the published paper by Glanville 2006.

### Appendix 3. MEDLINE Ovid economics search strategy

1. Economics/ 2. exp "costs and cost analysis"/ 3. Economics, Dental/ 4. exp economics, hospital/ 5. Economics, Medical/ 6. Economics, Nursing/ 7. Economics, Pharmaceutical/ 8. (economic$ or cost or costs or costly or costing or price or prices or pricing or pharmacoeconomic$).ti,ab. 9. (expenditure$ not energy).ti,ab. 10. value for money.ti,ab. 11. budget$.ti,ab. 12. or/1‐11 13. ((energy or oxygen) adj cost).ti,ab. 14. (metabolic adj cost).ti,ab. 15. ((energy or oxygen) adj expenditure).ti,ab. 16. or/13‐15 17. 12 not 16 18. letter.pt. 19. editorial.pt. 20. historical article.pt. 21. or/18‐20 22. 17 not 21 23. exp animals/ not humans/ 24. 22 not 23 25. bmj.jn. 26. "cochrane database of systematic reviews".jn. 27. health technology assessment winchester england.jn. 28. or/25‐27 29. exp myopia/ 30. (myopia or myopic or myopes).tw. 31. ((short or near) adj3 sight$).tw. 32. or/29‐31 33. ((undercorrect$ or slow$ or progress$ or control$ or retard$ or funct$) adj5 (myopia or myopic or myopes)).tw. 34. ((bifocal or multifocal) adj4 (myopia or myopic) adj4 (slow$ or progress$ or control$)).tw. 35. prismatic bifocal$.tw. 36. (near adj1 prism adj4 bifocal$).tw. 37. base‐in prism.tw. 38. (executive adj2 bifocal$).tw. 39. (progressive adj1 addition adj3 lens$).tw. 40. (positive adj1 lens$ adj3 addition).tw. 41. PA‐PALs.tw. 42. (peripheral adj2 defocus adj4 lens$).tw. 43. Defocus Incorporated Multiple Segments.tw. 44. (MyoVision or MyopiLux or Myosmart).tw. 45. or/34‐44 46. ((Concentric or gradient) adj3 lens$).tw. 47. (dual adj2 focus$).tw. 48. (extend$ adj2 depth adj3 focus).tw. 49. (extend$ adj2 depth adj4 field$).tw. 50. (extend$ adj2 range adj3 focus).tw. 51. (extend$ adj2 range adj4 field$).tw. 52. (extend$ adj2 DOF).tw. 53. EDOF.tw. 54. or/46‐53 55. 33 and 54 56. (MiSight or Biofinity Multifocal or Proclear Multifocal).tw. 57. Orthokeratologic Procedures/ 58. (orthokeratology or Ortho‐K).tw. 59. or/56‐58 60. Atropine/ 61. atropine$.tw. 62. Cyclopentolate/ 63. cyclopentolate$.tw. 64. Pirenzepine/ 65. pirenzepine$.tw. 66. Tropicamide/ 67. tropicamide$.tw. 68. methylxanthine$.tw. 69. or/60‐68 70. exp Leisure Activities/ 71. (outdoor$ or out door$).tw. 72. (outside or out side).tw. 73. (near adj2 work$).tw. 74. or/70‐73 75. 33 or 45 or 55 or 59 or 69 or 74 76. 32 and 75 77. 28 and 76

### Appendix 4. MEDLINE Ovid adverse events search strategy

1. (ae or co or de).fs. 2. (safe or safety or side effect$ or undesirable effect$ or treatment emergent or tolerability or toxicity or adrs).ti,ab. 3. (adverse adj2 (effect or effects or reaction or reactions or event or events or outcome or outcomes)).ti,ab. 4. or/1‐3 5. exp myopia/ 6. (myopia or myopic or myopes).tw. 7. ((short or near) adj3 sight$).tw. 8. or/5‐7 9. ((undercorrect$ or slow$ or progress$ or control$ or retard$ or funct$) adj5 (myopia or myopic or myopes)).tw. 10. ((bifocal or multifocal) adj4 (myopia or myopic) adj4 (slow$ or progress$ or control$)).tw. 11. prismatic bifocal$.tw. 12. (near adj1 prism adj4 bifocal$).tw. 13. base‐in prism.tw. 14. (executive adj2 bifocal$).tw. 15. (progressive adj1 addition adj3 lens$).tw. 16. (positive adj1 lens$ adj3 addition).tw. 17. PA‐PALs.tw. 18. (peripheral adj2 defocus adj4 lens$).tw. 19. Defocus Incorporated Multiple Segments.tw. 20. (MyoVision or MyopiLux or Myosmart).tw. 21. or/10‐20 22. ((Concentric or gradient) adj3 lens$).tw. 23. (dual adj2 focus$).tw. 24. (extend$ adj2 depth adj3 focus).tw. 25. (extend$ adj2 depth adj4 field$).tw. 26. (extend$ adj2 range adj3 focus).tw. 27. (extend$ adj2 range adj4 field$).tw. 28. (extend$ adj2 DOF).tw. 29. EDOF.tw. 30. or/22‐29 31. 9 and 30 32. (MiSight or Biofinity Multifocal or Proclear Multifocal).tw. 33. Orthokeratologic Procedures/ 34. (orthokeratology or Ortho‐K).tw. 35. or/32‐34 36. Atropine/ 37. atropine$.tw. 38. Cyclopentolate/ 39. cyclopentolate$.tw. 40. Pirenzepine/ 41. pirenzepine$.tw. 42. Tropicamide/ 43. tropicamide$.tw. 44. methylxanthine$.tw. 45. or/36‐44 46. exp Leisure Activities/ 47. (outdoor$ or out door$).tw. 48. (outside or out side).tw. 49. (near adj2 work$).tw. 50. or/46‐49 51. 9 or 21 or 31 or 35 or 45 or 50 52. 8 and 51 53. 4 and 52

The search filter for trials at the beginning of the MEDLINE strategy is from the published paper by Golder 2006

### Appendix 5. Embase Ovid search strategy

1. exp randomized controlled trial/ 2. exp randomization/ 3. exp double blind procedure/ 4. exp single blind procedure/ 5. random$.tw. 6. or/1‐5 7. (animal or animal experiment).sh. 8. human.sh. 9. 7 and 8 10. 7 not 9 11. 6 not 10 12. exp clinical trial/ 13. (clin$ adj3 trial$).tw. 14. ((singl$ or doubl$ or trebl$ or tripl$) adj3 (blind$ or mask$)).tw. 15. exp placebo/ 16. placebo$.tw. 17. random$.tw. 18. exp experimental design/ 19. exp crossover procedure/ 20. exp control group/ 21. exp latin square design/ 22. or/12‐21 23. 22 not 10 24. 23 not 11 25. exp comparative study/ 26. exp evaluation/ 27. exp prospective study/ 28. (control$ or prospectiv$ or volunteer$).tw. 29. or/25‐28 30. 29 not 10 31. 30 not (11 or 23) 32. 11 or 24 or 31 33. myopia/ 34. (myopia or myopic or myopes).tw. 35. ((short or near) adj3 sight$).tw. 36. or/33‐35 37. ((undercorrect$ or slow$ or progress$ or control$ or retard$ or funct$) adj5 (myopia or myopic or myopes)).tw. 38. ((bifocal or multifocal) adj4 (myopia or myopic) adj4 (slow$ or progress$ or control$)).tw. 39. prismatic bifocal$.tw. 40. (near adj1 prism adj4 bifocal$).tw. 41. base‐in prism.tw. 42. (executive adj2 bifocal$).tw. 43. (progressive adj1 addition adj3 lens$).tw. 44. (positive adj1 lens$ adj3 addition).tw. 45. PA‐PALs.tw. 46. (peripheral adj2 defocus adj4 lens$).tw. 47. Defocus Incorporated Multiple Segments.tw. 48. (MyoVision or MyopiLux or Myosmart).tw. 49. or/38‐48 50. ((Concentric or gradient) adj3 lens$).tw. 51. (dual adj2 focus$).tw. 52. (extend$ adj2 depth adj3 focus).tw. 53. (extend$ adj2 depth adj4 field$).tw. 54. (extend$ adj2 range adj3 focus).tw. 55. (extend$ adj2 range adj4 field$).tw. 56. (extend$ adj2 DOF).tw. 57. EDOF.tw. 58. or/50‐57 59. 37 and 58 60. (MiSight or Biofinity Multifocal or Proclear Multifocal).tw. 61. orthokeratology lens/ 62. (orthokeratology or Ortho‐K).tw. 63. or/60‐62 64. atropine/ 65. atropine$.tw. 66. cyclopentolate/ 67. cyclopentolate$.tw. 68. pirenzepine/ 69. pirenzepine$.tw. 70. tropicamide/ 71. tropicamide$.tw. 72. methylxanthine/ 73. methylxanthine.tw. 74. or/64‐73 75. exp recreation/ 76. (outdoor$ or out door$).tw. 77. (outside or out side).tw. 78. (near adj2 work$).tw. 79. or/75‐78 80. exp child/ 81. exp adolescent/ 82. exp pediatrics/ 83. (boy$ or girl$ or child$ or minor$).tw. 84. (adolescen$ or juvenile$ or teen or teens or teenage$ or youth or youths or underage).tw. 85. ((primary or elementary or high or secondary) adj1 school$).tw. 86. (schoolchild$ or schoolage or schoolboy$ orschoolgirl$ or highschool$).tw. 87. (paediatric$ or pediatric$).tw. 88. or/80‐87 89. 37 or 49 or 59 or 63 or 74 or 79 90. 36 and 89 91. 32 and 90 92. 88 and 91

### Appendix 6. Embase Ovid economics search strategy

1. Health Economics/ 2. exp Economic Evaluation/ 3. exp Health Care Cost/ 4. pharmacoeconomics/ 5. or/1‐4 6. (econom$ or cost or costs or costly or costing or price or prices or pricing or pharmacoeconomic$).ti,ab. 7. (expenditure$ not energy).ti,ab. 8. (value adj2 money).ti,ab. 9. budget$.ti,ab. 10. or/6‐9 11. 5 or 10 12. letter.pt. 13. editorial.pt. 14. note.pt. 15. or/12‐14 16. 11 not 15 17. (metabolic adj cost).ti,ab. 18. ((energy or oxygen) adj cost).ti,ab. 19. ((energy or oxygen) adj expenditure).ti,ab. 20. or/17‐19 21. 16 not 20 22. animal/ 23. exp animal experiment/ 24. nonhuman/ 25. (rat or rats or mouse or mice or hamster or hamsters or animal or animals or dog or dogs or cat or cats or bovine or sheep).ti,ab,sh. 26. or/22‐25 27. exp human/ 28. human experiment/ 29. or/27‐28 30. 26 not (26 and 29) 31. 21 not 30 32. 0959‐8146.is. 33. (1469‐493X or 1366‐5278).is. 34. 1756‐1833.en. 35. or/32‐34 36. 31 not 35 37. Conference abstract.pt. 38. 36 not 37 39. myopia/ 40. (myopia or myopic or myopes).tw. 41. ((short or near) adj3 sight$).tw. 42. or/39‐41 43. ((undercorrect$ or slow$ or progress$ or control$ or retard$ or funct$) adj5 (myopia or myopic or myopes)).tw. 44. ((bifocal or multifocal) adj4 (myopia or myopic) adj4 (slow$ or progress$ or control$)).tw. 45. prismatic bifocal$.tw. 46. (near adj1 prism adj4 bifocal$).tw. 47. base‐in prism.tw. 48. (executive adj2 bifocal$).tw. 49. (progressive adj1 addition adj3 lens$).tw. 50. (positive adj1 lens$ adj3 addition).tw. 51. PA‐PALs.tw. 52. (peripheral adj2 defocus adj4 lens$).tw. 53. Defocus Incorporated Multiple Segments.tw. 54. (MyoVision or MyopiLux or Myosmart).tw. 55. or/44‐54 56. ((Concentric or gradient) adj3 lens$).tw. 57. (dual adj2 focus$).tw. 58. (extend$ adj2 depth adj3 focus).tw. 59. (extend$ adj2 depth adj4 field$).tw. 60. (extend$ adj2 range adj3 focus).tw. 61. (extend$ adj2 range adj4 field$).tw. 62. (extend$ adj2 DOF).tw. 63. EDOF.tw. 64. or/56‐63 65. 43 and 64 66. (MiSight or Biofinity Multifocal or Proclear Multifocal).tw. 67. orthokeratology lens/ 68. (orthokeratology or Ortho‐K).tw. 69. or/66‐68 70. atropine/ 71. atropine$.tw. 72. cyclopentolate/ 73. cyclopentolate$.tw. 74. pirenzepine/ 75. pirenzepine$.tw. 76. tropicamide/ 77. tropicamide$.tw. 78. methylxanthine/ 79. methylxanthine.tw. 80. or/70‐79 81. exp recreation/ 82. (outdoor$ or out door$).tw. 83. (outside or out side).tw. 84. (near adj2 work$).tw. 85. or/81‐84 86. 43 or 55 or 65 or 69 or 80 or 85 87. 42 and 86 88. 38 and 87

### Appendix 7. Embase Ovid adverse events search strategy

1. DRUG/ae 2. (safe or safety or side effect$ or undesirable effect$ or treatment emergent or tolerability or toxicity or adrs).ti,ab. 3. (adverse adj2 (effect or effects or reaction or reactions or event or events or outcome or outcomes)).ti,ab. 4. or/1‐3 5. myopia/ 6. (myopia or myopic or myopes).tw. 7. ((short or near) adj3 sight$).tw. 8. or/5‐7 9. ((undercorrect$ or slow$ or progress$ or control$ or retard$ or funct$) adj5 (myopia or myopic or myopes)).tw. 10. ((bifocal or multifocal) adj4 (myopia or myopic) adj4 (slow$ or progress$ or control$)).tw. 11. prismatic bifocal$.tw. 12. (near adj1 prism adj4 bifocal$).tw. 13. base‐in prism.tw. 14. (executive adj2 bifocal$).tw. 15. (progressive adj1 addition adj3 lens$).tw. 16. (positive adj1 lens$ adj3 addition).tw. 17. PA‐PALs.tw. 18. (peripheral adj2 defocus adj4 lens$).tw. 19. Defocus Incorporated Multiple Segments.tw. 20. (MyoVision or MyopiLux or Myosmart).tw. 21. or/10‐20 22. ((Concentric or gradient) adj3 lens$).tw. 23. (dual adj2 focus$).tw. 24. (extend$ adj2 depth adj3 focus).tw. 25. (extend$ adj2 depth adj4 field$).tw. 26. (extend$ adj2 range adj3 focus).tw. 27. (extend$ adj2 range adj4 field$).tw. 28. (extend$ adj2 DOF).tw. 29. EDOF.tw. 30. or/22‐29 31. 9 and 30 32. (MiSight or Biofinity Multifocal or Proclear Multifocal).tw. 33. orthokeratology lens/ 34. (orthokeratology or Ortho‐K).tw. 35. or/32‐34 36. atropine/ 37. atropine$.tw. 38. cyclopentolate/ 39. cyclopentolate$.tw. 40. pirenzepine/ 41. pirenzepine$.tw. 42. tropicamide/ 43. tropicamide$.tw. 44. methylxanthine/ 45. methylxanthine.tw. 46. or/36‐45 47. exp recreation/ 48. (outdoor$ or out door$).tw. 49. (outside or out side).tw. 50. (near adj2 work$).tw. 51. or/47‐50 52. 9 or 21 or 31 or 35 or 46 or 51 53. 8 and 52 54. 4 and 53

The search filter for trials at the beginning of the MEDLINE strategy is from the published paper by Golder 2006.

### Appendix 8. ISRCTN search strategy

myopia AND (undercorrect OR slow OR progress OR control)

### Appendix 9. ClinicalTrials.gov search strategy

myopia AND (undercorrect OR slow OR progress OR control) | Interventional Studies | Child

### Appendix 10. WHO ICTRP search strategy

myopia AND undercorrect OR myopia AND slow OR myopia AND progress OR myopia AND control

## Data and analyses

**Comparison 1:** Undercorrection vs full correction spectacles

Outcome or subgroup title	No. of studies	No. of participants	Statistical method	Effect size
1.1 Change in refractive error from baseline	3		Mean Difference (IV, Fixed, 95% CI)	Subtotals only
1.1.1 At 1 year	2	142	Mean Difference (IV, Fixed, 95% CI)	‐0.15 [‐0.29, ‐0.00]
1.1.2 At 2 years	2	244	Mean Difference (IV, Fixed, 95% CI)	0.02 [‐0.05, 0.09]
1.2 Change in axial length from baseline	2		Mean Difference (IV, Fixed, 95% CI)	Subtotals only
1.2.1 At 1 year	1	94	Mean Difference (IV, Fixed, 95% CI)	0.05 [‐0.01, 0.11]
1.2.2 At 2 years	2	244	Mean Difference (IV, Fixed, 95% CI)	‐0.01 [‐0.06, 0.03]

**Comparison 2:** Multifocal spectacle lenses vs single vision spectacle lenses

Outcome or subgroup title	No. of studies	No. of participants	Statistical method	Effect size
2.1 Change in refractive error from baseline	10		Mean Difference (IV, Random, 95% CI)	Subtotals only
2.1.1 At 1 year	9	1463	Mean Difference (IV, Random, 95% CI)	0.14 [0.08, 0.21]
2.1.2 At 2 years	8	1401	Mean Difference (IV, Random, 95% CI)	0.19 [0.08, 0.30]
2.1.3 At 3 years	4	835	Mean Difference (IV, Random, 95% CI)	0.26 [‐0.07, 0.59]
2.2 Change in axial length from baseline	4		Mean Difference (IV, Fixed, 95% CI)	Subtotals only
2.2.1 At 1 year	4	896	Mean Difference (IV, Fixed, 95% CI)	‐0.06 [‐0.09, ‐0.04]
2.2.2 At 2 years	3	699	Mean Difference (IV, Fixed, 95% CI)	‐0.07 [‐0.12, ‐0.03]
2.2.3 At 3 years	2	558	Mean Difference (IV, Fixed, 95% CI)	‐0.12 [‐0.18, ‐0.07]
2.3 Change in refractive error following cessation of treatment (1 year)	1		Mean Difference (IV, Fixed, 95% CI)	Totals not selected

**Comparison 3:** Peripheral plus spectacles vs single vision spectacle lenses

Outcome or subgroup title	No. of studies	No. of participants	Statistical method	Effect size
3.1 Change in refractive error from baseline	6		Mean Difference (IV, Random, 95% CI)	Subtotals only
3.1.1 At 1 year	5	832	Mean Difference (IV, Random, 95% CI)	0.51 [0.19, 0.82]
3.1.2 At 2 years	2	329	Mean Difference (IV, Random, 95% CI)	0.34 [‐0.08, 0.76]
3.2 Change in axial length from baseline	4		Mean Difference (IV, Random, 95% CI)	Subtotals only
3.2.1 At 1 year	3	522	Mean Difference (IV, Random, 95% CI)	‐0.13 [‐0.24, ‐0.03]
3.2.2 At 2 years	2	329	Mean Difference (IV, Random, 95% CI)	‐0.20 [‐0.45, 0.05]

**Comparison 4:** Multifocal soft contact lenses vs single vision soft contact lenses

Outcome or subgroup title	No. of studies	No. of participants	Statistical method	Effect size
4.1 Change in refractive error from baseline	8		Mean Difference (IV, Random, 95% CI)	Subtotals only
4.1.1 At 1 year	8	1135	Mean Difference (IV, Random, 95% CI)	0.26 [0.17, 0.35]
4.1.2 At 2 years	5	843	Mean Difference (IV, Random, 95% CI)	0.30 [0.19, 0.41]
4.1.3 At 3 years	2	395	Mean Difference (IV, Random, 95% CI)	0.47 [0.13, 0.82]
4.2 Change in axial length from baseline	8		Mean Difference (IV, Random, 95% CI)	Subtotals only
4.2.1 At 1 year	8	1143	Mean Difference (IV, Random, 95% CI)	‐0.11 [‐0.13, ‐0.09]
4.2.2 At 2 years	5	843	Mean Difference (IV, Random, 95% CI)	‐0.15 [‐0.19, ‐0.12]
4.2.3 At 3 years	2	394	Mean Difference (IV, Random, 95% CI)	‐0.22 [‐0.34, ‐0.10]
4.3 Change in refractive error following cessation of treatment (1 year)	1		Mean Difference (IV, Fixed, 95% CI)	Totals not selected
4.4 Change in axial length following cessation of treatment (1 year)	1		Mean Difference (IV, Fixed, 95% CI)	Totals not selected

**Comparison 5:** Rigid gas‐permeable lenses vs control

Outcome or subgroup title	No. of studies	No. of participants	Statistical method	Effect size
5.1 Change in refractive error from baseline	2		Mean Difference (IV, Fixed, 95% CI)	Totals not selected
5.1.1 At 1 year	2		Mean Difference (IV, Fixed, 95% CI)	Totals not selected
5.1.2 At 2 years	2		Mean Difference (IV, Fixed, 95% CI)	Totals not selected
5.1.3 At 3 years	1		Mean Difference (IV, Fixed, 95% CI)	Totals not selected
5.2 Change in axial length from baseline	2		Mean Difference (IV, Fixed, 95% CI)	Subtotals only
5.2.1 At 1 year	2	415	Mean Difference (IV, Fixed, 95% CI)	0.02 [‐0.05, 0.10]
5.2.2 At 2 years	2	394	Mean Difference (IV, Fixed, 95% CI)	0.03 [‐0.05, 0.12]
5.2.3 At 3 years	1	116	Mean Difference (IV, Fixed, 95% CI)	0.05 [‐0.12, 0.22]

**Comparison 6:** Orthokeratology lenses vs single vision spectacle lenses lenses

Outcome or subgroup title	No. of studies	No. of participants	Statistical method	Effect size
6.1 Change in axial length from baseline	8		Mean Difference (IV, Random, 95% CI)	Subtotals only
6.1.1 At 1 year	7	759	Mean Difference (IV, Random, 95% CI)	‐0.19 [‐0.23, ‐0.15]
6.1.2 At 2 years	2	106	Mean Difference (IV, Random, 95% CI)	‐0.28 [‐0.38, ‐0.19]

**Comparison 7:** Anti‐muscarinics vs placebo

Outcome or subgroup title	No. of studies	No. of participants	Statistical method	Effect size
7.1 Change in refractive error from baseline (1 year)	9		Mean Difference (IV, Random, 95% CI)	Subtotals only
7.1.1 Atropine (high dose)	3	1072	Mean Difference (IV, Random, 95% CI)	0.90 [0.62, 1.18]
7.1.2 Atropine eyedrops (low dose)	4	804	Mean Difference (IV, Random, 95% CI)	0.38 [0.10, 0.66]
7.1.3 Pirenzepine 2% gel	2	326	Mean Difference (IV, Random, 95% CI)	0.32 [0.15, 0.49]
7.2 Change in axial length from baseline (1 year)	9		Mean Difference (IV, Random, 95% CI)	Subtotals only
7.2.1 Atropine eyedrops (high dose)	3	1072	Mean Difference (IV, Random, 95% CI)	‐0.33 [‐0.35, ‐0.30]
7.2.2 Atropine eyedrops (low dose)	4	804	Mean Difference (IV, Random, 95% CI)	‐0.13 [‐0.21, ‐0.05]
7.2.3 Pirenzepine 2% gel	2	326	Mean Difference (IV, Random, 95% CI)	‐0.10 [‐0.18, ‐0.02]
7.3 Change in refractive error from baseline (2 years)	5		Mean Difference (IV, Fixed, 95% CI)	Subtotals only
7.3.1 Atropine eyedrops (high dose)	2	916	Mean Difference (IV, Fixed, 95% CI)	1.26 [1.17, 1.36]
7.3.2 Atropine eyedrops (low dose)	2	497	Mean Difference (IV, Fixed, 95% CI)	0.24 [0.17, 0.31]
7.3.3 Pirenzepine eyedrops 2% gel	1	84	Mean Difference (IV, Fixed, 95% CI)	0.41 [0.13, 0.69]
7.4 Change in axial length from baseline (2 years)	4		Mean Difference (IV, Random, 95% CI)	Subtotals only
7.4.1 Atropine eyedrops (high dose)	2	916	Mean Difference (IV, Random, 95% CI)	‐0.47 [‐0.61, ‐0.34]
7.4.2 Atropine eyedrops (low dose)	2	497	Mean Difference (IV, Random, 95% CI)	‐0.16 [‐0.20, ‐0.12]
7.5 Change in refractive error following cessation of treament (1 year)	2		Mean Difference (IV, Fixed, 95% CI)	Totals not selected
7.6 Change in axial length following cessation of treatment (1 year)	1		Mean Difference (IV, Fixed, 95% CI)	Totals not selected

**Comparison 8:** 7‐methylxanthine vs placebo

Outcome or subgroup title	No. of studies	No. of participants	Statistical method	Effect size
8.1 Change in refractive error from baseline (1 year)	1	77	Mean Difference (IV, Fixed, 95% CI)	0.07 [‐0.09, 0.24]
8.2 Change in axial length from baseline (1 year)	1	77	Mean Difference (IV, Fixed, 95% CI)	‐0.03 [‐0.10, 0.03]

**Comparison 9:** Othokeratology plus atropine vs orthokeratology alone

Outcome or subgroup title	No. of studies	No. of participants	Statistical method	Effect size
9.1 Change in axial length	3		Mean Difference (IV, Fixed, 95% CI)	Subtotals only
9.1.1 At 1 year	3	172	Mean Difference (IV, Fixed, 95% CI)	‐0.13 [‐0.16, ‐0.09]
9.1.2 At 2 years	1	73	Mean Difference (IV, Fixed, 95% CI)	‐0.11 [‐0.21, ‐0.01]

## Characteristics of studies

### Characteristics of included studies [ordered by study ID]

**Table:** Adler 2006

***Study characteristics***	
Methods	Study design: parallel‐group RCT Study centre: urban private optometric practice in Jerusalem, Israel Number randomised: 62 children Study follow‐up: 18 months Exclusions and losses to follow‐up: 5 (8%) children who were randomised were excluded from the analyses; 9 (14.5%) were lost to follow‐up
Participants	Age: mean = 10.08 years (range 6‐15 years) Gender: 34 boys, 14 girls Culture: most children were orthodox Jews who attended school year‐round and performed a study method of swaying back and forth while learning and reading Inclusion criteria: pediatric patients aged 6‐15 years from study centres with early‐onset myopia Exclusion criteria: strabismus amblyopia VA < 6/9 spherical equivalent > ‐6.00 D or < ‐0.50 D in either eye astigmatism > 1.50 D in either eye anisometropia > 1.50 D a difference between objective and subjective refraction findings ≥ 0.75 D any ocular pathological manifestations premature birth
Interventions	Undercorrected group (n = 25): blurred by +0.50 D; glasses were to be worn continuously Fully corrected group (n = 23): glasses were to be worn continuously Note: changes in prescription were made if the subjective refraction had changed by ≥ 0.50 D for 1 or both eyes
Outcomes	**Progression of early‐onset myopia** Objective refractions without cycloplegia: static retinoscopy (spherical equivalent) Subjective refractions without cycloplegia: endpoint of maximum plus for best acuity Near lateral phoria: alternating cover test using 6/9 size picture target held at 40 cm from eye Measurements taken at baseline, 6 months, 12 months, and 18 months **Unit of analysis:** average values of both eyes used for all results
Notes	Study dates: enrolment occurred over an 8‐month period Trial registration: not reported Materials: free spectacle lenses were supplied by Einit Optical Clinic Additional data: study author provided unpublished data via email correspondence

**Table:** Anstice 2011

***Study characteristics***	
Methods	Study design: paired‐eye, cross‐over RCT Study centre: 1 Number randomised: 40 children Study follow‐up: 20 months (10 months for each period) Exclusions and losses to follow‐up: no exclusions; 5 (12.5%) and 6 (15.0%) were lost to follow‐up at 10‐month visit and 20‐month visit, respectively
Participants	Age: mean = 13.4 years (range 11‐14 years) Gender: 11 boys, 29 girls Culture: New Zealand, including East Asian ethnicity and others (European, Indian, and Maori/Pacifica) Inclusion criteria: 11‐14 years old at recruitment spherical equivalent between −1.25 and −4.50 D in the least myopic eye as determined by noncycloplegic subjective refraction myopia progression ≥ 0.50 D in the previous 12 months best‐corrected spectacle VA of Snellen 6/6 or better in each eye willingness to wear contact lenses for ≥ 8 h/day during the study Exclusion criteria: history of astigmatism ≥ 1.25 D anisometropia ≥ 1.00 D strabismus at distance or near as assessed by cover test ocular or systemic pathology likely to affect refractive development or successful contact lens wear birth weight ≤ 1250 g
Interventions	Group 1 (n = 21): 10 months wearing 2.00 D DF contact lens in the dominant eye and SVSCL in the contralateral eye, followed by 10 months wearing the swapped lens assignment Group 2 (n = 19): 10 months wearing DF contact lens in the nondominant eye and SVSCL in the contralateral eye, followed by 10 months wearing the swapped lens assignment
Outcomes	**Primary outcome:** Change in spherical equivalent refraction measured by cycloplegic autorefraction **Secondary outcome:** Change in AL measured by partial coherence interferometry Measurements taken at baseline and every 5 months for 20 months **Unit of analysis:** data analysed by dominant eye
Notes	Study dates: 2005 to not reported Trial registration: ACTRN12605000633684 Funding source: Maurice and Phyllis Paykel Trust; New Zealand Optometric and Vision Research Foundation; Cornea and Contact Lens Society of New Zealand Notes: study is also known as the Dual‐focus Inhibition of Myopia Evaluation in New Zealand (DIMENZ) study

**Table:** ATOM 2 Study 2012

***Study characteristics***	
Methods	Study design: parallel‐group RCT, with 2‐week run‐in period Study centre: 1 Number randomised: 400 children Study follow‐up: 2 years Exclusions and losses to follow‐up: 1 exclusion; 44 (11%) were lost to follow‐up
Participants	Age: mean = 9.7 years (range 6‐12 years) Gender: 211 boys, 189 girls Culture: Chinese (91%) in Singapore Inclusion criteria: age 6‐12 years myopia with SER error −2.00 D or worse in each eye as measured by cycloplegic autorefraction astigmatism not exceeding −1.50D myopic progression of ≥ 0.5 D in the past year distance vision correctable to logMAR 0.2 or better in both eyes normal ocular health other than myopia good general health with no history of cardiac or significant respiratory disease normal binocular function and stereopsis Exclusion criteria: ocular or systemic diseases that may affect vision or refractive error any ocular condition wherein topical atropine is contraindicated defective binocular function or stereopsis amblyopia or manifest strabismus including intermittent tropia previous or current use of atropine or pirenzepine
Interventions	0.01% atropine eyedrops (n = 84) 0.1% atropine eyedrops (n = 155) 0.5% atropine (n = 161)
Outcomes	**Primary outcome** Progression of myopia defined as the change in spherical equivalent refractive error from baseline and measured by cycloplegic autorefraction **Secondary outcomes** Change in axial length from baseline (Zeiss IOL Master) Ocular symptoms Changes in accommodative amplitude Photoptic and mesopic pupil sizes) Measurements taken at baseline and at 12 months and 24 months Note: baseline measurements recorded 2 weeks after treatment began to allow for stabilisation of the cycloplegic effect of atropine **Unit of analysis:** both eyes included in the analysis (Huber–White robust standard errors to allow for the correlation between eyes within person)
Notes	Study dates: not reported Trial registration: NCT00371124 Funding source: National Medical Research Council, Singapore and SingHealth

**Table:** ATOM Study 2006

***Study characteristics***	
Methods	Study design: parallel‐group RCT, with 2‐week run‐in period Study centre: 1 Number randomised: 400 children Study follow‐up: 2 years Exclusions and losses to follow‐up: no exclusions; 54 (13.5%) were lost to follow‐up
Participants	Age: mean = 9.2 years (range 6‐12 years) Gender: 220 boys, 180 girls Culture: Chinese (94%) and Indian children (4%) in Singapore Inclusion criteria: age 6‐12 years myopia with SER error between −1.00 D and −6.00 D in each eye as measured by cycloplegic autorefraction distance vision correctable to logMAR 0.2 or better in both eyes normal ocular health good general health with no history of cardiac or significant respiratory disease normal binocular function and stereopsis willingness and ability to tolerate monocular cycloplegia and mydriasis Exclusion criteria: astigmatism > −1.50 D by cycloplegic autorefraction IOP ≥ 21 mmHg allergies to atropine, cyclopentolate, proparacaine, or benzalkonium chloride previous or current use of contact lenses, BFs, PALs, or other forms of myopia treatment amblyopia or manifest strabismus, including intermittent tropia
Interventions	Atropine (n = 200): 1 eye was randomised to 1 drop of 1% atropine sulfate nightly; the other eye received nothing Placebo control (n = 200): 1 eye was randomised to 1 drop of vehicle nightly; the other eye received nothing Note: all children received single vision photochromatic lenses for correction of refractive errors
Outcomes	**Primary efficacy outcome** Progression of myopia defined as the change in SER error from baseline and measured by cycloplegic autorefraction **Secondary efficacy outcome** Change in AL from baseline and measured by A‐scan ultrasonography **Primary safety outcome** Occurrence of AEs **Secondary safety outcomes** BCVA, IOP, slit‐lamp biomicroscopy, fundus examination Measurements taken at baseline and annually for 2 years Note: baseline measurements recorded 2 weeks after treatment began to allow for stabilisation of the cycloplegic effect of atropine **Unit of analysis:** only 1 eye per child randomised to receive treatment (fellow eyes were controls)
Notes	Study dates: enrolment between April 1999 and September 2000 Trial registration: not reported Materials: vehicle drops were prepared by Alcon Laboratories; spectacles were SOLA Transitions SVLs Funding source: National Medical Research Council, Singapore Additional data: study author provided unpublished data via email correspondence

**Table:** Bao 2021

***Study characteristics***	
Methods	Study design: parallel‐group RCT Study centre: Eye Hospital of Wenzhou Medical University, Wenzhou, China Number randomised: 170 children Study follow‐up: 24 months Exclusions and losses to follow‐up: 9 (5%) were excluded or lost to follow‐up
Participants	Age: mean = 10.4 years (range 8‐13 years) Gender: 73 boys, 88 girls Culture: Chinese Inclusion criteria: cycloplegic SER between −0.75 D and −4.75 D astigmatism of cycloplegic autorefraction not exceeding 1.50 D anisometropia not exceeding 1.00 D based on SER monocular best corrected VA of 0.05 logMAR or better at distance for both eyes absence of ocular pathology absence of binocular vision issues and no history of ocular surgery or use of myopia control measures Exclusion criteria: history of PALs or BF use and no prior use of contact lenses strabismus by cover test at near and distance ocular or systemic medicine, which might affect myopia progression or VA through known effects on retina, accommodation or significant elevation of IOP
Interventions	HAL n = 58 SAL n = 57 SVL n = 55
Outcomes	**Primary outcomes** Change in SER error from baseline (cycloplegic autorefraction) Change in AL from baseline (Topcon KR‐800) **Secondary outcomes** Distance and near BCVA (ETDRS Chart) Time needed to adapt to the lenses Compliance (self‐reported daily wearing hours) AEs Measurements at 6‐monthly intervals for 24 months **Unit of analysis:** data from right eye analysed
Notes	Study dates: no dates provided Trial registration: ChiCTR1800017683 Funding source: International S&T Cooperation Program of China (grant number 2014DFA30940) and the collaborative research project with Essilor International (Wenzhou Medical University grant numbers 95013006 and 95016010). Disclosures: "Jinhua Bao is an Associate Director of Wenzhou Medical University–Essilor International Research Centre. Adeline Yang, Ee Woon Lim, Daniel P. Spiegel and Björn Drobe are employees of Essilor International."

**Table:** Bian 2020

***Study characteristics***	
Methods	Study design: parallel‐group RCT Study centre: Chengdu Aier Eye Hospital, China Number randomised: 200 children Study follow‐up: 12 months Exclusions and losses to follow‐up: not reported
Participants	Age: mean = 12.2 years (range 8‐14 years) Gender: 96 boys, 104 girls Culture: Chinese Inclusion criteria: spherical equivalent of −0.75 to −5.00D, ≤ 1.5 D with the rule astigmatism, ≤ 0.75 D against‐the‐rule astigmatism BCVA in either eye ≥ 1.0 No history of OK wear Exclusion criteria: ocular diseases such as strabismus, amblyopia, congenital cataract and optic nerve dysplasia history of eye surgery systematic disease, which can affect VA, such as diabetes and chromosome abnormality history of using contact lens, BF, MF lens or using atropine
Interventions	OK n = 100 SVLs n = 100
Outcomes	**Primary outcomes** Change in AL from baseline (Lenstar LS900) **Secondary outcomes** Change in central corneal thickness, anterior chamber depth and lens thickness Measurements at 6 months and 12 months **Unit of analysis:** data from 1 eye analysed
Notes	Study dates: January 2018‐ August 2018 Trial registration: not reported Funding source: not reported Disclosures: no declarations of interest reported

**Table:** BLINK Study 2020

***Study characteristics***	
Methods	Study design: parallel‐group RCT Study centre: University clinics in Houston Texas and Columbus Ohio, USA Number randomised: 294 children Study follow‐up: 36 months Exclusions and losses to follow‐up: 2 (0.7%) lost to follow‐up
Participants	Age: mean = 10.3 years (range 7‐11 years) Gender: 117 boys, 177 girls Culture: n = 200 (68%) white; n = 29 (10%) black; n = 25 (9%) Asian Inclusion criteria: SER −0.75 to −5.00D astigmatism < 1.00D vision correctable to 20/25 or better clinically acceptable fit with study contact lenses at baseline Exclusion criteria: > 1 month of gas permeable, soft BF, or OK contact lens wear > 1 month of myopia control (including atropine or BF spectacles) systemic issues that could affect myopia or myopia progression chronically using oral or ophthalmic steroids
Interventions	BF soft contact lenses (high add power (+2.50 D)) n = 98 BF soft contact lenses (med add power (+1.50 D) n = 98 SVSCL n = 98
Outcomes	**Primary outcome** Change in SER error from baseline (cycloplegic autorefraction) **Secondary outcomes** Change in AL from baseline (Haag‐Streit Lenstar LS 900) Association of peripheral defocus to myopic progression Ocular shape change at 36 months Adherence (parental report) AEs Measurements taken every 12 months for 36 months **Unit of analysis:** data from both eyes included (correlation between eyes adjusted in statistical model)
Notes	Study dates: enroled between 22 September 2014, and 20 June 2016. Follow‐up was completed on 24 June 2019. Trial registration: NCT02255474 Funding source: "This study was funded by grants from NIH granted to Drs Berntsen (U10 EY023204), Jordan (U10 023206), Walline (U10 023208), Mutti (U10 023210), Frishman (P30 EY007551), and Jackson (UL1 TR001070), and Bausch + Lomb provided contact lens solutions for the study" Disclosures: 7 authors declared support from Bausch and Lomb outside the submitted work

**Table:** Chamberlain 2019

***Study characteristics***	
Methods	Study design: parallel‐group RCT Study centre: university and hospital clinics in Canada, Portugal, Singapore and the UK Number randomised: 144 children Study follow‐up: 36 months Exclusions and losses to follow‐up: 40 (28%) were lost to follow‐up or withdrawn (includes 9 difficulty handling lenses/unacceptable fit and did not receive intervention)
Participants	Age: mean = 10.1 years (range 8 to < 13 years) Gender: 75 boys, 69 girls Culture: 79 (55%) white European, 34 (24%) East Asian, 12 (8%) West Asian, 13 (9%) mixed, 6 (4%) other Inclusion criteria: children with SER error between −0.75 and −4.00 D inclusive with < 1.00 D of astigmatism or anisometropia Exclusion criteria: current or prior contact lens wear current or prior use of any other myopia control intervention use of medications that could affect contact lens wear
Interventions	Dual focus soft contact lens (MiSight) (n = 70) SVSCLs (n = 74)
Outcomes	**Primary outcomes** Change in SER error from baseline (cycloplegic autorefraction) Change in ALfrom baseline (Zeiss IOL master) **Secondary outcomes** Number of particpants with biomicroscopic findings > grade 2 Ocular AE rate between groups Measurements taken at 12, 24 and 36 months **Unit of analysis:** data from both eyes included (correlation between eyes adjusted in statistical model)
Notes	Study dates: recruitment between November 2012 and April 2014 Trial registration: NCT01729208 Funding source: the study was sponsored by Coopervision Inc Disclosures: "PC is an employee of Coopervision"

**Table:** Charm 2013

***Study characteristics***	
Methods	Study design: parallel‐group RCT Study centre: 1 (Hong Kong Polytechnic University) Number randomised: 52 children Study follow‐up: 2 years Exclusions and losses to follow‐up: 14 (27%) children who were randomised, 7 in each group, were excluded or lost to follow‐up
Participants	Age: median = 10 years (range 8‐11 years) Gender: not reported Culture: children "recruited via advertisements posted on local newspapers and leaflets in the Optometry Clinic of the School of Optometry" Inclusion criteria: aged 8‐11 years myopia with SER error ≥ −5.00 D by cycloplegic manifest refraction monocular Snellen VA 20/25 or better willingness to wear OK and to be available for monthly follow‐up Exclusion criteria: astigmatism > 1.25 D binocular vision problems any ocular or systemic condition that may affect vision or vision development contraindications for contact lens wear previous experience with refractive surgery, PALs, or OK
Interventions	OK (n = 26): partial reduction OK contact lenses of target 4.00 D (DreamLite, Procornea Ltd, The Netherlands); "residual refractive errors were corrected by a pair of single vision spectacles to be worn during daytime" SVLs (n = 26) Note: "spectacle prescription would be updated at any subsequent visit for either group of subjects if difference in residual refractive errors (sphere or astigmatism) obtained at that visit exceeded 0.50 D"
Outcomes	**Primary outcome** Change in AL **Secondary outcomes** Objective and subjective cycloplegic refraction Fundus examination VA Slit‐lamp examination Corneal topography Measurements taken every 6 months for 2 years **Unit of analysis:** child‐based (right eye)
Notes	Study dates: not reported Trial registration: NCT00977236 Funding source: "this study was supported by a Collaborative Research Agreement between The Hong Kong Polytechnic University (PolyU) and Procornea Nederland B.V. and a Niche Area Funding (J‐BB7P) from PolyU. We thank Menicon Company Limited for supplying Menicon O2 Care for the study" Conflict of interest: "the authors have no proprietary interest in any of the products used in the study"

**Table:** Cheng 2010

***Study characteristics***	
Methods	Study design: parallel‐group RCT Study centre: 1 (optometric practice in Mississauga, Ontario, Canada) Number randomised: 150 children Study follow‐up: 2 years Exclusions and losses to follow‐up: 15 (10%) children who were randomised were excluded from the analyses; 4 (3%) were lost to follow‐up
Participants	Age: mean = 10 years (range 8‐13 years) Gender: 62 boys and 73 girls received treatment Culture: Chinese Canadian children were recruited by reviewing clinical records and mailing invitation letters addressed to their parents, or by responding to poster in the practice or during regular eye examinations Inclusion criteria: Chinese Canadian children who were seen at the practice in the last 9‐18 months age 8‐13 years myopia between −1.00 D and −5.50 D myopia progression ≥ 0.50 D in the preceding year distance monocular VA of 6/6 or better near monocular VA of 6/6 or better stereoacuity ≤ 40 s of arc at 40 cm single vision distance lens wear consent of child and parent for study participation Exclusion criteria: astigmatism > 1.50 D anisometropia > 1.50 D strabismus inability to respond to subjective testing history of systemic or ocular disease history of BF lens wear and/or contact lens use
Interventions	SVLs (n = 50): single vision distance lenses BF lenses (n = 50): BF lenses with +1.50 D near addition Prismatic BF lenses (n = 50): prismatic BF lenses with +1.50 D addition and a 3‐prism diopter base‐in prism in the near segment Note: distance prescription changes were made if subjective refraction changed by ≥ 0.50 D in either eye
Outcomes	**Primary outcome** Myopic progression defined as difference between the mean cycloplegic spherical equivalent measured by an automated refractor at the baseline visit and subsequent 6‐month visits for 24 months **Secondary outcome** Eye growth defined as difference between mean ALs measured by ultrasonography at the baseline visit and at subsequent 6‐month visits for 24 months Measurements taken at baseline and every 6 months for 2 years **Unit of analysis:** child‐based (right eye)
Notes	Study dates: April 2003‐April 2008 Trial registration: NCT00787579 Funding source: Essilor International of France Auxiliary data: "Parents and/or guardians completed questionnaires related to vision habits of the enroled child and the child's birth parents' refractive errors. The number of years the children were myopic before entering the study was estimated from clinical records. Auxiliary data were used as covariates for regression statistics and to test the hypothesis that bifocal treatment is more effective with a shorter duration of myopia" Additional data: study author provided unpublished data via email correspondence

**Table:** Cheng 2016

***Study characteristics***	
Methods	Study design: parallel‐group RCT Study centre: Korb and Associates in Boston, Massachusetts, USA Number randomised: 127 children Study follow‐up: 12 months (planned for 24 months) Exclusions and losses to follow‐up: 6 (4.7%) children who were randomised were excluded from the analyses; 15 (11.8%) were lost to follow‐up
Participants	Age: mean = 9.7 years (range 8‐11 years) Gender: 59 boys, 68 girls Culture: 90.6% were Asian and 8.7% were white Inclusion criteria: aged 8‐11 years myopia −0.75 to −4.00 D sphere by cycloplegic refraction ≤ 1.00 D astigmatism ≤ 1.00 D difference between eyes in spherical equivalent 20/25 + 2 or better VA in each eye with spherocylindrical refraction 20/25 or better VA with best sphere Exclusion criteria: ocular or systemic pathology history of eye surgery history of myopia control
Interventions	Soft contact lens + SAL group (n = 64): soft daily disposable contact lenses with positive spherical aberration (0.175 μm) Soft contact lens group (n = 63): soft daily disposable contact lenses without the positive spherical aberration Note: control and test lenses had identical material and appearance; spherical aberration was chosen to negate the negative spherical aberration that occurred in myopes during accommodation
Outcomes	**Primary outcome** Change in spherical equivalent cycloplegic autorefraction **Secondary outcome** Change in AL Measurements taken every 6 months for 2 years **Unit of analysis:** child‐based (right eye)
Notes	Study dates: April 2008‐October 2011 Trial registration: NCT01829230 Funding source: Johnson and Johnson Vision Care, Inc. Disclosures of interest: "Xu Cheng, Jing Xu, Khaled Chehab, and Noel Brennan are all paid employees of Johnson and Johnson Vision Care, Inc. Joan Exford of Korb & Associates is a contract principal investigator paid by Johnson and Johnson Vision Care, Inc."; "We thank Dr. Jichang He of New England College of Optometry and Dr. Victor Finnemore of Korb & Associates for collecting data for the study and Dr. Myles Jaffe of Innova Medical Communications, LLC, who is a contract medical writer paid by Johnson and Johnson Vision Care, Inc. for preparing this manuscript" Notes: "the study was terminated because sufficient data had been collected from concurrent internal studies of similar designs"

**Table:** Chung 2002

***Study characteristics***	
Methods	Study design: parallel‐group RCT Study centre: patient care unit at the Department of Optometry, Faculty of Allied Health Science, National University of Malaysia Number randomized: 106 children Study follow‐up: 2 years Exclusions and losses to follow‐up: no exclusions; 12 (11%) were lost to follow‐up
Participants	Age: mean = 11.56 years (range 9‐14 years) Gender: 39 boys, 55 girls Culture: Malay and Chinese ethnic origin Inclusion criteria: age 9‐14 years myopia with SER error ≥ −0.50 D in both eyes, with no principal meridian being plano or having any amount of plus power corrected VA of 6/6 or better in each eye normal ocular health willingness to give written consent Exclusion criteria: > 2 D of astigmatism in each eye binocular vision problems, including anisometropia > 2.00 D, problems requiring refractive therapy, strabismus, and amblyopia previous contact lens wear family was planning to leave the area before the end of the study period
Interventions	Undercorrected group (n = 47): monocular VA blurred to 6/12 (approximately +0.75 D) in each eye with spectacles Fully corrected group (n = 47): monocular VA maintained at 6/6 or better in each eye with spectacles Note: in the fully corrected group, changes in prescription were made if subjective refraction had changed by ≥ 0.50 D for 1 or both eyes. For the undercorrected group, changes in prescription were made to maintain a vision of 6/12 in each eye
Outcomes	**Progression of early‐onset myopia** Static retinoscopy without cycloplegia Keratometry Subjective cycloplegic refractions using the endpoint of maximum plus or minimum plus for best acuity Ocular components measurements by means of A‐scan ultrasonography Measurements taken at baseline and every 6 months for 2 years **Unit of analysis:** average values of both eyes used for all results
Notes	Study dates: not reported Trial registration: not reported Funding source: IRPA grant Compliance in wearing glasses was monitored via questionnaires. Compliance was defined as wearing glasses for at least 8 h/day (40 children in the undercorrected group vs 41 in the fully corrected group). Partial compliance was defined as wearing glasses 6‐8 h/day (7 children in the undercorrected group vs 6 in the fully corrected group)

**Table:** CLAMP Study 2004

***Study characteristics***	
Methods	Study design: parallel‐group RCT, with run‐in period Study centre: 1 (the Ohio State University College of Optometry, USA) Number randomised: 116 children Study follow‐up: 3 years Exclusions and losses to follow‐up: none
Participants	Age: mean = 10.7 years (range 8‐12 years) Gender: 47 boys, 69 girls Culture: Columbus, Ohio, USA; 84.5% white (not of Hispanic origin), 8.6% Asian or Pacific Islander, 4.3% black (not of Hispanic origin) Inclusion criteria: 8‐11 years old at time of randomisation myopia with SER error between −0.75 D and −4.00 D in each eye, as measured by cycloplegic refraction corrected VA of 20/20 or better in each eye Exclusion criteria: astigmatism > 1.50 DC in each eye by cycloplegic refraction or > 1.00 DC on manifest refraction previous or attempted history of contact lens wear anisometropia > 1.00 D between eyes eye disease and binocular vision problems systemic disease that may affect vision or vision development Note: all participants had to successfully complete a run‐in period before enrolment into the study to exclude those who could not adapt to rigid contact lenses; 32 children did not complete the run‐in period and were excluded. Success for the run‐in period was defined as wearing the lenses at least 40 h/week and stating that the lenses were "always comfortable" or "usually comfortable"
Interventions	(n = 59): RGPs worn during waking hours for 3 years (n = 57): soft contact lenses worn during waking hours for 3 years Note: prescription changes were made by an unmasked examiner based on participant complaints and improvement in VA
Outcomes	**Primary outcome** Change in cycloplegic autorefraction during 3 years (spherical equivalent) **Secondary outcomes** Change in AL Change in peripheral autorefraction Change in crystalline lens curvatures Change in corneal curvature and thickness Change in accommodation Change in IOP Measurements taken at baseline and every 6 months for 3 years **Unit of analysis:** data analysed for right eye only
Notes	Study dates: enrolment 9 July 1998 to 26 February 2000 Trial registration: NCT00009529 Funding source: National Eye Institute, National Institutes of Health; Menicon Co, Ltd.; CIBA Vision Corporation; SOLA Optical; and Essilor

**Table:** COMET2 Study 2011

***Study characteristics***	
Methods	Study design: parallel‐group RCT Study centres: 8 (including 7 optometry colleges and schools and 1 community‐based ophthalmology practice) Number randomised: 118 children Study follow‐up: 3 years Exclusions and losses to follow‐up: no exclusions; 8 (7%) were lost to follow‐up
Participants	Age: mean = 10.1 years (range 8‐12 years) Gender: 54 boys, 64 girls Culture: USA Inclusion criteria: age 8 to < 12 years refractive error determined by cycloplegic autorefraction, which meets all of the following: spherical equivalent −0.50 to −3.00 D in both eyes; astigmatism ≤ 1.5 D in both eyes; anisometropia ≤ 1.00 D difference between eyes in spherical equivalent VA at least 20/20 with best subjective refraction in both eyes accommodative response at near vision (33 cm) is < 2.0 D by noncycloplegic autorefraction near esophoria (≥ 2.0 pupillary distance) present by alternate prism and cover test (APCT) at near vision using best refractive correction determined from noncycloplegic subjective refraction Exclusion criteria: history of strabismus current or prior use of PALs, BFs, or contact lenses in either eye (prior or current use of SVLs was permitted)
Interventions	PAL group (n = 59): Varilux Ellipse PALs with a +2.00 D near addition; worn during all waking hours for 3 years SVL group (n = 59): standard SVLs (spectacles); worn during all waking hours for 3 years Notes: "The distance correction was changed if the endpoint of the noncycloplegic subjective refraction differed from the current prescription by 0.50 D or more in spherical equivalent. Prescription changes could be made for smaller differences at investigator discretion if the new prescription improved the patient’s visual acuity by at least 1 line over that in their current correction"
Outcomes	**Primary outcome** Change in SER error in D from baseline to 3‐year visit measured by cycloplegic autorefraction **Secondary outcomes** Main axis astigmatism (*J*₀, dioptric power of a Jackson cross‐cylinder with axis at 0°) and oblique astigmatism (*J*₄₅, dioptric power of a Jackson cross‐cylinder with axis at 45°) by using the power vector approach Measurements taken at baseline and every 6 months for 3 years **Unit of analysis:** child‐based (median for each eye averaged to obtain the spherical equivalent used for analysis)
Notes	Study dates: enrolment from April 2005‐March 2007 Trial registration: NCT00320593 Funding source: National Institutes of Health, Department of Health and Human Services, USA Materials: Essilor of America and Eyewear Designs provided spectacles at a reduced cost Study name: Progressive addition lenses vs single vision lenses for slowing progression of myopia in children with high accommodative lag and near esophoria

**Table:** COMET Study 2003

***Study characteristics***	
Methods	Study design: parallel‐group RCT Study centre: multicentre, including a study chair a co‐ordinating centre 4 clinical centres the National Eye Institute, USA Number randomised: 469 children Study follow‐up: 3 years Exclusions and losses to follow‐up: no exclusions; 7 (1.5%) were lost to follow‐up
Participants	Age: mean = 9.3 years (range 6‐11 years) Gender: 223 boys, 246 girls Culture: 4 major cities in the USA (Birmingham, Alabama: n = 133; Boston, Massachusetts: n = 110; Philadelphia, Pennsylvania: n = 108; and Houston, Texas: n = 118) Inclusion criteria: 6‐11 years old myopia with SER error between −1.25 D and −4.50 D in both eyes, as measured by cycloplegic autorefraction astigmatism ≤ 1.50 D no anisometropia (difference in spherical equivalent < 1.00 D between eyes) BCVA of 20/32 or better no strabismus by cover test for far (4.0 m) and/or near (0.33 m) fixation willingness to not wear contact lenses for study duration Exclusion criteria: strabismus detected by cover test any ocular, systemic, or neurodevelopmental conditions that could influence refractive development chronic medication use that might affect myopia progression or VA birth weight < 1250 g previous use of BFs, PALs, or contact lenses problems with adherence to the protocol or the follow‐up period
Interventions	PAL group (n = 235): MF lenses (no‐line BFs) with gradual and progressive change toward less negative or more positive power from the distance portion to the near portion of the lens (power +2.00 D); worn during waking hours for 3 years SVL (n = 234): SVLs with same focal power throughout the lens area; worn during waking hours for 3 years Note: "Prescription changes were made if the subjective refraction had changed by at least 0.50 D for 1 or both eyes. Smaller prescription changes were made if clinically indicated. Both groups were offered single vision sports glasses to use while participating in sports activities"
Outcomes	**Primary outcome** Change in refractive error Magnitude of change in SER error relative to baseline measured by cycloplegic autorefraction with 2 drops of 1% tropicamide **Secondary outcomes** AL (magnitude of change in AL relative to baseline using average 3‐5 measurements with the Sonomed A‐scan) Changes in ocular components, including lens thickness, anterior chamber depth, vitreous chamber depth Accommodation and phoria by Maddox rod Corneal curvature based on keratometry measured with the autorefractor Normal reading distance for standardised age‐appropriate text Measurements taken at baseline and every 6 months for 3 years **Unit of analysis:** child‐based Average values of both eyes used if the correlation coefficient was > 0.85 between eyes and the mean difference was not statistically significant; otherwise the eye with greater myopic change used for each child
Notes	Study dates: enrolment was from September 1997‐September 1998; follow‐up was designed for 3 years but continued for 7 years, including 5 years wearing original lens assignments and 2 years wearing either glasses or contact lenses Trial registration: NCT00000113 Funding source: NEI grants, Essilor of America, Marchon Eyewear, Marco Technologies, and Welch Allyn Sample of 150 children were followed up at 1 month to evaluate possible lens‐induced phoria changes; no problems were detected in either group Compliance in wearing glasses was monitored via separate questionnaires for children and parents (93% compliance in PAL group, 96% compliance in SVL group). Attitude toward wearing glasses and self‐esteem were also measured Additional data: study author provided unpublished data via email correspondence

**Table:** CONTROL Study 2016

***Study characteristics***	
Methods	Study design: parallel‐group RCT Study centre: 1 Number randomised: 86 children Study follow‐up: 1 year Exclusions and losses to follow‐up: 8 children did not complete the study
Participants	Age: mean = 13 years (range 8‐18 years) Gender: 26 boys, 60 girls Culture: California, USA Inclusion criteria: myopia between −0.50 D and −6.00 D, with documented progression of ≥ −0.50 D since last examination eso fixation disparity at 33 cm with distance correction astigmatism ≤ 1.00 D anisometropia ≤ 2.00 D BCVA 20/20 or better in each eye ability to wear SCLs and attend follow‐up visits Exclusion criteria: presence of ocular disease affecting eye growth or preventing wear of contacts prior ocular surgery history of wearing RGPs in previous 2 years or extended wear SCLs in previous 6 months pregnancy or nursing use of certain medications
Interventions	BFSCL group (n = 39): Vistakon Acuvue Bifocal lenses (distance centre, alternating 5‐ring), worn on a daily basis SVSCL group (n = 40): Vistakon Acuvue 2, worn on a daily basis
Outcomes	**Primary outcomes** Changes in cycloplegic autorefraction at 1 year Changes in cycloplegic subjective refraction at 1 year Changes in AL at 1 year **Secondary outcomes** Keratometric changes at 1 year Changes in manifest refraction at 1 year Relationship between residual fixation disparity and myopia progression Measurements taken at baseline, 6 months, and 12 months **Unit of analysis:** average values for both eyes
Notes	Study dates: start date was October 2003; study was completed in 2006 Trial registration: NCT00214487 Funding source: Vistakon Additional information: study author provided unpublished information via email correspondence

**Table:** Cui 2021

***Study characteristics***	
Methods	Study design: parallel‐group RCT Study centre: Hospital of Zhengzhou University, China Number randomised: 400 children Study follow‐up: 24 months Exclusions and losses to follow‐up: 100 (25%) were lost to follow‐up by 24 months
Participants	Age: mean = 9.4 years (range 6‐14 years) Gender: 210 boys, 190 girls Culture: Chinese Inclusion criteria: aged 6–14 years myopic SER of −1.25 to −6.00 D in both eyes astigmatism of < 2.0 D anisometropia of < 1.0 D monocular BCVA of 16/20 or better IOP 10‐21 mmHg no other eye diseases or surgery Exclusion criteria: previously used atropine, pirenzepine, or RGP or ortho‐K lenses or MF contact lens to control myopia progression
Interventions	0.02% atropine eyedrops (n = 138) 0.01% atropine eyedrops (n = 142) SVLs (n = 120) (this was a non‐randomised comparison group)
Outcomes	**Primary outcomes** AL (IOLMaster; Carl Zeiss Meditec AG, Germany) Corneal power (IOLMaster; Carl Zeiss Meditec AG, Germany) **Secondary outcomes** Anterior chamber depth (IOLMaster; Carl Zeiss Meditec AG, Germany) Pupil diameter (NIDEK, AR‐1, Japan) Accommodation amplitude (Push‐up technique) Cycloplegic autorefraction (Topcon RM 8000A, CA) Incidence of AEs Measuremnents taken at 4‐monthly intervals for 24 months **Unit of analysis:** child‐based (right eye)
Notes	Study dates: January 2018‐August 2020 Trial registration: ChiCTR‐IPD‐16008844 Funding source: "Funding was provided by Medical Science and Technology Research Projects of Henan Province Health Commission (Grant No. 201602073), Key Research and Promotion Special Projects of Henan Provincial Science and Technology Department (Grant No. 201801591), Key School Research Projects of Henan Provincial Department of Education (Grant No. 19A320066), Health and Family Planning Science and Technology Talents Overseas Training Project of Henan Province (Grant No. 2018038)." Disclosures: "The authors declare no competing interests."

**Table:** DISC Study 2011

***Study characteristics***	
Methods	Study design: parallel‐group RCT Study centre: 1 (Hong Kong Polytechnic University) Number randomised: 221 children Study follow‐up: 2 years Exclusions and losses to follow‐up: 38 (34.2 %) in BFSCL group and 36 (32.7%) in SVSCL group were excluded; 8 (7.2 %) in BFSCL group and 11 (10.0%) in SVSCL group were lost to follow‐up
Participants	Age: mean = 11 years (range 8‐13 years) Gender: 85 boys, 136 girls Culture: Hong Kong, China Inclusion criteria: age 8‐13 years spherical equivalent −1.00 to −5.00 D astigmatism ≤ 1.00 D anisometropia ≤ 1.25 D spectacle‐corrected monocular VA 0.0 logMAR or better contact lens–corrected monocular VA 0.1 logMAR or better willingness to wear contact lenses regularly and parents' understanding and acceptance of random allocation of intervention Exclusion criteria: ocular or systemic abnormalities affecting visual function or refractive development prior use of PALs or BF contact lenses contraindication for contact lens wear
Interventions	BFSCL group (n = 111): dual‐focus incorporated soft contact (DISC) lenses, which were custom‐made BFSCLs with distance correction in the centre and alternating rings of defocusing (+2.50 D addition) and distance correction zones SVSCL group (n = 110): SVSCLs Note: children were instructed to wear lenses for 5‐10 h/day and to wear spectacles with full prescription when not wearing contact lenses
Outcomes	**Primary outcomes** Refractive error (cycloplegic autorefraction) AL **Secondary outcome** Corneal curvature Measurements taken every 6 months over 2 years **Unit of analysis:** individual (right eye used for analysis)
Notes	Study dates: September 2007‐ October 2009 Trial registration: NCT00919334 Funding source: "the study was supported by grants of RGC GRF (B‐Q04G) and Niche Areas Fund (J‐BB7P) from The Hong Kong Polytechnic University" Conflict of interest: reported "none"

**Table:** Edwards 2002

***Study characteristics***	
Methods	Study design: parallel‐group RCT Study centre: 1 (Centre for Myopia Research, Hong Kong) Number randomised: 298 children Study follow‐up: 2 years Exclusions and losses to follow‐up: no exclusions; 44 (15%) were lost to follow‐up
Participants	Age: mean = 9.09 years (range 7‐10.5 years) Gender: 122 boys, 132 girls Culture: Hong Kong children, recruited through newspaper advertisements Inclusion criteria: 7‐10.5 years old SER error between −1.25 D and −4.50 D, as measured under cycloplegia BCVA of 0.00 logMAR or better no previous use of contact lenses and willingness to not wear contact lenses willingness to wear glasses constantly parents' acceptance of randomisation Exclusion criteria: astigmatism > 1.50 D anisometropia > 1.50 D in spherical or cylindrical error any ocular or systemic condition that might affect refractive development previous use of BFs or PALs problems with adherence to the protocol or the follow‐up period
Interventions	PAL group (n = 138): SOLA MC PALs (add +1.50 D); worn constantly for 2 years SVL (n = 160): SOLA SVLs; worn constantly for 2 years Note: prescription changes were made if there was a reduction in aided vision of ≥ 0.10 logMAR units
Outcomes	**Primary outcomes** Refractive error measured under cycloplegia (by autorefraction for data analysis and by subjective refraction for spectacle prescription) AL measured under cycloplegia **Secondary outcomes** Aided visual acuity in each eye Mean monocular and binocular distance and near PD Noncycloplegic refraction Horizontal and vertical heterophoria Normal reading distance for standardised age‐appropriate text Measurements taken at baseline and every 6 months for 2 years **Unit of analysis:** only data from right eyes reported
Notes	Study dates: not reported Trial registration: not reported Materials: lenses provided by Sola (Hong Kong) Ltd Funding source: Centre for Myopia Research (Area of Strategic Development), The Hong Kong Polytechnic University

**Table:** Fujikado 2014

***Study characteristics***	
Methods	Study design: cross‐over RCT Study centre: 1 (Osaka University School of Medicine), Japan Number randomised: 24 children Study follow‐up: 12 months for each phase Exclusions and losses to follow‐up: "in the second year, two children dropped out from the study because their families moved to another city"
Participants	Age: mean = 14 years (range 6‐16 years) Gender: 7 boys, 17 girls Culture: Japan Inclusion criteria: 6‐16 years of age myopic refractive error between −0.75 D and −3.50 D anisometropia ≤ 1.0 D astigmatism ≤ 1.0 D BCVA 20/20 or better willingness to wear lenses Exclusion criteria: amblyopia, strabismus, or other ocular disease other than refractive error history of OK, BF spectacles, or PALs in past 12 months
Interventions	BFSCL group (n = 11 in phase 1): progressive addition soft contact lenses (+0.50 D) with 8.6 mm base curve, 14.5 mm diameter, 3.25 mm central zone, and horizontal thick zones to prevent rotation (Mipafilcon A; Menicon, Nagoya, Japan) SVSCL group (n = 13 in phase 1): SVSCLs
Outcomes	**Primary outcomes** AL Spherical equivalent at 12 and 24 months (cycloplegic autorefraction) **Secondary outcomes** Peripheral refraction Compliance Measurements taken months 1, 3, 6, 9, and 12 in each phase **Unit of analysis:** individual (average of both eyes except for 1 child whose right eye only was enroled)
Notes	Study dates: January 2011‐March 2013 Trial registration: JPRN‐UMIN000007989 Funding sources: Menicon Corp., Itami Central Ophthalmology Clinic (Japan) Conflict of interest: "AS and MN are employees of Menicon. The authors report no other conflicts of interest in this work"

**Table:** Fulk 1996

***Study characteristics***	
Methods	Study design: parallel‐group RCT Study centre: 1 (Indian Health Service Hospital, Optometry Department, Tahlequah, Oklahoma, USA) Number randomised: 32 children Study follow‐up: 18 months Exclusions and losses to follow‐up: no exclusions; 4 (12.5%) were lost to follow‐up
Participants	Age: range 6‐13 years Gender: included boys and girls (numbers not reported) Culture: children with myopia and near point esophoria identified from medical records and referred by local optometrists Inclusion criteria: at least 0.50 D of myopia in both principal meridians of both eyes ages 6‐13.99 years for boys and 6‐12.99 years for girls near point esophoria corrected acuity of at least 20/25 in each eye, distance and near, with SVLs ability to respond to subjective tests Exclusion criteria: strabismus astigmatism > 2.00 D in either eye anisometropia> 2 D convergence insufficiency accompanied by symptoms diabetes or other systemic disease with potential effects on refractive error ocular disease other than mild inflammation of the adnexa
Interventions	BFs (n = 16): BFs with +1.25 D addition SVLs (n = 16): SVLs Note: prescription changes were made if the spherical equivalent in either eye had changed by 0.50 D
Outcomes	**Primary outcomes** Change in refractive error (SER) measured by cycloplegic autorefraction Change in AL measured by Humphrey A/B Scan under cycloplegia Measurements taken at baseline and every 6 months for 18 months **Unit of analysis:** average values of both eyes
Notes	Study dates: not reported Trial registration: not reported Funding source: Northeastern State University Faculty Research Committee (Tahlequah, Oklahoma, USA)

**Table:** Fulk 2002

***Study characteristics***	
Methods	Study design: parallel‐group RCT and study of variables that may influence myopia progression in children Study centre: 2 (Tahlequah and Tulsa, Oklahoma, USA) Number randomised: 82 children Study follow‐up: 30 months Exclusions and losses to follow‐up: no exclusions; 7 (8.5%) were lost to follow‐up
Participants	Age: mean = 10.7 years (range 6‐12 years) Gender: 43 boys, 39 girls Culture: children with myopia and near point esophoria recruited locally and through clinics operated by the Cherokee Nation: 58% white, 29% Native American, 5% Hispanic, 4% African American, 3% other, 1% Asian/Pacific Islander Inclusion criteria: at least 0.50 D of myopia in both principal meridians of both eyes ages 6‐12.99 years for boys and 6‐11.99 years for girls near point esophoria corrected VA of at least 20/25 in each eye at distance and binocularly with SVLs corrected stereoacuity of at least 40 s arc with SVLs at 40 cm assent of child and consent to participate Exclusion criteria: strabismus astigmatism or anisometropia > 2.00 D diabetes or other systemic disease with potential effects on refractive error ocular disease other than mild inflammation of the adnexa known history of allergic reaction to proparacaine or tropicamide history of use of RGPs current use of bifocals or use within the last year high myopia of ≥ −6.00 D for children < 9 years or ≥ −8.00 D for children ≥ 9 years inability to respond to subjective testing or hold fixation sufficiently to allow for study measurements
Interventions	BFs (n = 42): BF lenses with +1.50 D add SVLs (n = 40) Note: prescription changes were made if (1) the spherical equivalent in either eye had changed by 0.50 D, or (2) any combination of sphere or cylinder change could improve the distance acuity by ≥ 3 letters in either eye
Outcomes	**Primary outcome** Change in refractive error (SER) (cycloplegic autorefraction) **Secondary outcomes** Change in AL (A‐scan ultrasonography) Change in vitreous chamber depth (A‐scan ultrasonography) Changes in cylinder component (J₀ and J₄₅) Variables associated with myopia progression: parental myopia, season, near point habits, and academic achievement Measurements taken at baseline and every 6 months for 30 months **Unit of analysis:** average values of both eyes
Notes	Study dates: enrolment 20 August‐15 October 15 1996; original follow‐up was for 30 months; some children remained for 54 months Trial registration: NCT00000128 Funding source: National Eye Institute, National Institutes of Health Notes: study was also known as the Myopia Progression Study

**Table:** Garcia‐del Valle 2021

***Study characteristics***	
Methods	Study design: parallel‐group RCT Study centre: 7 university and hospital clinics in Spain: Madrid (n = 3), Andalucía (n = 3), and Murcia (n = 1) Number randomised: 70 children Study follow‐up: 12 months Exclusions and losses to follow‐up: 12 (21%) were lost to follow‐up
Participants	Age: mean = 12.1 years (range 7‐15 years) Gender: 21 boys, 37 girls Culture: European (Spanish) Inclusion criteria: children aged 7‐15 SER −0.50 to −8.75 BCVA = 1.0 (20/20 good ocular and general health able to handle and wear contact lenses Exclusion criteria: uncontrolled psychiatric or neurological disorders and manifest disability due to age physical or mental conditions to wear contact lenses
Interventions	MFSCLs (n = 36) SVSCLs (n = 34)
Outcomes	**Primary outcomes** Change in SER error from baseline (cycloplegic autorefraction) Change in AL from baseline (Zeiss IOL Master 700) **Secondary outcomes** Proportion of participants reporting good comfort and good quality of vision Frequency of ocular AEs Measurements taken at 12 months **Unit of analysis:** data from both eyes included (correlation between eyes adjusted in statistical model)
Notes	Study dates: May 2014‐April 2017 Trial registration: not reported Funding source: "Tiedra Farmacéutica S.L. was the sponsor for this study. Tiedra Farmacéutica S.L. is the owner of the patent for Esencia design and provided the study contact lenses and maintenance solutions" Disclosures: not reported

**Table:** Guo 2021

***Study characteristics***	
Methods	Study design: parallel‐group RCT Study centre: Optometry Clinic of The Hong Kong Polytechnic University Number randomised: 82 children Study follow‐up: 12 months Exclusions and losses to follow‐up: 24 (30%) were excluded or lost to follow‐up
Participants	Age: mean = 9.2 years (range 6 to <11 years) Gender: 28 boys, 42 girls Culture: Chinese Inclusion criteria: age 6 to <11 years Chinese ethnicity (both parents) myopia between −4.00 D to −0.75 D; astigmatism; axes 180 30: ≥ −2.50 D; other axes: ≥ −0.50 D; < 1.00 D difference in spherical equivalent between the two eyes BCVA logMAR 0.10 or better in both eyes symmetrical corneal topography with corneal toricity < 2.00 D in either eye normal ocular health other than myopia Exclusion criteria: history of myopia control treatment strabismus or amblyopia systemic condition, which might affect refractive development contraindications to contact lens wear history of ocular inflammation or infection corneal dystrophy
Interventions	OK lenses of BOZD 6 mm (n = 42) OK lenses of BOZD 5 mm (n = 40)
Outcomes	**Primary outcomes** Change in AL from baseline (Zeiss IOL Master 500) **Secondary outcomes** Change in cycloplegic refraction Change in BCVA Measurements taken at 6 and 12 months **Unit of analysis:** data from right eye analysed
Notes	Study dates: June 2017‐March 2021 Trial registration: NCT03191942 Funding source: The Hong Kong Polytechnic University Research Residency Scheme of the School of Optometry Disclosures: "R Kojima is a Clinical Research and Development Director for Precision Technology Services (Vancouver, Canada), a partner in the KATT Design Group (Vancouver, Canada) and a clinical advisor to Medmont International Pty, (Nunawading, Australia)"

**Table:** Han 2018

***Study characteristics***	
Methods	Study design: parallel‐group RCT Study centre: 1 (Affiliated Yixing People Hospital of Jiangsu University) Number randomised: 240 children Study follow‐up: 1 year Exclusions and losses to follow‐up: none
Participants	Age: mean = 9.8 years (range 9‐14 years) Gender: 117 boys, 123 girls Culture: China Inclusion criteria: children with myopia treated in the study authors’ hospital Exclusion criteria: not reported
Interventions	Ordinary frame glasses (n = 90) M‐OK lenses (n = 90): Mouldway OK lenses; described as “four‐district seven‐arc reverse geometric design. The main component is Boston XO (Bausch + Lomb, USA [Hexafocon A, main component fluorosiliconepropenylphenol ester]) and the standard piece was the Mouldway IV‐DF type” Medcall lenses (n = 60): “fitted with a new paracentral defocus‐reducing lens” Note: none
Outcomes	**Primary outcome** Outcomes not clearly specified as primary or secondary. Outcomes reported included “diopter, accommodative lag, and accommodative facility” **Secondary outcome** Not reported Measurements taken at 1 year **Unit of analysis:** individual (1 eye per person enroled)
Notes	Study dates: May 2013‐May 2015 Trial registration: not reported Funding source: “the authors have no funding or conflicts of interest to disclose”

**Table:** Han 2019

***Study characteristics***	
Methods	Study design: parallel‐group RCT Study centre: Shanghai Tongji Hospital, China Number randomised: 150 children Study follow‐up: 24 months Exclusions and losses to follow‐up: 16 (11%) were lost to follow‐up by 24 months
Participants	Age: mean = 9.4 years (range 6‐12 years) Gender: 75 boys, 75 girls Culture: Chinese Inclusion criteria: aged 6‐12 years myopia −0.25 D to −6.00 D no ocular and underlying diseases Exclusion criteria: anisometropia amblyopia allergy or intolerance to the use of anticholinergic drops
Interventions	1% atropine eyedrops (n = 60) Combined treatment (0.5% racanisodamine eye drops and 1% atropine eyedrops) (n = 60) No treatment (n = 30)
Outcomes	**Primary outcomes** SER Corneal curvature AL IOP and AEs Measurements taken at 6‐monthly intervals for 24 months **Unit of analysis:** not reported
Notes	Study dates: July 2013‐June 2014 Trial registration: not reported Funding source: Shanghai Municipal Commision of Health and Family Planning (General program) (201540252) Disclosures: not reported

**Table:** Hasebe 2008

***Study characteristics***	
Methods	Study design: cross‐over RCT Study centre: 1 (Okayama University Medical School) Number randomised: 92 children Study follow‐up: 3 years Exclusions and losses to follow‐up: no exclusions; 6 (6.5%) were lost to follow‐up
Participants	Age: mean = 9.85 years (range 6‐12 years) Gender: 47 boys, 45 girls Culture: Okayama, Japan Inclusion criteria: age 6‐12 years SER error between −1.25 D and −6.00 D in both eyes, as measured by noncycloplegic autorefraction BCVA of 20/20 or better in each eye no other eye disease experience wearing spectacles willingness to wear glasses constantly and attend follow‐up visits acceptance of randomisation Exclusion criteria: astigmatism > 1.50 D in both eyes anisometropia > 1.50 D manifest strabismus; birth weight < 1250 g heterotropia or severe ophthalmic disease that may affect refractive development previous use of PALs or contact lenses
Interventions	PALs (n = 46): 18 months wearing PALs (add +1.50 D), followed by 18 months wearing SVLs SVLs (n = 46): 18 months wearing SVLs, followed by 18 months wearing PALs (addition +1.50 D) Note: prescription changes were made if corrected distance VA was < 20/30 in at least 1 eye
Outcomes	**Primary outcome** Progression of myopia measured by cycloplegic autorefraction **Secondary outcomes** Noncycloplegic autorefraction Noncycloplegic subjective refraction Cycloplegic subjective refraction Distant vision and myopia place Corrected distant vision Lags of accommodation measured by noncycloplegic, open‐field autorefraction Near point of accommodation Reaction of accommodation by open‐field autorefraction Measurements taken at baseline and every 6 months for 3 years **Unit of analysis:** child‐based (mean of both eyes or right eye only)
Notes	Study dates: enroled July 2002‐June 2003 Trial registration: ISRCTN28611140 Funding source: Japanese Ministry of Education, Culture, Sports, Science and Technology, and Megane Tanaka Chain, Ltd

**Table:** Hasebe 2014

***Study characteristics***	
Methods	Study design: parallel‐group RCT Study centre: 3 (Okayama University Medical School, Japan; Eye Hospital of Wenzhou Medical College, China; Eulji University, South Korea) Number randomised: 197 children (120 from China and 77 from Japan) Study follow‐up: 2 years Exclusions and losses to follow‐up: the trial in South Korea was terminated after 12 months due to protocol violation and the data were not included; 28/197 (14%) did not complete 2 years of follow‐up
Participants	Age: mean = 10 years (range 6‐12 years) Gender: 95 boys, 74 girls Culture: Chinese and Japanese children Inclusion criteria: age 6‐12 years SER error between −0.50 D and −4.50 D astigmatism ≤ 1.50 D anisometropia ≤ 1.50 in spherical or cylindrical error BCVA of 6/9 (20/30) or better in each eye normal ocular and general health willingness to wear spectacle lenses continuously willingness and ability to tolerate cycloplegia informed parental consent Exclusion criteria: amblyopia or manifested squint history of rigid contact lens or BF contact lens wear use of BF or progressive lenses or other myopia treatment in previous 12 months abnormal binocular function vestibular disorders or motor imbalance any systemic condition affecting refractive development or vision, or any condition precluding adherence to the study protocol (e.g. not available for follow‐up for 2 years)
Interventions	PA‐PALs +1.0 D (n = 67): positively aspherised PALs with +1.00 D add PA‐PALs +1.5 D (n = 63): positively aspherised PALs with +1.50 D add SVLs (n = 67) Note: all lenses are worn during normal waking hours
Outcomes	**Primary outcomes** Refractive error, measured by cycloplegic autorefraction AL, measured by IOL Master (Carl Zeiss Meditec) **Secondary outcome:** peripheral refractive error, measured using an open field autorefractor Measurements taken at baseline and at 6, 12, 18, and 24 months **Unit of analysis:** eye (both eyes of each child analysed)
Notes	Study dates: July 2008‐June 2009 Trial registration: ACTRN12608000566336 Funding source: "supported by Carl Zeiss Vision" Conflict of interest: "S. Hasebe, Carl Zeiss Vision Australia Holdings Ltd. (F); J. Jun, Carl Zeiss Vision Australia Holdings Ltd. (F); S.R. Varnas, Carl Zeiss Vision Australia Holdings Ltd. (E), P"

**Table:** Hieda 2021

***Study characteristics***	
Methods	Study design: parallel‐group RCT Study centre: 7 university hospitals in Japan Number randomised: 171 children Study follow‐up: 24 months Exclusions and losses to follow‐up: 13 (8%) were withdrawn or lost to follow‐up
Participants	Age: mean = 9.0 years (range 6‐12 years) Gender: 74 boys, 94 girls Culture: Japanese Inclusion criteria: aged 6‐12 years cycloplegic SER between −1.00 D and −6.00 D in both eyes anisometropia of objective spherical equivalent ≤ 1.50 D astigmatism of ≤ 1.50 D (5) corrected VA ≥ 1.0 children with normal IOP Exclusion criteria: abnormal binocular function; amblyopia or manifest strabismus children with ocular diseases other than myopia children with ocular or systemic diseases that potentially have an effect on myopia or refractive power previous or current use of contact lenses, BFs, progressive lenses, or other forms of treatment (including atropine) for myopia children with a history of cardiac or respiratory disease children with a history of pharmacotherapy for asthma over the past year
Interventions	Atropine 0.01% eyedrops (n = 85) Placebo eyedrops (n = 86)
Outcomes	**Primary outcomes** Change in SER error from baseline (cycloplegic autorefraction) Change in AL from baseline (Zeiss IOL Master) **Secondary outcomes** Incidence of AEs Measurements taken every 6 months for 24 months **Unit of analysis:** data from both eyes included (correlation between eyes adjusted in statistical model)
Notes	Study dates: December 2014‐September 2019 Trial registration: JPRN‐UMIN000018041 Funding source: "This study was supported by Eye‐Lens Pte., Ltd., Singapore. The sponsor had no role in the design or conduct of this research." Disclosures: several authors declared support in the form of lecture fees or honoraria from pharmaceutical companies

**Table:** Houston Study 1987

***Study characteristics***	
Methods	Study design: parallel‐group RCT Study centre: 1 (University of Houston, Texas, USA) Number randomised: 207 children Study follow‐up: 3 years Exclusions and losses to follow‐up: 83 (40%) children were excluded from or dropped out of the study
Participants	Age: range 6‐15 years Gender: 58 boys and 66 girls completed the study Culture: children were recruited from patients, from family members of faculty and staff, and from the racially diverse Houston community Inclusion criteria: myopia of −0.25 D in 1 or both eyes ages 6‐15 years BCVA of 20/20 or 20/15 normal ocular health ability to provide informed consent Exclusion criteria: strabismus or amblyopia contact lens wearers astigmatism of ≥ 2.00 D particularly high or low gradient AC/A ratios
Interventions	BFs 1: BFs with +1.00 D addition BFs 2: BFs with +2.00 D addition SVLs Note: prescription changes were made if (1) there was a change in spherical power of ≥ 0.50 D in one or both eyes, or (2) there was an improvement of 1 line of VA. 1 participant was allowed to wear contact lenses when playing basketball
Outcomes	**Patient care team outcomes (unmasked)** Change in refractive error (SER, noncycloplegic subjective refraction) Characteristics of children for whom BFs were most effective in reducing the progression of myopia **Evaluation team outcomes (masked)** Change in refractive error (cycloplegic retinoscopy, noncycloplegic autorefraction, and cycloplegic autorefraction) Change in corneal refracting power Change in anterior chamber depth Change in lens radii of curvature and thickness Change in vitreous chamber depth Change in AL of the eye Measurements taken at baseline and every 6 months for 3 years **Unit of analysis:** data from right eyes
Notes	Study dates: "subjects were admitted to the study over a period of 20 months, in five 'accrual groups.' The first group of subjects entered the study in February, 1981 and completed the study in February, 1984, whereas the last group of subjects entered the study in October, 1982," and completed the study in October, 1985 Trial registration: not reported Materials: BFs were executive 1‐piece lenses in CR‐39 plastic (American Optical Corporation); SVLs were polycarbonate lenses (Gentex Corporation)

**Table:** Jakobsen 2022

***Study characteristics***	
Methods	Study design: parallel‐group RCT Study centre: Department of Ophthalmology, Vejle Hospital, University Hospital of Southern Denmark Number randomised: 60 children Study follow‐up: 18 months Exclusions and losses to follow‐up: 12 (5%) were excluded or lost to follow‐up
Participants	Age: mean = 9.97 years (range 6‐12 years) Gender: 26 boys, 34 girls Culture: European (Scandinavian) Inclusion criteria: myopia –0.5 to –4.75 D cycloplegic spherical in both eyes regular astigmatism ≤ 2.5 D in cycloplegia in both eyes age 6‐12 years at time of inclusion anisometropia < 1.5 D spherical equivalent BCVA of 78 ETDRS letters or better in both eyes Exclusion criteria: manifest or latent squint contraindications to the use of OK lenses (keratoconus, allergic conjunctivitis, keratoconjunctivitis sicca) previous eye surgery chronic eye disease demanding daily use of eye drops 1 or both parents being ethnic Middle Eastern, Asian, African, Latin American, Hispanic or Spanish
Interventions	OK lenses (n = 30) SVLs (n = 30)
Outcomes	**Primary outcomes** Change in AL from baseline (Zeiss IOL Master) **Secondary outcomes** Change in SER from baseline QoL (PREP 2) Safety evaluation (Efron grading scale Measurements taken at 6, 12 and 18 months **Unit of analysis:** average of both eyes analysed
Notes	Study dates: March 2017‐April 2020 Trial registration: NCT03246464 Funding source: grants from the Region of Southern Denmark; The Danish Eye Research Foundation; Fight for Sight, Denmark; The Danish Eye Research Foundation; Disclosures: the study authors declare no conflicts of interest

**Table:** Jensen 1991

***Study characteristics***	
Methods	Study design: parallel‐group RCT Study centre: 1 (Odense University Hospital, Denmark) Number randomised: 159 children Study follow‐up: 2 years Exclusions and losses to follow‐up: 4 (2.5%) children who were randomised were excluded from the analyses; 16 (10%) were lost to follow‐up
Participants	Age: mean = 10.9 years Gender: 87 boys, 72 girls Culture: medical records of children from schools in Odense, Denmark, were screened for myopia (n = 8769). Possible cases of myopia underwent a primary examination (n = 1216). Myopic children with at least −1.0 D in either eye, and in 2nd to 5th grades, were examined at the eye clinic (n = 361). Children meeting inclusion/exclusion criteria at the eye exam were mailed invitations to participate in the trial (n = 227) Inclusion criteria: in 2nd to 5th grades at screening myopia with SER error between −1.25 D and −6.00 D in both eyes normal corrected vision Danish parents affirmative response to mailed invitation for study Exclusion criteria: unilateral myopia eye disease or general illness, especially heart/lung disease experience in pilot study
Interventions	BFs (n = 57): constant wear of BFs with +2.0 D addition to upper edge of reading segment Timolol (n = 51): 1 drop of 0.25% timolol maleate in each eye twice daily and constant wear of SVLs for corrected VA ≥ 0.8 Control (n = 51): constant wear of SVLs for corrected VA ≥ 0.8 Note: participants were permitted to wear their own SVLs if corrected VA was ≥ 0.8
Outcomes	**Primary outcomes** Rate of myopia progression and changes in refractive components (SER measured by cycloplegic autorefraction) Prevention or delay of myopia with BFs Prevention or delay of myopia with pressure‐lowering eye drops **Secondary outcomes** Changes in the fundus IOP Phoria status Accommodation Close work Body growth Measurements taken at baseline and every 6 months for 2 years **Unit of analysis:** right eyes and left eyes analysed separately
Notes	Study dates: screening January‐April 1983; eye clinic exams October 1984‐April 1985 Trial registration: not reported Notes: children who chose not to participate in the study (n = 44) did not statistically differ from those examined with regard to age and degree of myopia

**Table:** Katz 2003

***Study characteristics***	
Methods	Study design: parallel‐group RCT, with 3‐month adaptation period Study centre: 1 (Myopia Clinic of the Singapore Eye Research Institute) Number randomised: 564 children (428 children attended initial visit; 383 children completed the adaptation period) Study follow‐up: 2 years Exclusions and losses to follow‐up: 136 (24%) children who were randomised did not attend the initial visit, and 45 (8%) more did not complete the adaptation period; 86 (22%) of the 383 children who completed the adaptation period were lost to follow‐up
Participants	Age: mean = 8.3 years (range 6‐12 years) Gender: 204 boys, 179 girls Culture: Singaporean children with Chinese ethnicity Inclusion criteria: age 6‐12 years myopia with SER error between −1.0 D and −4.0 D Chinese ethnicity provided informed consent Exclusion criteria: astigmatism > 2.0 D previous contact lens wear other ocular pathologies Note: all participants were provided a 3‐month period to adapt to assigned intervention
Interventions	Contact lenses (n = 158): RGPs worn daily for at least 8 h/day Spectacles (n = 225): SVLs worn daily for at least 8 h/day Note: prescription changes were made if corrected VA fell below 20/40
Outcomes	**Primary outcome** Change in refractive error (SER) Measured by subjective cycloplegic refraction from post adaption through 2 years of follow‐up **Secondary outcomes** Change in keratometry (autokeratometry) Change in AL (A‐scan ultrasonography) Measurements taken at baseline and every 3 months over a 24‐month period **Unit of analysis:** only data from right eyes reported
Notes	Materials: Asian Design Lens, Baush and Lomb, Rochester, New York, USA Trial registration: not reported Adherence to treatment was measured for children and parents (agreement was almost 100%) and was defined as use of contact lenses or spectacle use for at least 8 h/day, 7 days/week Notes: study is also known as the Contact Lens‐Myopia Treatment Study (CL‐MTS) Additional data: study author provided unpublished data via email correspondence

**Table:** Kinoshita 2020

***Study characteristics***	
Methods	Study design: parallel‐group RCT Study centre: Konno Eye Clinic and Omiya Hamada Eye Clinic, Japan Number randomised: 80 children Study follow‐up: 24 months Exclusions and losses to follow‐up: 7 (9%) were withdrawn or lost to follow‐up
Participants	Age: mean = 10.3 years (range 8‐12 years) Gender: 36 boys, 37 girls Culture: Japanese Inclusion criteria: cycloplegic SER of −1.00 D to −6.00D in both eyes astigmatism of ≤ 1.50 D in both eyes anisometropia of ≤ 1.50 D BCVA of ≤ 0.00 logarithm of the minimum angle of resolution (logMAR) unit in each eye Exclusion criteria: presence of ocular disorders such as strabismus and amblyopia systemic disorders such as cardiac or respiratory illness low birth weight of ≤ 1500 g a history of hypersensitivity to atropine using OK and/or atropine ophthalmic solutions
Interventions	Combination group: OK + 0.01% atropine eyedrops (n = 38) Monotherapy group: OK only (n = 35)
Outcomes	**Primary outcomes** Change in AL from baseline (Zeiss IOL Master) **Secondary outcomes** Corneal endothelial cell density Measurements taken every 6 months for 24 months **Unit of analysis:** child‐based average of both eyes
Notes	Study dates: June 2014‐December 2016 Trial registration: UMIN000014362 Funding source: JSPS KAKENHI (Grant No. JP26462646) from the Japan Society for the Promotion of Science, Tokyo, Disclosures: the study authors declare no competing interests

**Table:** Koomson 2016

***Study characteristics***	
Methods	Study design: parallel‐group RCT Study centre: 1 (Kumasi, Ghana) Number randomised: 150 children Study follow‐up: 24 months Exclusions and losses to follow‐up: 1 child in the fully corrected group dropped out before the 24‐month visit
Participants	Age: mean = 12.39 years (range 10‐15 years) Gender: 60 boys, 90 girls Culture: recruited from "eight purposively chosen high socioeconomic schools in the Kumasi metropolis" in Ghana Inclusion criteria: healthy children, ages 10‐15 years spherical equivalent −1.25 to −4.50 D as measured by cycloplegic refraction VA of 0.20 logMAR or worse with habitual spectacles and logMAR 0.00 or better with full correction willingness to wear study spectacles only and to wear them during waking hours Exclusion criteria: strabismus amblyopia astigmatism > 1.25 D anisometropia > 1.00 D parental myopia allergy to cycloplegic agents use of MF optical lenses or pharmacological agents history of contact lens wear
Interventions	Undercorrected group (n = 75): SVLs blurred by +0.50 D Fully corrected group (n = 75): SVLs Note: changes in prescription were made if refraction had changed by at least 0.50 D for 1 or both eyes
Outcomes	**Primary outcome** Change in refractive error (SER) measured by cycloplegic autorefraction at 24 months of follow‐up **Secondary outcomes** Change in AL at 24 months of follow‐up Correlation between baseline accommodative lag and SER changes at 24 months and between average lag (average of the 6th, 12th, 18th, and 24th months near lags) and SER changes at 24 months Measurements taken at 6‐month intervals for 2 years **Unit of analysis:** child‐based (right eye)
Notes	Study dates: enrolment September 2010‐March 2011 Trial registration: not reported Funding source: not reported Disclosures of interest: not reported

**Table:** Lam 2020

***Study characteristics***	
Methods	Study design: parallel‐group RCT Study centre: Centre for Myopia Research, School of Optometry, The Hong Kong Polytechnic University Number randomised: 183 children Study follow‐up: 24 months Exclusions and losses to follow‐up: 14 (8%) lost to follow‐up 9 (5%) withdrawn by 24 months
Participants	Age: mean = 10.1 years (range 8‐13 years) Gender: 105 boys, 78 girls Culture: Chinese Inclusion criteria: SER −1.00 to −5.00D astigmatism and anisometropia of ≤ 1.50 D monocular best VA of 0.00 logMAR or better Exclusion criteria: strabismus and binocular vision abnormalities ocular and systemic abnormalities prior experience of myopia control
Interventions	Defocus incorporated Multiple Segments (DIMS) spectacle lenses (n = 93) SVSs (n = 90)
Outcomes	**Primary outcomes** Change in SER error from baseline (cycloplegic autorefraction) Change in AL from baseline (Zeiss IOL Master) **Secondary outcomes** Distance and near VA (EDTRS charts) Near phoria and accommodative lag Vision quality, comfort and frequency of visual symptoms with lens wear Measurements taken every 6 months for 24 months **Unit of analysis:** data from right eye analysed
Notes	Study dates: August 2014‐July 2017 Trial registration: NCT02206217 Funding source: "This was a collaborative research supported by HOYA, Tokyo, Japan (PolyU grant numbers H‐ZG3B and 1‐87LK). In addition to the financial support, the sponsor also provided manufacturing spectacle lenses and frames. It was a joint collaboration in the design of the DIMS lens" Disclosures: the study authors declare no conflicts of interest

**Table:** LAMP Study 2019

***Study characteristics***	
Methods	Study design: parallel‐group RCT Study centre: CUHK Eye Centre of the Chinese University of Hong Kong, Hong Kong, China Number randomised: 438 children Study follow‐up: 12 months Exclusions and losses to follow‐up: 55 (13%) were withdrawn or lost to follow‐up
Participants	Age: mean = 8.4 years (range 4‐12 years) Gender: 248 boys, 190 girls Culture: Chinese Inclusion criteria: aged 4‐12 years myopic refraction of at least 1.0 D in both eyes astigmatism of < 2.5 D documented myopic progression of at least 0.5 D in the past 1 year Exclusion criteria: ocular diseases (e.g. cataract, congenital retinal diseases, amblyopia, and strabismus) previous use of atropine or pirenzepine, or OK lens or other optical methods for myopia control allergy to atropine systemic diseases (e.g. endocrine, cardiac, and respiratory diseases)
Interventions	Atropine 0.05% eyedrops (n = 102) Atropine 0.025% eyedrops (n = 91) Atropine 0.01% eyedrops (n = 97) Placebo eyedrops (n = 93)
Outcomes	**Primary outcomes** Change in SER error from baseline (cycloplegic autorefraction) Change in AL from baseline (Zeiss IOL Master) **Secondary outcomes** Change in photopic pupil size Change in accommodative amplitude Change in distance VA (logMAR) Change in near VA (logMAR) Change in vision‐related quality of life Measurements taken 4 monthly intervals for 12 months **Unit of analysis:** data from both eyes included (correlation between eyes adjusted in statistical model)
Notes	Study dates: January 2016‐November 2017 Trial registration: CUHK_CCT00383 Funding source: supported in part by the General Research Fund, Research Grants Council, Hong Kong (14111515 [J.C.Y.]); the Direct Grants of the Chinese University of Hong Kong (4054197 [C.P.P.], 4054193 [L.J.C.], and 4054121 and 4054199 [J.C.Y.]); the UBS Optimus Foundation Grant 8984 (J.C.Y.); and the CUHK Jockey Club Children Eye Care Programme Disclosures: the study authors declare no competing interests

**Table:** Lu 2015

***Study characteristics***	
Methods	Study design: parallel‐group RCT Study centre: 1 (Guangzhou Red Cross Hospital, School of Medicine, Jinan University, China) Number randomised: 80 children Study follow‐up: 1 year Exclusions and losses to follow‐up: not reported
Participants	Age: mean = 11.21 years (range 9‐14 years) Gender: 43 boys, 37 girls Culture: Chinese Inclusion criteria: age 9‐14 years progressive (≥ 0.50 D change) myopia from −1.00 D to −5.00 D astigmatism with ≤ 1.50 D with‐rule, ≤ 0.75 D against‐rule BCVA 1.0 or better in both eyes by Snellen chart ocular pressure < 21 mmHg compliance with examination and treatment Exclusion criteria: other ocular condition (glaucoma, cataract, iritis, congenital small cornea, keratoconus, fundus lesions, congenital amblyopia, dominant strabismus) family history of hereditary eye disease (e.g. high myopia, Leber disease) recent or current use of drugs that may affect myopia development previous RGP wear other systemic disease (diabetes, Marfan syndrome, albinism, severe sinusitis, etc.)
Interventions	Mid‐periphery additional lenses (n = 40): addition up to +2.50 D and adjustment training SVLs (n = 40): frame glasses
Outcomes	**Primary outcomes** Change in VA Change in D Change in AL Accommodation amplitude Adjustment reaction index AC/A value **Secondary outcomes** Not distinguished Measurements taken every 3 months for 1 year **Unit of analysis:** eye (both eyes of each child analysed)
Notes	Study dates: January 2014‐July 2015 Trial registration: not reported Funding source: Guangdong Medical Science and Technology Research Foundation (No. A2014557); Department of Ophthalmology, Guangzhou Red Cross Hospital Affiliated to School of Medicine, Jinan University, China

**Table:** Lyu 2020

***Study characteristics***	
Methods	Study design: parallel‐group RCT Study centre: Zhengzhou University People’s Hospital, Henan Eye Hospital, Zhengzhou University, China Number randomised: 102 children Study follow‐up: 13 months Exclusions and losses to follow‐up: 15 (15%) excluded or lost to follow‐up
Participants	Age: mean = 12.6 years (range 8‐12 years) Gender: 49 boys, 42 girls Culture: Chinese Inclusion criteria: SER error −6.00 to −8.75 D astigmatism < 1.50 D BCVA ≤ 0 (logarithmic acuity) normal IOP (10–21 mm Hg) tear break‐up time ≥ 10 s and Schirmer test ≥ 10 mm Exclusion criteria: ocular or systematic diseases that could cause impaired vision or the progression of myopia
Interventions	OK lenses (target myopia reduction −6.00D) (n = 34) OK lenses (target myopia reduction −4.00D) (n =34) SVLs (n = 34)
Outcomes	**Primary outcomes** Change in SER error from baseline (cycloplegic autorefraction) Change in AL from baseline (Zeiss IOL Master) **Secondary outcomes** Satety evaluation (biomicroscopiic examination and Efron grading scales) Change of corneal curvature Change in corneal endothelial density Measurements taken 6 months and up to 13 months **Unit of analysis:** average of both eyes analysed
Notes	Study dates: January 2014‐March 2015 Trial registration: not reported Funding source: The Medical Sciences Project of Henan Province, China (201503203) Disclosures: not reported

**Table:** MIT Study 2001

***Study characteristics***	
Methods	Study design: parallel‐group RCT Study centre: 1 (National Taiwan University Hospital Vision Care Center) Number randomised: 227 children Study follow‐up: 18 months Exclusions and losses to follow‐up: 39 (17%) children were excluded or lost to follow‐up
Participants	Age: range 6‐13 years Gender: 105 boys, 122 girls Culture: school children in Taiwan with an average myopia of −3.27 D Inclusion criteria: age 6‐13 years provided informed consent willing to wear glasses available for follow‐up period Exclusion criteria: tropia or amblyopia increase of > 2 D in any eye during the treatment period
Interventions	SVLs (n = 76): regular SVLs worn all the time and placebo drops PALs (n = 75): MF lenses with the near addition part for reading and placebo drops PALs plus atropine (n = 76): 0.5% atropine instilled once a day at bedtime, in addition to PALs Note: 'prescription changes were made for any child whose refractive error increased by > 0.75 D'
Outcomes	**Primary outcome** Myopic progression measured by cycloplegic autorefraction (SER) **Secondary outcomes** Change in IOP (Tonopen) Change in biometric AL (A‐scan ultrasonography) Change in corneal radius (autorefraction) Measurements taken at baseline and every 3 months over an 18‐month period **Unit of analysis:** data from right eyes analysed
Notes	Study dates: 1997‐2000 Trial registration: not reported Materials: Hoyalux plastic lenses were used for PALs; polycarbonate plastic lenses were used for SVLs Additional data: study author provided unpublished data via email correspondence. PALs plus atropine arm was omitted from the analysis.

**Table:** Moriche‐Carretero 2021

***Study characteristics***	
Methods	Study design: parallel‐group RCT Study centre: Spanish outpatient hospital Number randomised: 339 children Study follow‐up: 24 months Exclusions and losses to follow‐up: 12 (4%) were lost to follow‐up
Participants	Age: mean = 7.3 years (range 5 ‐11 years) Gender: 155 boys, 184 girls Culture: Spansh Inclusion criteria: age 5‐11 years inclusively at baseline cycloplegic SER between −0.50 and −4.50 D in each eye astigmatism ≤ 1.50 D in both eyes anisometropia ≤ 1.00 D no strabismus as confirmed in a cover test BCVA 20/30 or better Exclusion criteria: systemic disease prematurity prior corneal surgery ocular motility anomalies (e.g. corneal transplant or trauma) or ocular inflammation or infection
Interventions	0.01% atropine eyedrops (n = 171) Control (no treatment) (n = 168)
Outcomes	**Primary outcomes** SER error by cyloplegic autorefraction (Potec PRK 5000, Potek, Korea) AL (IOL Master 500, Carl Zeiss Meditec) **Secondary outcomes** Anterior chamber depth Corneal curvature AEs Measurements taken at baseline and 24 months **Unit of analysis:** child‐based (random eye)
Notes	Study dates: 2016‐2017 Trial registration: not reported Funding source: not reported Disclosures: "The authors declare that they have no competing interest"

**Table:** Pärssinen 1989

***Study characteristics***	
Methods	Study design: parallel‐group RCT Study centre: 1 (outpatient clinic of the Central Hospital of Central Finland) Number randomised: 240 children Study follow‐up: 3 years Exclusions and losses to follow‐up: 1 (0.4%) child who was randomised was excluded from the analyses; 2 (0.8%) were lost to follow‐up
Participants	Age: mean = 10.9 years (range 8.8 ‐12.8 years) Gender: 119 boys, 121 girls Culture: schoolchildren with suspected myopia were referred by school nurses and doctors after routine vision check‐ups Inclusion criteria: in 3rd‐5th grade myopia with SER error between −0.25 D and −3.0 D in both eyes and ≥ −0.50 D in the worst eye corrected VA of 6/6 or better in both eyes Exclusion criteria: astigmatism > 2.0 D anisometropia > 2.0 D manifest strabismus horizontal phorias more than −10 or +9 Δ or vertical > 1 Δ previous use of spectacles for myopia eye disease or serious general disease plans to move out of the area in the near future or the child not wanting to have spectacles
Interventions	Distant use (n = 80): minus lenses with full correction to be used for distant vision only; advised to read at greatest distance possible Bifocals (n = 80): clear plastic bifocal lenses with +1.75 D addition for continuous use Continuous use (n = 79): minus lenses with full correction for continuous use; advised to remove spectacles only if there was danger of breaking them Note: prescription changes were made if corrected VA fell below 20/40
Outcomes	**Primary outcome** Change in SER (subjective cycloplegic refraction) **Secondary outcomes** Change in spherical refraction Change in VA Change in astigmatism Change in reading distance Measurements taken at baseline and annually for 3 years **Unit of analysis:** right eyes and left eyes analysed separately
Notes	Study dates: enrolment March 1983‐April 1985 Trial registration: not reported Funding source: Academy of Finland Compliance was measured by questionnaires and participants were classified as compliant, partly compliant, or noncompliant

**Table:** PIR‐205 Study 2004

***Study characteristics***	
Methods	Study design: parallel‐group RCT Study centres: 13 (US academic clinics and private practices) Number randomised: 174 children Study follow‐up: 1 year (planned), plus 1 year extension Exclusions and losses to follow‐up: 27 (15.5%) children who were randomised were excluded from the analyses; 2 (1%) were lost to follow‐up
Participants	Age: mean = 9.9 ± 1.3 years (range 8‐12 years) Gender: 71 boys, 103 girls Culture: children from USA cities of study centres: 73% white, 7% black, 4% Asian, 12% Hispanic, 4% other Inclusion criteria: age 8‐12 years myopia of −0.75 D to −4.00 D BCVA of 20/25 or better normal pupils good general health Exclusion criteria: anisometropia or astigmatism > 1.00 D any manifest tropia current use of either contact lenses or BFs history of ocular surgery, trauma, or chronic ocular disease, including allergic conjunctivitis disease requiring long‐term or regular intermittent medication behavioural or neurological disorder that would interfere with the study participation in any study that involved an investigational drug within 1 month of enrolment intolerance or hypersensitivity to topical anaesthetics, mydriatics, or components of the formulations contraindications to antimuscarinic agents pregnancy or planned pregnancy
Interventions	Pirenzepine (n = 117): 2% pirenzepine ophthalmic gel applied twice a day Control (n = 57): vehicle‐placebo gel applied twice a day
Outcomes	**Primary outcome** Change in refractive error measured by cycloplegic autorefraction (SER) **Secondary outcome** Change in AL measured by A‐scan ultrasonography Measurements taken at baseline and every 3 months for 1 year **Unit of analysis:** average of both eyes
Notes	Study dates: 1 March 2000‐28 February 2002 Trial registration: not reported Funding source: Valley Forge Pharmaceuticals, Inc. Notes: study is also known as the Collaborative Assessment of Myopia Progression with Pirenzepine (CAMPP) study

**Table:** Ren 2017

***Study characteristics***	
Methods	Study design: parallel‐group RCT Study centre: Changsha Honglang Eye Hospital, Changsha 410000, Hunan Province, China Number randomised: 150 children Study follow‐up: 12 months Exclusions and losses to follow‐up: not reported
Participants	Age: mean = 11.96 years (range 8‐15 years) Gender: 72 boys, 78 girls Culture: Chinese Inclusion criteria: not reported Exclusion criteria: not reported
Interventions	Low concentration atropine (0.01%) (n = 50) OK lenses (n = 50) Single vision spectacle lenses (n = 50)
Outcomes	**Primary outcomes** Change in AL from baseline Change in SER from baseline Measurements taken at 12 months **Unit of analysis:** not reported
Notes	Study dates: January 2014‐March 2015 Trial registration: not reported Funding source: not reported Disclosures: not reported

**Table:** ROMIO Study 2012

***Study characteristics***	
Methods	Study design: parallel‐group RCT Study centre: 1 (Hong Kong Polytechnic University) Number randomised: 102 children Study follow‐up: 2 years Exclusions and losses to follow‐up: 24 (24%) children who were randomised (14 in the OK group and 10 in the control group) were excluded from the analyses, of whom, 9 (8.8%) were lost to follow‐up
Participants	Age: mean = 9 years (range 6‐10 years) Gender: 52 boys, 50 girls Culture: Hong Kong Inclusion criteria: aged 6‐10 years myopia between 0.50 D and 4.00 D in at least 1 eye and between 0.50 D and 4.50 D in both eyes astigmatism < 1.50 D, with‐the‐rule astigmatism (axes 180 ± 30) ≤ 1.25 D, astigmatism of other axes ≤ 0.50 D in both eyes anisometropia ≤ 1.50 D) BCVA logMAR 0.10 or better in both eyes symmetrical corneal topography with corneal toricity < 2.00 D in either eye agree to randomisation Exclusion criteria: strabismus at distance or near history of contact lens wear or myopia control treatment contraindication for contact lens wear and OK history of ocular surgery, trauma, or chronic ocular disease concurrent use of medications that may affect tear quality systemic or ocular conditions that may affect tear quality or contact lens wear or that may affect refractive development poor compliance with tests lack of willingness to comply with allocated treatment and follow‐up schedule
Interventions	OK (n = 51): OK lenses SVLs (n = 51) Participants wore assigned treatment on a daily basis
Outcomes	**Primary outcome** Axial elongation **Secondary outcome** AEs Measurements taken at baseline and at 6, 12, 18, and 24 months **Unit of analysis:** child‐based (right eye)
Notes	Study dates: enrolment March 2008‐November 2009 Trial registration: NCT00962208 Funding source: "supported by a collaborative agreement between The Hong Kong Polytechnic University and Menicon Co. Ltd., Japan; contact lenses and solutions and spectacles were sponsored by Menicon Co. Ltd., NKL Contactlenzen B.V., Alcon Hong Kong, Bausch & Lomb Hong Kong, Skyview Optical Co. Ltd., Hong Kong, and Hong Kong Optical Lens Co., Ltd.; and Niche Myopia Funding Grant J‐BB7P for facilities at the Centre for Myopia Research"

**Table:** Ruiz‐Pomeda 2018

***Study characteristics***	
Methods	Study design: parallel‐group RCT Study centre: Novovision ophthalmologic clinic and the Universidad Europea [European University] of Madrid, Spain Number randomised: 79 children Study follow‐up: 24 months Exclusions and losses to follow‐up: 5 (6%) lost to follow‐up
Participants	Age: mean = 10.6 years (range 8‐12 years) Gender: 33 boys and 41 girls Culture: European (Spanish) "87.3% of fathers and 86.1% of mothers were Caucasian [white]" Inclusion criteria: SER −0.75 to −4.00D astigmatism < 1.00 D monocular best VA of +0.10 logMAR or better Exclusion criteria: current or prior contact lenses wear; current or prior use of BFs, PALs, atropine, pirenzepine, or any other myopia control treatment ; regular use of ocular medications and artificial tears; current uses of systemic medications, which may significantly affect contact lens wear, tear film production, pupil size, accommodation, or refractive state a history of corneal hypoesthesia, corneal ulcer, corneal infiltrates, ocular viral or fungal infections, or other recurrent ocular infections strabismus by cover test at far (4 m) or near (40 cm) wearing distance correction; systemic or ocular disease affecting ocular health; keratoconus or an irregular cornea CCLRU grade ≥ 2 for any given anterior segment ocular clinical signs; having pathological myopia; and connective tissue disorders
Interventions	Dual focus soft contact lens (MiSight) (n = 46) SVSCLS (n = 33)
Outcomes	**Primary outcomes** Change in SER error from baseline (cycloplegic autorefraction) Change in AL from baseline (Zeiss IOL Master) Measurements taken every 6 months for 24 months **Unit of analysis:** data from the dominant eye analysed
Notes	Study dates: September 2013‐June 2016 Trial registration: NCT01917110 Funding source: CooperVision S.L. Spain provided financial support. CooperVision S.L. provided the study contact lenses and the funding to carry out the clinical trial Disclosures: the study authors declare no conflicts of interest

**Table:** Sankaridurg 2010

***Study characteristics***	
Methods	Study design: parallel‐group RCT Study centre: 1 (Zhongshan Ophthalmic Center, Sun Yet Sen University, China) Number randomised: 210 children Study follow‐up: 12 months (study was originally planned to be 2 years in duration) Exclusions and losses to follow‐up at 12‐month visit: 2 children who were randomised were excluded from the analyses; 7 (3.3%) were lost to follow‐up
Participants	Age: mean = 11 years (range 6‐16 years) Gender: 110 boys, 100 girls Culture: Chinese children in Guangzhou, China Inclusion criteria: age 6‐16 years bilaterally myopic (spherical component range from −0.75 D to −3.50 D inclusive) with astigmatism not exceeding −1.50 D and maximum of 1.00 D of anisometropia vision correctable to 6/9.5 or better in each eye ocular findings considered to be normal willingness to wear study spectacles and adhere to the protocol schedule
Interventions	Novel spectacle lens type I (n = 50): a rotationally symmetrical design; featured a clear central aperture of 20 mm diameter, with maximum spherical equivalent of +1.0 D relative peripheral power achieved 25 mm from its axis Novel spectacle lens type II (n = 60): a rotationally symmetrical design; featured a clear central aperture of 14 mm diameter, with maximum spherical equivalent of +2.00 D relative peripheral power achieved 25 mm from its axis Novel spectacle lens type III (n = 50): an asymmetrical design; a clear central aperture extended approximately 10 mm either side of centre along the horizontal meridian and a similar distance inferiorly, with positive additional peripheral power of 1.9 D 25 mm from the axis in that meridian SVLs (n = 50): conventional, single vision design Note: lenses were fitted to spectacle frames that ranged in eye‐size from 45 mm to 55 mm with depths from 27 mm to 33 mm
Outcomes	**Primary outcome** Cycloplegic autorefraction assessed with an open‐field autorefractor **Secondary outcome** AL Measurements taken at baseline, 6 months, and 12 months **Unit of analysis:** average of both eyes
Notes	Study dates: recruitment October 2007‐January 2009 Trial registration: not reported Funding source: Australian Federal Government; Institute for Eye Research, Sydney, Australia; Vision CRC, Australia Lenses were provided by industry

**Table:** Sankaridurg 2019

***Study characteristics***	
Methods	Study design: parallel‐group RCT Study centre: Brien Holden Vision Institute clinical trial facility located at Zhongshan Ophthalmic Centre, Guangzhou, China Number randomised: 508 children Study follow‐up: 24 months Exclusions and losses to follow‐up: 118 (27%) lost to follow‐up
Participants	Age: mean = 10.4 years (range not reported) Gender: 246 boys, 262 girls Culture: Chinese Inclusion criteria: SER −0.75 to −3.50D astigmatism ≤ 0.7D vision correctable to 6/9.5 or better normal ocular health Exclusion criteria: pre‐existing ocular or systemic conditions that precluded lens fitting and safe wear of lenses those who underwent corneal refractive surgery those with keratoconus systemic/syndromic conditions associated with myopia such as Marfan syndrome those that underwent atropine treatment, or other forms of myopia control such as PALs or OK
Interventions	Silicon hydrogel contact lenses that imposed myopic defocus across peripheral and central retina (test CL I +1.00 D centrally and +2.50 at 3 mm semi‐chord) (n = 103) Silicon hydrogel; contact lenses that imposed myopic defocus across peripheral and central retina (test CL II; +1.00 D centrally and +1.50 for CL at 3 mm semi‐chord) (n = 101) Extended depth of focus (EDOF) hydrogel contact lenses incorporating higher order aberrations to modulate retinal image quality (test CL III; extended depth of focus of up to +1.75 D) (n = 98) Extended depth of focus (EDOF) hydrogel contact lenses incorporating higher order aberrations to modulate retinal image quality (test CL IV; extended depth of focus of up to +1.25 D) (n = 104) Single vision, silicone hydrogel contact lenses (n = 102)
Outcomes	**Primary outcomes** Change in SER error from baseline (cycloplegic autorefraction) Change in AL from baseline (Haag‐Streit Lenstar 900) Measurements taken every 3 months for 24 months **Unit of analysis:** data from both eyes included (correlation between eyes adjusted in statistical model)
Notes	Study dates: February 2014‐January 2017 Trial registration: ChiCTR‐TRC‐14004227 Funding source: grant support from the Brien Holden Vision Institute. Some of the contact lenses used in the study were supplied by Sauflon Pharmaceuticals Disclosures: none

**Table:** Schwartz 1981

***Study characteristics***	
Methods	Study design: parallel‐group RCT in twins Study centre: not reported Number randomised: 52 children (26 twin pairs) Study follow‐up: 3 years (planned), extended 6 months Exclusions and losses to follow‐up: 2 (4%) children (1 twin pair) who were randomised were excluded from the study; none were lost to follow‐up
Participants	Age: mean = 11.2 years (range 7‐14 years) Gender: 26 boys (13 twin pairs) and 24 girls (12 twin pairs) completed the study Culture: pairs of monozygotic (MZ) twins identified from the Twin Registry of Eye Examinations from the Washington, DC area; all were white Inclusion criteria: MZ twins with bilateral myopia age 7‐13 years shared domicile in local area good general health vision correctable to 20/20 or better third‐degree fusion no other significant abnormality Exclusion criteria: astigmatism or anisometropia > 1.00 D difference in refraction between co‐twins of ≥ 1.50 D in the more advanced eye
Interventions	Treatment group (n = 26): combined treatment of BF spectacles with 1.25 D addition and 2 drops of 1% tropicamide ophthalmic solution instilled to each eye nightly Control group (n = 26): standard spectacle correction (SVLs) Note: full cycloplegic correction in the treatment group was sometimes reduced up to 0.50 D when it did not impair vision below 20/20
Outcomes	**Primary outcome** Change in refractive error (SER) (cycloplegic refraction) **Secondary outcome** Compliance with treatment regimen (child and parent interviews) Measurements taken at baseline and every 6 months for 3 years **Unit of analysis:** average values of both eyes
Notes	Study dates: not reported Trial registration: not reported Materials: 1% tropicamide (Mydriacyl) ophthalmic solution supplied by Alcon Laboratories Inc.

**Table:** Shih 1999

***Study characteristics***	
Methods	Study design: parallel‐group RCT Study centre: 1 (National Taiwan University Hospital) Number randomised: 200 children Study follow‐up: 2 years Exclusions and losses to follow‐up: 14 (7%) children who were randomised were excluded from the study; none were lost to follow‐up
Participants	Age: mean = 9.2 years (range 6‐13 years) Gender: included boys and girls Culture: children recruited from the vision care centre at National Taiwan University Hospital Inclusion criteria: age 6‐13 years myopia with refractive error between −0.50 D and −6.75 D Exclusion criteria: amblyopia or tropia astigmatism ≥ −2.00 D anisometropia ≥ −2.00 D
Interventions	Atropine 0.5% (n = 50): 1 drop of 0.5% atropine nightly; advised to wear BF spectacles Atropine 0.25% (n = 50): 1 drop of 0.25% atropine nightly; advised to wear slightly undercorrected spectacles Atropine 0.1% (n = 50): 1 drop of 0.1% atropine nightly; advised to wear fully corrective spectacles Control (n = 50): 1 drop of 0.5% tropicamide nightly Note: all children were advised to wear sunglasses with UV protection in bright light
Outcomes	**Primary outcome** Change in refractive error measured by cycloplegic autorefraction (SER) Measurements taken at baseline and every 3 months for 2 years **Unit of analysis:** average values of both eyes
Notes	Study dates: 1994 Trial registration: not reported Funding source: Department of Health grant (Taiwan) Additional data: study author provided unpublished data via email correspondence

**Table:** STAMP Study 2012

***Study characteristics***	
Methods	Study design: parallel‐group RCT Study centre: 1 (The Ohio State University College of Optometry, USA) Number randomised: 85 children Study follow‐up: 2 years Exclusions and losses to follow‐up: 2 (2.3%) children did not complete the study
Participants	Age: mean = 9.8 years (range 6‐11 years) Gender: 41 boys, 44 girls Culture: Ohio, USA: 20% black, 68% white, 7% Asian, 5% other Inclusion criteria: 6‐11 years of age at least −0.75 D myopia in each meridian measured with cycloplegic autorefraction but not more than −4.50 D in each meridian in each eye ≥ 1.30 D accommodative lag (4 D stimulus) without correction esophoria at near if > −2.25 D spherical equivalent astigmatism ≤ 2.00 DC in each eye anisometropia ≤ 2.00 D BCVA of at least 20/32 logMAR equivalent birth weight ≥ 1250 g by parental report Exclusion criteria: strabismus history of contact lens wear or previous BF wear diabetes mellitus
Interventions	PALs (n = 42): PALs with + 2.00 D addition (Varilux Ellipse; Essilor of America, Dallas, TX) SVLs (n = 43) Note: children were randomly assigned to wear either PALs or SVLs for the first year of the study; all children wore SVLs for the second year of the study
Outcomes	**Primary outcome** 1‐year change in SER (cycloplegic autorefraction) of the right eye after 1 and 2 years **Secondary outcomes** AL Peripheral ocular shape Central and peripheral aberrations Accommodative lag AC/A ratio Corneal shape and thickness Anterior chamber depth Crystalline lens thickness and curvatures Phoria IOP Measurements taken at baseline and at 6‐month intervals for 2 years **Unit of analysis:** the individual (right eye only)
Notes	Study dates: study recruitment from December 2006‐May 2008 Trial registration: NCT00335049 Funding source: National Eye Institute, National Institutes of Health, USA; Essilor of America, Inc.; American Optometric Foundation Ezell Fellowship Study name: study of theories about myopia progression (STAMP)

**Table:** Swarbrick 2015

***Study characteristics***	
Methods	Study design: paired‐eye, cross‐over RCT Study centre: 1 (School of Optometry and Vision Science, University of New South Wales, Australia) Number randomised: 32 children Study follow‐up: 12 months (two 6‐month periods) Exclusions and losses to follow‐up: 6 (19%) during first period and 8 (25%) during 12‐month study
Participants	Age: mean = 13.4 years (range 8‐16 years) Gender: 14 boys, 12 girls Culture: East Asian ethnicity Inclusion criteria: 8 ‐16 years of age myopic refractive error between −1.00 D and −4.00 D in both eyes with < 0.75 D difference between eyes evidence of myopic progression in 12 months before enrolment with‐the‐rule astigmatism < 1.50 D and no against‐the‐rule astigmatism anisometropia ≤ 0.75 D BCVA of 6/9 or better East Asian ethnicity good general and ocular health Exclusion criteria: contraindications for rigid contact lens wear history of previous rigid contact lens wear abnormal corneal topography abnormal BF function ocular pathology or active ocular surface disease precluding contact lens wear
Interventions	OK (n = 26): OK lens in 1 eye (overnight wear) RGP (n = 26): RGP contact lens in the other eye (daily or extended wear) Note: children were randomly assigned to wear the OK lens in 1 eye and the RGP lens in the other eye for 6 months; at 6 months, the lenses were switched for each eye. The clinical trials registry record also mentioned a matched control group of children who wore spectacles for 12 months; this group was not mentioned in the journal article
Outcomes	**Primary outcome** AL change at 6 months, measured by the IOLMaster ocular biometer **Secondary outcomes** Refractive error (noncycloplegic autorefraction) Corneal curvature Corneal epithelial cell exfoliation during gentle eye wash with sterile saline Amount of bacterial binding Peripheral refractive status Measurements taken at baseline and at 3, 6, 9, and 12 months **Unit of analysis:** the eye
Notes	Study dates: not reported Trial registration: ACTRN12608000007336 Funding sources: Australian Research Council (ARC) Linkage Project Grant Scheme, BE Enterprises Pty Ltd., Capricornia Contact Lens Pty Ltd. (Australia); Boston Products Group of Bausch & Lomb (USA) Disclosures of interest: "the authors have no proprietary or commercial interest in any materials discussed in this article"

**Table:** Tan 2005

***Study characteristics***	
Methods	Study design: parallel‐group RCT Study centres: 7 (academic centres and clinical practices in Singapore, Hong Kong, and Thailand) Number randomised: 353 children Study follow‐up: 1 year Exclusions and losses to follow‐up: 55 (16%) children who were randomised were dropped from the analyses
Participants	Age: mean = 8.7 years (range 6‐13 years) Gender: 177 boys, 176 girls Culture: 99.4% Asian Inclusion criteria: age 6‐12 years myopia of −0.75 D and −4.00 D good general health round pupils refractive to light BCVA of 20/25 or better in each eye Exclusion criteria: astigmatism > 1.00 D anisometropia > 1.00 D strabismus current use of either contact lenses or BFs history of ocular surgery, trauma, or chronic ocular disease, including allergic conjunctivitis previous use of atropine for myopia disease requiring long‐term or regular intermittent medication behavioural or neurological disorder that would interfere with the study participation in any study that involved an investigational drug within 1 month of enrolment intolerance or hypersensitivity to topical anaesthetics, mydriatics, or components of the formulations contraindications to antimuscarinic agents pregnancy or planned pregnancy
Interventions	Gel/gel (n = 142): 2% pirenzepine ophthalmic gel applied twice a day Placebo/gel (n = 140): 2% pirenzepine ophthalmic gel applied once a day and placebo gel applied once a day Placebo/placebo (n = 71): vehicle‐placebo gel applied twice a day
Outcomes	**Primary outcome** Change in refractive error measured by cycloplegic autorefraction (SER) **Secondary outcome** Change in AL measured by A‐scan ultrasonography Measurements taken at baseline and every 3 months for 1 year **Unit of analysis:** average of both eyes
Notes	Study dates: November 2000‐July 2002 Trial registration: not reported Funding source: Valley Forge Pharmaceuticals, Inc., and Novartis Ophthalmics AG

**Table:** Tan 2020

***Study characteristics***	
Methods	Study design: parallel‐group RCT Study centre: HKU eye clinic at Grantham Hospital, Hong Kong, China Number randomised: 72 children Study follow‐up: 12 months Exclusions and losses to follow‐up: 9 (13%) were withdrawn or lost to follow‐up
Participants	Age: mean = 9.0 years (range 6‐11 years) Gender: 23 boys, 36 girls Culture: Chinese Inclusion criteria: 6‐11 years of age low‐to‐moderate myopia (1.00–4.00 D, inclusive) in both eyes refractive astigmatism (negative cylinder) no greater than 2.50 D anisometropia < 1.00 D Exclusion criteria: any contraindications to atropine (e.g. allergy, cardiovascular disease, epilepsy) contact lens wear any history of prior myopia control treatment any ocular or systemic conditions that might influence refractive developments
Interventions	Combined atropine 0.01% eyedrops + OK (n = 36) OK only (n = 36)
Outcomes	**Primary outcomes** Rate of axial elongation (Zeiss IOL Master) **Secondary outcomes** BCVA (ETDRS) SER Accommodation (push‐up method ‐ Royal Air Force Rule) Pupil size (OPD‐Scan III) Corneal topography (Medmont E300) Measurements taken at 6 and 12 months **Unit of analysis:** child‐based‐(right eye)
Notes	Study dates: not reported Trial registration: NCT02955927 Funding source: OK lenses were sponsored by Precision Technology Services, Vancouver, B.C., Canada, and contact lens solutions by Ophtecs Corporation, Japan. Atropine eye drops were partially supported by Aseptic Innovative Medicine Co., Ltd., Taiwan Disclosures: the study authors declare no competing interests

**Table:** Tang 2021

***Study characteristics***	
Methods	Study design: parallel‐group RCT Study centre: Department of Ophthalmology, First affiliated Hospital of Chengdu Medical College, Chengdu 610500, Scichuan Province, China Number randomised: 104 children Study follow‐up: 12 months Exclusions and losses to follow‐up: not reported
Participants	Age: mean = 11.04 years (range not reported) Gender: 48 boys, 49 girls Culture: Chinese Inclusion criteria: spherical equivalent of −1 D to −6.00 D and with‐the‐rule astigmatism ≤ 1.00 D against‐the‐rule astigmatism ≤ 0.50 ≤ 1.0D anisometropia no history of OK wear no other eye system disease and ocular disease decimal BCVA of at least 1.0 in each eye and ocular movement were normal Exclusion criteria: not reported
Interventions	OK lenses (n = 52) SVSCLs (n = 52)
Outcomes	**Primary outcomes** Change in AL from baseline Change in relative peripheral refraction Measurements taken 12 months **Unit of analysis:** not reported
Notes	Study dates: not reported Trial registration: not reported Funding source: Grant of Education Department of Sichuan Province Disclosures: not reported

**Table:** Trier 2008

***Study characteristics***	
Methods	Study design: parallel‐group RCT Study centre: 1 Number randomised: 83 children Study follow‐up: 3 years (intervention 12 months) Exclusions and losses to follow‐up: 6 (7.2%), 9 (10.8%), and 7 (8.4%) were lost to follow‐up during the first year, the second year, and the third year, respectively
Participants	Age: mean 11.3 years (range 8‐13 years) Gender: not reported Culture: Denmark Inclusion criteria: age 8‐13 years minimum myopia of −0.75 D in 1 eye average AL growth rate 0.075 mm‐0.39 mm per 6‐month period Exclusion criteria: severe general ailment (e.g. diabetes, epilepsy, psychiatric disease) other eye disease (e.g. cataract, keratoconus, chronic iritis, glaucoma)
Interventions	Systemic 7‐mx (n = 35): one 400 mg 7‐mx tablet every morning Placebo (n = 42): 1 placebo tablet every morning Notes: children received either 7‐mx or placebo for the first 12 months; all participants received 7‐mx after 12 months (400 mg 7‐mx tablet once or twice per day); "all children used single vision lenses"
Outcomes	**Primary outcome** Axial growth rate measured with noncontact, partial coherence interferometer (Zeiss IOL‐Master) **Secondary outcome** Spherical equivalent measured with autorefractor (Retinomax, Nikon) 30 min after 1 drop of 1% cyclopentolate Measurements taken at −6, 0, 12, 24, and 36 months **Unit of analysis:** the individual (average of both eyes)
Notes	Study dates: October 2003 Trial registration: NCT00263471 Funding source: "supported by grants from 'Jørgen Bagenkop Nielsens Myopi‐Fond' and 'Generalkonsul Einar Høyvalds Fond', and by 'Øjenlæge Klaus Trier ApS'" Declarations of interest: 2 study authors affiliated with Trier Research Laboratories

**Table:** Wang 2017

***Study characteristics***	
Methods	Study design: parallel‐group RCT Study centre: 1 (The People’s Hospital of Yan’an and Affiliated Hospital of Yan’an Medical University, China) Number randomised: 126 children Study follow‐up: 1 year Exclusions and losses to follow‐up: 7 (11.1%) in intervention group and 5 (7.9%) in control group discontinued intervention; 2 (3.2%) in intervention group and 3 (4.8%) in control group were lost to follow‐up
Participants	Age mean (SD): 9.1 (1.4) years in intervention group; 8.7 (1.5) years in control group Gender: 36 (57.1%) boys and 27 (42.9%) girls in intervention group; 31 (49.2%) boys and 32 (50.8%) girls in control group Culture: China Inclusion criteria: diagnosis of low myopia (SER between −0.50 D and −2.00 D by cycloplegic autorefraction) age 5‐10 years normal IOP < 21 mmHg not on any other treatment within 1 month before study enrolment provided informed consent Exclusion criteria: abnormal binocular function or stereopsis other eye disease history of hemostatic or other systemic disorder contact lens or any other intervention for myopia allergy to atropine
Interventions	Atropine (n = 63): 0.5% eye drops once daily at night Placebo (n = 63): vehicle eye drops once daily at night
Outcomes	**Primary outcome** Progression of myopia, measured as a change in SER **Secondary outcome** AL **Safety outcome** AEs Measurements taken at 4, 8, and 12 months **Unit of analysis:** individual (eye with more severe myopia used)
Notes	Study dates: January 2014‐December 2016 Trial registration: not reported Funding source: none

**Table:** Wei 2020

***Study characteristics***	
Methods	Study design: parallel‐group RCT Study centre: Beijing Tongren Hospital, Beijing, China Number randomised: 220 children Study follow‐up: 12 months Exclusions and losses to follow‐up: 61 (28%) were excluded or lost to follow‐up by 12 months
Participants	Age: mean = 9.6 years (range 6‐12 years) Gender: 117 boys,103 girls Culture: Chinese Inclusion criteria: aged 6‐12 years SER −1.00 D to −6.00 D in both eyes astigmatism of ≤ −1.50 D both eyes distance BCVA 0.20 logMAR or better in both eyes IOP < 21 mm Hg Exclusion criteria: children with ocular diseases (eg, amblyopia, strabismus, corneal scar, cataract, glaucoma, or ocular tumour) previous or current treatment with atropine, pirenzepine, contact lenses, BFs, or PALs for myopia allergy to atropine, cyclopentolate, or excipients
Interventions	0.01% atropine eyedrops (n = 110) Placebo eyedrops (n = 110)
Outcomes	**Primary outcomes** SER (HRK7000 A; Huvitz) AL (Haag Streit Lenstar LS900) Measurements taken at baseline 6 and 12 months **Unit of analysis:** child‐based (right eye)
Notes	Study dates: April 2018‐July 2020 Trial registration: ChiCTR‐IOR‐17013898 Funding source: supported by grants from the Integration, Translation and Development on Ophthalmic Technology (Jingyiyan 2016‐5), the Capital Health Research and Development of Special (2016‐4‐2056), the Ministry of Science and Technology, Beijing Nova Program (Z121107002512055), the National Natural Science Foundation of China (81300797), Sanming Project of Medicine in Shenzhen (SZSM201512045) and the Beijing University‐CMU, Advanced Innovation Centre for Big Data‐Based Precision Medicine, Ophthalmic Subcenter (BHME2018‐2019) Disclosures: the study authors declared no competing interest

**Table:** Yang 2009

***Study characteristics***	
Methods	Study design: parallel‐group RCT Study centre: 1 (Guangzhou City, China) Number randomised: 178 children Study follow‐up: 2 years Exclusions and losses to follow‐up: no exclusions; 29 (16%) were lost to follow‐up
Participants	Age: range 7‐13 years Gender: 94 boys, 84 girls Culture: urban children from Guangzhou City, China Inclusion criteria: age 7‐13 years myopia with SER error between −0.50 D and −3.00 D in both eyes, as measured under cycloplegia astigmatism ≤ 1.50 D no anisometropia (difference in SER ≤ 1.00 D between eyes) BCVA 6/6 or better no strabismus normal IOP willingness to wear glasses constantly for study duration understanding of random assignment and willingness to not use other medications Exclusion criteria: any ocular or systemic condition known to influence refractive development use of medication that might affect refractive development moderately or highly myopic (< −3.00 D) parents birth weight ≤ 1250 g previous use of BFs, PALs, or contact lenses
Interventions	PAL group (n = 89): MFl lenses with +1.50 D near addition worn constantly SVL group (n = 89): SVLs worn constantly Note: prescription changes were made if subjective refraction had changed by at least 0.50 D for 1 or both eyes or if clinically indicated
Outcomes	**Primary outcome** Progression of myopia Change in SER error relative to baseline measured by cycloplegic autorefraction with 0.5% tropicamide + 0.5% phenylephrine hydrochloride **Secondary outcomes** Change in vitreous chamber depth by A‐scan ultrasonography Distance (5 m) and near (33 cm) horizontal heterophobia by cover test Accommodative response by open‐field autorefractor Near workload, compliance, and adherence assessed by questionnaire Measurements taken at baseline and every 6 months for 2 years **Unit of analysis:** not reported
Notes	Study dates: enrolment was from July 2004‐March 2005 Trial registration: not reported Funding source: National Natural Science Grant, China Materials: lenses provided by Sola (China) Ltd Compliance in wearing glasses was monitored with separate questionnaires for children and parents (87% overall compliance)

**Table:** Yen 1989

***Study characteristics***	
Methods	Study design: parallel‐group RCT Study centre: 1 (Refraction Clinic, Veterans General Hospital, Taipei, Taiwan) Number randomised: 247 children Study follow‐up: 1 year Exclusions and losses to follow‐up: 151 (61%) children were excluded or lost to follow‐up
Participants	Age: mean = 9 years (range 6‐14 years) Gender: 118 boys, 129 girls Culture: children with simple myopia were randomly selected from clinic records Inclusion criteria: age 6‐14 years myopia with refractive error between −0.5 D and −4.0 D Exclusion criteria: amblyopia or tropia cylinder refraction > 1.0 D
Interventions	Atropine: 1% atropine drops every other night; BF spectacles prescribed 2 weeks after treatment began Cyclopentolate: 1% cyclopentolate drops every night; SVLs prescribed if necessary Saline control: normal saline eye drops every night; SVLs prescribed if necessary
Outcomes	**Primary outcome** Change in refractive error measured by cycloplegic refraction (SER) **Secondary outcomes** Changes in vision, fundoscopy, and IOP Measurements taken at baseline and every 3 months for 1 year Note: baseline for atropine group was measured 2 weeks after treatment began **Unit of analysis:** right eyes only
Notes	Study dates: enrolment from 1 July 1985‐31 October 1986 Trial registration: not reported Funding source: not reported Additional data: study author provided unpublished data via email correspondence

**Table:** Yi 2015

***Study characteristics***	
Methods	Study design: parallel‐group RCT Study centre: 1 (The Third People’s Hospital of Chongqing City, China) Number randomised: 140 children Study follow‐up: 12 months Exclusions and losses to follow‐up: 6 (8%) in treatment group and 2 (3%) in control group withdrew from the study
Participants	Age: mean = 9.8 years (range 7‐12 years) Gender: 65 boys, 67 girls Culture: China Inclusion criteria: children with low myopia: refractive error between −0.50 and −2.00 D in both eyes as measured by cycloplegic autorefraction normal binocular function and stereopsis normal IOP < 21 mmHg willingness and ability to tolerate cycloplegia and mydriasis Exclusion criteria: astigmatism > −1.00 D other ocular disease, such as amblyopia, strabismus, congenital cataract, glaucoma, corneal scar, optic neuropathy, traumatic ocular injury, uveitis, or ocular tumour history of any ocular surgery any systemic disease or condition that could affect visual function and development, including diabetes mellitus and/or chromosome anomaly previous or current use of contact lenses, BFs, PALs, or other forms of treatment (including atropine) for myopia
Interventions	Atropine (n = 70): 1% atropine sulfate once nightly in both eyes Placebo (n = 70): vehicle eye drops (Tears Naturale Free; Alcon, Fort Worth, TX) once nightly in both eyes
Outcomes	**Primary outcomes** Uncorrected distance VA SER (cycloplegic autorefraction) AL Ophthalmoscopy Slit‐lamp biomicroscopy Fundus examination AEs **Secondary outcomes** Not distinguished Measurements taken at baseline and every 3 months up to 1 year **Unit of analysis:** individual (right eye)
Notes	Study dates: enrolment from January‐October 2012 Trial registration: not reported Funding source: not reported Declarations of interest: not reported

**Table:** Zhang 2021

***Study characteristics***	
Methods	Study design: parallel‐group RCT Study centre: Peking University Third Hospital, China Number randomised: 60 children Study follow‐up: 24 months Exclusions and losses to follow‐up: 22 (28%) were excluded or lost to follow‐up
Participants	Age: mean = 11 years (range 8‐14 years) Gender: 29 boys, 31 girls Culture: Chinese Inclusion criteria: 8‐14 years old; myopia (both eyes) − 0.75 D to − 5.00 D; astigmatism ≤ 1.50 D; anisometropia ≥1.00 D BCVA logMAR: 0.10 or better in both eyes Exclusion criteria: previous experience wearing contact lenses contraindication for contact lenses (e.g. dry eye, trichiasis) intermittent or constant strabismus a history of ocular surgery, trauma concurrent use of medications that may affect refractive development (e.g. atropine) systemic conditions that may affect tear quality or contact lens wear (e.g. diabetes, allergies)
Interventions	OK lenses (n = 30) SVLs (n = 110)
Outcomes	**Primary outcomes** AL **Secondary outcomes** AEs Measurements taken at baseline 6 and 12 months **Unit of analysis:** not reported
Notes	Study dates: not reported Trial registration: ChiCTR 1800017535 Funding source: Capital’s Funds for Health Improvement and Research (grant number 2018‐2‐4092) Disclosures: "The authors declare that they have no known competing financial interests or personal relationships that could have appeared to influence the work reported in this paper."

**Table:** Zhao 2021

***Study characteristics***	
Methods	Study design: parallel‐group RCT Study centre: Affiliated Hospital of Dalian Medical University, China Number randomised: 80 children Study follow‐up: 12 months Exclusions and losses to follow‐up: not reported
Participants	Age: mean = 10.3 years (range 5‐14 years) Gender: 40 boys, 40 girls Culture: Chinese Inclusion criteria: cycloplegic SER at least −1.00 D and within −1.00 to −6.00 DS astigmatism ≤ −1.00 DC Exclusion criteria: wearing contact lenses within 3 days at the start of examination children with ocular disorders such as glaucoma, cataract, keratopathy, strabismus, and amblyopia, and systemic disorders such as cardiac and respiratory illnesses IOP > 21 mm Hg and difference between the eyes > 8 mm Hg use of anticholinergic and cholinergic drugs within the past 1 month wearing OK lenses therapy of traditional Chinese medicine low birth weight (< 1500 g) history of hypersensitivity to atropine or anticholinergic drugs
Interventions	Atropine 0.01% eyedrops (n = 20) SVLs (n = 20) OK lenses (n = 20) Combination OK lenses + atropine 0.01% eyedrops (n = 20)
Outcomes	**Primary outcomes** AL (LS 900 biometer) SER (TOPCON (KR‐800)) **Secondary outcomes** IOP (TOPCON (CT‐IP)) Corneal topography (OPD‐Scan III) Measurements taken at baseline 3, 6 and 12 months **Unit of analysis:** eye (both eyes of each child analysed)
Notes	Study dates: January 2019‐April 2020 Trial registration: not reported Funding source: "This study was funded by Life Science Society of Liaoning." Disclosures: "The authors declare that they no conflict of interest."

**Table:** Zhu 2021

***Study characteristics***	
Methods	Study design: parallel‐group RCT Study centre: The Second People’s Hospital of Yunnan Province, China Number randomised: 660 children Study follow‐up: 48 months Exclusions and losses to follow‐up: 90 (14%) were excluded or lost to follow‐up
Participants	Age: mean = 9.1 years (range 5‐14 years) Gender: 286 boys, 284 girls Culture: Chinese Inclusion criteria: age 6‐12 years initial myopic SER −2.0D to−8.00D astigmatism ≤ 1.0 D SE progression rate ≥ 1 D/year in the last year normal binocular function and stereopsis normal IOP Exclusion criteria: ocular diseases, such as amblyopia, strabismus, congenital cataract, glaucoma, corneal scar, optic neuropathy, traumatic ocular injury, uveitis, or ocular tumour history of any ocular surgeries; any systemic diseases or conditions that could affect visual function and development, including diabetes mellitus and/or chromosome anomaly previous or current use of contact lenses, BFs, PALs, or other forms of treatment, including atropine, for the control of myopia
Interventions	Atropine 1% eyedrops (n = 262); years 1 and 2: once monthly dosing, year 3: once every 2nd month, year 4: no treatment Placebo eyedrops (n = 308); same dosing schedule as for the active comparator
Outcomes	**Primary outcomes** SER (cycloplegic autorefraction) AL (Zeiss IOL Master 500) **Secondary outcomes** IOP (Nidek Co. Ltd, Tokyo, Japan) Measurements taken every 6 months for 48 months **Unit of analysis:** not reported
Notes	Study dates: December 2014‐December 2018 Trial registration: not reported Funding source: "This work is supported by the National Natural Science Foundation of China, Grant No. 81560168." Disclosures: "The authors declare that they no conflict of interest."

**7‐mx:** 7‐methylxanthine; **AC/A:** accommodative‐convergence (AC) over accommodation (A); **AE:** adverse event; **AL:** axial (eye) length; **BCVA:** best corrected visual acuity; **BF:** bifocal; **BOZD:** back optic zone diameter; **D:** dioptre; **DF:** dual focus: **EDTRS:** standardised chart for measuring visual acuity (Early Treatment of Diabetic Retinopathy Study); **HAL:** highly aspheric spectacle lenses; **IOP:** intraocular pressure; **MF:** multifocal; **OK:** orthokeratology; **PAL:** progressive addition lens; **QoL:** quality of life; **RCT:** randomised controlled trial; **RGP:** rigid gas‐permeable (contact lenses); **SAL:** slightly aspheric spectacle lenses; **SER:** spherical equivalent refraction; **SVL:** single vision spectacle lenses; **SVSCL:** single vision soft contact lenses; **VA:** visual acuity;

### Characteristics of excluded studies [ordered by study ID]

**Table:**

Study	Reason for exclusion
Abraham 1966	Not randomised: case report
ACHIEVE Study 2008	Not intended to control progression of myopia: glasses vs contacts for self‐esteem in school children
ACTRN12620000159954	Not randomised
ACTRN12620001046998	Not randomised
Aller 2008	Interventional twin case series: included only 1 pair of twins: 1 randomised to wear BF SCLs and the other to wear SVSCLs for 1 year; both wore BFSCLs for the second year
Anderson 2016	Ineligible outcome
Andreo 1990	Not randomised: not intended to control progression of myopia; participants > 18 were included
Avetisov 2019	Not randomised
Bakaraju 2015	6‐month data only
Baldwin 1969	Not randomised: participants selected treatment assignment
Baltimore Myopia Project 1946	Interventions not eligible: vision training for myopia; interventions of vision training were not prespecified in the protocol
Baronet 1979	Notr andomised: retrospective review of patients treated with atropine at a medical practice with no comparison group
Bedrossian 1979	Not randomised: method of allocation was not specified. Cross‐over study of atropine in 1 eye for 1 year, with the fellow eye serving as the control, then alternated treatment after each year for 4 years
Berkeley OK Study 1983	Population not eligible: participants were 21‐28 years old
Bier 1988	Not randomised: sequential assignment to groups
Brodstein 1984	Not randomised: "the lack of randomization permits a possibility for bias"
Cambridge Anti‐Myopia Study 2013	Ineligible population (included children and young adults)
Chan 2014	Interventional twin case series: included only 1 pair of twins: 1 randomised to wear OK lens and the other to wear SVLs for 2 years
Chan 2020	Outcome not eligible
Chen 2012	Not randomised: allocation was done by parental decision
Chen 2014	Not randomised: cohort study of children wearing SVLs with full correction or undercorrection
Chen 2016	Not randomised: treatment group included participants who chose to wear OK lenses; controls included participants who had never worn OK lenses
Cheung 2018	Wrong outcome
ChiCTR2000034760	Population not eligible
ChiCTR2000038078	Population not eligible
ChiCTR2100052322	Intervention not eligible
ChiCTR‐IOC‐17010525	Population not eligible
ChiCTR‐OON‐17010470	Not randomised
ChiCTR‐TRC‐070000297	Intervention not eligible
Cho 2012	Interventions not eligible: comparison of fenestrated OK lenses vs nonfenestrated OK lenses; interventions comparing types of OK lenses were not prespecified in the protocol
Cho 2017	Interventions not eligible: comparison of continuing vs discontinuing OK wear after 2 years; interventions comparing length of OK wear were not prespecified in the protocol
Choi 2005	Not randomised: study was reported only as a conference abstract and randomisation was not specified ("We prescribed 1% atropine once a day with bifocal glasses to the treated group (41 patients) and prescribed only glasses to the control group (43 patients)")
Chou 1997	Not randomised: allocation was by parental decision
Diaz‐Llopis 2018	Not randomised
Dumbleton 1999	Interventions not eligible: lenses with different oxygen permeability; interventions comparing oxygen permeability not prespecified in the protocol
Dyer 1979	Not randomised: case‐control study
Ebri 2007	Not intended to control progression of myopia: cycloplegic effect and pupillary dilation outcomes, as well as cost‐effectiveness; follow‐up 3 days
Eissa 2018	Interventions were not eligible
Filip 2000	Population was not eligible: myopia progression in adults
French 2016	Letter/commentary
Gimbel 1973	Not randomised: comparison of patients vs an historical cohort
Goss 1984	Not randomised: treatment group included patients with overcorrection; controls included random patients selected retrospectively
Grosvenor 1991	Not randomised: historical control group
He 2015	Population not eligible
He 2016	Not randomised: retrospective cohort study; comparison of OK lenses vs SVLs
Horner 1999	Not intended to control progression of myopia: comparison of soft spherical contact lenses vs spectacles; SCLs not expected to slow myopia progression. In fact, the study was conducted because researchers believed that SCLs may increase myopia progression
Hosaka 1982	Not randomised: interventional case series of children aged 6‐14 years treated with labetalol ophthalmic solution
Hosaka 1988	Not randomised: interventional case series
Hua 2017	Interventions not eligible: cluster‐RCT of elevated light levels in classrooms to prevent myopia onset or progression; interventions of light levels were not prespecified in the protocol
Huang 2015a	Intervention not eligible
Huang 2020	Not randomised
Huffman 2002	Not intended to control progression of myopia: aspheric vs spheric lenses; outcome to decrease spherical aberration; adults were included
Jiang 2018	Not randomised
Jiang 2021	Not randomised
Jin 2015	Not randomised
Jones Jordan 2012	Not randomised
Jong 2015	Ineligible outcome
JPRN‐jRCTs032180418	Intervention not eligible
Kao 1988	Not randomised: children were enroled in 2 separate series of participants
Keller 1996	Not randomised: all children wore RGPs
Kennedy 1995	Not randomised: treatment was atropine; controls were patients matched by medical records
Khoo 1999	Not randomised: study reported that "children were randomly selected from the various schools in Singapore. They were then randomly selected for contact lens wear" Children in the RGP cohort who completed 3 years of follow‐up were compared with a cohort of children who wore spectacles
Kubena 2002	Not randomised: cohort study that compared spectacle lenses that filtered non‐visible light vs conventional spectacle lenses
Lakkis 2006	Not intended to control progression of myopia: 2‐week randomised cross‐over trial to evaluate visual performance and satisfaction of clear and photochromic spectacle lenses in children aged 10‐15 years wearing fully corrected spectacles
Lam 2018	Ineligible outcome
Lee 2016	Not randomised: dosing study conducted to compare 0.125% or 0.25% atropine; controls were patients who preferred SVLs
Leung 1999	Not randomised: odd or even case numbers determined the 2 groups
Li 2005	Not randomised: experimental group received progressive MF lenses; control group wore common glasses; participants were 6‐23 years old
Liang 2008	Interventions not eligible: RCT comparing atropine eye drops alone vs combined treatment with atropine and stimulation of the auricular acupoints in school‐aged children with myopia
Lu 2010	Not randomised: case‐control study comparing myopic children treated with seasonal doses of atropine vs nonmyopic children
Lu 2019	Outcome not eligible
Lyu 2021	Ineligible study design
Ma 2014	Interventions not eligible: cluster‐RCT with 3 groups: free spectacles provided in class; vouchers for free spectacles; and prescriptions for spectacles; interventions of accessibility to spectacles were not prespecified in the protocol
Mandell 1959	Not randomised: historical cohort, including adults
Marcotte Collard 2019	Outcome not eligible
Meythaler 1971	Not randomised: interventional cases series (70 eyes in people from 8‐35 years of age were checked); 3 groups were based on age; youngest group was 8‐19 years old
Mori 2021	Intervention not eligible
NCT00348166	Not randomised
NCT00848900	Population not eligible
NCT02055378	Ineligible intervention
NCT03372551	Ineligible patient population
NCT03512626	Ineligible patient population
NCT03761758	Ineligible patient population
NCT04126057	Outcome not eligible
NCT04238897	Intervention not eligible
NCT04301323	Intervention not eligible
NCT04492397	Outcome not eligible
NCT04923841	Population not eligible
NCT05156190	Ineligible outcome
Neetens 1985	Not randomised: control group consisted of participants who could not use BFs
Nesterov 1990	Not randomised: comparison of a group using cycloplegics and ocular hypotensives vs a reference group for progression of myopia
Ng 2019	Outcome not eligible
Oakley 1975	Not randomised: control group consisted of children (or parents) who refused BFs
Parker 1958	Not randomised: comparison of author's practice vs other practices
Perrigin 1990	Not randomised: treatment group was given silicone lenses; control consisted of an historical cohort
Pirenzepine 2003	Not randomised: review of pirenzepine studies and mechanism of action
Plowright 2015	Not intended to control progression of myopia: RCT to evaluate daily disposable contact lenses vs SVLs for 2 weeks
Pritchard 1999	Not intended to control progression of myopia: extended wear for low Dk vs high Dk lenses in adults
Rah 2002	Population not eligible: overnight OK in adults (LOOK study); not randomised
Rainey 2000	Interventions not eligible: vision therapy vs control; interventions for vision training were not prespecified in the protocol
Ritchey 2005	Population not eligible: included adults aged ≥ 18 (COLM study)
Sankaridurg 2003	Not intended to control progression of myopia: RCT conducted to compare AEs for SCLs vs SVLs; participants were 16‐35 years old
Santodomingo‐Rubido 2012	Not randomised: allocation was done by parental decision
Savoliuk 1968	Not randomised: comparison of groups using SVLs continuously or for distance use only vs no spectacles
Saxena 2021	Letter/commentary
Shen 2011	Allocation method not clear, randomisation not specified: compared groups using 0.25% atropine vs no atropine
Shimmyo 2003	Allocation method not clear, randomisation not specified: atropine vs control for 2 years
Shum 2003	Not randomised: comparison of groups using OK vs no OK
SMART Study 2009	Not randomised: comparison of groups using OK lenses vs daily wear silicone hydrogel SCLs
Soni 2006	Not randomised: included adults
Stone 1976	Not intended to control progression of myopia: study authors state that "the research team is not purposely attempting to flatten the cornea in order to arrest the myopia"
Sun 2007	Not randomised: case‐control study of spectacle users vs controls
Syniuta 2001	Not randomised: intervention group included patients whose parents requested treatment for myopic progression; control group comprised the next myopic child by alphabetical order after study child’s record number
Takano 1964	Not randomised: cohort study comparing treatment with Mydrine (tropicamide + phenylephrine) eye drops with or without Neosynesin (phenylephrine) eye drops; included boys and girls with myopia ages 7‐19 years; follow‐up was 20 days
Tan 2012	Not randomised
Tan 2019	Outcome not eligible
Tang 2020	Ineligible study design
Tian 2022	Outcome not eligible
Tilia 2018	Ineligible outcome
Toki 1960	Not randomised: cohort study of patients receiving 5% Neosynesin (phenylephrine) eye drops; included boys and girls with myopia ages 7‐21 years; follow‐up was 14‐28 days
Tokoro 1964	Not randomised: non‐randomised study of treatment with Mydrine (tropicamide + phenylephrine) eye drops + 5% Neosynesin (phenylephrine) eye drops + low‐frequency electro stimulus in children ages 7‐15 years; included children with hyperopia
Tokoro 1965	Not randomised: retrospective cohort comparing full correction spectacles vs undercorrection (< −1 D) spectacles or full correction in case of need in children ages 7‐14 years; included children with hyperopia
TO‐SEE Study 2013	Not randomised: prospective cohort study of children wearing OK lenses vs SVLs
Wan 2020	Ineligible study design
Wu 2018	Letter/commentary
Xiao 2009	Not randomised: observational study of 2 groups of children who wore RGPs vs spectacles
Yamada 2004	Not randomised: review article with some cohort data on children with high myopia
Yamaji 1967	Not randomised: observation of children treated with Mydrine‐M; no control group
Yang 2017	Not intended to control progression of myopia: evaluated accommodative lag in groups using OK vs SVLs for 1 year
Yi 2011	Population not eligible
Young 1992	Not intended to control progression of myopia: comparison of overnight lenses for 12 months in adults only
Zeng 2009	Not intended to control progression of myopia: RCT to evaluate visual performance and satisfaction of ready‐made spectacles vs custom spectacles in Chinese school‐aged children with uncorrected refractive error
Zhang 2019	Outcome not eligible
Zhao 2017	Ineligible intervention
Zhou 2015	Not intended to control progression of myopia: evaluated accommodative lag in groups using RGPs vs SVLs for 1 year
Zhou 2016	Not randomised: 400 children wearing OK lenses or SVLs selected from patient records
Zhou 2021	Not randomised

**AE:** adverse event; **BF:** bifocal;**Dk:** oxygen permeability; **MF:** multifocal; **OK:** orthokeratology; **RCT:** randomised controlled trial; **RGPs:** rigid gas‐permeable (contct lenses); **SCL:** soft contact lens; **SVSCL:** single vision soft contact lenses; **SVL:** single vision spectacle lenses

### Characteristics of studies awaiting classification [ordered by study ID]

**Table:** Viswanath 2022

Methods	Study design: parallel‐group RCT Study centre: not reported Number randomised: 60 children Study follow‐up: 12 months Exclusions and losses to follow‐up: not reported
Participants	Age: mean = intervention group 11.33 ± 3.31 years, placebo 10.8 ± 3.41) Gender: not reported Culture: Indian Inclusion criteria: baseline myopia ≥ −2.00 D to −6.00 D Exclusion criteria: not reported
Interventions	0.01% atropine (n = 30) Placebo (n = 30)
Outcomes	**Primary outcomes** SER AL Unit of analysis: child‐level
Notes	Study period: not reported Trial registration: not reported Funding source: not reported

**Table:** Wang 2005

Methods	Study design: parallel‐group RCT Study centre: 1 (Shanghai, China) Number randomised: 104 children Study follow‐up: 18 months Exclusions and losses to follow‐up: not reported
Participants	Age: mean = 11.6 years (range 6‐15 years) Gender: 51 boys, 53 girls Culture: recruited from outpatient department of Eye & Ear, Nose, Throat Hospital in Shanghai, China Inclusion criteria: age 6‐15 years myopia Exclusion criteria: not reported
Interventions	PAL group (n = 50): add not reported SVL (n = 54)
Outcomes	**Primary outcomes** Refractive error (cycloplegic autorefraction) AL Anterior chamber depth Lens thickness Corneal curve (vertical and horizontal) Heterophoria (vertical and horizontal) **Secondary outcomes** Not distinguished Measurements taken at baseline and every 6 months for 18 months **Unit of analysis:** not reported
Notes	Study period: enrolment from April 1999‐April 2000 Trial registration: not reported Funding source: not reported

**AL:** axial length; **PAL:** progressive addition lenses; **SER: s**pherical equivalent refracton; **SVL:** single vision spectacle lenses

### Characteristics of ongoing studies [ordered by study ID]

**Table:** ACTRN12605000633684

Study name	Trial of an experimental soft contact lens designed to inhibit the progression of axial myopia in children
Methods	Randomised cross‐over design (within‐person study)
Participants	**Inclusion criteria:** 40 children aged 11‐14 years with progressing myopia, SER of −1.50 to −4.00, VA of 6/6 or better **Exclusion criteria:** children with astigmatism > 0.75 D, anisometropia > 1.00 D, abnormal binocular vision, ocular pathology, systemic disease with ocular complications, active anterior surface disease that would preclude contact lens wear, inadequate fit of soft contact lenses
Interventions	**Intervention:** frequent replacement soft contact lens that both corrects vision and simultaneously produces myopic retinal defocus **Comparison intervention:** standard frequent replacement SVSCLs
Outcomes	**Primary outcome:** myopia progression rate **Secondary outcomes:** SER, AL **Maximum follow‐up:** 20 months
Starting date	November 2005 **Estimated end date:** not reported
Contact information	anzctr.org.au/ACTRN12605000633684.aspx
Notes

**Table:** ACTRN12608000566336

Study name	Myopia control lens efficacy trial
Methods	Randomised parallel‐group design
Participants	**Inclusion criteria:** 300 children aged 6‐12 years with SER error of –0.50 to ‐4.50 D, astigmatism of not > −1.50 D, anisometropia of not more than −1.50 D in spherical or cylindrical error, BVCA of at least 6/9 (20/30) in each eye, normal ocular health other than myopia, no prior use of BF or progressive lenses in the last 12 months, no rigid contact lenses or BF contact lens experience, willingness not to wear contact lenses, in satisfactory health, willingness and ability to tolerate cycloplegia, informed parental consent **Exclusion criteria:** no availability for follow‐up for at least 2 years, absence of parental consent to the random assignment of their child to 1 of 3 spectacle lens groups, any systemic condition that might affect refractive development or systemic disease that may affect vision or refractive error, previous use of contact lens/PALs or other treatment for myopia within the last 12 months, defective binocular function, amblyopia and or manifested squint, vestibular disorders or motor imbalance, any other conditions precluding adherence to the protocol
Interventions	**Intervention 1:** binocular 1.00 D PALs **Intervention 2:** binocular 1.50 D PALs **Comparison intervention:** single vision binocular lens
Outcomes	**Primary outcomes:** SER, AL **Secondary outcome:** peripheral refractive error **Maximum follow‐up:** 24 months
Starting date	September 2008 **Estimated end date:** September 2009
Contact information	anzctr.org.au/Trial/Registration/TrialReview.aspx?id=83124
Notes

**Table:** ACTRN12611000499987

Study name	Duplex orthokeratology (DOK) and myopia progression in children
Methods	Randomised parallel‐group design
Participants	**Inclusion criteria:** 10‐14 years of age SER error between −1.25 D and −4.00 D myopia progression of at least 0.50 D in previous 12 months astigmatism < 1.50 D anisometropia < 1.00 D BCVA of 6/6 or better in both eyes good general and ocular health parents and child able to communicate in English **Exclusion criteria:** recent rigid contact lens wear history of corneal surgery active eye disease including keratoconus severe dry eye symptoms systemic disease affecting VA taking medication that could affect ocular health
Interventions	**Intervention:** duplex (dual focus optic zone) OK lens in 1 eye (overnight wear) **Intervention comparison:** conventional OK lens in the other eye (overnight wear) Note: children were randomly assigned to wear the OK lens in the dominant eye or the nondominant eye
Outcomes	**Primary outcome:** change in vitreous chamber depth, measured by non‐contact Optical Low‐Coherence Reflectometry (Lenstar LS 900, Haag Streit, Switzerland) **Secondary outcomes:** magnitude of central and peripheral refractive error, amplitude of accommodation, contrast sensitivity
Starting date	May 2011 **Estimated end date:** not reported
Contact information	John Phillips, PhD, or Martin Loertscher Department of Optometry and Vision Science The University of Auckland 85 Park Road Grafton, Auckland 1023 email: j.phillips@auckland.ac.nz; m.loertscher@auckland.ac.nz anzctr.org.au/ACTRN12611000499987.aspx
Notes

**Table:** ACTRN12611000582954

Study name	Myopia control with progressive spectacle lenses trial (MCPAL‐3)
Methods	Randomised parallel‐group design
Participants	**Inclusion criteria:** 167 children aged 7‐12 years with refractive error between −1.00 D and −4.50 D, BCVA of at least 6/9 or 20/30 in each eye, and anisometropia not more than −1.50 D, astigmatism not greater than −1.50 D, no other ocular conditions, no history of using BF or PALs in 12 months preceding study, and tolerant to cycloplegia, with parental consent **Exclusion criteria:** systemic condition affecting vision or refractive errors, history of contact lens or other treatment for myopia in the preceding 12 months, impaired binocular function, history of amblyopia, manifest squint, vestibular disorders or motor imbalance, other conditions that prevent adherence to protocol
Interventions	**Intervention:** PALs **Comparison intervention:** SVLs
Outcomes	**Primary outcome:** progression in refractive error (SER using cycloplegic autorefraction) **Secondary outcome:** AL **Maximum follow‐up:** 24 months
Starting date	June 2011 **Date of last participant enrolment:** June 2012
Contact information	anzctr.org.au/Trial/Registration/TrialReview.aspx?id=343027
Notes

**Table:** ACTRN12611001148965

Study name	To determine the rate of refractive error change in children wearing multifocal soft contact lens as compared to those wearing single vision soft contact lenses
Methods	Randomised parallel‐group design
Participants	**Inclusion criteria:** 40 children aged 8‐14 years with cycloplegic autorefraction: sphere −0.50 D to −4.00 D; cylinder 0 to −0.75 D; BCVA 6/9 or better; ability to safely wear contact lenses; distortion‐free keratometric readings; no active corneal infection, inflammation, or infection of the anterior chamber, eye disease, injury or abnormality of the cornea; conjunctiva or eyelids affecting wearing of contact lenses; no previous ocular surgery; no severe insufficiency of lacrimal secretion; no evidence of corneal hypoesthesia; no systemic disease or use of medications that may affect the eye or produce an adverse response by the wearing of contact lenses **Exclusion criteria:** binocular vision problems, strabismus, amblyopia, external ocular problems that may impact lens fit (i.e. lid ptosis, chalazia, swollen lids)
Interventions	**Intervention:** MFSCLS **Comparison intervention:** SVCLs
Outcomes	**Primary outcome:** rate of myopia progression **Secondary outcomes:** fitting characteristics of, and ocular response to, soft contact lenses **Maximum follow‐up**: 3 years
Starting date	November 2005 **Estimated end date:** not reported
Contact information	anzctr.org.au/Trial/Registration/TrialReview.aspx?id=347659
Notes

**Table:** ACTRN12617000598381

Study name	A pilot study to evaluate the effectiveness of daily 0.01% atropine eye drop therapy in modifying the progression of myopia, in Australian children
Methods	Randomised parallel‐group design
Participants	**Inclusion criteria:** aged 6‐16 years, myopia with SER error ≥ −1.5 D in each eye, documented myopic progression of ≥ −0.5 D over the previous 12 months in either eye, astigmatism < −1.5 D, intraocular difference in spherical equivalent < 1 D, corrected VA > logMar 0.2, normal IOP, normal ocular health, no history of cardiac/respiratory disease, willingness and ability to provide details of parents' country of origin, ability to provide appropriate parental/carer consent **Exclusion criteria:** astigmatism of ≤ 1.5 D; ≥ 1 D anisometropia; severe developmental delay (inability to participate in subjective refraction of testing); ocular comorbidities such as glaucoma, aphakia, pseudophakia, uveitis, keratoconus, or connective tissue disease (e.g. Marfan syndrome, vitreoretinal dystrophies); severe ocular surface disease; previous atropine treatment for amblyopia at any time in the past
Interventions	**Intervention:** 0.01% atropine eye drops **Comparison intervention:** placebo eye drops
Outcomes	**Primary outcome:** mean change in SER error **Secondary outcomes:** amplitude of accommodation, choroidal thickness, corneal curvature and AL, Wilkins Rate of Reading test comparison, IOP, stereovision assessment, QoL **Maximum follow‐up:** 24 months
Starting date	January 2017 **Estimated end date:** December 2020
Contact information	anzctr.org.au/Trial/Registration/TrialReview.aspx?id=372668
Notes

**Table:** ACTRN12618000242224

Study name	Prospective, contralateral, randomized, cross‐over dispensing clinical trial to compare the myopia progression rate between a myopia control contact lens and single vision contact lenses
Methods	Randomised cross‐over design (within‐person study)
Participants	**Inclusion criteria:** 45 participants aged 6‐17 years, spherical equivalent −0.75 D to −3.50 D, cylinder no more than −1.00 D, anisometropia ≤ 0.75 D, vision correctable to 6/9.5 or better **Exclusion criteria:** pre‐existing ocular irritation precluding contact lens fitting, systemic or ocular condition or injury, corneal refractive surgery, keratoconus, allergy to cyclopentolate, astigmatism > 1.00 D in either eye, strabismus, amblyopia, any ocular or systemic disease associated with myopia, retinopathy of prematurity, current orthoptic treatment or vision training, eye injury or surgery within 12 weeks before enrolment, atropine treatment for myopia control, previously worn BF or PAL spectacles or antimyopia contact or OK lenses, anisometropic by > 0.75 D
Interventions	**Intervention:** experimental contact lens (lens type not reported) **Comparison intervention:** single vision contact lens
Outcomes	**Primary outcome:** change in cycloplegic autorefraction spherical equivalent **Secondary outcomes:** change in axial length **Maximum follow‐up:** 12 months
Starting date	January 2018 **Estimated end date:** not reported
Contact information	anzctr.org.au/Trial/Registration/TrialReview.aspx?id=374450
Notes

**Table:** Azuara‐Blanco 2020

Study name	Low‐dose (0.01%) atropine eye‐drops to reduce progression of myopia in children: a multicentre placebo‐controlled randomised trial in the UK (CHAMP‐UK)—study protocol
Methods	Randomised parallel‐group design
Participants	**Inclusion criteria: aged** 6–12 years, myopia ‐0.50 D or greater, SER error in both eyes, BCVA distance 0.20 logMAR or better in both eyes, and no other significant ocular or systemic morbidities **Exclusion criteria:** children with myopia ≥ −10.00 D or astigmatism ≥ 2.00 D in either eye will be excluded
Interventions	**Intervention:** atropine 0.01% eyedrops 1 drop in the randomised eye for 2 years **Comparison intervention:** placebo
Outcomes	**Primary outcome:** SER after 24 months **Secondary outcome:** AL BCVA distance (uniocular and binocular), uniocular and binocular near VA (ETDRS), reading speed, pupil diameter, accommodation, AE rates and allergic reactions, QoL (EQ‐5D‐Y) and tolerability **Maximum follow‐up:** 24 months
Starting date	April 2019 **Estimated end date:** February 2024
Contact information	clinicaltrials.gov/ct2/show/NCT03690089
Notes	Trial registration numberS: ISRCTN99883695, NCT03690089

**Table:** ChiCTR1800016504

Study name	Clinical effect of vitamin B12 eye drops on myopia in children
Methods	Randomised parallel‐group design
Participants	**Inclusion criteria:** age 6‐12 years; the refractive power of the eyes after dilation is between −1.0 and −3.0 D; no refractive error (binocular D within −1.0 D); binocular astigmatism < −1.5 D; far vision of the eyes can be corrected to at least 0.8; the IOP is < 21 mmHg; no allergy to dilated pupils; no corneal plasticiser has been used to treat myopia; no amblyopia, squint, etc. **Exclusion criteria:** failing to meet the inclusion criteria; unwilling to participate in this study
Interventions	**Intervention:** vitamin B12 eye drop **Comparison intervention:** no intervention
Outcomes	**Primary outcome:** dioptre **Secondary outcomes:** not reported **Maximum follow‐up:** 12 months
Starting date	July 2018 **Estimated end date:** June 2019
Contact information	chictr.org.cn/showprojen.aspx?proj=26962
Notes

**Table:** ChiCTR1800017535

Study name	Randomized controlled trial for orthokeratology lens to correct anisometropia in children
Methods	Randomised parallel‐group design
Participants	**Inclusion criteria:** aged 8‐14 years, myopia in both eyes −0.75 D to −5.00 D, astigmatism ≤ 1.50 D, interocular difference in spherical equivalent ≥ 1.00 D **Exclusion criteria:** wearing any type of contact lenses for > 3 months, eye diseases such as trichiasis, conjunctivitis, dry eye, incomplete eyelid closure, intermittent or manifest strabismus, diabetes, asthma, low immunity or other general diseases, systemic or local application of atropine or other drugs that may affect AL; intolerance of corneal contact lenses or spectacles
Interventions	**Intervention:** OK lenses worn overnight **Comparison intervention:** SVLs
Outcomes	**Primary outcome:** AL, SER **Maximum follow‐up:** 12 months
Starting date	September 2018 **Estimated end date:** December 2020
Contact information	chictr.org.cn/showproj.aspx?proj=29222
Notes

**Table:** ChiCTR1800017683

Study name	A double‐masked comparative study of peripheral defocus lenses
Methods	Randomised parallel‐group design
Participants	**Inclusion criteria:** age 8‐13 years; SER of −0.75 to −4.75 D in each eye, as measured by cycloplegic autorefraction; astigmatism of not more than 1.50 D; anisometropia of not more than 1.00 D; BCVA ≥ 0.05 LogMAR (≥ 0.9 as Snellen) **Exclusion criteria:** history of PALs or BFl use and no prior use of contact lenses; strabismus by cover test at near and distance; ocular disease with full ophthalmic examination, such as retinal disease, cataract and ptosis; systemic or neurodevelopmental conditions; ocular or systemic medicine, which might affect myopia progression or VA through known effects on retina, accommodation or significant elevation of IOP
Interventions	**Intervention 1:** "defocus lenses" **Intervention 2:** "defocus lenses" **Comparison intervention:** SVLs
Outcomes	**Primary outcome:** refractive power; AL; contrast VA **Secondary outcomes:** not reported **Maximum follow‐up:** not reported
Starting date	July 2018 **Estimated end date:** November 2020
Contact information	chictr.org.cn/hvshowproject.aspx?id=13585
Notes

**Table:** ChiCTR1800018092

Study name	Comparison of myopia control effect between single use ortho‐k and combined with 0.01% atropine eye drops in children
Methods	Randomised parallel‐group design
Participants	**Inclusion criteria**: children with myopia were included in the randomised control, with no gender limitation, aged 7‐12 years old, clear refractive media, equivalent spherical lens ≤−5.00D, 40.00D ≤ corneal base curvature < 45.50 D, and corneal astigmatism ≤ 1.50 D **Exclusion criteria**: rule out basic eye diseases that may affect vision, corneal plasticiser and potion
Interventions	**Intervention**: OK glass **Comparison intervention**: 0.01% atropine eye drops once per night
Outcomes	**Primary outcome**: AL **Secondary outcomes**: SER, corneal curvature **Maximum follow‐up**: not reported
Starting date	
Contact information	
Notes	Study name: Comparison of myopia control effect between single use ortho‐k and combined with 0.01% atropine eye drops in children

**Table:** ChiCTR1900021316

Study name	Clinical observation for auricular acupoint stimulation combined with low‐concentration atropine in myopia control and its effect on accommodative microfluctuations
Methods	Randomised parallel‐group design
Participants	**Inclusion criteria:** age 6‐11 years children; male or female; with simple myopia; 0.5% tocarbamide mydriatic optometry: +0.5 DS to −6.0 DS; corneal topography Kmax: 42‐44 D; astigmatism of < 1.50 D, anisometropia of < 1.00 D, IOP of 10‐21 mmHg; patient with good compliance who volunteers to join the study and signs informed consent **Exclusion criteria:** patient with other ocular diseases (e.g. cataract, congenital retinal disease, strabismus, amblyopia) or systemic diseases; patient with active eye lesions or undergoing eye surgery; allergy to atropine; patient whose skin of the auricular acupoint area is broken or patient who has allergy to auricular plaster; guardians do not hold reasonable expectations
Interventions	**Intervention:** 0.01% atropine eyedrops combined with auricular acupoint stimulation **Comparison intervention:** 0.01% atropine eyedrops
Outcomes	**Primary outcome:** uncorrected distance VA; dioptre; AL **Secondary outcomes:** anterior chamber depth; accommodation amplitude; accommodative microfluctuations **Maximum follow‐up:** not reported
Starting date	February 2019 **Estimated end date:** May 2020
Contact information	chictr.org.cn/hvshowproject.aspx?id=15141
Notes

**Table:** ChiCTR2000033904

Study name	Clinical study of combined orthokeratology (OK lens) and 0.01% atropine solution to control myopia progression in children
Methods	Randomised cross‐over design
Participants	**Inclusion criteria:** children aged 8‐12 years; spherical equivalent myopia −1 D to −4 D; astigmatism < 1.5 D; anisometropia < 1.0 D; corrected vision ≥ 1.0; no history of eye surgery; no eye or systemic disease affecting vision **Exclusion criteria:** congenital or pathological myopia; premature infants and low birth weight; allergic to atropine; using other drugs or treatments to control myopia
Interventions	**Intervention:** combined OK and 0.01% atropine eye drops **Comparison intervention:** OK and placebo (blank solvent)
Outcomes	**Primary outcome:** myopia progression (AL, SER) **Maximum follow up:** not reported
Starting date	June 2020 **Estimated end date:** February 2022
Contact information	chictr.org.cn/com/25/hvshowproject.aspx?id=160051
Notes

**Table:** ChiCTR2000036880

Study name	A multicenter, double‐blind, randomized controlled clinical trial for defocused spectacle lenses in controlling progression of high myopia in children
Methods	Randomised parallel‐group design
Participants	**Inclusion criteria:** children aged 8‐14 years, SER −5 to −8 D, astigmatism ≤ 1.5 D, anisometropia ≤ 1.50 D, progression of myopia in the last year ≥ 0.5 D; BCVA ≥ 0.8, near acuity ≥ 1.0, birth weight ≥ 1500 g **Exclusion criteria:** ocular or systemic diseases (e.g. Marfan's syndrome, retinopathy of prematurity, etc.) that may affect vision or refractive development; other treatment for myopia control in the last year, corneal refractive surgery
Interventions	**Intervention:** defocussed spectacle lenses **Comparison intervention:** SVLs
Outcomes	**Primary outcome:** AL **Secondary outcome:** refractive status, VA, accomodative amplitude, pupil diameter, contrast sensitivity, AES **Maximum follow‐up:** not reported
Starting date	October 2020 **Estimated end date:** September 2022
Contact information	chictr.org.cn/showproj.aspx?proj=59891
Notes

**Table:** ChiCTR2000036917

Study name	A multicenter, double‐blind, randomized controlled clinical trial for defocused soft contact lens in controlling progression of high myopia in children
Methods	Randomised parallel‐group design
Participants	**Inclusion criteria:** children aged 8‐14 years, SER −8 D to −5 D, astigmatism ≤ 1.5 D, anisometropia ≤ 1.5 D, progression of myopia in the last year ≥ 0.5 D; BCVA ≥ 0.8 **Exclusion criteria:** ocular or systemic diseases (e.g. Marfan's syndrome, retinopathy of prematurity, etc.) that may affect vision and refractive development, patients with xerophthalmia, allergic conjunctivitis, entropion, trichiasis, severe keratoconjunctival infection, keratoconus and other eye diseases, allergies or contraindications to cycloplegia drug, received other treatment for myopia control in the last year, such as atropine and other anticholinergic drugs, OK, defocused soft contact lens, defocused spectacles, etc), prior corneal refractive surgery
Interventions	**Intervention:** defocussed soft contact lenses **Comparison intervention:** SVSCLs
Outcomes	**Primary outcome:** AL **Secondary outcome:** refractive status, VA, accomodative amplitude, pupil diameter, contrast sensitivity, AEs **Maximum follow‐up:** not reported
Starting date	October 2020 **Estimated end date:** September 2022
Contact information	chictr.org.cn/showproj.aspx?proj=59881
Notes

**Table:** ChiCTR2000037113

Study name	Precise intervention of progressive myopia in children, adolescents and young adults. A randomized clinical trial
Methods	Randomised parallel‐group design
Participants	**Inclusion criteria:** children and adolescents aged 8‐15 years, equivalent spherical power (8‐9 years old −6.00 D to −2.00 D, 10 years old: −6.00 D to −3.00 D, 11‐12 years old: −6.00 D to −4.00 D, 13‐15 years old: −6.00 D to −5.00 D), astigmatism ≤ 1.50 D; spherical anisometropia ≤ 1.50 D **Exclusion criteria:** eye diseases that may affect vision or ametropia, systemic disease (immune system diseases, central nervous system diseases, Down's syndrome, asthma, severe cardiopulmonary function, severe liver and kidney dysfunction), contraindications to atropine, use of anticholinergic drugs within the past month e.g. atropine or pirenzipine; use of OK, multifocal soft lens or myopia control spectacles within the past month
Interventions	**Intervention:** 0.01% atropine eyedrops **Intervention:** 1% atropine eyedrops **Intervention:** combined OK and 0.01% atropine eyedrops **Intervention:** combined OK and 1% atropine eyedrops
Outcomes	**Primary outcome:** SER, AL **Secondary outcome:** choroidal thickness, BCVA, near VA, accommodation amplitude, pupil size **Maximum follow up:** not reported
Starting date	October 2020 **Estimated end date:** not reported
Contact information	chictr.org.cn/showproj.aspx?proj=60282
Notes

**Table:** ChiCTR2000037443

Study name	A randomized parallel controlled trial of the effect of peripheral myopia defocus lens for preventing and controling myopia in children
Methods	Randomised parallel‐group design
Participants	**Inclusion criteria:** aged 6‐15 years, emmetropia (equivalent spherical power between +0.75 and −0.50 D), myopia (equivalent spherical power between −0.75 to −8.00 D), astigmatism ≤ 1.50 D, spherical anisometropia ≤ 2.00 D, VA ≥ 1.0, clear refractive media, no nystagmus, good fixation **Exclusion criteria:** narrow anterior chamber or IOP > 20 mmHg or glaucoma, keratitis, acute infection or inflammation, contact lens wear (including those wearing contact lens during the study)
Interventions	**Intervention:** peripheral defocus spectacle lenses (Hoya Myosmart) **Comparison intervention:** SVLs
Outcomes	**Primary outcome:** ocular health evaluation, cycloplegic refraction, AL, VA, contrast sensitivity **Maximum follow up:** not reported
Starting date	September 2020 **Estimated end date:** December 2021
Contact information	chictr.org.cn/com/25/hvshowproject.aspx?id=59371
Notes

**Table:** ChiCTR2000040990

Study name	The effect of myopia control and influence of visual quality in children treated with orthokeratology of aspherical base curve design
Methods	Randomised parallel‐group design
Participants	**Inclusion criteria:** age between 8‐12 years old, BCVA (ETDRS) in a single eye ≥ 20/25, SER −0.75 D ~ −4.00 D, corneal astigmatism ≤ 1.50 D, anisometropia ≤ 1.00 D, no other methods of myopia control, no history of wearing contact lenses **Exclusion criteria:** narrow anterior chamber or IOP > 21 mmHg; suffering from keratitis, keratoconus, glaucoma, strabismus or amblyopia; accommodative insufficiency
Interventions	**Intervention:** aspherical base curve designed OK lenses **Intervention:** spherical base curve designed OK lenses
Outcomes	**Primary outcome:** AL, objective refraction, relative peripheral refraction, choroidal thickness, ocular comfort (OSDI questionnaire), AEs **Maximum follow up:** not reported
Starting date	December 2020 **Estimated end date:** June 2022
Contact information	chictr.org.cn/hvshowproject.aspx?id=84332
Notes

**Table:** ChiCTR2100041788

Study name	The effect of peripheral defocus modifying spectacle lenses on myopia control
Methods	Randomised cross‐over trial
Participants	**Inclusion criteria:** 8‐14 years old, myopia −1.00 to −4.00 D, astigmatism ≤ −2.00: BCVA ≥ 1.0, anisometropia ≤ 2 D **Exclusion criteria:** wearing contact lenses, peripheral defocus modifying spectacle lenses or using 0.01% atropine, strabismus, intermittent exotropia
Interventions	**Intervention:** peripheral defocus modifying spectacle lenses **Comparison intervention:** OK lenses
Outcomes	**Primary outcome:** AL **Maximum follow up:** not reported
Starting date	January 2021 **Estimated end date:** August 2023
Contact information	chictr.org.cn/hvshowproject.aspx?id=82249
Notes

**Table:** ChiCTR‐INR‐17013794

Study name	The effectiveness safety of corneal contact lens used to correct myopia: a multi‐center, randomized, open and positive parallel control clinical trial
Methods	Randomised parallel‐group design
Participants	**Inclusion criteria:** 41 patients aged 8‐40 years with myopia ≤ 4.00 D, astigmatism with‐the‐rule of < 1.75 D, and astigmatism against‐the‐rule of < 1.00 D; BCVA not less than 20/20; corneal curvature at 40.00 D‐46.00 D; dioptre stay stability before trial; has not worn hard contact lenses in the past 2 months **Exclusion criteria:** systemic disease that causes low immunity or effects on corneal shape; corneal abnormality; corneal surgery; history of corneal or ocular trauma; hypocorneal sensory impairment; intraocular surgery; fundus lesions; ocular disease; pregnant or lactating; use of drugs that cause dry eyes or affect corneal curvature; allergy to contact lens or its solution; pupil diameter > 6.2 mm
Interventions	**Intervention:** corneal contact lens 2 (not specified) **Comparison intervention:** corneal contact lens 2 (not specified)
Outcomes	**Primary outcome:** VA **Secondary outcomes:** not reported **Maximum follow‐up:** not reported
Starting date	May 2017 **Estimated end date:** December 2018
Contact information	chictr.org.cn/showprojen.aspx?proj=23702
Notes

**Table:** ChiCTR‐INR‐17013853

Study name	Effects of orthokeratology and combined with 0.01% atropine on myopia control: a multicenter comparative study
Methods	Randomised parallel‐group design
Participants	**Inclusion criteria:** 216 children aged 8‐15 years; spherical degree without dilation ≥ −1.00 D and ≤ −5.50 D; equivalent spherical degree ≥ −1.00 D and ≤ −5.50 D; astigmatism ≤ −1.50 D; BCVA ≥ 1.0 D; no strabismus; no contact lens wearing history; no history of myopia control by optical or drug route; no active inflammation or ocular surface disease; no serious ocular appendage lesions and eye organic disease; co‐operation with researchers **Exclusion criteria:** systemic connective tissue disease and autoimmune disease; history of ocular trauma or surgery; history of severe ocular infection
Interventions	**Intervention 1:** OK at night **Intervention 2:** OK at night and 0.01% atropine eye drops before sleep **Comparison intervention:** SVLs
Outcomes	**Primary outcomes:** AL, refraction, eyesight **Secondary outcomes:** IOP, corneal topography **Maximum follow‐up:** 12 months
Starting date	December 2017 **Estimated end date:** June 2019
Contact information	chictr.org.cn/showprojen.aspx?proj=22940
Notes

**Table:** ChiCTR‐IOR‐17010432

Study name	Myopia progression with invisible round segment bifocal spectacle lenses
Methods	Randomised parallel‐group design
Participants	**Inclusion criteria:** BCVA of 6/9.5 or better with spectacles in each eye; normal ocular health; ability to comply with trial protocol; parental ability to understand English and Mandarin and parental consent **Exclusion criteria:** history of allergy to topical anaesthetics; strabismus; eye surgery; ocular or systemic condition affecting vision; ocular injury; use of BFs, spectacles, OK, vision training, orthoptic training, or conditions that affect ability to wear spectacles
Interventions	**Intervention:** BF spectacles **Comparison intervention:** SVLs
Outcomes	**Primary outcome:** SER **Secondary outcome:** AL **Maximum follow‐up**: not reported
Starting date	February 2017 **Estimated end date:** September 2018
Contact information	chictr.org.cn/showproj.aspx?proj=17727
Notes

**Table:** ChiCTR‐IOR‐17011993

Study name	Prospective, masked, contralateral, randomized, cross‐over dispensing clinical trial to compare the myopia progression rate between myopia control contact lenses and single vision contact lenses
Methods	Randomised cross‐over design
Participants	**Inclusion criteria:** aged 7‐13 years inclusive; spherical component −0.75 D to −3.50 D with cylinder no more than −0.75 D; anisometropia ≤ 0.75 D; informed consent; parent or guardian who is able to read and comprehend Mandarin and give informed consent as demonstrated by signing a record of informed consent by both parent/guardian and participant; ocular health findings considered to be normal and that would not prevent patient from safely wearing contact lenses; vision correctable to 6/9.5 or better in each eye with study contact lenses **Exclusion criteria:** pre‐existing ocular irritation that would preclude contact lens fitting; any systemic or ocular condition or ocular injury that may preclude safe wearing of contact lenses; having undergone corneal refractive surgery; at baseline, astigmatism > 0.75 D in either eye; past strabismus and/or current ongoing amblyopia; any ocular, systemic, or other condition or disease with possible associations with myopia or affecting refractive development; current orthoptic treatment or vision training; eye injury or surgery within 12 weeks immediately before enrolment for this study; having undergone atropine treatment for myopia control, worn BF or PALs or antimyopia contact lenses previously; having worn OK lenses previously; requiring anticholinergic medication for gastrointestinal or other conditions; at baseline, anisometropic by > 0.75 D
Interventions	**Intervention 1:** single vision contact lenses in both eyes **Intervention 2:** myopia control contact lens in 1 eye, and single vision contact lens in the other eye; contact lenses swapped between eyes after 6 months **Comparison intervention:** myopia control contact lens in 1 eye, and single vision contact lens in the other eye; contact lenses swapped between eyes after 6 months
Outcomes	**Primary outcome:** SER, AL **Secondary outcomes:** not reported **Maximum follow‐up:** not reported
Starting date	Not reported **Estimated end date:** not reported
Contact information	http://www.chictr.org.cn/showprojen.aspx?proj=20301
Notes

**Table:** ChiCTR‐IPD‐16008844

Study name	Clinical study of low‐concentration atropine in controlling child myopia
Methods	Randomised parallel‐group design
Participants	**Inclusion criteria:** 400 children aged 6‐12 years; myopia spherical equivalent degree: −1.25 to −6.0; astigmatism < 2.0; distance corrected VA ≥ 0.8, without significant skew and other eye disease; no ocular inflammation; no history of ocular trauma; no history of ocular surgery **Exclusion criteria:** congenital myopia and pathological myopia; premature and low birth weight myopia patients, with no other related myopia drugs and training method in the past 6 months
Interventions	**Intervention 1:** 0.005% concentration atropine **Intervention 2:** 0.01% concentration atropine **Intervention 3:** 0.02% concentration atropine **Intervention 4:** 0.02% concentration atropine, once every 2 days **Comparison intervention:** spectacles
Outcomes	**Primary outcomes:** "myopia degree" **Secondary outcome:** not reported **Maximum follow‐up:** not reported
Starting date	July 2016 **Estimated end date:** July 2020
Contact information	chictr.org.cn/com/25/hvshowproject.aspx?id=11127
Notes

**Table:** ChiCTR‐TRC‐07000029

Study name	Double‐blinded, randomized controlled trial about the influence of new lenses on the progress of children's myopia
Methods	Randomised parallel‐group design
Participants	**Inclusion criteria:** 200 children aged 6‐16 years; degree of myopia > −0.50 D and < −4.50 D; astigmatism degree < −1.50 D; binocular anisometropic degree < 1 D; healthy ocular region; VA can be corrected to 6/9 (20/30) or higher **Exclusion criteria:** strabismus or amblyopia; history of allergy to tropicamide; any ophthalmopathy, previous ophthalmic surgery, systemic disease that may be related to myopia; using anticholinergic drugs; taking part in other myopia‐controlled study; previous wearing of OK lenses in the last 2 weeks; accepted or are participating in orthophoria treatment or vision training
Interventions	**Intervention 1:** type A lenses **Intervention 2:** type B lenses **Intervention 3:** type C lenses **Comparison intervention:** routine lenses
Outcomes	**Primary outcomes:** axial length **Secondary outcome:** "diopter" **Maximum follow‐up:** not reported
Starting date	October 2007 **Estimated end date:** November 2009
Contact information	chictr.org.cn/showproj.aspx?proj=9496
Notes

**Table:** ChiCTR‐TRC‐07000044

Study name	Clinical randomized controlled trial of progressive addition lenses on control of myopia in Chinese adolescents
Methods	Randomised parallel‐group design
Participants	**Inclusion criteria:** 178 adolescents aged 7‐18 years; computer optometry after cycloplegia; binocular myopia; spherical equivalent degree between −0.75 and −3.00 D; astigmatism degree ‐1.50 D; binocular anisometropic degree < 1.00 D; bilateral corrected VA > 1.0; normal IOP: binocular IOP < 21 mmHg, and difference < 2 mmHg; no history of wearing contact lenses, BFs, or multifocal lenses; term infants; birth weight > 1250 g; agree to wear lenses and follow up for > 2 years; understand the study objective and accept the randomised allocation **Exclusion criteria:** manifest strabismus or other ophthalmopathy; systematic disease; use of drugs that may influence the refractive status; myopia degree of either parent > 3 D; use of contact lenses or other myopia treatment methods in the study
Interventions	**Intervention:** gradual focal lens **Comparison intervention:** routine single lens
Outcomes	**Primary outcomes:** myopic degree, eyeball biotest **Secondary outcome:** heterophoria **Maximum follow‐up:** not reported
Starting date	July 2004 **Estimated end date:** May 2007
Contact information	chictr.org.cn/showproj.aspx?proj=9481
Notes

**Table:** ChiCTR‐TRC‐09000476

Study name	Novel spectacle lenses vs single vision spectacle lenses on progression of myopia in children: a randomized clinical trial
Methods	Randomised parallel‐group design
Participants	**Inclusion criteria:** children aged 6‐12 years with SER between −0.75 D and −3.50 D; astigmatism ≤ −1.50 D; BCVA of at least 6/9.5 with spectacles; ability to comply with study protocol; normal ocular health **Exclusion criteria:** anisometropia ≤ 1.00 D; history of allergy to topical anaesthetics; strabismus; eye surgery; ocular or systemic conditions affecting vision; ocular injury; use of BFs, spectacles, OK, vision training, orthoptic training, or conditions that affect ability to wear spectacles; concurrent participation in another clinical trial
Interventions	**Intervention:** not reported (“Iteration E”) **Intervention:** not reported (“Iteration G”) **Intervention:** not reported (“Iteration F”) **Intervention:** not reported (“Iteration H”) **Comparison intervention:** SVLs
Outcomes	**Primary outcomes:** cycloplegic autorefraction **Secondary outcome:** not reported **Maximum follow‐up:** not reported
Starting date	August 2009 **Estimated end date:** December 2011
Contact information	chictr.org.cn/showprojen.aspx?proj=9058
Notes

**Table:** ChiCTR‐TRC‐10000914

Study name	Progression of refractive error in myopic Chinese children wearing commercially available single vision spectacles
Methods	Randomised parallel‐group design
Participants	**Inclusion criteria:** children aged 7‐14 years; SER between −0.50 D and −3.50 D; astigmatism ≤ 0.75 D; BCVA in each eye of at least 6/9.5; ability to comply with protocol; parental ability to comprehend Mandarin; parental ability to consent **Exclusion criteria:** anisometropia not greater than 1.50 D; prior use of atropine for myopia control; prior use of BF or PAL spectacles or concurrent use of OK contact lenses in the previous 12 months; prior eye surgery or ocular trauma; history of ocular or systematic condition that affects refractive development
Interventions	**Intervention:** spherical profile spectacle lenses **Comparison intervention:** aspheric front surface spectacle lenses
Outcomes	**Primary outcomes:** SER, AL **Secondary outcome:** not reported **Maximum follow‐up:** not reported
Starting date	July 2010 **Estimated end date:** September 2013
Contact information	chictr.org.cn/showprojen.aspx?proj=8624
Notes

**Table:** ChiCTR‐TRC‐11001463

Study name	Efficacy of MyoVision spectacle lenses for slowing the progression of myopia
Methods	Randomised parallel‐group design
Participants	**Inclusion criteria:** 200 children aged 6‐12 years; myopic; spherical component −0.75 D to −3.50 D with astigmatism no more than −1.50 D; having at least 1 parent who is myopic; willingness to comply with wearing and visit schedule; having normal ocular health findings; having vision correctable to 6/9.5 or better in each eye with spectacles **Exclusion criteria:** allergy to tropicamide or topical anaesthetics; anisometropic by > 1.00 D; strabismus or amblyopia; previous eye surgery; ocular or systemic disease with possible associations with myopia; any ocular injury or condition of the cornea or conjunctiva or eyelids; having worn BFs or MyoVision spectacles in the last 12 months; having worn OK or BF contact lenses in the last 12 months; current orthoptic treatment or vision training
Interventions	**Intervention:** MyoVision spectacles **Comparison intervention:** SVLs
Outcomes	**Primary outcome:** myopia progression **Secondary outcome:** AL **Maximum follow‐up:** not reported
Starting date	August 2011 **Estimated end date:** January 2014
Contact information	chictr.org.cn/hvshowproject.aspx?id=1096
Notes

**Table:** ChiCTR‐TRC‐11001746

Study name	Assessment of myopia progression rates in children wearing either a multifocal center near or single vision soft contact lens
Methods	Randomised parallel‐group design
Participants	**Inclusion criteria:** 100 children aged 10‐17 years; Chinese ethnicity; myopic (short‐sighted) up to −8.00 D of spherical equivalent; willingness to comply with wearing and clinical trial visit schedule as directed by the investigator; having ocular health findings considered to be “normal” and that would prevent the patient from safely wearing contact lenses; having distance vision correctable to 6/9.5 or better in each eye with study contact lenses **Exclusion criteria:** pre‐existing ocular irritation, injury, or condition; any systemic disease that adversely affects ocular health; eye surgery within 12 weeks immediately before enrolment for this study; previous corneal refractive surgery; keratoconus; known allergy to, or history of, intolerance to tropicamide or topical anaesthetics; past strabismus and/or amblyopia; any ocular, systemic, or other condition or disease with possible associations with myopia or affecting refractive development; current orthoptic treatment or vision training; having undergone atropine treatment for myopia control; having worn BF or PAL spectacles in the previous 12 months; having worn OK lenses in the previous 12 months; requiring anticholinergic medication for gastrointestinal or other conditions; pregnant or lactating female patients
Interventions	**Intervention 1:** multifocal silicone hydrogel contact lens **Intervention 2:** spherical silicone hydrogel contact lens
Outcomes	**Primary outcomes:** cycloplegic autorefraction, AL **Secondary outcomes:** not reported **Maximum follow‐up:** not reported
Starting date	December 2011 **Estimated end date:** December 2015
Contact information	chictr.org.cn/hvshowproject.aspx?id=1766
Notes

**Table:** ChiCTR‐TRC‐13003396

Study name	Myopia progression with sedentary use, small segment, concentric bifocals
Methods	Randomised parallel‐group design
Participants	**Inclusion criteria:** children aged 6‐12 years, with spherical equivalent of −0.75 D to −3.50 D; astigmatism not greater than −1.50 D; normal ocular health; parental willingness to comply with the protocol; ability to consent **Exclusion criteria:** anisometropia ≤ 1.00 D; history of allergy to topical anaesthetics; strabismus; eye surgery; ocular or systemic conditions affecting vision; ocular injury; use of BFs, spectacles, OK, vision training, orthoptic training, or condition that affects ability to wear spectacles; concurrent participation in another clinical trial
Interventions	**Intervention:** intermittent alternate use of spectacles with concentric BF lenses and SVLs **Comparison intervention:** SVLs
Outcomes	**Primary outcome:** change in SER **Secondary outcome:** change in AL **Maximum follow‐up:** not reported
Starting date	August 2013 **Estimated end date:** March 2015
Contact information	chictr.org.cn/hvshowproject.aspx?id=6324
Notes

**Table:** ChiCTR‐TRC‐13004032

Study name	Chinese university low dose atropine for myopia progression study (CU‐LAMP)
Methods	Randomised parallel‐group design
Participants	**Inclusion criteria:** age 4‐12 years; myopia: SE −1 to −10 D; astigmatism: < 2.5 D; anisometropia: < 2.0 D; myopia progression > 1 D for BE in one year; informed parental consent **Exclusion criteria:** ophthalmic diseases other than refractive errors; previous use of treatment of atropine; allergy or intolerance to atropine; inability to attend regular follow‐up assessment
Interventions	**Intervention 1:** 0.05% atropine eye drops **Intervention 2:** 0.025% atropine eye drops **Intervention 3:** 0.01% atropine eye drops **Comparison intervention:** 0.9% normal saline eye drops
Outcomes	**Primary outcome:** SER (cycloplegic refraction); AL **Secondary outcomes:** safety variable: BCVA, pupil size, IOP **Maximum follow‐up:** not reported
Starting date	January 2014 **Estimated end date:** not reported
Contact information	chictr.org.cn/hvshowproject.aspx?id=14749
Notes

**Table:** ChiCTR‐TRC‐14004227

Study name	Assessment rate of progression of myopia with contact lenses in Chinese children
Methods	Randomised parallel‐group design
Participants	**Inclusion criteria:** 450 children aged 8‐12 years; Chinese ethnicity; myopic (short‐sighted); −0.75 D to −3.50 D of cycloplegic spherical equivalent with astigmatism no more than 0.75 D; preferably progressive myopia; ocular health findings considered to be “normal”; vision correctable to 6/9.5 or better in each eye with study contact lenses **Exclusion criteria:** pre‐existing ocular irritation that would preclude contact lens fitting; any systemic or ocular condition or ocular injury that may preclude safe wearing of contact lenses; having undergone corneal refractive surgery; keratoconus; allergy to or history of intolerance to tropicamide or topical anaesthetics; astigmatism > 0.75 D in either eye; past strabismus and/or current ongoing amblyopia; any ocular, systemic, or other condition or disease with possible associations with myopia or affecting refractive development; eye injury or surgery within 12 weeks immediately before enrolment for this trial; having undergone atropine treatment for myopia control; having worn BF or PAL spectacles or anti‐myopia contact lenses previously; having worn OK lenses previously; requiring anticholinergic medication for gastrointestinal or other conditions; anisometropic by > 1.50 D; current enrolment in another clinical trial/research project
Interventions	**Intervention 1:** Clariti contact lenses **Intervention 2:** Aquamax contact lenses
Outcomes	**Primary outcome:** myopia progression **Secondary outcomes:** not reported **Maximum follow‐up:** not reported
Starting date	5 February 2014 **Estimated end date:** 30 October 2017
Contact information	chictr.org.cn/hvshowproject.aspx?id=8971
Notes

**Table:** ChiCTR‐TRC‐14004990

Study name	Low‐concentration atropine to slow myopic progression in children
Methods	Randomised parallel‐group design
Participants	**Inclusion criteria:** 100 children aged 8‐12 years with myopia of spherical equivalent −1 D to −6 D; astigmatism < 1.5 D; anisometropia < 2D; BCVA > 0.8; IOP < 21 mmHg; myopia progression > 0.5 D in 1 year **Exclusion criteria:** ophthalmic disease other than refractive error or systematic disease; previous use of treatment of atropine, RGP, or OK; allergy or intolerance to atropine or tropicamide
Interventions	**Intervention:** 0.01% atropine eye drops **Comparison intervention:** placebo eye drops
Outcomes	**Primary outcome:** refraction **Secondary outcomes:** AL, pupil size, residue accommodation **Maximum follow‐up:** not reported
Starting date	July 2014 **Estimated end date:** not reported
Contact information	chictr.org.cn/showproj.aspx?proj=4584
Notes

**Table:** CTRI/2016/11/007450

Study name	Atropine eye drops to decrease myopia progression in children
Methods	Randomised parallel‐group design
Participants	**Inclusion criteria:** 40 children aged 6‐12 years; SER error between −2 D and −6 D in each eye; distance vision correctable to logMAR 0.2 or better in both eyes; normal ocular health other than myopia; informed consent; willingness to follow up **Exclusion criteria:** astigmatism > −1.5 D; amblyopia; strabismus; allergy to atropine or homatropine; previous or concurrent use of contact lenses, BFs, PALs or other forms of treatment for myopia; history of cardiac, neurological, or significant respiratory disease; unwillingness to give consent/follow‐up
Interventions	**Intervention 1:** 0.01% atropine eye drop **Comparison intervention:** 0.5% carboxymethylcellulose eye drop
Outcomes	**Primary outcome:** myopia progression **Secondary outcomes:** AEs **Maximum follow‐up:** 1 year
Starting date	January 2016 **Estimated end date:** not reported
Contact information	ctri.nic.in/Clinicaltrials/pdf_generate.php?trialid=15817&EncHid=&modid=&compid=%27,%2715817det%27
Notes

**Table:** CTRI/2019/05/018970

Study name	Atropine eyedrops for preventing increase in refractive error (shortsight and astigmatism)
Methods	Randomised paired eye design (within‐person study)
Participants	**Inclusion criteria:**. children or young adults in the age group 5‐15 years, myopic SER error > 1.00 D, astigmatism of > 1.50 D, documented progression of myopic component of compound myopic astigmatism; normal ocular health other than refractive error, normal IOP (< 21 mmHg) **Exclusion criteria:** allergy or hypersensitivity to atropine, cyclopentolate, phenylephrine or proparacaine, amblyopia in at least one eye; history of significant cardiac or respiratory illness, no previous or current use of contact lenses or BFs or progressive lenses or other forms of treatment (including atropine in any strength) for myopia
Interventions	**Intervention:** atropine 0.01% eyedrops **Comparison intervention:** atropine eyedrops will be used only in 1 eye.The other eye will receive no treatment.
Outcomes	**Primary outcome:** progression of the myopic component of compound myopic astigmatism estimated as the change in SER error relative to the baseline **Secondary outcome:** change in AL relative to the baseline **Maximum follow up:** 12 months
Starting date	May 2019 **Estimated end date:** not reported
Contact information	ctri.nic.in/Clinicaltrials/pmaindet2.php?trialid=27716
Notes

**Table:** CTRI/2019/10/021538

Study name	Atropine eyedrops for treatment of increasing shortsight
Methods	Randomised paired eye design (within‐person study)
Participants	**Inclusion criteria:**. children or young adults in the age group 5‐15 years, myopic SER error > 1.00D, astigmatism of < 1.50 D; documented progression of myopia, normal ocular health other than refractive error, normal IOP (< 21 mmHg) **Exclusion criteria:** known allergy or hypersensitivity to atropine, cyclopentolate, phenylephrine or proparacaine, amblyopia in at least 1 eye, history of significant cardiac or respiratory illness, no previous or current use of contact lenses or BFs or progressive lenses or other forms of treatment (including atropine in any strength) for myopia
Interventions	**Intervention:** atropine 0.01% eyedrops **Comparison intervention:** other eye is not treated and will be used as the comparator to the treated eye
Outcomes	**Primary outcome:** progression of the myopic component of compound myopic astigmatism estimated as the change in SER error relative to baseline **Secondary outcome:** change in AL relative to baseline **Maximum follow up:** 12 months
Starting date	October 2019 **Estimated end date:** not reported
Contact information	ctri.nic.in/Clinicaltrials/pmaindet2.php?trialid=27712
Notes

**Table:** CTRI/2021/10/037447

Study name	Role of 0.01% atropine in myopia control of high myopic children of Moradabad (India)
Methods	Randomised parallel‐group design
Participants	**Inclusion criteria:** age 6‐16 years, myopia ≥ 5.00 D (spherical equivalent), no prior or current treatment for preventing myopia progression **Exclusion criteria:** BCVA < 0.5 (6/12), astigmatism ≥ 1.50 D, amblyopia; ocular hypertension/glaucoma, prior intraocular surgery, allergy to atropine eye drops, systemic diseases associated with myopia such as Marfan syndrome, Stickler syndrome, history of cardiac or significant respiratory diseases
Interventions	**Intervention:** 1 drop of atropine 0.01% eyedrops **Comparison intervention:** no drug or placebo
Outcomes	**Primary outcome:** progression of myopia in dioptres (spherical equivalent relative to baseline) **Secondary outcome:** change in AL **Maximum follow up:** 36 months
Starting date	November 2021 **Estimated end date:** not reported
Contact information	ctri.nic.in/Clinicaltrials/pmaindet2.php?trialid=60934
Notes

**Table:** EUCTR2016‐003340‐37‐IE

Study name	Myopia outcome study of atropine in children (MOSAIC)
Methods	Randomised parallel‐group design
Participants	**Inclusion criteria:** age 6–16 years old, cycloplegic SER ≥ −1.00, astigmatism ≤ 2.50 D and the least myopic meridian must be more myopic or equal to −0.50D, anisometropia ≤ 1.50 D, corrected VA of 0.2 logMAR or better in both eyes, normal IOP (≤ 21 mmHg), normal ocular health, good general health **Exclusion criteria:** strabismus or amblyopia, previous pharmaceutical or optical myopia control interventions, previous allergy to atropine, cyclopentolate HCl or proxymetacaine HCl
Interventions	**Intervention:** 0.01% atropine eye drops **Comparison intervention:** placebo
Outcomes	**Primary outcome:** change in SER at 24 months measured by cycloplegic auto‐refraction **Secondary outcomes:** change in ocular AL at 24 months measured by optical low‐coherence interferometry, change in SER and AL at 12 months, percentage of participants who progress < 0.25 D (dioptre), 0.25 D ≤ 0.75 D and > 0.75 D in 24 months, rebound acceleration in myopic refractive error after cessation of atropine treatment, measured as change in SER and AL between 24 and 36 months, QoL impact associated with atropine use at 24 months, frequency of AEs recorded on study‐specific report forms **Maximum follow up:** 24 months
Starting date	October 2017 **Estimated end date:** May 2023
Contact information	clinicaltrialsregister.eu/ctr‐search/trial/2016‐003340‐37/IE
Notes

**Table:** EUCTR2018‐001286‐16‐DK

Study name	Low‐dose atropine for the prevention of nearsightedness in Danish children
Methods	Randomised parallel‐group design
Participants	**Inclusion criteria:** children aged 6‐9 years: myopia = −1 (spherical equivalent) in at least 1 eye; children aged 9‐12 years: myopia = −2 (spherical equivalent) in at least 1 eye; cylinder < 1.5 D **Exclusion criteria:** myopia related to retinal dystrophies; collagen syndromes (Ehlers‐Danlos syndrome, Marfan syndrome and Stickler syndrome); other ocular pathology (e.g. amblyopia, strabismus); previous eye surgery; previous use of agents thought to affect myopia progression, e.g. atropine, pirenzepine or 7‐mx (metabolite of caffeine and theobromine) and OK contact lenses; known allergy to atropine or any of the contents of the study medication (active and inactive ingredients) used in the study; non‐compliance to eye examinations; serious systemic health troubles (e.g. cardiac or respiratory illness) and developmental disorders and delays
Interventions	**Intervention 1**: atropine 0.01% **Comparison intervention 1**: atropine 0.1% **Comparison intervention 2**: placebo eye drops
Outcomes	**Primary outcome**: AL elongation; change in spherical equivalent **Secondary outcomes**: patient reported outcome; AEs and reactions; change in choroidal thickness; change in ocular biometry (i.e. keratometry, anterior chamber depth, lens thickness, vitreous axial distance); change in higher‐order aberrations **Maximum follow‐up**: 36 months
Starting date	Not reported
Contact information	Clinicaltrialsregister.eu/ctr‐search/trial/2020‐001052‐18/DK
Notes

**Table:** EUCTR2019‐002535‐28‐FR

Study name	Braking effect on myopia with atropine eye drops at 0.01%
Methods	Randomised parallel‐group design
Participants	**Inclusion criteria:** children age 4‐12 years, myopia between −1.00 D and −6.00 D, progressive myopia characterised by a minimal rate of progression of −0.75 D in the last 12 months **Exclusion criteria:** astigmatism > 1.5 D, anisometropy > 2 D, presence of an ocular pathology, strabismus or disturbance of stereoscopic vision, amblyopia, contraindication to the use of the investigational medicinal product and/or to the explorations provided for in the protocol, hypersensitivity to atropine or any of the excipients of the eye drops present in the raw material
Interventions	**Intervention:** 0.01% atropine eyedrops **Comparison intervention:** placebo **Maximum follow up:** 12 months
Outcomes	**Primary outcome:** degree of myopia measured in spherical dioptres **Secondary outcome:** change in SER and AL from baseline, total macular and choroidal thickness, pupil diameter, accommodation, AEs
Starting date	November 2021 **Estimated end date:** not reported
Contact information	trialsearch.who.int/Trial2.aspx?TrialID=EUCTR2019‐002535‐28‐FR
Notes

**Table:** EUCTR2020‐001575‐33‐DE

Study name	Low‐dose atropIne for myopia control in children
Methods	Randomised parallel‐group design
Participants	**Inclusion criteria:** aged 8‐12 years, myopia −1.00 D to −6.00 D, reported or documented annual progression = 0.5 D of myopia **Exclusion criteria:** Asian or African origin, abnormal binocularity, strabismus, astigmatism > 1.5 D, anisometropia > 1.5 D, history of amblyopia, corrected VA in any eye < 0.63, any acquired or developmental organic eye disease, premature birth, any known systemic metabolic disease or chromosomal anomaly, previous use of any kind of contact lenses, previous use of atropine eye drops, epilepsy, known hypersensitivity to the active substances or any of the excipients
Interventions	**Intervention:** atropine 0.01% eyedrops **Intervention:** atropine 0.02% eyedrops **Comparison intervention:** placebo
Outcomes	**Primary outcome:** change in SER error relative to baseline **Secondary outcome:** change in AL relative to baseline **Maximum follow up:** 12 months
Starting date	June 2021 **Estimated end date:** not reported
Contact information	trialsearch.who.int/Trial2.aspx?TrialID=EUCTR2020‐001575‐33‐DE
Notes

**Table:** EUCTR2020‐002046‐16‐CZ

Study name	A randomised, double‐blinded, placebo‐controlled, multicenter study of efficacy, safety and side effects of highly diluted atropine collyrium in slowing the progression of myopia (shortsightedness) in children
Methods	Randomised paired‐eye design
Participants	**Inclusion criteria:** age 6‐12 years, myopia ‐ spherical component of refraction −0.50 D to −4.75 D, astigmatism 0 to −2.5 D in both eyes, distance BCVA of worse eye better or equal to 0.2 logMAR (according to EDTRS), normal ocular findings, normal binocular functions, normal IOP, axial growth at 6 months in the pre‐randomisation period of the study (6‐7 years 0.10 mm, 8‐9 years 0.11 mm, 10‐11 years 0.12 mm) **Exclusion critera:** general diseases with myopia (Marfan's, Stickler's syndrome) or affecting visual functions (diabetes mellitus, chromosomal anomalies), previous pharmacological, surgical and/or OK therapy of myopia, previous long‐term treatment with atropine, presence and/or history of allergic reaction to ophthalmologics (atropine; cycloplegics ‐ cyclopentolate, tropicamide; local anaesthetics ‐ e.g. oxybuprocaine, etc.), presence of strabismus, amblyopia, glaucoma, corneal damage and/or scarring and current and/or previous ocular conservative, contactology and/or surgical therapy; presence and/or history of general disease (including allergy, myasthenia gravis, cardiac, respiratory and/or renal‐urological disease and/or dysfunction)
Interventions	**Intervention:** 0.02% atropine eye drops **Intervention:** 0.04% atropine eye drops **Comparison intervention:** placebo
Outcomes	**Primary outcome:** difference in AL (0.02% atropine vs placebo) **Secondary outcome:** difference in AL (0.04% atropine vs placebo, 0.04% atropine vs 0.02% atropine), SER, rebound in both arms, QoL, AEs, distance BCVA, contrast sensitivity **Maximum follow up:** 36 months
Starting date	Not reported
Contact information	trialsearch.who.int/Trial2.aspx?TrialID=EUCTR2020‐002046‐16‐CZ
Notes

**Table:** EUCTR2020‐003976‐42‐NL

Study name	A large scale study to confirm and expand the information on the safety and effectiveness of atropine in treating the progression of myopia in pediatric subjects
Methods	Randomised parallel‐group design
Participants	**Inclusion criteria:** aged 3‐15 years of either sex and any race or ethnicity, myopia between −1.00 D and −6.00D, astigmatism ≤ 1.50 D, anisometropia = 1.0 D, distance BCVA) of logMAR = 0.4 (approximately Snellen 20/50) for 3‐year‐olds; logMAR = 0.3 (approximately Snellen 20/40) for 4‐year‐olds; logMAR = 0.18 (approximately Snellen 20/30) for 5‐year‐olds) in each eye **Exclusion critera:** known contraindications or sensitivity to atropine, clinically significant abnormal findings on slit lamp biomicroscopy exam, clinically significant abnormal findings on indirect dilated fundoscopy exam in either eye at screening or a known history of a clinically significant retinal findings in either eye, evidence of an eye movement disorder or restriction of extraocular movement (e.g. nystagmus), have undergone any myopia control treatment including atropine, OK, RPGs, BF contact lenses, PAL spectacles, or other lenses to reduce myopia progression in the previous 6 months, myopic correction in the form of SVLs and/or SVSCLs are allowed, have undergone any form of refractive eye surgery cataract extraction, or any form of intraocular lens implantation, IOP < 9 mmHg or > 21 mmHg in either eye, or have a prior diagnosis of ocular hypertension or glaucoma; surgical intervention (ocular or systemic) within 6 months prior to initial visit or planned surgical intervention during the study
Interventions	**Intervention:** 0.01% atropine eyedrops **Comparison intervention:** placebo
Outcomes	**Primary outcome:** percentage of study eyes with a −0.75 D of progressive myopia, safety and tolerability of 0.01% atropine **Secondary outcome:** change from baseline in study eye spherical equivalent (D), change from baseline in study eye AL **Maximum follow up:** 36 months
Starting date	Not reported
Contact information	trialsearch.who.int/Trial2.aspx?TrialID=EUCTR2020‐003976‐42‐NL
Notes

**Table:** EUCTR2021‐003373‐64‐ES

Study name	Clinical trial with DIMS lenses for the control of myopia in pediatric population
Methods	Randomised paired‐eye design (within‐person study)
Participants	**Inclusion criteria:** aged 4‐16 years, myopia > −1.00 D, progression of myopia of at least −0.50 D in the last 12 months, astigmatism ≤ 2 D, anisometropia of ≤ 1.50 D, monocular BCVA of 0.2 logMAR (6/9) or better **Exclusion criteria:** strabismus and binocular vision abnormalities, ocular pathology of the anterior segment (opacity of media such as cataracts, glaucoma, aphakia, pseudophakia, uveitis, keratoconus or surface alterations) and any pathology of the posterior segment that prevents correct vision, previous eye surgery, amblyopia, systemic pathology (cardiopulmonary pathology, connective tissue disorders, neurological or psychiatric disorders), previous treatments for the control of myopia, including OK, rigid contact lenses, BFSCLs or for the control of myopia, BF and MF ophthalmic lenses in the 3 months prior to the study
Interventions	**Intervention:** DIMS spectacle lenses and 0.01% atropine eyedrops **Comparison intervention:** DIMS spectacle lenses
Outcomes	**Primary outcome:** degree of myopia **Secondary outcome:** AL, choroidal and retinal thickness, IOP **Maximum follow up:** 24 months
Starting date	November 2021 **Estimated end date:** not reported
Contact information	trialsearch.who.int/Trial2.aspx?TrialID=EUCTR2021‐003373‐64‐ES
Notes

**Table:** IRCT20100414003714N3

Study name	Study of the effect of atropine eye drops with concentration of 0.1% & 0.01% and placebo in natural course of myopia progression in children 6 to 18 years old
Methods	Randomised parallel‐group design
Participants	**Inclusion criteria:** aged 6‐18 years; myopia or astigmatism (2‐6 D); no amblyopia **Exclusion criteria:** strabismus
Interventions	**Intervention 1:** 0.1% atropine eye drops for 12 months **Intervention 2:** 0.11% atropine eye drops for 12 months **Comparison intervention:** artificial eye drops for 12 months
Outcomes	**Primary outcomes:** percentage of myopic power, AL changes **Secondary outcomes:** not reported **Maximum follow‐up:** 6 months
Starting date	June 2018 **Estimated end date:** December 2019
Contact information	en.irct.ir/trial/31944
Notes

**Table:** IRCT20180216038747N1

Study name	Controlling myopia progression
Methods	Not reported
Participants	**Inclusion criteria:** myopia −0.50 D to −6.00 D; astigmatism ≤ 0.75 D **Exclusion criteria:** myopic children with any ocular disease such as cataract, glaucoma, uveitis, strabismus; history of trauma; history of any ocular surgery systemic disease
Interventions	**Intervention 1:** 0.01% atropine eye drops for 1 year **Intervention 2:** 0.02% atropine eye drops for 1 year **Comparison intervention:** artificial tear drops for 1 year
Outcomes	**Primary outcomes:** AL of the eye, accommodation amplitude, pupil size **Secondary outcomes:** not reported **Maximum follow‐up:** 12 months
Starting date	April 2018 **Estimated end date:** May 2019
Contact information	www.irct.ir/trial/30096
Notes

**Table:** ISRCTN36732601

Study name	Efficacy, safety, and mechanisms of atropine eye drops in slowing the progression of shortsightedness (myopia) in children
Methods	Randomised cross‐over design (within‐person study)
Participants	**Inclusion criteria:** 250 children aged 6‐16 years; myopia of −1.0 D or worse in each eye; astigmatism refractive error < −1.50 D; progressive myopia of at least −0.50 D over the last year; intraocular difference in spherical difference ≤ 1.00 D; corrected VA ≥ logMAR 0.2 in both eyes; normal IOP; normal ocular health **Exclusion criteria:** ocular or systemic disease affecting vision; allergy to study‐related drugs; defective binocular vision; previous pharmaceutical or optical myopia control interventions
Interventions	**Intervention:** 0.01% atropine eye drops **Comparison intervention:** placebo eye drops
Outcomes	**Primary outcome:** SER **Secondary outcomes:** AL, off‐axis refraction, ocular growth, visual performance, ocular function, QoL, AEs **Maximum follow‐up:** 24 months
Starting date	October 2017 **Estimated end date:** May 2023
Contact information	isrctn.com/ISRCTN36732601
Notes

**Table:** JPRN‐jRCTs032200060

Study name	Comparison of myopia control effects by combination therapy with multifocal SCL and atropine 0.01% ophthalmic solution, multifocal SCL monotherapy, combination therapy with sphere SCL and atropine 0.01% ophthalmic solution, versus sphere SCL monotherapy: a 1‐year randomized four‐armed clinical trial in myopic schoolchildren
Methods	Randomised parallel‐group design
Participants	**Inclusion criteria:** children aged 6‐12, equivalent spherical power in the range of −1.00 D to −6.00 D, difference in equivalent spherical power between the left and right eyes within 1.50 D, astigmatic power is within ±1.00 D **Exclusion criteria:** abnormal binocular function, amblyopia, corrected VA measured with glasses is < 1.0, abnormal IOP, eye‐related diseases other than myopia, eye‐related or systemic disorders that may affect VA or power, children receiving or who have received myopia treatment
Interventions	**Intervention:** MF contact lenses plus atropine 0.01% eyedrops **Intervention:** MF contact lenses plus placebo **Intervention:** single focus contact lenses plus atropine 0.01% eyedrops **Comparison intervention:** single focus contact lenses plus placebo
Outcomes	**Primary outcome:** difference in axial elongation and myopia progression **Secondary outcome:** change in SER and AL from baseline **Maximum follow up:** 12 months
Starting date	August 2021 **Estimated end date:** not reported
Contact information	trialsearch.who.int/Trial2.aspx?TrialID=JPRN‐jRCTs032200060
Notes

**Table:** JPRN‐jRCTs051180041

Study name	The efficacy of 0.01% atropine ophthalmic solution for controlling the progression of childhood myopia (ATOM‐J Study)
Methods	Randomised parallel‐group design
Participants	**Inclusion criteria:** children aged 6‐12 years, spherical equivalent myopia of −1.00 D to −6.00 D in each eye, anisometropia within 1.50 D, astigmatism within +/‐ 1.50 D, corrected VA of at least 1.0 **Exclusion criteria:** abnormal visual function, amblyopia or manifest strabismus, ocular disorders other than myopia, ocular or systemic disorders that potentially affect myopia or refractive power, previous treatment for myopia that included atropine therapy such as contact lenses, BF lenses, or progressive lenses with atropine therapy, history of cardiovascular or respiratory disease, pharmacotherapy for asthma in the past year, history of allergy to atropine, cyclopentolate, or benzalkonium
Interventions	**Intervention:** atropine 0.01% eyedrops **Comparison intervention:** placebo **Maximum follow up:** 24 months
Outcomes	**Primary outcome:** change in SER from baseline **Secondary outcome:** change in AL from baseline; incidence rate of AEs and side effects, accommodative function, IOP
Starting date	August 2015 **Estimated end date:** not reported
Contact information	trialsearch.who.int/Trial2.aspx?TrialID=JPRN‐jRCTs051180041
Notes

**Table:** JPRN‐jRCTs061180091

Study name	Effect of 0.01% atropine eye drops in children with moderate to high grade myopia
Methods	Randomised parallel‐group design
Participants	**Inclusion criteria:** age 5‐12 years, spherical equivalent myopia −4.50 to −9.00 D, anisometropia ≤ 1.50 D, astigmatism ≤ 1.50 D, BCVA ≥ 1.0, IOP ≤ 21 mmHg **Exclusion criteria:** abnormal binocular function, amblyopia or manifest strabismus, eye diseases besides myopia, ophthalmic and/or systematic diseases that may influence VA or refractive error, previous history of using atropine, contact lenses, BF or PAL, or OK, eye or general diseases that may affect myopia progression, history of asthma treatment within 1 year, allergic history to atropine, cyclopentolate, or benzalkonium
Interventions	**Intervention:** atropine 0.01% eyedrops **Comparison intervention:** placebo **Maximum follow up:** not reported
Outcomes	**Primary outcome:** change in SER and AL between baseline and final visit **Secondary outcome:** occurrence of AEs
Starting date	March 2018 **Estimated end date:** not reported
Contact information	trialsearch.who.int/Trial2.aspx?TrialID=JPRN‐jRCTs061180091
Notes

**Table:** JPRN‐UMIN000005054

Study name	Clinical trial to evaluate effect of spectacle lens that reduces myopia progression
Methods	Randomised parallel‐group design
Participants	**Inclusion criteria:** 6‐12 years of age; myopic refractive error between −1.50 D and −4.50 D; astigmatism < 1.5 D; BCVA 1.0 or better; father or mother with myopia **Exclusion criteria:** strabismus; having worn BFs or PALs in previous year; history of OK lens wear; prior participation in myopia studies; any eye disease other than myopia
Interventions	**Intervention:** eyeglasses that reduce myopic progression **Control:** normal eyeglasses
Outcomes	Not reported
Starting date	February 2011 **Estimated end date:** not reported
Contact information	Takeshi Morimoto Department of Applied Visual Science Osaka University School of Medicine 2‐2 Yamadaoka, Suita, Osaka, Japan email: takeshi.morimoto@ophthal.med.osaka‐u.ac.jp umin.ac.jp/ctr/index.htm
Notes

**Table:** JPRN‐UMIN000007989

Study name	Clinical trial to prevent myopia progression by progressive additional soft contact lens compared with monofocal soft contact lens in children
Methods	Randomised cross‐over design
Participants	**Inclusion criteria:** 20 children age 6‐16 years; refractive error −0.75 D to −3.5 D; corrected VA by spherical spectacle lens: better than (0.7) **Exclusion criteria:** anisometropia > 1.0 D; amblyopia; strabismus
Interventions	**Intervention:** wearing progressive additional soft contact lens **Comparison intervention:** wearing monofocal soft contact lens
Outcomes	**Primary outcome:** ocular refraction **Secondary outcome:** AL **Maximum follow‐up:** not reported
Starting date	January 2011 **Estimated end date:** not reported
Contact information	upload.umin.ac.jp/cgi‐open‐bin/ctr/ctr.cgi?function=brows&action=brows&recptno=R000009401&type=summary&language=E
Notes

**Table:** JPRN‐UMIN000013698

Study name	Examination of the nearsighted progress depression effect of the low‐concentrated atropine in the Japanese primary school child
Methods	Randomised parallel‐group design
Participants	**Inclusion criteria:** 90 children aged 6‐12 years with no eye disease except refractive error **Exclusion criteria:** children with contact lens; history of myopia progress suppression treatment
Interventions	**Intervention 1:** 0.01% atropine eye drops **Intervention 2:** 0.025% cyclopentolate eye drops **Comparison intervention:** raw diet instillation
Outcomes	**Primary outcomes:** refractive error, AL **Secondary outcomes:** not reported **Maximum follow‐up:** not reported
Starting date	March 2014 **Estimated end date:** August 2017
Contact information	upload.umin.ac.jp/cgi‐open‐bin/ctr/ctr.cgi?function=brows&action=brows&type=summary&recptno=R000015991&language=E
Notes

**Table:** JPRN‐UMIN000014362

Study name	Examination of suppressive effect by combined treatment of OK and atropine 0.01% ophthalmic solution on myopia progression
Methods	Randomised parallel‐group design
Participants	**Inclusion criteria:** cycloplegic SER error of −1.00 to −6.00 D in both eyes; astigmatism of < 1.50 D in both eyes; anisometropia of < 1.50 D; BCVA of > 1.0 in both eyes **Exclusion criteria:** eye disorders such as strabismus and amblyopia; systemic disorders such as cardiac or respiratory illness; birth weight of < 1500 g; history of hypersensitivity to atropine, use of OK and/or atropine ophthalmic solutions
Interventions	**Intervention:** OK contact lens **Comparison intervention:** atropine 0.01% ophthalmic solution
Outcomes	**Primary outcomes:** AL **Secondary outcomes:** corneal endothelial cell density; corneal endothelial cell density **Maximum follow‐up:** 2 years
Starting date	June 2014 **Estimated end date:** March 2019
Contact information	rctportal.niph.go.jp/en/detail?trial_id=UMIN000014362
Notes

**Table:** JPRN‐UMIN000018041

Study name	The efficacy of 0.01% atropine ophthalmic solution for controlling the progression of childhood myopia (ATOM‐J Study)
Methods	Randomised parallel‐group design
Participants	**Inclusion criteria:** 180 children aged 6‐12 years; decrease in VA within the past year; cycloplegic objective spherical equivalent of −1.00 D to −6.00 D in each eye; anisometropia within 1.50 D; astigmatism within ± 1.50 D; corrected VA of at least 1.0; no IOP abnormalities; capable of undergoing cycloplegia **Exclusion criteria:** abnormal visual function; amblyopia or manifest strabismus; difference in objective spherical equivalent with and without cycloplegia > 1.00 D in each eye; ocular disorders other than myopia; ocular or systemic disorders that potentially affect myopia or refractive power; previous treatment for myopia including atropine therapy, contact lenses, BFs, or progressive lenses with atropine therapy (does not apply to children who discontinued 0.4% tropicamide ophthalmic solution at least 3 months previously); history of cardiovascular or respiratory disease; children who have received pharmacotherapy for asthma in the past year; allergy to atropine, cyclopentolate, or benzalkonium; children who cannot instil medication into the eye, requiring contact lenses, BF lenses, or progressive lenses during the clinical study period
Interventions	**Intervention:** 0.01% atropine ophthalmic solution **Comparison intervention:** placebo ophthalmic solution
Outcomes	**Primary outcome:** change in objective spherical equivalent **Secondary outcome:** none reported **Maximum follow‐up:** 24 months
Starting date	July 2015 **Estimated end date:** not reported
Contact information	apps.who.int/trialsearch/Trial2.aspx?TrialID=JPRN‐UMIN000018041
Notes

**Table:** JPRN‐UMIN000019237

Study name	Effect of dual‐focus soft contact lens wear on myopia progression
Methods	Randomised parallel‐group design
Participants	**Inclusion criteria:** 28 children aged 10‐14 years; no previous wearing of contact lenses; −1.0 D to −6.0 D refraction in each eye under non‐accommodative palsy; total astigmatism dioptre within −1.5 D in each eye; corrected VA > 1.0 D in each eye; no eye misalignment; not a premature infant; no ocular or systemic maldevelopment; no drug use; ability to wear contact lens for 1 week **Exclusion criteria:** as deemed appropriate by study investigators
Interventions	**Intervention:** BF contact lenses **Comparison intervention:** spectacles
Outcomes	**Primary outcomes:** refractivity, optic axis length **Secondary outcomes:** not reported **Maximum follow‐up:** not reported
Starting date	May 2015 **Estimated end date:** not reported
Contact information	rctportal.niph.go.jp/en/detail?trial_id=UMIN000019237
Notes

**Table:** JPRN‐UMIN000023386

Study name	Clinical trial on the use of outdoor environment glasses for a suppressive effect on myopia progression
Methods	Randomised parallel‐group design
Participants	**Inclusion criteria:** 140 children, aged 6‐12 years; paralysis of accommodation in both eyes; spherical equivalent of each is between −1.50 D and 4.50 D; at least 1 parent who has myopia; no eye disease other than refractive error **Exclusion criteria:** history of wearing BF or progressive power lenses; history of wearing OK lenses; unequal parallax > 1.50 D; astigmatism > 1.50 D; overt strabismus; received refractive surgery in the past; keratoconus or herpes conjunctivitis; papillary proliferation; participating in other similar clinical research
Interventions	**Intervention:** wearing outdoor environment glasses **Comparison intervention:** wearing normal glasses
Outcomes	**Primary outcome:** change in AL **Secondary outcome:** none reported **Maximum follow‐up:** 24 months
Starting date	July 2016 **Estimated end date:** not reported
Contact information	upload.umin.ac.jp/cgi‐open‐bin/ctr_e/ctr_view.cgi?recptno=R000026874
Notes

**Table:** JPRN‐UMIN000027940

Study name	Clinical study on the effect of multifocal contact lens on myopia progression in myopia school children
Methods	Randomised parallel‐group design
Participants	**Inclusion criteria:** 100 children aged 6‐12 years; moderate myopia (objective equivalent spherical power −1.00 D to −6.00 D) **Exclusion criteria:** anisometropia; astigmatism beyond 1.5 D
Interventions	**Intervention:** multifocal contact **Comparison intervention:** normal contact
Outcomes	**Primary outcome:** refractive power change **Secondary outcome:** change in AL **Maximum follow‐up:** not reported
Starting date	August 2017 **Estimated end date:** not reported
Contact information	upload.umin.ac.jp/cgi‐open‐bin/ctr/ctr_view.cgi?recptno=R000032004
Notes

**Table:** Kinoshita 2018

Study name	Additive effects of orthokeratology and atropine 0.01% ophthalmic solution in slowing axial elongation in children with myopia: first year results
Methods	Randomised parallel‐group design
Participants	**Inclusion criteria:** aged 8‐12; SER error of −1.00 to −6.00 D **Exclusion criteria:** not reported
Interventions	**Intervention:** OK and atropine 0.01% ophthalmic solution **Comparison intervention:** OK
Outcomes	**Primary outcome:** AL **Secondary outcomes:** not reported **Maximum follow‐up:** 1 year
Starting date	Not reported **Estimated end date:** not reported
Contact information	Nozomi Kinoshita, Department of Ophthalmology, Saitama Medical Center, Jichi Medical University, 1‐847 Amanuma‐cho, Omiya‐ku, Saitama‐shi, Saitama, 330‐8503, Japan. Email: nozomik@omiya.jichi.ac.jp
Notes

**Table:** Li 2013

Study name	The full correction and undercorrection of myopia evaluation trial (FUMET)
Methods	Randomised parallel‐group design
Participants	**Inclusion criteria:** 7‐15 years of age; 6/6 or better in each eye; spherical error between −1.5 and −6.0 D; astigmatism < 1.5 D in each eye; anisometropia < 1.0 D between the 2 eyes; no history of contact lens use, strabismus, amblyopia, or other ocular and systematic disease that influences refractive growth **Exclusion criteria:** inability to live close to study centre for 2 years; inability to co‐operate with examinations or surveys; allergy to mydriatic drugs; use of other treatments to prevent myopia progression
Interventions	**Intervention:** full correction **Intervention comparison:** undercorrection (blurred by +0.5 D)
Outcomes	**Primary outcomes:** change in cycloplegic autorefraction; change in AL after 2 years **Secondary outcomes:** not specified **Maximum follow‐up:** 2 years
Starting date	November 2010 **Estimated end date:** January 2013
Contact information	Professor Ning‐Li Wang, Beijing Tongren Eye Center, Beijing Tongren Hospital, Capital Medical University, Beijing 100730, China. Email: wningli@vip.163.com
Notes	Registration number ChiCTR‐TRC‐10001122 Funding source: grants from “Major State Basic Research Development Program of China (‘973’ Program, 2011CB504601) of the Ministry of Science and Technology”; “Major International (Regional) Joint Research Project (81120108807) of the National Natural Science Foundation of China”; “China Postdoctoral Science Foundation (20110490247)”; Research Foundation of Beijing Tongren Hospital Affiliated to Capital Medical University (2012‐YJJ‐019)”

**Table:** Li 2020

Study name	Evaluating the myopia progression control efficacy of defocus incorporated multiple segments (DIMS) lenses and Apollo progressive addition spectacle lenses (PALs) in 6‐ to 12‐year‐old children: a prospective, multi‐center, randomized controlled trial
Methods	Randomised parallel‐group design
Participants	**Inclusion criteria:** age 6–12 years, cycloplegic SER −1.00 to −4.00 D, astigmatism ≤ 1.50 D, anisometropia ≤ 1.50 D, difference between the right and left pupil sizes ≤ 2 mm, monocular BCVA 20/20 (0.0 logMAR) or better **Exclusion criteria:** strabismus, any ocular and systemic diseases, including abnormalities, that might affect visual functions or refractive development; previous experience with myopia control, including OK, PAL spectacles, BF lenses, and pharmaceutical treatment (e.g.atropine)
Interventions	**Intervention:** DIMS spectacle lenses **Comparison intervention:** PAL spectacles
Outcomes	**Primary outcome:** difference in the subjective SER between baseline and the last follow‐up visit **Secondary outcome:** difference in AL between baseline and the last follow‐up visit **Maximum follow up:** 36 months
Starting date	October 2019 **Estimated end date:** not reported
Contact information	www.chictr.org.cn/showproj.aspx?proj=42927.
Notes	Trial registration ChiCTR1900025645

**Table:** MASS 2018

Study name	MiSight assessment study Spain (MASS)
Methods	Randomised parallel‐group design
Participants	**Inclusion criteria:** aged 8‐12 with myopia (−0.75 to −4.00 D sphere) and astigmatism (< −1.00 D cylinder) **Exclusion criteria:** current or prior contact lenses wear; current or prior use of BFs, PALs, atropine, pirenzepine, or any other myopia control treatment; regular use of ocular medications and artificial tears; current use of systemic medications, which may significantly affect contact lens wear, tear film production, pupil size, accommodation, or refractive state; known allergy to fluorescein, benoxinate, proparacaine, or tropicamide; history of corneal hypoesthesia, corneal ulcer, corneal infiltrates, ocular viral or fungal infection, or other recurrent ocular infection; strabismus by cover test at far (4 m) or near (40 cm); wearing distance correction; systemic or ocular disease affecting ocular health; keratoconus or an irregular cornea; CCLRU grade ≥ 2 for any given anterior segment ocular clinical signs; having pathological myopia; connective tissue disorder
Interventions	**Intervention:** lens study group (MiSight) **Comparison intervention:** control group (single vision)
Outcomes	**Primary outcomes:** VA, subjective refraction **Secondary outcomes:** AL, anterior chamber, corneal power, cycloplegic autorefraction **Maximum follow‐up:** 24 months
Starting date	September 2013 **Estimated end date:** June 2016
Contact information	Alicia Ruiz‐Pomeda, Department of Pharmacy, Biotechnology, Optics and Optometry, European University of Madrid, C/Tajo s/n, Villaviciosa de Odón, 28670, Madrid, Spain. Email: alicia.ruiz@universidadeuropea.es.
Notes

**Table:** NCT00214487

Study name	Bifocal soft contact lenses and their effect on myopia progression in children and adolescents
Methods	Randomised parallel‐group design
Participants	**Inclusion criteria:** myopia between −0.50 and −6.00; eso fixation disparity at 33 cm with distance correction; astigmatism ≤ 1.00; ability to wear soft contact lenses **Exclusion criteria:** presence of ocular disease preventing wear of contacts; pregnancy or nursing; use of certain medications
Interventions	**Intervention:** BF contact lenses **Comparison intervention:** SVSCLs
Outcomes	**Primary outcomes:** cycloplegic autorefraction, cycloplegic subjective refraction, AL **Secondary outcomes:** keratometric changes, manifest refraction **Maximum follow‐up:** 1 year
Starting date	October 2003 **Estimated end date:** March 2006
Contact information	clinicaltrials.gov/ct2/show/record/NCT00214487
Notes

**Table:** NCT00627874

Study name	Trial of myopia prevention using +3 D lenses (PLS)
Methods	Randomised parallel‐group design
Participants	**Inclusion criteria:** 1200 children (age reported), with juvenile‐onset myopia **Exclusion criteria:** hyperopia > +2.0 D; high myopia > −6.0 D; astigmatism > 1.5 D; anisometropia > 1.5 D; strabismus and amblyopia; any ocular, systemic, or neurodevelopmental conditions that could influence refractive development; chronic medication use that might affect myopia progression or VA; already receiving other treatment for progressing myopia
Interventions	**Intervention:** +3 D lenses **Comparison intervention:** not reported
Outcomes	**Primary outcome:** AL of eyes **Secondary outcome:** autorefraction **Maximum follow‐up:** not reported
Starting date	April 2010 **Estimated end date:** April 2012
Contact information	clinicaltrials.gov/ct2/show/NCT00627874
Notes

**Table:** NCT00762970

Study name	Controlling myopia progression with soft contact lenses
Methods	Randomised parallel‐group design
Participants	**Inclusion criteria:** myopic children aged 8‐12 years; best sphere contact lens correction must lie between −0.75 D (best of the 2 eyes) and −5.00 D (worst of the 2 eyes); astigmatism must be ≤ 1.00 D; 1.00 D or less difference in spherical equivalent between the 2 eyes; BCVA of 0.8 + 2 (20/25 + 2); SER VA of 0.820/25 or better in both eyes; at least 8 D of accommodation **Exclusion criteria:** any ocular or systemic allergy or disease that may interfere with contact lens wear; systemic disease or autoimmune disease or use of medication (e.g. antihistamine) that may interfere with lens wear; clinically significant (grade 3 or 4) abnormality of the cornea that may contraindicate contact lens wear; clinically significant (grade 3 or 4) tarsal abnormalities or bulbar injection; any ocular infection; any corneal distortion; any infectious disease (e.g. hepatitis, tuberculosis) or immunosuppressive disease (e.g. HIV); diabetes; anisometropia > 1.00 D; astigmatism > 1.00 D in either eye; eye injury or surgery within 8 weeks immediately before enrolment for this study; previous refractive surgery; rigid contact lens wear; OK; keratoconus or other corneal irregularity in either eye; aphakia; strabismus; central corneal scar or pupil/lid abnormality in either eye; contraindications to contact lens; surgically altered eyes; ocular infection of any type; ocular inflammation; anterior chamber angle grade 2 or narrower
Interventions	**Intervention 1:** soft contact lens, Test Lens 1 **Intervention 2:** soft contact lens, Test Lens 2 **Comparison intervention:** spectacle lenses
Outcomes	**Primary outcomes:** SER, AL **Secondary outcomes:** not reported **Maximum follow‐up:** 2 years
Starting date	April 2007 **Estimated end date:** February 2010
Contact information	clinicaltrials.gov/ct2/show/NCT00762970
Notes

**Table:** NCT01704729

Study name	The children's WEAR trial (phases 1 & 2)
Methods	Randomised parallel‐group design
Participants	**Inclusion criteria:** aged 12‐17 years; ≤ −1.00 D of myopic refractive error in each eye, with uncorrected vision ≤ 6/12 in at least 1 eye thought to be due to refractive error **Exclusion criteria:** significant strabismus or vision abnormality; vision deficiency
Interventions	**Intervention 1:** noncycloplegic self‐refraction and conventional glasses **Intervention 2:** cycloplegic subjective refraction by experienced optometrist and conventional glasses **Intervention 3:** cycloplegic subjective refraction by rural refractionist programme and conventional glasses **Comparison intervention:** cycloplegic subjective refraction by experienced optometrist and ready‐made glasses
Outcomes	**Primary outcome:** VA **Secondary outcomes:** visual functioning, frequency of glasses‐wear, accuracy of spectacles, value and satisfaction **Maximum follow‐up:** 2 months
Starting date	September 2012 **Estimated end date:** January 2013
Contact information	clinicaltrials.gov/ct2/show/NCT01704729
Notes

**Table:** NCT01729208

Study name	An evaluation of the effectiveness of dual focus soft contact lenses in slowing myopia progression
Methods	Randomised parallel‐group design
Participants	**Inclusion criteria:** 300 children aged 8‐12 years; BCVA by manifest refraction of +0.10 logMAR; SER error between 0.75 and −4.00 D inclusive; astigmatism < −0.75 D; anisometropia < 1.00 D; possess wearable and visually functional eyeglasses; agree to wear assigned contact lenses for a minimum of 10 h/day at least 6 days/week, for the duration of the 3‐year study **Exclusion criteria:** previously wore or currently wears contact lenses or RGP contact lenses, including OK lenses; currently or within 30 days before this study has been an active participant in another clinical study; current or prior use of BFs, PALs, atropine, pirenzepine, or any other myopia control treatment; regular use of ocular medications, artificial tears, or wetting agents; current use of systemic medications that may significantly affect contact lens wear, tear film production, pupil size, accommodation, or refractive state; allergy to fluorescein, benoxinate, proparacaine, or tropicamide; strabismus; any ocular, systemic, or neurodevelopmental condition that could influence refractive development
Interventions	**Intervention:** dual focus soft contact lens **Comparison Intervention:** SVSCL
Outcomes	**Primary outcomes:** change in refractive error relative to baseline, change in AL relative to baseline **Secondary outcomes:** incidence of AEs **Maximum follow‐up:** 3 years
Starting date	November 2012 **Estimated end date:** May 2019
Contact information	clinicaltrials.gov/ct2/show/NCT01729208
Notes

**Table:** NCT01787760

Study name	Controlling myopia progression with soft contact lenses
Methods	Randomised parallel‐group design
Participants	**Inclusion criteria:** myopic children aged 8‐12 years; best sphere contact lens correction must lie between −0.75 D (best of the 2 eyes) and −5.00 D (worst of the 2 eyes); astigmatism must be ≤ 1.00 D; 1.00 D or less difference in spherical equivalent between the 2 eyes; BCVA of 0.8 + 2 (20/25 + 2); SER VA of 0.820/25 or better in both eyes; at least 8 D of accommodation **Exclusion criteria:** any ocular or systemic allergy or disease that may interfere with contact lens wear; systemic disease or autoimmune disease or use of medication (e.g. antihistamine) that may interfere with lens wear; clinically significant (grade 3 or 4) abnormality of the cornea, which may contraindicate contact lens wear; clinically significant (grade 3 or 4) tarsal abnormalities or bulbar injection; any ocular infection; any corneal distortion; any infectious disease (e.g. hepatitis, tuberculosis) or immunosuppressive disease (e.g. HIV); diabetes; anisometropia > 1.00 D; astigmatism > 1.00 D in either eye; eye injury or surgery within 8 weeks immediately before enrolment for this study; previous refractive surgery; rigid contact lens wear; OK; keratoconus or other corneal irregularity in either eye; aphakia; strabismus; central corneal scar or pupil/lid abnormality in either eye; contraindications to contact lens; surgically altered eyes; ocular infection of any type; ocular inflammation; anterior chamber angle grade 2 or narrower
Interventions	**Intervention 1:** soft contact lens, Test Lens B **Intervention 2:** soft contact lens, Test Lens C **Comparison intervention:** spectacle lenses
Outcomes	**Primary outcomes:** SER, AL **Secondary outcomes:** not reported **Maximum follow‐up:** 3 years
Starting date	April 2007 **Estimated end date:** April 2010
Contact information	clinicaltrials.gov/ct2/show/NCT01787760
Notes

**Table:** NCT01829191

Study name	Controlling myopia progression with soft contact lenses (contact lens control)
Methods	Randomised parallel‐group design
Participants	**Inclusion criteria:** myopic children aged 8‐12 years; best sphere contact lens correction must lie between −0.75 D (best of the 2 eyes) and −5.00 D (worst of the 2 eyes); astigmatism ≤ 1.00 D; 1.00 D or less difference in spherical equivalent between the 2 eyes; BCVA of 0.8 + 2 (20/25 + 2); SER VA of 0.820/25 or better in both eyes; at least 8 D of accommodation **Exclusion criteria:** any ocular or systemic allergy or disease that may interfere with contact lens wear; systemic disease or autoimmune disease or use of medication (e.g. antihistamine) that may interfere with lens wear; clinically significant (grade 3 or 4) abnormality of the cornea, which may contraindicate contact lens wear; clinically significant (grade 3 or 4) tarsal abnormalities or bulbar injection; any ocular infection; any corneal distortion; any infectious disease (e.g. hepatitis, tuberculosis) or immunosuppressive disease (e.g. HIV); diabetes; anisometropia > 1.00 D; astigmatism > 1.00 D in either eye; eye injury or surgery within 8 weeks immediately before enrolment for this study; previous refractive surgery; rigid contact lens wear; OK; keratoconus or other corneal irregularity in either eye; aphakia; strabismus; central corneal scar or pupil/lid abnormality in either eye; contraindications to contact lens; surgically altered eyes; ocular infection of any type; ocular inflammation; anterior chamber angle grade 2 or narrower
Interventions	**Intervention 1:** soft contact lens, Test Lens A **Intervention 2:** soft contact lens, Test Lens C **Comparison intervention:** spectacle lenses
Outcomes	**Primary outcome:** SER error **Secondary outcomes:** AL **Maximum follow‐up:** 2 years
Starting date	April 2008 **Estimated end date:** May 2010
Contact information	clinicaltrials.gov/ct2/show/NCT01829191
Notes

**Table:** NCT01923675

Study name	The role of cone opsin mutations & glasses that control axial elongation
Methods	Randomised parallel‐group design
Participants	**Inclusion criteria:** nearsightedness with refractive error of at least −0.5 D; myopia progression at least −0.50 D/year in previous year; astigmatism and anisometropia not more than 1.5 D; distance monocular acuity 6/6 or better; near monocular acuity of 0.4 M or better; stereoacuity not more than 40 seconds of arc at 40 cm; no contact lens use during the study; willingness to donate a blood sample or a buccal swab for genetic analysis; can be refracted to 20/20 or 20/15 **Exclusion criteria:** glaucoma; amblyopia; strabismus; ocular disease; developmental delay; history of wearing BF lenses; many types of eye surgery; colour vision deficiency
Interventions	**Intervention 1:** spectacles with red‐blocking tint **Intervention 2:** spectacles with holographic diffuser and colour neutral tint **Intervention 3:** spectacles with holographic diffuser and red‐blocking tint **Comparison intervention:** spectacles with colour neutral tint
Outcomes	**Primary outcome:** axial elongation **Secondary outcomes:** cycloplegic autorefraction **Maximum follow‐up:** 18 months
Starting date	September 2013 **Estimated end date:** November 2016
Contact information	clinicaltrials.gov/ct2/show/record/NCT01923675
Notes

**Table:** NCT02001415

Study name	Efficacy study of different lens treatments on Chinese adolescent myopia (DLTCAM)
Methods	Randomised parallel‐group design
Participants	**Inclusion criteria:** 120 adolescent myopia patients aged 10‐15; myopic refraction between −1.00 D and −4.50 D; astigmatism ≤ −1.50 D; normal break‐up time of tear film **Exclusion criteria:** existence of any ocular disease except ametropia, hyperopia, severe dry eye
Interventions	**Intervention 1:** MyoVision spectacles **Intervention 2:** OK lenses at night **Comparison intervention:** spectacles
Outcomes	**Primary outcome:** ocular AL **Secondary outcomes:** SER **Maximum follow‐up:** 12 months
Starting date	November 2013 **Estimated end date:** September 2016
Contact information	clinicaltrials.gov/ct2/show/NCT02001415
Notes

**Table:** NCT02130167

Study name	Low‐concentration atropine for myopia progression in schoolchildren
Methods	Randomised parallel‐group design
Participants	**Inclusion criteria:** 60 children aged 6‐12 years with myopia of at least 0.5 D and astigmatism of ≤ −1.50 D **Exclusion criteria:** children with strabismus, amblyopia, cataract, glaucoma, or any ocular disease; any ocular surgery; history of systemic disease
Interventions	**Intervention:** 0.01% atropine eye drops **Comparison intervention:** 0.05% atropine eye drops
Outcomes	**Primary outcome:** cycloplegic spherical refraction **Secondary outcomes:** axial change, pupil size, accommodation, questionnaire **Maximum follow‐up:** 1 year
Starting date	August 2012 **Estimated end date:** August 2017
Contact information	clinicaltrials.gov/ct2/show/NCT02130167
Notes

**Table:** NCT02186184

Study name	Effect of orthokeratology vs spectacles on myopia progression in Chinese children: a crossover trial
Methods	Randomised cross‐over design
Participants	**Inclusion criteria:** aged 7‐14 years; VA 20/20 or better in each eye; spherical error ranging from −0.5 D to −5.0 D and astigmatism < 1.5 D in each eye; anisometropia < 1.0 D between the 2 eyes; no strabismus, amblyopia, or any other ocular or systematic disease that may affect refractive development **Exclusion criteria:** currently using or history of using other interventions to control myopia progression (acupuncture, drugs, contact lenses, ear needles, and so on); inability to co‐operate with the ocular examination; questionnaire survey; OK wearing
Interventions	**Intervention:** OK **Comparison intervention:** spectacles
Outcomes	**Primary outcomes:** refraction, AL **Secondary outcomes:** tear film break‐up time, self‐evaluation of comfort, corneal endothelial cell density **Maximum follow‐up:** 2 years
Starting date	June 2014 **Estimated end date:** June 2017
Contact information	clinicaltrials.gov/ct2/show/record/NCT02186184
Notes

**Table:** NCT02206217

Study name	Myopia control with the multisegment lens
Methods	Randomised parallel‐group design
Participants	**Inclusion criteria:** estimated 183 children aged 8‐13 years with SER between −1.00 D and −5.00 D; anisometropia and astigmatism not greater than 1.50 D; BCVA logMAR of 0 or better using spectacles; parental understanding of random allocation **Exclusion criteria:** ocular or systemic condition affecting vision or refractive development; prior treatment with any intervention for control of myopia
Interventions	**Intervention:** multisegment spectacle lens **Comparison intervention:** SVLs
Outcomes	**Primary outcome:** cycloplegic refraction **Secondary outcome:** AL **Maximum follow‐up:** 2 years
Starting date	August 2014 **Estimated end date:** July 2017
Contact information	clinicaltrials.gov/ct2/show/NCT02206217
Notes

**Table:** NCT02544529

Study name	Echothiophate iodide for the prevention of progression of myopia
Methods	Randomised parallel‐group design
Participants	**Inclusion criteria:** between 8‐15 years of age; documentation of progression of myopia within the 12 months before enrolment **Exclusion criteria:** any history of retinopathy of prematurity, glaucoma, cataracts, corneal disease, uveitis, manifest strabismus, nystagmus, or ocular trauma; any history of unstable asthma, diabetes, or juvenile idiopathic arthritis; systemic muscarinic agents, steroids, or anticholinesterase agents; benzalkonium chloride preservative allergy; astigmatism > 0.75 D; anisometropia > 1.50 D; pregnancy or positive pregnancy test at the screening visit
Interventions	**Intervention:** echothiophate iodide 0.03% ophthalmic solution **Comparison intervention:** carboxymethylcellulose sodium (0.5%)
Outcomes	**Primary outcome:** cycloplegic refraction **Secondary outcomes:** AL, choroidal thickness **Maximum follow‐up:** 12 weeks
Starting date	June 2016 **Estimated end date:** June 2017
Contact information	clinicaltrials.gov/ct2/show/NCT02544529
Notes

**Table:** NCT02643342

Study name	A 2‐year longitudinal study on the structural and optical effects of orthokeratology treatment on eye
Methods	Randomised parallel‐group design
Participants	**Inclusion criteria:** 90 children aged 6‐10 years; myopia between 0.50 D and 4.00 D in both eyes; astigmatism < 1.50 D; ≤ 1.25 D for with‐the‐rule astigmatism (axes 180 ± 30); ≤ 0.50 D for astigmatism of other axes in both eyes; anisometropia ≤ 1.50 D; symmetrical corneal topography with corneal toricity < 2.00 D in both eyes; agree for randomisation **Exclusion criteria:** contraindications for OK wear (e.g. limbus‐to‐limbus corneal cylinder, dislocated corneal apex); any type of strabismus or amblyopia; myopic treatment (e.g. refractive surgery, progressive lens wear for myopic control) before and during the study period; rigid contact lenses (including OK lenses); systemic condition that might affect refractive development (e.g. Down's syndrome, Marfan’s syndrome); ocular condition that might affect the refractive error (e.g. cataract, ptosis); poor compliance with lens wear to follow‐up
Interventions	**Intervention 1:** OK with normal compression factor **Intervention 2:** OK with increased compression factor **Comparison intervention:** SVLs
Outcomes	**Primary outcome:** AL **Secondary outcomes:** ocular aberration, corneal biomechanics, accommodation lag, choroidal thickness **Maximum follow‐up:** 2 years
Starting date	June 2016 **Estimated end date:** December 2019
Contact information	clinicaltrials.gov/ct2/show/NCT02643342
Notes

**Table:** NCT02643758

Study name	Myopia control using soft bifocal lenses
Methods	Randomised parallel‐group design
Participants	**Inclusion criteria:** 97 children aged 6‐12 years with refractive sphere −0.75 D to −4.50 D; refractive cylinder not to exceed 1.00 D; spherical equivalent: −0.75 D to−5.00 D; distance BCVA (logMAR) 0.14 or better in each eye and 0.10 or better in both eyes; difference in refractive error (SERt) in the 2 eyes not to exceed 1.00 D **Exclusion criteria:** children with prior history of myopia control treatment; contraindication to contact lens wear; binocular anomalies (e.g. strabismus)
Interventions	**Intervention:** BF soft contact lenses **Comparison intervention:** SVLs
Outcomes	**Primary outcomes:** AL, cycloplegic refractive error **Secondary outcomes:** wavefront aberrations, accommodation responses **Maximum follow‐up:** 2 years
Starting date	January 2016 **Estimated end date:** September 2018
Contact information	clinicaltrials.gov/ct2/show/NCT02643758
Notes

**Table:** NCT02700139

Study name	Shamir aspheric ophthalmic lenses (MyLens) for myopic control clinical trial
Methods	Randomised parallel‐group design
Participants	**Inclusion criteria**: myopia between 0.75 ~ to 4.50 D and with‐the‐rule astigmatism not more than 1.50 D; difference between eyes, no more than 1.25 spherical equivalent; BCVA is equal to or better than 0.10 in logMAR scale (Snellen VA 6/7.5 or better); eyes straight at distance and near with best subjective correction; willing to be randomised and wear the study spectacles according to the instructions from practitioner; willing to come back for follow‐up; in the Optometry Clinic during the study period **Exclusion criteria**: abnormal ocular and general health; prior myopic treatment (e.g. refractive surgery and progressive lens wear for myopic control) before and during the study period; history of rigid contact lenses (including OK lenses) wearing; systemic condition which might affect refractive development (for example, Down's syndrome, Marfan's syndrome); ocular conditions which might affect the refractive error (for example, cataract, ptosis)
Interventions	**Intervention**: aspheric lens **Comparison intervention**: single vision spheric/toric lens
Outcomes	**Primary outcome**: AL **Secondary outcomes**: not reported **Maximum follow‐up**: 1 year
Starting date	January 2016
Contact information	clinicaltrials.gov/ct2/show/NCT02700139
Notes

**Table:** NCT02955927

Study name	Combined atropine with orthokeratology in childhood myopia control (AOK): a randomized controlled trial
Methods	Randomised parallel‐group design
Participants	**Inclusion criteria:** 60 children aged 6‐11 years; myopia between 1.00 and 4.00 D in both eyes; astigmatism ≤ 2.50 D; with‐the‐rule astigmatism (axes 180 ± 30) ≤ 2.50 D; astigmatism with other axes ≤ 0.50 D in both eyes; < 1.00 D difference in manifest spherical equivalent (SE); cycloplegic objective refraction between 1.00 and 4.00 D in sphere; astigmatism ≤ 2.50 D; < 1.00 D difference in manifest SE between the 2 eyes; BCVA logMAR 0.10 or better in both eyes; symmetrical corneal topography with corneal toricity < 2.00 D in either eye; normal ocular health other than myopia; agree to be randomised and to attend scheduled visits and aftercare **Exclusion criteria:** contraindications to atropine (known allergies or cardiovascular disease, epilepsy); contraindications to contact lens wear and OK strabismus or amblyopia; history of myopia control treatment; rigid contact lens (including OK) wear experience; systemic condition that might affect refractive development; ocular condition that might affect refractive, poor response to lens wear including poor lens handling, poor vision and/ocular response after lens modifications, and poor compliance with scheduled visits
Interventions	**Interventions:** OK and 0.01% atropine eye drops **Comparison intervention:** OK
Outcomes	**Primary outcomes:** changes in AL **Secondary outcomes:** none reported **Maximum follow‐up:** 24 months
Starting date	November 2016 **Estimated end date:** April 2020
Contact information	clinicaltrials.gov/ct2/show/NCT02955927
Notes

**Table:** NCT02980445

Study name	Time outdoors as an intervention for myopia in children
Methods	Cluster‐RCT
Participants	**Inclusion criteria:** at baseline be enroled in grade 1 and 2 of primary schools **Exclusion Criteria**: any systemic or ocular pathology that may affect the refractive error status of the eye; strabismus and amblyopia; intellectual disability; using any anti‐myopia treatments (OK, atropine, accommodation function training, acupuncture, auricular point sticking, PALs or other anti‐myopia contact lenses)
Interventions	**Interventions**: test group I (40 min additional outdoor time/day): test group II (80 min additional outdoor time/day) **Comparison intervention:** usual pattern of outdoor activity
Outcomes	**Primary outcomes**: SER **Secondary outcomes**: AL **Maximum follow up**: 12 months
Starting date	October 2016 **Estimated end date**:November 2018
Contact information	clinicaltrials.gov/ct2/show/record/NCT02980445
Notes	The purpose of this study is to determine whether improved outdoor time has an effect on the onset and progression of myopia in children

**Table:** NCT03242226

Study name	The effect of +3.00 ADD on myopia progression in Chinese children
Methods	Randomised parallel‐group design
Participants	**Inclusion criteria:** 440 children aged 8‐12 years; refractive error (cycloplegic autorefraction); spherical equivalent −1.00 to −6.00 D in both eyes; astigmatism ≤ 2.00 D in both eyes; spherical equivalent anisometropia ≤ 1.50 D; BCVA ≥ 6/9.5 **Exclusion criteria:** allergy to tropicamide or topical anaesthetic drugs; eye disease causing visual impairment including strabismus, amblyopia, ocular surface–related disease, cataract, trauma, ocular fundus disease, ocular surgery; previous wearing of RGPs, PALs, BF spectacles, peripheral defocus modifying contact lenses; receiving visual function training
Interventions	**Intervention:** SVLs (distant vision) and +3.00 ADD spectacles (near vision) **Comparison intervention:** SVLs
Outcomes	**Primary outcome:** SER **Secondary outcomes:** AL, corneal curvature, binocular vision **Maximum follow‐up:** 3 years
Starting date	October 2016 **Estimated end date:** December 2018
Contact information	clinicaltrials.gov/ct2/show/NCT03242226
Notes

**Table:** NCT03246464

Study name	Clinical study of nearsightedness treatment with orthokeratology lenses (CONTROL)
Methods	Randomised parallel‐group design
Participants	**Inclusion criteria:** 50 children aged 6‐12 years; myopia −0.5 to −4.75 D spherical in 1 or both eyes; regular astigmatism ≤ −2.5 D in 1 or both eyes; anisometropia < 1.5 D spherical equivalent; BCVA 0.1 logMAR or better in both eyes; acceptance of treatment randomisation **Exclusion criteria:** manifest or latent squint; contraindications to use of OK comprising keratoconus, allergic conjunctivitis, and keratoconjunctivitis sicca; previous eye surgery; chronic eye disease demanding daily use of eye drops; noncompliance with eye examinations (unstable fixation or intolerance to OK); 1 or both parents being ethnic Asian, African, Hispanic, or Spanish
Interventions	**Intervention:** OK lenses **Comparison intervention:** regular SVLs
Outcomes	**Primary outcome:** AL **Secondary outcomes:** QoL, safety **Maximum follow‐up:** 18 months
Starting date	March 2017 **Estimated end date:** October 2020
Contact information	ichgcp.net/clinical‐trials‐registry/NCT03246464
Notes

**Table:** NCT03329638

Study name	A study assessing the efficacy and safety of DE‐127 ophthalmic solution in subjects with mild or moderate myopia (APPLE)
Methods	Randomised parallel‐group design
Participants	**Inclusion criteria:** 100 children aged 6‐11 years; SER error −1.0 D to −6.0 D in both eyes; anisometropia of spherical equivalent ≤ 1.50 diopters in both eyes; distance vision correctable to logMAR 0.2 or better in both eyes; normal IOP not greater than 21 mmHg in both eyes; no allergy to atropine, cyclopentolate, proparacaine, and benzalkonium chloride **Exclusion criteria:** amblyopia or manifest strabismus including intermittent tropia; ocular disorder that potentially affects myopia or refractive power; previous or current use of contact lenses, BFl lenses, PALs, or other forms of treatment (including atropine and pirenzepine) for myopia; systemic disorder that potentially affects myopia or refractive power
Interventions	**Intervention 1:** DE‐127 ophthalmic solution low dose **Intervention 2:** DE‐127 ophthalmic solution medium dose **Intervention 3:** DE‐127 ophthalmic solution high dose **Comparison intervention:** placebo ophthalmic solution
Outcomes	**Primary outcome:** spherical equivalent **Secondary outcomes:** not reported **Maximum follow‐up:** 12 months
Starting date	October 2017 **Estimated end date:** December 2019
Contact information	clinicaltrials.gov/ct2/show/NCT03329638
Notes

**Table:** NCT03334253

Study name	Low‐dose atropine for treatment of myopia
Methods	Randomised parallel‐group design
Participants	**Inclusion criteria:** 186 children aged 5‐12 years; myopia −1.00 D to −6.00 D spherical equivalent in both eyes; astigmatism ≤ 1.50 D in both eyes; anisometropia < 1.00 D spherical equivalent; gestational age ≥ 32 weeks; birth weight > 1500 g; understanding of the protocol and willingness to accept randomisation to atropine or placebo by parents; willingness to participate in a 2‐ to 4‐week run‐in phase using daily artificial tear eye drops; ability to return in 2‐ 4 weeks for possible randomisation; accessible to phone; willingness to be contacted by Investigator's site staff **Exclusion criteria:** current or previous use of BFs, PALs, or MF contact lenses; current or previous use of OK, RGP, or other contact lenses to reduce myopia progression; known atropine allergy; abnormality of the cornea, lens, central retina, iris, or ciliary body; current or prior history of manifest strabismus, amblyopia, or nystagmus; prior eyelid, strabismus, intraocular, or refractive surgery; Down's syndrome or cerebral palsy; female patients who are pregnant, lactating, or intending to become pregnant within the next 30 months; negative urine pregnancy test (required for all female patients who have experienced menarche); current or previous myopia treatment with atropine, pirenzepine, or other antimuscarinic agent within 4 weeks of 13th birthday
Interventions	**Intervention:** 0.01% atropine eye drops **Comparison intervention:** placebo eye drops
Outcomes	**Primary outcome:** SER error **Secondary outcome:** spherical equivalent **Maximum follow‐up:** 30 months
Starting date	June 2018 **Estimated end date:** October 2022
Contact information	clinicaltrials.gov/ct2/show/NCT03334253
Notes

**Table:** NCT03350620

Study name	CHAMP: study of NVK‐002 in children with myopia
Methods	Randomised cross‐over design (within‐person study)
Participants	**Inclusion criteria:** 483 children aged 3‐17 years; myopia SER of at least −0.50 D and no greater than −6.00 D myopia in each eye **Exclusion criteria:** astigmatism > −1.50 D in either eye; current or history of amblyopia or strabismus; history of any disease or syndrome that predisposes the patient to severe myopia; history in either eye of abnormal ocular refractive anatomy; serious systemic illness that, in the investigator’s opinion, would render the patient ineligible; chronic use (> 3 days/week) of any topical ophthalmic medication (prescribed or over‐the‐counter) other than the assigned study medication
Interventions	**Intervention 1:** NVK‐002 concentration 1 **Intervention 2:** NVK‐002 concentration 2 **Comparison intervention:** vehicle (placebo)
Outcomes	**Primary outcome:** myopia progression **Secondary outcomes:** mean progression rates, proportion of participants who show < −0.75 D progression, median time to change in myopia < −0.75 D **Maximum follow‐up:** 36 months
Starting date	November 2017 **Estimated end date:** November 2022
Contact information	clinicaltrials.gov/ct2/show/NCT03350620
Notes

**Table:** NCT03374306

Study name	Topical application of low‐concentration (0.01%) atropine on the human eye with fast and slow myopia progression rate
Methods	Randomised parallel‐group design
Participants	**Inclusion criteria:** 80 children aged 7‐10 years; good general health; no family history of ocular disease; no current or history of epilepsy or asthma; myopia −0.50 to −1.00 D (inclusive, both eyes); astigmatism ≤ 0.50 D; no hyperopia, amblyopia, or strabismus; no reported ocular eye disease or disorder or drug allergy **Exclusion criteria:** not reported
Interventions	**Intervention:** atropine 0.01% **Comparison intervention:** artificial tears
Outcomes	**Primary outcomes:** refractive errors **Secondary outcome:** AL **Maximum follow‐up:** 24 months
Starting date	January 2018 **Estimated end date:** June 2020
Contact information	clinicaltrials.gov/ct2/show/NCT03374306
Notes

**Table:** NCT03402100

Study name	Eye drops study for myopia control in schoolchildren
Methods	Randomised parallel‐group design
Participants	**Inclusion criteria:** 150 children aged 6‐12 years with myopia diagnosed with SER at least −0.5 D; able to use eye drops **Exclusion criteria:** children with astigmatism ≥ −1.50 D; strabismus, amblyopia, cataract, glaucoma, any ocular disease, any ocular surgery; history of systemic disease; contact lens user; OK user
Interventions	**Intervention 1:** 0.01% atropine eye drops **Intervention 2:** 0.005% atropine eye drops **Intervention 3:** 0.25% ketorolac eye drops **Intervention 4:** 0.01% atropine plus 0.25% ketorolac eye drops **Intervention 5:** 0.005% atropine plus 0.25% ketorolac eye drops
Outcomes	**Primary outcome:** cycloplegic spherical refraction, AL **Secondary outcome:** IOP, accommodation (dioptre), pupil size, anterior chamber depth, posterior chamber depth **Maximum follow‐up:** 1 year
Starting date	October 2014 **Estimated end date:** December 2019
Contact information	clinicaltrials.gov/ct2/show/NCT03402100
Notes

**Table:** NCT03413085

Study name	To evaluate the efficacy and safety of multifocal soft contact lens in myopia control
Methods	Randomised parallel‐group design
Participants	**Inclusion criteria:** 59 schoolchildren aged 6‐15 years, with SER error between −1.00 D and −10.00 D; VA with contact lens of 20/25 or better in each eye; astigmatism ≤ 1.50 D; anisometropia ≤ 1.00 D **Exclusion criteria:** eye disease interfering with contact lens wearing, use of BFs, PALs, RGP contact lenses, OK lenses; myopia control treatment within 1 month before screening visit; systemic disease affecting vision or contact lens wearing; autoimmune disease, infectious disease, or immunosuppressive disease; surgically altered eyes; receiving medication for long‐term use that interferes with contact lens wearing, tear film production, pupil size, accommodation, or refractive state; nasal decongestants, antihistamines, prednisolone, or methylphenidate
Interventions	**Intervention:** MFSCLs **Comparison intervention:** SVSCLs
Outcomes	**Primary outcomes:** objective cycloplegic refractive error, AL **Secondary outcomes:** myopia progression, axial elongation, patient self‐assessment, average wearing hours across the study period, reasons and rates for discontinued wear during the study period **Maximum follow‐up:** 48 weeks
Starting date	May 2018 **Estimated end date:** March 2020
Contact information	clinicaltrials.gov/ct2/show/NCT03413085
Notes

**Table:** NCT03465748

Study name	Effectiveness of orthokeratology in myopia control
Methods	Randomised parallel‐group design
Participants	**Inclusion criteria:** myopia progression > −1.00 D in 1 year; prescription between −1.00 D and −6.00 D; BCVA 20/25 or better; at least 1 eye with refractive astigmatism < 1.50 D **Exclusion criteria:** contraindications for OK; refractive surgery; current RGP contact lens wearers
Interventions	**Intervention:** OK **Comparison intervention:** SVLs
Outcomes	**Primary outcomes:** VA, AL, myopia progression **Secondary outcomes:** not reported **Maximum follow‐up:** 2 years
Starting date	May 2017 **Estimated end date:** May 2019
Contact information	clinicaltrials.gov/ct2/show/record/NCT03465748
Notes

**Table:** NCT03508817

Study name	Atropine 0.01% eye drops in myopia study (AIMS)
Methods	Randomised parallel‐group design
Participants	**Inclusion criteria:** age 6‐15 years; myopia ≥ 2.00 D (cycloplegic refraction; spherical equivalent); no prior or current treatment for preventing myopia progression (BFs/PALs/OK) **Exclusion criteria:** BCVA < 0.5 (6/12); refractive myopia; astigmatism ≥ 1.5 D; amblyopia; ocular hypertension/glaucoma; prior intraocular surgery; allergy to atropine eye drops; systemic diseases associated with myopia such as Marfan syndrome, Stickler syndrome; history of cardiac or significant respiratory diseases; lack of consent for participating in the study
Interventions	**Intervention:** atropine sulphate 0.01% eye drops **Comparison intervention:** control
Outcomes	**Primary outcomes:** SER error **Secondary outcomes:** AL; AEs **Maximum follow‐up:** 2 years
Starting date	December 2018 **Estimated end date:** January 2022
Contact information	clinicaltrials.gov/ct2/show/NCT03508817
Notes

**Table:** NCT03519490

Study name	Can distance center and near center multifocal contact lenses control myopia progression in children?
Methods	Randomised parallel‐group design
Participants	**Inclusion criteria**: myopia: ≥ 0.5 D in least myopic meridian, < 12.0 D in most myopic meridian); anisometropia (interocular difference in refractive error) ≤ 2 D; astigmatism: ≤ 3 D; myopia progression ≥ 0.5 D in at least 1 eye based on available clinical records or based on habitual spectacle prescription; BCVA of 20/20 or better in each eye; capable of proper handling, insertion and removal of hybrid contact lenses **Exclusion criteria**: ocular health: any pathology that may alter eye growth (e.g. history of retinal detachment and treatment for the same), and/or may adversely impact contact lens wear (e.g. chronic, poorly controlled allergic conjunctivitis) will be grounds for exclusion; strabismus, amblyopia; systemic disease that may affect vision, vision development or contact lens wear; chronic use of medications that may affect immunity, such as oral or topical corticosteroids; rigid or hybrid contact lens wear within the preceding 3 months; prior ocular surgery, nursing or pregnant mothers; participants who cannot commit to the 24‐month study period or who have a high likelihood of leaving the area within the 24‐month study period
Interventions	**Intervention**: MFl hybrid contact lens **Comparison intervention**: single vision hybrid contact lens
Outcomes	**Primary outcome**: myopia progress rate, AL **Secondary outcomes**: subjective myopia progression rate, macular pigment optical density, tear film dynamics and meibomian gland health **Maximum follow‐up**: 24 months
Starting date	1 June 2018
Contact information	clinicaltrials.gov/ct2/show/NCT03519490
Notes

**Table:** NCT03538002

Study name	The effect of blue‐light filtering spectacle lenses on myopia progression in schoolchildren
Methods	Randomised parallel‐group design
Participants	**Inclusion criteria:** refraction: myopia of −1.00 D to −5.00D; astigmatism: ≤ −1.50 D; anisometropia: ≤ 1.00 D; monocular BCVA: 0.0 LogMAR or better after full correction; parents' understanding and acceptance of random allocation of grouping **Exclusion criteria:** any ocular and systemic abnormalities might affect visual functions or refractive development; prior treatment of myopic control, e.g. drugs, OK, PALs, BFs, drugs (e.g. atropine), etc
Interventions	**Intervention:** blue light‐filtering spectacle lenses **Comparison intervention:** conventional anti‐reflection coated spectacle lens
Outcomes	**Primary outcomes:** cycloplegic refraction **Secondary outcomes:** AL **Maximum follow‐up:** 2 years
Starting date	September 2018 **Estimated end date:** January 2021
Contact information	clinicaltrials.gov/ct2/show/NCT03538002
Notes

**Table:** NCT03552016

Study name	Evaluation of progression of myopia in children treated with vitamin B2 and outdoor sunlight exposure
Methods	Randomised parallel‐group design
Participants	**Inclusion criteria:** age 6‐12 years old with myopia > 0.50 D and astigmatism no more than 1.5 D; caretakers who choose to enrol their child in the study must agree to participate in the study on their own will after knowledge of potential alternatives (spectacle correction, OK, atropine eye drops, etc.) are explained to the patient's caretaker **Exclusion criteria:** known allergy to riboflavin; birth history of premature birth; developmental delay or other neurological or mental conditions; major systemic health problems; significant anisometropia > 1.5 D; any other eye condition that may complicate interpretation of data including: congenital glaucoma, congenital cataract, ectatic corneal condition, amblyopia or strabismus
Interventions	**Intervention 1:** 200 mg riboflavin (oral) **Intervention 2:** 400 mg riboflavin (oral) **Comparison intervention:** 0 mg riboflavin (oral)
Outcomes	**Primary outcomes:** cycloplegic refraction **Secondary outcomes:** AL, keratometry values, uncorrected best VA **Maximum follow‐up:** 3 years
Starting date	October 2018 **Estimated end date:** October 2021
Contact information	clinicaltrials.gov/ct2/show/NCT03552016
Notes

**Table:** NCT03623074

Study name	Control of myopia using novel spectacle lens designs (CYPRESS)
Methods	Randomised parallel‐group design
Participants	**Inclusion criteria:** age 6‐10 years (day prior to 10th birthday) at time of informed consent/assent; SER error between −0.75 and −4.50 D; spherical equivalent refraction power between the 2 eyes must be ≤ 1.50 D; willingness to participate in the trial for 3 years without content lens wear **Exclusion criteria:** previous or current use of contact lenses; previous or current use of BFs, PAL spectacles; previous or current use of myopia control treatment; astigmatism worse than −1.25 DC in either eye
Interventions	**Intervention:** novel spectacle lens design **Comparison intervention:** spectacle lenses
Outcomes	**Primary outcomes:** AL; SER **Secondary outcomes:** not reported **Maximum follow‐up:** 36 months
Starting date	July 2018 **Estimated end date:** January 2022
Contact information	clinicaltrials.gov/ct2/show/NCT03623074
Notes

**Table:** NCT03681366

Study name	Myopia control using optimized optical defocus: a randomized double masked control trial
Methods	Randomised parallel‐group design
Participants	**Inclusion criteria:** age at enrolment 8‐13 years; Hong Kong Chinese; SER −1.00 to −5.00D; astigmatism ≤ −1.00 D; anisometropia ≤ 1.25 D; spectacle corrected monocular VA 0.0 logMAR or better; contact lens corrected monocular VA 0.1 logMAR or better; normal binocular function; willingness to wear contact lenses regularly; parents' understanding and acceptance of random allocation of grouping and masking **Exclusion criteria:** prior myopia control treatment, e.g. OK, defocus soft contact lenses, PALs, BF lenses, drugs (e.g. atropine), etc.; strabismus or decompensated phoria (checked by cover test at far and near in screening); known contraindications for contact lens wear; have any ocular and systemic diseases and abnormalities that might affect visual function or refractive development
Interventions	**Intervention:** SVSCLs **Comparison intervention:** DISC3.5 Plus lens
Outcomes	**Primary outcomes:** spherical equivalent **Secondary outcomes:** AL **Maximum follow‐up:** 12 months
Starting date	October 2018 **Estimated end date:** April 2021
Contact information	clinicaltrials.gov/ct2/show/NCT03681366
Notes

**Table:** NCT03690089

Study name	Low‐dose atropine eye drops to reduce progression of myopia in children in the United Kingdom (CHAMP‐UK)
Methods	Randomised parallel‐group design
Participants	**Inclusion criteria:** age 6‐12 years (at the time of consenting); myopia of ≥ −0.5 D (SER error) in both eyes; distance BCVA 0.20 logMAR or better in both eyes **Exclusion criteria:** other ocular morbidities; myopia of ≥ −10 D in either eye; astigmatism of ≥ 2 D in either eye; amblyopia; significant health problems that can compromise the ability to attend research visits or complete the study
Interventions	**Intervention:** atropine sulphate 0.01% eye drops **Comparison intervention:** placebo eye drops
Outcomes	**Primary outcome:** SER error **Secondary outcomes:** AL, BCVA distance, near VA, reading speed, pupil diameter, accommodation, spectacle correction, eye drop tolerability, AEs, QoL **Maximum follow‐up:** 24 months
Starting date	April 2019 **Estimated end date:** December 2024
Contact information	clinicaltrials.gov/ct2/show/record/NCT03690089
Notes

**Table:** NCT03690414

Study name	Evaluation of short‐term use of experimental eye drops BHVI2, 0.02% atropine, and BHVI2 plus 0.02% atropine eye drops
Methods	Randomised parallel‐group design
Participants	**Inclusion criteria:** age 6‐13 years; myopic; normal ocular findings; spherical equivalent between −0.50 D and −6.00 D; vision correctable to at least 20/25 or better in each eye with spectacles **Exclusion criteria:** pre‐existing ocular irritation, systematic disease, eye trauma, myopia control interventions
Interventions	**Intervention 1:** experimental BHVI2 **Intervention 2:** atropine sulphate 0.02% eye drops **Comparison intervention:** combination eye drops
Outcomes	**Primary outcomes:** pupillary diameter, accommodative amplitude **Secondary outcomes:** not reported **Maximum follow‐up:** 1 month
Starting date	October 2018 **Estimated end date:** February 2019
Contact information	clinicaltrials.gov/ct2/show/NCT03690414
Notes

**Table:** NCT03865160

Study name	Low‐dose atropIne for myopia control in children (AIM)
Methods	Randomised parallel‐group design
Participants	**Inclusion criteria:** aged 6‐11 years with −1.00 to −10.00 D myopia **Exclusion criteria:** any organic eye disease, strabismus, astigmatism and/or anisometropia > 1.5 D, prematurity, use of mono‐/multifocal contact lenses, pre‐treatment with atropine
Interventions	**Intervention:** 0.01% atropine eye drops **Comparison intervention:** placebo
Outcomes	**Primary outcome:** change of cycloplegic refraction/year [D/year] **Secondary outcome:** change in axial eye length/year [mm/year] **Maximum follow up:** 36 months
Starting date	June 2021 **Estimated end date:** April 2025
Contact information	clinicaltrials.gov/ct2/show/NCT03865160
Notes

**Table:** NCT03881358

Study name	Orthokeratology for high myopia (OHM) study
Methods	Randomised parallel‐group design
Participants	**Inclusion criteria:** myopia: at least 5.00 D in 1 eye or in both eyes; astigmatism: ≤ 1.50 D; with‐the‐rule astigmatism (axes 180 ± 30) ≤ 1.25 D, astigmatism of other axes ≤ 0.50 D in both eyes, anisometropia not be more than 1.00 D in the former and not more than 2.00 D in the latter, Monocular Snellen BCVA 6/7.5 or better **Exclusion criteria:** strabismus at distance or near, previous experience in contact lens wear or myopia control treatment (e.g. refractive therapy or progressive spectacles), contraindication for contact lens wear and OK (e.g. limbus to limbus corneal cylinder and dislocated corneal apex), previous history of ocular surgery, trauma, or chronic ocular disease, concurrent use of medications that may affect tear quality or contact lens wear, systemic or ocular conditions that may affect tear quality or contact lens wear (e.g allergy and concurrent medication) or that may affect refractive development (e.g Down syndrome, ptosis)
Interventions	**Intervention:** OK lenses (target for 4.00 D) and thinner spectacles during daytime **Comparison intervention:** OK lenses for high myopia (target for full correction)
Outcomes	**Primary outcome:** change in AL elongation over 24 months **Secondary outcome:** first fit success rate of a newly designed OK lens for high‐myopic children, QoL (PREP: Pediatric Refractive Error Profile) **Maximum follow up:** 24 months
Starting date	August 2018 **Estimated end date:** January 2024
Contact information	clinicaltrials.gov/ct2/show/NCT03881358
Notes

**Table:** NCT03911271

Study name	Low‐dose atropine for the prevention of myopia progression in Danish children (APP)
Methods	Randomised parallel‐group design
Participants	**Inclusion criteria:** aged ≥ 6 to ≤ 9 years, myopia ≤ −1 (spherical power) in at least 1 eye, aged ≥ 9 to ≤ 12 years, myopia ≤ −2 (spherical power) in at least 1 eye, cylinder < 1.5 D **Exclusion criteria:** myopia related to retinal dystrophies, collagen syndromes (Ehlers‐Danlos syndrome, Marfan syndrome and Stickler syndrome), other ocular pathology (e.g. amblyopia, strabismus), previous eye surgery, previous use of agents thought to affect myopia progression, e.g. atropine, pirenzepine or 7‐mx (metabolite of caffeine and theobromine) and OK contact lenses, known allergy to atropine or any of the contents of the trial medication (active and inactive ingredients) used in the study, serious systemic health troubles (e.g. cardiac or respiratory illness) and developmental disorders and delays
Interventions	**Intervention:** in phase 1 (treatment phase), 0.1% atropine loading dose for 6 months followed by 0.01% atropine for 18 months. In phase 2 (washout phase) treatment will be stopped, and the participants monitored for 12 months **Intervention:** in phase 1 (treatment phase), 0.01 % atropine for 24 months. In phase 2 (washout phase), treatment will be stopped, and the participants monitored for 12 months **Comparison intervention:** in phase 1 (treatment phase) placebo eye drops for 24 months. In phase 2 (washout phase), treatment will be stopped, and the participants monitored for 12 months
Outcomes	**Primary outcome:** change in AL, change in spherical equivalent **Secondary outcome:** AEs and adverse reactions, change in choroidal thickness, change in ocular biometry (i.e. keratometry, anterior chamber depth, lens thickness, vitreous axial distance) from baseline, change in higher‐order aberrations. **Maximum follow up:** 36 months
Starting date	May 2019 **Estimated end date:** April 2024
Contact information	clinicaltrials.gov/ct2/show/NCT03911271
Notes

**Table:** NCT03918915

Study name	The safety and efficacy of SYD‐101 in children with myopia (STAR)
Methods	Randomised parallel‐group design
Participants	**Inclusion criteria:** age 2‐14 years, myopia of 0.5 D‐6.00 D (inclusive) in both eyes, astigmatism ≤ 1.50 D in both eyes, anisometropia ≤ 1.00 D in both eyes, BCVA, Snellen equivalent of 20/32 or better **Exclusion criteria:** history or current evidence of a medical condition predisposing patients to degenerative myopia (e.g. Marfan syndrome, Stickler syndrome), or a condition that may affect visual function or development (e.g. diabetes mellitus, chromosome anomaly), current use of a monoamine oxidase inhibitor, evidence of any ocular inflammation or infection in either eye, including blepharitis, conjunctivitis, keratitis, and scleritis, past, present or future plans to use OK, RGP, BF, PALs, MF, or other lenses to reduce myopia progression; or the use of atropine, pirenzepine or other anti‐muscarinic agent for myopia, history or evidence of ocular surgery or planned future ocular surgery in either eye
Interventions	**Intervention:** Part 1 (treatment phase) 0.01% atropine eye drops. Part 2 (withdrawal phase), 0.01% atropine eye drops. **Intervention:** Part 1 (treatment phase) 0.01% atropine eye drops. Part 2 (withdrawal phase), placebo. **Intervention:** Part 1 (treatment phase) 0.01% atropine eye drops. Part 2 (withdrawal phase), 0.01% atropine eye drops. **Intervention:** Part 1 (treatment phase) 0.01% atropine eye drops. Part 2 (withdrawal phase), placebo **Comparison intervention:** Part 1 (treatment phase), placebo. Part 2 (withdrawal phase), 0.01% atropine eye drops
Outcomes	**Primary outcome:** proportion of participants with confirmed myopic progression > 0.75 D, based on SER **Secondary outcome:** time to progression of myopia > 0.75 D, progression of myopia measured as SER, mean change in AL from baseline **Maximum follow up:** 36 months
Starting date	April 2019 **Estimated end date:** June 2025
Contact information	clinicaltrials.gov/ct2/show/NCT03918915
Notes

**Table:** NCT03942419

Study name	Microdosed atropine 0.1% and 0.01% ophthalmic solutions for reduction of pediatric myopia progression
Methods	Randomised parallel‐group design
Participants	**Inclusion criteria:** age 3‐12 years, myopia −1.00 D to −6.00 D in both eyes, astigmatism ≤ 1.50 D in both eyes, anisometropia < 1.50 D, BCVA in current correction of 0.2 logMAR or better with interocular difference ≤ 0.1 logMAR **Exclusion criteria:** current or previous myopia treatment with non‐study atropine, pirenzepine or other topical anti‐muscarinic agent, current use of BFs, PALs, or MFSCLs, use of RGPs, including OK lenses within 90 days of screening, known atropine allergy, abnormality of the cornea, lens, central retina, iris or ciliary body. Current or prior history of manifest strabismus, amblyopia, or nystagmus, prior eyelid, strabismus, intraocular, or refractive surgery,IOP > 26 mmHg, history of premature birth, medical conditions predisposing patient to degenerative myopia, abnormal ocular refractive anatomy, and/or any history of intraocular surgery
Interventions	**Intervention:** 0.1% atropine eyedrops **Intervention:** 0.01% atropine eyedrops **Comparison intervention:** placebo
Outcomes	**Primary outcome:** proportion of primary study eyes showing < 0.50 D (spherical equivalent) myopia progression compared to baseline measured using cycloplegic autorefraction **Maximum follow up:** 36 months
Starting date	June 2019 **Estimated end date:** June 2025
Contact information	clinicaltrials.gov/ct2/show/NCT03942419
Notes

**Table:** NCT03949101

Study name	Atropine for children and adolescent myopia progression study
Methods	Randomised parallel‐group design
Participants	**Inclusion criteria:** aged 7‐12 years, spherical equivalent myopia range −0.5D to −6.0 D, without other eye diseases except for ametropia **Exclusion criteria:** other eye diseases: amblyopia, strabismus, eye trauma, etc, cycloplegia contradictions, atropine use, severely allergic to atropine, using other eye drops for treatment, severe heart, lung, liver and kidney diseases
Interventions	**Intervention:** combined use of 1% atropine ointment and 0.01% atropine eye drops **Intervention:** 0.01% atropine eye drops
Outcomes	**Primary outcome:** spherical equivalent progression, AL change **Secondary outcome:** choroidal thickness change, choroidal blood flow density, anterior chamber depth, IOP **Maximum follow up:** 24 months
Starting date	May 2019 **Estimated end date:** September 2021
Contact information	clinicaltrials.gov/ct2/show/NCT03949101
Notes

**Table:** NCT04048148

Study name	Myopia progression trial with novel myopia control design spectacle lenses
Methods	Randomised cross‐over design (within‐person study)
Participants	**Inclusion criteria:** aged 8‐13 years, spherical refractive error of −0.75 to −4.75 D, astigmatism of not more than 1.50 D, anisometropia of not more than 1.00 D, BCVA of equal or better than 0.05 LogMAR, no strabismus by cover test at near and distance, absence of ocular disease such as retinal disease, cataract and ptosis. Good general health, without systemic or neurodevelopmental conditions, without ocular or systemic medicine, which might affect myopia progression or VA through known effects on retina, accommodation or significant elevation of IOP, no history of PALs or BF use and no prior use of contact lenses **Exclusion criteria:** vulnerability of the patient, participation in another study that might have an influence on vision or interfere with study assessments
Interventions	**Intervention:** novel myopia control spectacle lenses. This group will be randomised to wear test lenses for 6 months followed by control lenses for 6 months and then test lenses for another 6 months. **Intervention:** SVLs. This group will be randomised to wear control lenses for 6 months followed by test lenses for 12 months.
Outcomes	**Primary outcome:** change in myopia progression measured by cycloplegic refraction **Secondary outcome:** change in ocular AL **Maximum follow up:** 12 months
Starting date	May 2019 **Estimated end date:** May 2021
Contact information	clinicaltrials.gov/ct2/show/NCT04048148
Notes

**Table:** NCT04173780

Study name	Topical 0.01% atropine for the control of fast progressing myopia (Myopie‐STOP)
Methods	Randomised parallel‐group design
Participants	**Inclusion criteria:** age 4‐12 years, spherical equivalent myopia from −1.00 to −6.00D, fast progressing myopia (> 0.75 D/year) **Exclusion criteria:** astigmatism > 1.50 D, anisometropia > 2.00 D, concomitant pathology of anterior or posterior segments, other ocular diseases (ocular inflammation, strabismus), atropine hypersensitivity or allergy
Interventions	**Intervention:** 0.01% atropine eyedrops **Comparator intervention:** placebo
Outcomes	**Primary outcome:** myopia in spherical dioptres **Secondary outcome:** AL, AEs, QoL questionnaire **Maximum follow up:** 12 months
Starting date	February 2020 **Estimated end date:** February 2023
Contact information	clinicaltrials.gov/ct2/show/NCT04173780
Notes

**Table:** NCT04293328

Study name	Monthly replacement orthokeratology for myopia control in young children (MR2)
Methods	Randomised parallel‐group design
Participants	**Inclusion criteria:** aged 6‐10 years, refractive sphere between −0.75 to −4.00 D, refractive cylinder ≤ −1.50 D and anisometropia ≤ −1.00 D, BCVA better than 0.08 logMAR in the worse eye, normal binocular function and accommodative status, no prior experience in contact lens wear and myopia control treatment, normal ocular and general condition and not on medication that may contraindicate OK lens wear **Exclusion criteria:** strabismus at distance or near, contraindication for OK lens wear, prior history of ocular surgery, trauma, or chronic ocular disease, systemic or ocular conditions that may interfere with refractive development, systemic or ocular conditions that may interfere with tear quality and contact lens wear
Interventions	**Intervention:** OK lenses with weekly protein removal **Intervention:** OK lenses without weekly protein removal
Outcomes	**Primary outcome:** axial elongation, changes in back surface lens deposits **Secondary outcome:** number of participants with serious AEs, serious AEs of the cornea, the palpebral, bulbar and tarsal conjunctiva **Maximum follow up:** 12 months
Starting date	July 2020 **Estimated end date:** March 2022
Contact information	clinicaltrials.gov/ct2/show/NCT04293328
Notes

**Table:** NCT04295707

Study name	Monthly replacement orthokeratology for myopia control in existing lens wearers (MR1)
Methods	Randomised parallel‐group design
Participants	**Inclusion criteria:** aged 6‐15 years, normal ocular and general condition and not on medication that may contraindicate OK lens wear, refractive sphere between −0.75 to −4.00 D, refractive cylinder ≤ −1.50 D and anisometropia ≤ −1.00D, best correctable vision better than 0.08 logMAR in the worse eye, normal binocular function and accommodative status **Exclusion criteria:** strabismus at distance or near, contraindication for OK lens wear, prior history of ocular surgery, trauma, or chronic ocular disease, systemic or ocular conditions that may interfere with refractive development, systemic or ocular conditions that may interfere with tear quality and contact lens wear
Interventions	**Intervention:** monthly replacement OK lenses with weekly protein removal **Intervention:** monthly replacement OK lenses without weekly protein removal **Intervention:** yearly replacement OK lenses with weekly protein removal
Outcomes	**Primary outcome:** axial elongation, back surface lens deposits **Secondary outcome:** number of participants with serious AEs **Maximum follow up:**
Starting date	March 2020 **Estimated end date:** December 2022
Contact information	clinicaltrials.gov/ct2/show/NCT04295707
Notes

**Table:** NCT04618510

Study name	SEED‐LVPEI myopia study (SLIMS)
Methods	Randomised parallel‐group design
Participants	**Inclusion criteria:** age 7‐15 years, myopia (SE) between −0.50 D to −10.00 D, astigmatism < 0.75 D, anisometropia < 1.00 D, neophyte or existing soft contact lens wearer, BCVA ≤ 20/20 **Exclusion criteria:** participants who had any ocular or systemic conditions that could influence the refractive error, poor compliance of contact lenses from existing wearer, prior use of OK lenses/BF lenses/anti‐myopia strategies, participants who had any medications that could influence the refractive error
Interventions	**Intervention:** extended depth of focus contact lenses **Comparator intervention:** SVLs
Outcomes	**Primary outcome:** changes in SER error from baseline, change in SER error among different degrees of myopia from baseline, AL changes in the intervention and control group from baseline, changes in AL among different degrees of myopia from baseline, peripheral refractive error changes of the individuals in the intervention and control group from baseline, changes in peripheral SER error among different degrees of myopia from baseline **Secondary outcome:** qualitative assessment of discomfort and visual experience of centre‐distance MF contact lens will be measured on a scale of 0‐4 (0 = Never, 1 = Rarely, 2 = Sometimes, 3 = Often, 4 = Always) **Maximum follow up:** 12 months
Starting date	December 2020 **Estimated end date:** August 2022
Contact information	clinicaltrials.gov/ct2/show/NCT04618510
Notes

**Table:** NCT04699357

Study name	The effect and safety of different doses of atropine on myopic progression of highly myopic children: multi‐centered randomized clinical trial
Methods	Randomised parallel‐group design
Participants	**Inclusion criteria:** age 6‐12 years, BCVA of distant vision is at least 0.5, near vision is at least 1.0, Titmus stereo vision is < 80 seconds, far exotropia is < 10 prism degrees, far esotropia is < 6‐8 prism degrees, and astigmatism ≤ −2.50 D; myopia progressed > 0.5D in the past year **Exclusion criteria:** diseases of the study eye: keratitis, keratoconus, congenital cataract, glaucoma, fundus diseases; present situation with anterior segment or posterior segment inflammation, such as acute conjunctivitis, iridocyclitis, systemic diseases affecting drug use: albinism, epilepsy, serious mental and neurological diseases, congenital heart disease, arrhythmia, atropine allergy, very low‐birthweight infants with birthweight < 1500 g, receiving other treatment to control the development of myopia, including anticholinergic drugs such as atropine, or participated in other functional frame lens, MFl soft lens in the past year
Interventions	**Intervention:** 0.01% atropine eye drops **Intervention:** 0.04% atropine eye drops **Intervention:** 0.1% atropine eye drops
Outcomes	**Primary outcome:** changes of spherical equivalent, changes of AL **Maximum follow up:** not reported
Starting date	July 2021 **Estimated end date:** August 2025
Contact information	clinicaltrials.gov/ct2/show/NCT04699357
Notes

**Table:** NCT04770610

Study name	Study of OT‐101 in treating myopia
Methods	Randomised parallel‐group design
Participants	**Inclusion criteria:** aged 3‐15 years, refractive error ≥ −1.00 D of spherical equivalent, astigmatism ≥ 1.50 D cylinder, progression of at least −0.50 D of spherical equivalent in the last 12 months **Exclusion criteria:** active or a history of chronic or recurrent episodes of ocular inflammation (e.g. moderate to severe blepharitis, allergic conjunctivitis, peripheral ulcerative keratitis, scleritis) in either eye, have undergone any myopia control treatment including OK, RGP contact lenses, BF contact lenses, PAL spectcles, or other lenses to reduce myopia progression in the previous 6 months, myopic correction in the form of SVLs and/or SVSCLs are allowed, have undergone any form of refractive eye surgery including incisional keratotomy, photorefractive keratectomy, laser in situ keratomileusis, laser‐assisted sub‐epithelial keratectomy), corneal inlay procedures, conductive keratoplasty, small incision lenticule extraction (SMILE), cataract extraction, or any form of intraocular lens implantation
Interventions	**Intervention:** 0.01% atropine eye drops for 4 years **Intervention:** 0.01% atropine eye drops for 3 years and placebo for 1 year **Comparator intervention:** placebo for 4 years
Outcomes	**Primary outcome:** percentage of study eyes with a −0.75 D of progressive myopia defined as an increase in spherical equivalent of ≥ −0.75 D **Secondary outcome:** change in spherical equivalent (D) in the study eye, change in study eye AL **Maximum follow up:** 36 months
Starting date	April 2021 **Estimated end date:** May 2026
Contact information	clinicaltrials.gov/ct2/show/NCT04770610
Notes

**Table:** NCT04813640

Study name	Eye length signal with myopia control
Methods	Randomised parallel‐group design
Participants	**Inclusion criteria:** aged 7‐14 years,spherical component −0.50 D to −4.50 D, astigmatism ≤ −1.50 D, ocular health findings considered to be "normal". Correctable vision to at least 6/9.5 (20/30) or better in each eye with spectacles **Exclusion criteria:** known allergy to, or a history of intolerance to tropicamide or topical anaesthetics, strabismus and/or amblyopia, previous eye surgery (including strabismus surgery), any ocular, systemic or other condition or disease with possible associations with myopia or affecting refractive development e.g. Marfan syndrome, retinopathy of prematurity, diabetes, any ocular injury or condition (including keratoconus and herpes keratitis) of the cornea, conjunctiva or eyelids, worn BFs or PALs, worn OK or BF contact lenses, current orthoptic treatment or vision training
Interventions	**Intervention:** novel myopia control spectacles (Prototype 1) **Intervention:** novel myopia control spectacles (Prototype 2) **Intervention:** commercially available myopia control spectacles **Comparator intervention:** SVLs
Outcomes	**Primary outcome:** change in AL **Secondary outcome:** vision and choroidal physiology **Maximum follow up:** 6 months
Starting date	February 2021 **Estimated end date:** October 2021
Contact information	clinicaltrials.gov/ct2/show/NCT04813640
Notes

**Table:** NCT04854447

Study name	Part‐time versus full‐time spectacles for myopia control (ParMA Study)
Methods	Randomised parallel‐group design
Participants	**Inclusion criteria:** age 4‐16 years old, SER between −0.50D and −6.00D, astigmatism ≤ 1.50 D, in each eye, anisometropia ≤ 1.50 D between the 2 eyes, BCVA LogMAR 0.1 or better, absence of any ocular or systemic condition that could influence refractive development, other than myopia **Exclusion criteria:** presence of strabismus, amblyopia, prematurity (gestational age < 37 weeks), ocular condition affecting refraction (i.e. cataract, dislocated lens), systemic condition affecting refraction (i.e. Down syndrome, Marfan syndrome), allergy to cyclopentolate, severe ocular or systemic allergies
Interventions	**Intervention:** part‐time myopia correction with SVLs **Intervention:** full‐time myopia correction with SVLs
Outcomes	**Primary outcome:** change in SER, change in AL **Secondary outcome:** change in choroidal thickness, subjective tolerance using a standardised questionnaire **Maximum follow up:** 12 months
Starting date	February 2021 **Estimated end date:** October 2021
Contact information	clinicaltrials.gov/ct2/show/NCT04854447
Notes

**Table:** NCT05062031

Study name	Myopia control in children: comparison of Defocus Incorporated Multiple Segments® lenses versus atropine 0.05% eyedrops (ATROSMART)
Methods	Randomised parallel‐group design
Participants	**Inclusion criteria:** aged 4‐14 years, sphere power between −1.00 and −6.00 D in at least 1 of the 2 eyes, cylindrical power < 2 D, maximum refractive error strictly inferior to 8 D in the flattest axis, no previous myopia control strategy (OK, soft defocusing lenses, low‐concentration atropine eye drops, peripheral defocusing corrective lenses) **Exclusion criteria:** history of genetic disease, or general condition suggesting a syndromic myopia (including AL > 27 mm), strabismus, amblyopia defined by BCVA strictly inferior to 10/10 on 1 of the 2 eyes, anisometropia defined by a difference of ≥ 2 D between the 2 eyes (in spherical equivalent), history of allergy to atropine, history of severe anaphylaxis, optical correction with contact lenses, previous ophthalmologic surgery of the cornea, lens, retina, history of glaucoma or any other chronic ophthalmological disease in the course of treatment (including vernal keratoconjunctivitis)
Interventions	**Intervention:** Defocus Incorporated Multiple Segments(DIMS) spectacle lenses **Intervention:** 0.05% atropine plus single vision contact lenses
Outcomes	**Primary outcome:** difference in AL and SER **Maximum follow up:** 24 months
Starting date	October 2021 **Estimated end date:** October 2024
Contact information	clinicaltrials.gov/ct2/show/NCT05062031
Notes

**Table:** NCT05134935

Study name	Defocus (DIMS) spectacles versus ortho‐K lenses (OKL) for slowing myopia progression in Danish children aged 6‐12 years. (NISDO)
Methods	Randomised parallel‐group design
Participants	**Inclusion criteria:** aged 6‐12 years. Myopia of the 6‐8‐year‐olds −1.00 to −4.75 D spherical component and up to −2.50 D of regular astigmatism. Myopia of the 9‐12‐year‐olds −2.00 to −4.75 D spherical component and up to −2.50 D of regular astigmatism. Anisometropia < 1.5 D cycloplegic SER error. BCVA age 6‐8 (inclusive) years 0.8 Snellen (equivalent to ≥ 3/5 letters on the 0.8 line = 78 ETDRS letters); age 9‐12 years: 1.0 Snellen (equivalent to ≥ 3/5 letters on the 1.0 line = 83 ETDRS letters) **Exclusion criteria:** manifest or latent squint, contraindications to the use of OK lenses comprising keratoconus, chronic allergic conjunctivitis and keratoconjunctivitis sicca, previous eye surgery, chronic eye disease demanding daily use of eye drops
Interventions	**Intervention:** OK lenses **Intervention:** DIMS spectacle lenses
Outcomes	**Primary outcome:** AL growth of the eye **Secondary outcome:** overall eye length growth, defined as the sum of AL and choroidal thickness, pupil size, choroidal thickness, vision‐related QoL using a standardised questionnaire (PREP2) **Maximum follow up:** 18 months
Starting date	June 2022 **Estimated end date:** May 2025
Contact information	clinicaltrials.gov/ct2/show/NCT05134935
Notes

**Table:** NCT05159765

Study name	Progressive myopia treatment evaluation for NaturalVue multifocal contact lens trial (PROTECT)
Methods	Randomised parallel‐group design
Participants	**Inclusion criteria:** aged 7 to < 13 (inclusive), SER error between −0.75 and −5.00 D, astigmatism ≤ −0.75 D, anisometropia < 1.00 D **Exclusion criteria:** previously worn or currently wears rigid or gas‐permeable contact lenses, including OK lenses, appears to exhibit poor personal hygiene, that, in the investigator's opinion, might prevent safe contact lens wear, current or prior use of BFs, PALs, atropine, pirenzepine, MF or specialised contact lenses, or any other myopia control treatment
Interventions	**Intervention:** MF contact lenses **Comparator intervention:** single vision contact lenses
Outcomes	**Primary outcome:** change in refractive error from baseline **Secondary outcome:** change in AL from baseline **Maximum follow up:** 36 months
Starting date	December 2021 **Estimated end date:** August 2025
Contact information	clinicaltrials.gov/ct2/show/NCT05159765
Notes

**Table:** NCT05192824

Study name	Effects of different designs of orthokeratology lens on myopia control and visual quality
Methods	Randomised parallel‐group design
Participants	**Inclusion criteria:** age 8‐13 years, myopia between −1.00 D and 4.00 D in both eyes, astigmatism < 1.5 D for with the rule astigmatism or < 1.00 D for against‐the‐rule astigmatism, BCVA ≥ 20/20 in both eyes **Exclusion criteria:** contraindications of wearing OK, diagnosis of strabismus, amblyopia and other refractive development of the eye or systemic diseases, systemic condition which might affect refractive development (for example, Down syndrome, Marfan's syndrome), ocular conditions which might affect the refractive error (for example, cataract, ptosis)
Interventions	**Intervention:** OK lenses (5 mm optic zone) **Intervention:** OK lenses (5.5 mm optic zone) **Intervention:** OK lenses (6 mm optic zone) **Intervention:** OK lenses (6 mm optical zone and the increased height of peripheral reverse curve) **Comparator intervention:** SVLs
Outcomes	**Primary outcome:** change in AL, change in cycloplegic subjective refractive error **Secondary outcome:** change in visual questionnaire from baseline, change in high‐order aberrations, change in contrast sensitivity, change in choroidal thickness **Maximum follow up:** 24 months
Starting date	December 2021 **Estimated end date:** December 2025
Contact information	clinicaltrials.gov/ct2/show/results/NCT05192824
Notes

**Table:** PACT Study

Study name	Personalized addition lenses clinical trial
Methods	RCT
Participants	**Inclusion criteria:** 7‐12 years of age; myopic refractive error between −0.75 D and −4.00 D; cycloplegic spherical equivalent; astigmatism < 1.50 D; BCVA logMAR +0.05 or better in each; anisometropia < 1.00 D; at least 0.50 D progression by cycloplegic autorefraction over the past year **Exclusion criteria:** strabismus with or without add; ocular or systemic condition that may affect refractive error development
Interventions	**Intervention 1:** individualised add power **Intervention 2:** +2.00 D add power **Comparison intervention:** single vision
Outcomes	**Primary outcome:** change in cycloplegic SER error **Secondary outcome:** change in axial elongation **Maximum follow‐up:** 2 years
Starting date	July 2014 **Estimated end date:** March 2017
Contact information	Eye Hospital of Wenzhou Medical University
Notes	None

**Table:** Yuan 2021

Study name	Efficacy of combined orthokeratology and 0.01% atropine for myopia control: a randomized, controlled, double‐blind, and multicenter trial
Methods	Randomised parallel‐group design
Participants	**Inclusion criteria:** aged 8–12 years old; SER error between −1.00 and −4.00 D in either eye, astigmatism ≤ 1.50 D in either eye, BCVA of no worse than 25/25 in both eyes; birthweight was no less than 1500 g **Exclusion criteria:** patients with ocular disorders, such as strabismus, amblyopia, cataract, or ptosis; patients previously used OK lens or atropine eye drops to prevent myopia progression; patients with disorders contraindicated to atropine, such as known allergies, cardiovascular disease, or epilepsy; patients with disorders contraindicated to OK lens wear, such as ocular inflammation or infection; and patients with systemic disorders that might affect refractive development, such as Down syndrome or Marfan’s syndrome
Interventions	**Intervention:** OK lenses and atropine 0.01% eye drops **Comparison intervention:** OK lenses and placebo
Outcomes	**Primary outcome:** AL change from baseline **Secondary outcome:** change of pupil size and refraction from baseline, safety evaluated through the corneal endothelial cell and ocular surface function **Maximum follow up:** 24 months
Starting date	January 2019 **Estimated end date:** not reported
Contact information	www.chictr.org.cn/showproj.aspx?proj=29216
Notes	Trial registration: ChiCTR1800018419

**7‐mx:** 7‐methylxanthine; **AL:** axial length; **BCVA:** best corrected visual acuity; **BF:** bifocal; **CCLRU:** Cornea and Contact Lens Research Unit; **DIMS:** Defocus Incorporated Multiple Segments; **DS:** dioptre sphere; **ETDRS:** Early Treatment Diabetic Retinopathy Study; **EQ‐5D‐Y:** European Quality of Life‐5 Dimensions Youth questionnaire; **IOP:** intraocular pressure; **Kmax:** maximum keratometry; **MF:** multifocal; **MFSCL:** multifocus soft contact lens; **OK:** orthokeratology; **PAL:** progressive addition lens; **PREP:** Pediatric  Refractive Error Profile; **QoL:** quality of life; **RCT:** randomised controlled trial; **SER:** spherical equivalent refraction; **SVL:** single vision spectacle lens; **SVSCL:** single vision soft contact lens; **VA:** visual acuity

## Differences between protocol and review

We did not perform the generic search described in Electronic searches for adverse events, instead  we added a filter to the search strategy to identify systematic reviews of adverse events associated with myopia control interventions. These were discussed in the 'Agreements and disagreements with other studies or reviews' section of the Discussion

Since we mostly reported on direct (pairwise) evidence we used GRADE to assess our confidence in the estimates of effect rather than CINEMA as planned.

We used SUCRA to generate a relative ranking of myopia control interventions rather than mean rank values.
